# Publisher Correction: Evolution of sexual size dimorphism in tetrapods is driven by varying patterns of sex-specific selection on size

**DOI:** 10.1038/s41559-025-02635-5

**Published:** 2025-01-09

**Authors:** Alex Slavenko, Natalie Cooper, Shai Meiri, Gopal Murali, Daniel Pincheira-Donoso, Gavin H. Thomas

**Affiliations:** 1 Cesar Australia, Brunswick, Victoria Australia; 2 Natural History Museum London, London, UK; 3 School of Zoology, Tel Aviv University, Tel Aviv, Israel; 4 The Steinhardt Museum of Natural History, Tel Aviv, Israel; 5 Department of Ecology and Evolutionary Biology, University of Arizona, Tuscson, AZ USA; 6 MacroBiodiversity Lab, School of Biological Sciences, Queen’s University Belfast, Belfast, UK; 7 School of Biosciences, University of Sheffield, Sheffield, UK

**Keywords:** Evolution, Evolutionary ecology, Evolutionary ecology, Phylogenetics, Sexual selection

Correction to: *Nature Ecology & Evolution* 10.1038/s41559-024-02600-8. Published online 23 December 2024.

In the version of the article originally published, the credit lines for icons were missing in the legends for Extended Data Figs. 1 and 2. In addition, icons have been updated to match the credit lines and the icons in the main figures. The figures and legends have been amended in the HTML and PDF versions of the article.

