# Supplementary material for: Evolution of sexual size dimorphism in tetrapods is driven by varying patterns of sex-specific selection on size
Source: Nat Ecol Evol. 2024 Dec 23;9(3):464–73. doi: 10.1038/s41559-024-02600-8 (PMC11893467; doi:10.1038/s41559-024-02600-8)

nature ecology & evolution

Supplementary information https://doi.org/10.1038/s41559-024-02600-8

Evolution of sexual size dimorphism in tetrapods is driven by varying patterns of sex-specific selection on size

In the format provided by the authors and unedited

SUPPLEMENTARY MATERIAL

Clade	Sex	Model	Run	Marginal Likelihood	Mean Marginal Likelihood
**Mammals**	**Female**	BM	1	-2002.42	-2002.44
			2	-2002.47	
			3	-2002.43	
			4	-2002.45	
		OU	1	-2009.97	-2009.88
			2	-2009.76	
			3	-2009.87	
			4	-2009.93	
		Delta	1	-1999.94	-1999.93
			2	-1999.93	
			3	-1999.89	
			4	-1999.94	
		Evolve	1	-1835.29	-1834.54
			2	-1832.94	
			3	-1833.99	
			4	-1835.94	
		Evolve + Trend	1	-1819.82	-1820.783
			2	-1820.42	
			3	-1821.34	
			4	-1821.55	
		Fabric	1	-1794.22	-1789.42
			2	-1787.89	
			3	-1788.16	
			4	-1787.40	
		**Fabric + Trend**	**1**	**-1784.29**	**-1784.64**
			**2**	**-1783.47**	
			**3**	**-1787.84**	
			**4**	**-1782.97**	
	**Male**	BM	1	-2079.28	-2079.34
			2	-2079.40	
			3	-2079.32	
			4	-2079.35	
		OU	1	-2086.73	-2086.70
			2	-2086.60	
			3	-2086.67	
			4	-2086.78	
		Delta	1	-2077.32	-2077.35
			2	-2077.98	
			3	-2077.33	
			4	-2077.37	

		Evolve	1	-1910.93	-1910.47
			2	-1910.70	
			3	-1909.73	
			4	-1910.53	
		Evolve + Trend	1	-1893.11	-1895.57
			2	-1895.95	
			3	-1894.42	
			4	-1898.79	
		Fabric	1	-1848.21	-1851.57
			2	-1852.97	
			3	-1848.82	
			4	-1856.29	
		**Fabric + Trend**	**1**	**-1847.78**	**-1850.16**
			**2**	**-1851.50**	
			**3**	**-1856.04**	
			**4**	**-1845.34**	
**Birds**	**Female**	BM	1	-3231.57	-3231.54
			2	-3231.53	
			3	-3231.52	
			4	-3231.55	
		OU	1	-3221.49	-3221.78
			2	-3221.87	
			3	-3221.67	
			4	-3222.08	
		Delta	1	-3233.15	-3233.24
			2	-3233.25	
			3	-3233.18	
			4	-3233.38	
		Evolve	1	-2500.26	-2504.43
			2	-2501.93	
			3	-2510.35	
			4	-2505.19	
		Evolve + Trend	1	-2493.57	-2489.55
			2	-2486.44	
			3	-2489.31	
			4	-2488.88	
		Fabric	1	-2406.69	-2413.42
			2	-2420.91	
			3	-2414.14	
			4	-2411.93	
		**Fabric + Trend**	**1**	**-2395.03**	**-2401.09**
			**2**	**-2395.39**	
			**3**	**-2410.49**	
			**4**	**-2403.44**	
	**Male**	BM	1	-3392.73	-3392.87
			2	-3392.89	

			3	-3392.94	
			4	-3392.93	
		OU	1	-3378.82	-3378
			2	-3378.64	
			3	-3379.00	
			4	-3378.65	
		Delta	1	-3392.26	-3392.87
			2	-3392.78	
			3	-3393.67	
			4	-3392.77	
		Evolve	1	-2558.75	-2558.48
			2	-2557.81	
			3	-2560.01	
			4	-2557.33	
		Evolve + Trend	1	-2538.42	-2542.39
			2	-2550.00	
			3	-2540.73	
			4	-2540.41	
		Fabric	1	-2466.35	-2407.23
			2	-2469.17	
			3	-2479.90	
			4	-2465.52	
		**Fabric + Trend**	**1**	**-2457.13**	**-2461.25**
			**2**	**-2461.88**	
			**3**	**-2463.06**	
			**4**	**-2462.91**	
**Squamates**	**Female**	BM	1	-4200.92	-4200.86
			2	-4200.83	
			3	-4200.86	
			4	-4200.81	
		OU	1	-4134.82	-4134.61
			2	-4134.79	
			3	-4134.86	
			4	-4133.94	
		Delta	1	-4166.55	-4166.28
			2	-4166.15	
			3	-4167.37	
			4	-4165.02	
		Evolve	1	-3741.30	-3739.39
			2	-3738.21	
			3	-3738.22	
			4	-3739.83	
		Evolve + Trend	1	-3734.33	-3733.21
			2	-3737.26	
			3	-3726.78	
			4	-3734.46	

		Fabric	1	-3653.57	-3655.79
			2	-3657.70	
			3	-3655.21	
			4	-3656.67	
		**Fabric + Trend**	**1**	**-3656.96**	**-3653.93**
			**2**	**-3652.04**	
			**3**	**-3654.36**	
			**4**	**-3652.36**	
	**Male**	BM	1	-4326.30	-4326.25
			2	-4326.21	
			3	-4326.25	
			4	-4326.25	
		OU	1	-4257.55	-4257.58
			2	-4257.27	
			3	-4257.29	
			4	-4258.20	
		Delta	1	-4290.07	-4290.21
			2	-4289.82	
			3	-4290.54	
			4	-4290.42	
		Evolve	1	-3908.44	-3907.81
			2	-3907.32	
			3	-3908.46	
			4	-3907.03	
		Evolve + Trend	1	-3904.82	-3907.25
			2	-3909.32	
			3	-3908.26	
			4	-3906.60	
		Fabric	1	-3802.06	-3826.74
			2	-3894.38	
			3	-3805.35	
			4	-3805.18	
		**Fabric + Trend**	**1**	**-3804.18**	**-3804.79**
			**2**	**-3809.54**	
			**3**	**-3807.57**	
			**4**	**-3797.88**	
**Amphibians**	**Female**	BM	1	-3965.26	-3965.26
			2	-3965.26	
			3	-3965.28	
			4	-3965.25	
		OU	1	-3781.73	-3781.85
			2	-3781.54	
			3	-3782.23	
			4	-3781.89	
		Delta	1	-3916.99	-3916.86
			2	-3917.14	

			3	-3917.02	
			4	-3916.29	
		Evolve	1	-3332.12	-3332.58
			2	-3332.29	
			3	-3332.58	
			4	-3333.33	
		Evolve + Trend	1	-3333.82	-3335.48
			2	-3334.78	
			3	-3333.29	
			4	-3340.04	
		Fabric	1	-3258.47	-3255.6
			2	-3252.94	
			3	-3253.46	
			4	-3257.52	
		**Fabric + Trend**	**1**	**-3253.97**	**-3255.57**
			**2**	**-3252.63**	
			**3**	**-3257.00**	
			**4**	**-3258.69**	
	**Male**	BM	1	-3727.09	-3727.09
			2	-3727.08	
			3	-3727.09	
			4	-3727.11	
		OU	1	-3606.10	-3606.07
			2	-3606.21	
			3	-3606.04	
			4	-3605.94	
		Delta	1	-3690.15	-3690.35
			2	-3690.71	
			3	-3690.87	
			4	-3689.67	
		Evolve	1	-3282.39	-3282.66
			2	-3281.72	
			3	-3281.51	
			4	-3285.03	
		Evolve + Trend	1	-3285.90	-3284.91
			2	-3284.62	
			3	-3285.74	
			4	-3283.38	
		Fabric	1	-3218.39	-3217.33
			2	-3214.76	
			3	-3216.52	
			4	-3219.66	
		**Fabric + Trend**	**1**	**-3218.22**	**-3216.88**
			**2**	**-3214.60**	
			**3**	**-3217.05**	
			**4**	**-3217.67**	

Table S1. Marginal likelihoods of the BayesTraits models (Brownian Motion [BM], Ornstein-Uhlenbeck (OU), accelerating and decelerating evolution (Delta), Evolvability (Evolve), Evolvability with Global Trend (Evolve + Trend), Fabric, and Fabric with Global Trend [Fabric + Trend]), estimated using a stepping-stone sampler. The best model for each sex of each clade, chosen by the highest mean marginal likelihood across four runs, is highlighted in bold.

**SSD**	**Contrast**	**n**	**Estimate**	**CI low**	**CI high**	***p***	***p*-adj**
	**Within Sexes**						
**Female-biased**	Female	52	0.44	0.30	0.59	0.488	0.651
	**Male**	**49**	**0.76**	**0.61**	**0.87**	**<0.001**	**0.003**
Monomorphic	Female	109	0.50	0.41	0.60	1.000	1.000
	Male	104	0.59	0.49	0.68	0.095	0.182
**Male-biased**	Female	138	0.41	0.33	0.50	0.050	0.120
	**Male**	**149**	**0.32**	**0.25**	**0.40**	**<0.001**	**<0.001**
	**Between Sexes**						
**Female-biased**	Decreasing	60	0.38	0.26	0.52	0.092	0.182
	**Increasing**	**41**	**0.71**	**0.54**	**0.84**	**0.012**	**0.039**
Monomorphic	Decreasing	116	0.47	0.38	0.57	0.643	0.771
	Increasing	97	0.56	0.45	0.66	0.310	0.493
Male-biased	Decreasing	105	0.54	0.44	0.64	0.435	0.602
	Increasing	182	0.45	0.37	0.52	0.159	0.272

Table S2. Results of binomial tests comparing the number of increasing and decreasing directional changes within and between sexes for mammals. Under column contrast is the group within which the comparison is made - e.g. for contrast “Female” (in within sexes comparisons) the comparison is between number of decreasing and increasing changes, for contrast “Increasing” (in between sexes comparisons) the comparison is between number of female and male changes. n is the number of trials (all changes within the contrast group), estimate is the estimated probability of success (i.e. selecting decreasing or female, in within- and between-sexes comparisons, respectively), CI low and high are the 95% confidence intervals around the estimate, *p* is the p-value for a two-sided null

hypothesis of probability of success = 0.5, and *p*-adj is the p-value adjusted for multiple comparisons using a Benjamini-Hochberg false discovery rate (FDR) correction. Statistically significant comparisons (*p*-adj <0.05) are in bold.

**SSD**	**Contrast**	**n**	**Estimate**	**CI low**	**CI high**	***p***	***p*-adj**
	**Within Sexes**						
**Female-biased**	Female	82	0.44	0.33	0.55	0.320	0.493
	**Male**	**80**	**0.65**	**0.54**	**0.75**	**0.010**	**0.036**
**Monomorphic**	**Female**	**322**	**0.42**	**0.36**	**0.47**	**0.003**	**0.015**
	Male	334	0.44	0.39	0.50	0.043	0.114
**Male-biased**	Female	167	0.47	0.40	0.55	0.536	0.677
	**Male**	**167**	**0.39**	**0.31**	**0.47**	**0.005**	**0.021**
	**Between Sexes**						
Female-biased	Decreasing	88	0.41	0.31	0.52	0.109	0.194
	Increasing	74	0.62	0.50	0.73	0.047	0.120
Monomorphic	Decreasing	282	0.48	0.42	0.54	0.439	0.602
	Increasing	374	0.50	0.45	0.55	0.959	1.000
Male-biased	Decreasing	144	0.55	0.46	0.63	0.279	0.461
	Increasing	190	0.46	0.39	0.54	0.346	0.503

Table S3. Results of binomial tests comparing the number of increasing and decreasing directional changes within and between sexes for birds. Under column contrast is the group within which the comparison is made - e.g. for contrast “Increasing” (in within sexes comparisons) the comparison is between number of female and male changes, for contrast “female” (in between sexes comparisons) the comparison is between number of decreasing and increasing changes. n is the number of trials (all changes within the contrast group), estimate is the estimated probability of success (i.e. selecting female or decreasing, in within- and between-sexes comparisons, respectively), CI low and high are the 95% confidence intervals around the estimate, *p* is the p-value for a two-sided null hypothesis of probability of success = 0.5, and *p*-adj is the p-value adjusted for multiple comparisons using a

Benjamini-Hochberg false discovery rate (FDR) correction. Statistically significant comparisons (*p*-adj <0.05) are in bold.

**SSD**	**Contrast**	**n**	**Estimate**	**CI low**	**CI high**	***p***	***p*-adj**
	**Within Sexes**						
**Female-biased**	Female	194	0.48	0.41	0.56	0.720	0.785
	**Male**	**199**	**0.69**	**0.62**	**0.76**	**<0.001**	**<0.001**
Monomorphic	Female	156	0.52	0.44	0.60	0.689	0.783
	Male	151	0.54	0.46	0.62	0.329	0.493
**Male-biased**	Female	180	0.50	0.42	0.58	1.000	1.000
	**Male**	**197**	**0.23**	**0.17**	**0.29**	**<0.001**	**<0.001**
	**Between Sexes**						
**Female-biased**	**Decreasing**	**232**	**0.41**	**0.34**	**0.47**	**0.005**	**0.020**
	**Increasing**	**161**	**0.62**	**0.54**	**0.70**	**0.003**	**0.014**
Monomorphic	Decreasing	163	0.50	0.42	0.58	1.000	1.000
	Increasing	144	0.52	0.44	0.60	0.677	0.783
**Male-biased**	**Decreasing**	**135**	**0.67**	**0.58**	**0.75**	**<0.001**	**0.001**
	**Increasing**	**242**	**0.37**	**0.31**	**0.44**	**<0.001**	**<0.001**

Table S4. Results of binomial tests comparing the number of increasing and decreasing directional changes within and between sexes for squamates. Under column contrast is the group within which the comparison is made - e.g. for contrast “Increasing” (in within sexes comparisons) the comparison is between number of female and male changes, for contrast “female” (in between sexes comparisons) the comparison is between number of decreasing and increasing changes. n is the number of trials (all changes within the contrast group), estimate is the estimated probability of success (i.e. selecting female or decreasing, in within- and between-sexes comparisons, respectively), CI low and high are the 95% confidence intervals around the estimate, *p* is the p-value for a two-sided null hypothesis of probability of success = 0.5, and *p*-adj is the p-value adjusted for

multiple comparisons using a Benjamini-Hochberg false discovery rate (FDR) correction. Statistically significant comparisons (*p*-adj <0.05) are in bold.

**SSD**	**Contrast**	**n**	**Estimate**	**CI low**	**CI high**	***p***	***p*-adj**
	**Within Sexes**						
**Female-biased**	Female	403	0.55	0.50	0.60	0.073	0.263
	**Male**	**403**	**0.58**	**0.53**	**0.63**	**0.002**	**0.012**
Monomorphic	Female	40	0.68	0.51	0.81	0.038	0.109
	Male	39	0.36	0.21	0.53	0.108	0.194
**Male-biased**	Female	27	0.56	0.35	0.75	0.701	0.783
	**Male**	**32**	**0.13**	**0.04**	**0.29**	**<0.001**	**<0.001**
	**Between Sexes**						
Female-biased	Decreasing	453	0.49	0.44	0.53	0.573	0.705
	Increasing	353	0.52	0.46	0.57	0.523	0.677
Monomorphic	Decreasing	41	0.66	0.49	0.80	0.060	0.136
	Increasing	38	0.34	0.20	0.51	0.073	0.152
Male-biased	Decreasing	19	0.79	0.54	0.94	0.019	0.058
	Increasing	40	0.30	0.17	0.47	0.017	0.053

Table S5. Results of binomial tests comparing the number of increasing and decreasing directional changes within and between sexes for amphibians. Under column contrast is the group within which the comparison is made - e.g. for contrast "Increasing" (in within sexes comparisons) the comparison is between number of female and male changes, for contrast "female" (in between sexes comparisons) the comparison is between number of decreasing and increasing changes. n is the number of trials (all changes within the contrast group), estimate is the estimated probability of success (i.e. selecting female or decreasing, in within- and between-sexes comparisons, respectively), CI low and high are the 95% confidence intervals around the estimate, *p* is the p-value for a two-sided null hypothesis of probability of success = 0.5, and *p*-adj is the p-value adjusted for

multiple comparisons using a Benjamini-Hochberg false discovery rate (FDR) correction. Statistically significant comparisons (*p*-adj <0.05) are in bold.

Supporting Information S2

Each page contains a phylogeny, showing a single clade subset from the full class phylogeny. Branches are coloured by SSD. Nodes where directional changes have been reconstructed on the branches leading to them are marked by triangles - upward facing representing increasing size, and downward facing representing decreasing size. Blue triangles (left of the node) represent changes in female size, and red triangles (right of the node) represent changes in male size.

Full description of how directional changes were located and estimated can be found in the Methods section.

Mammals

Afrosoricida

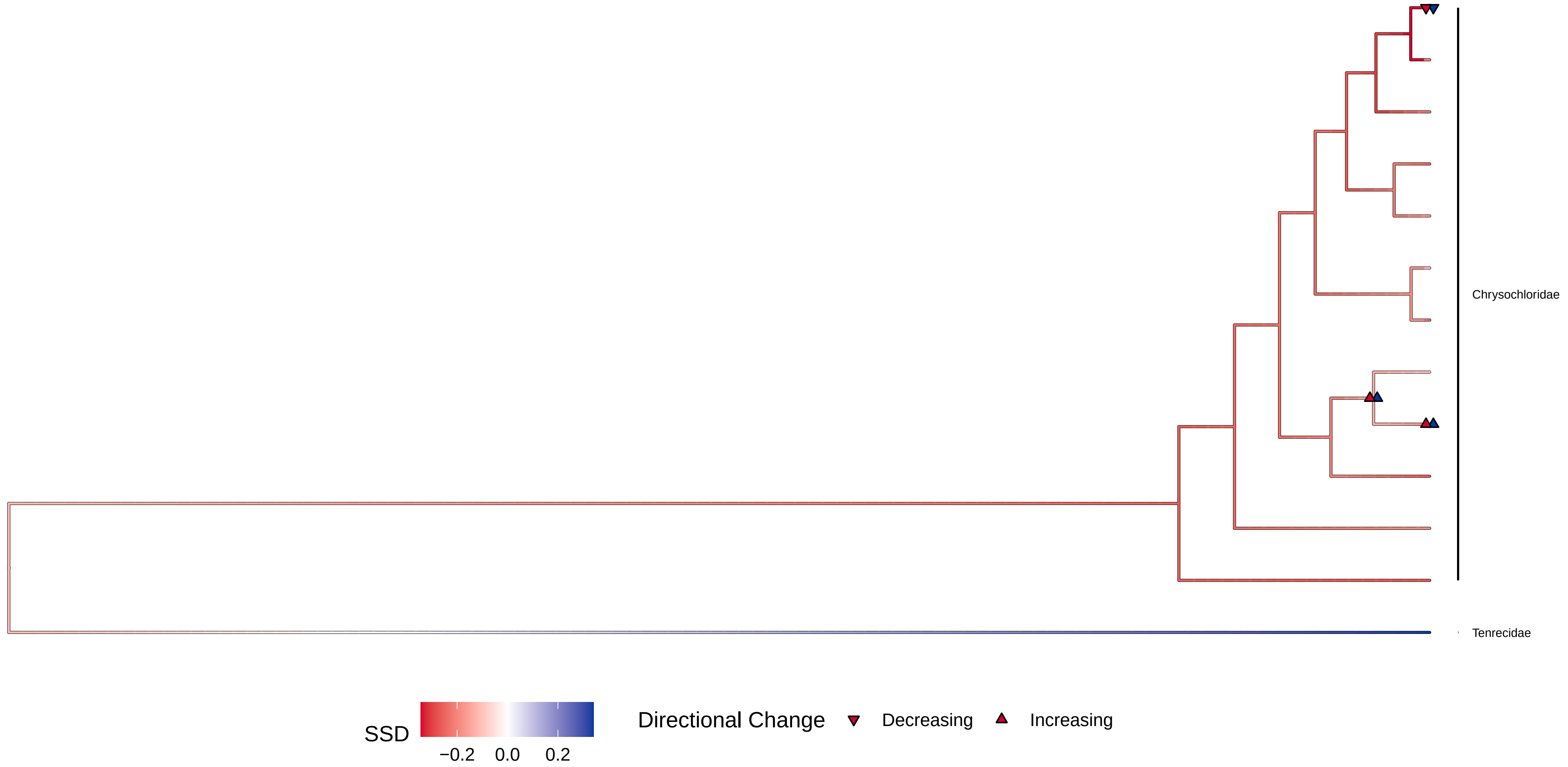

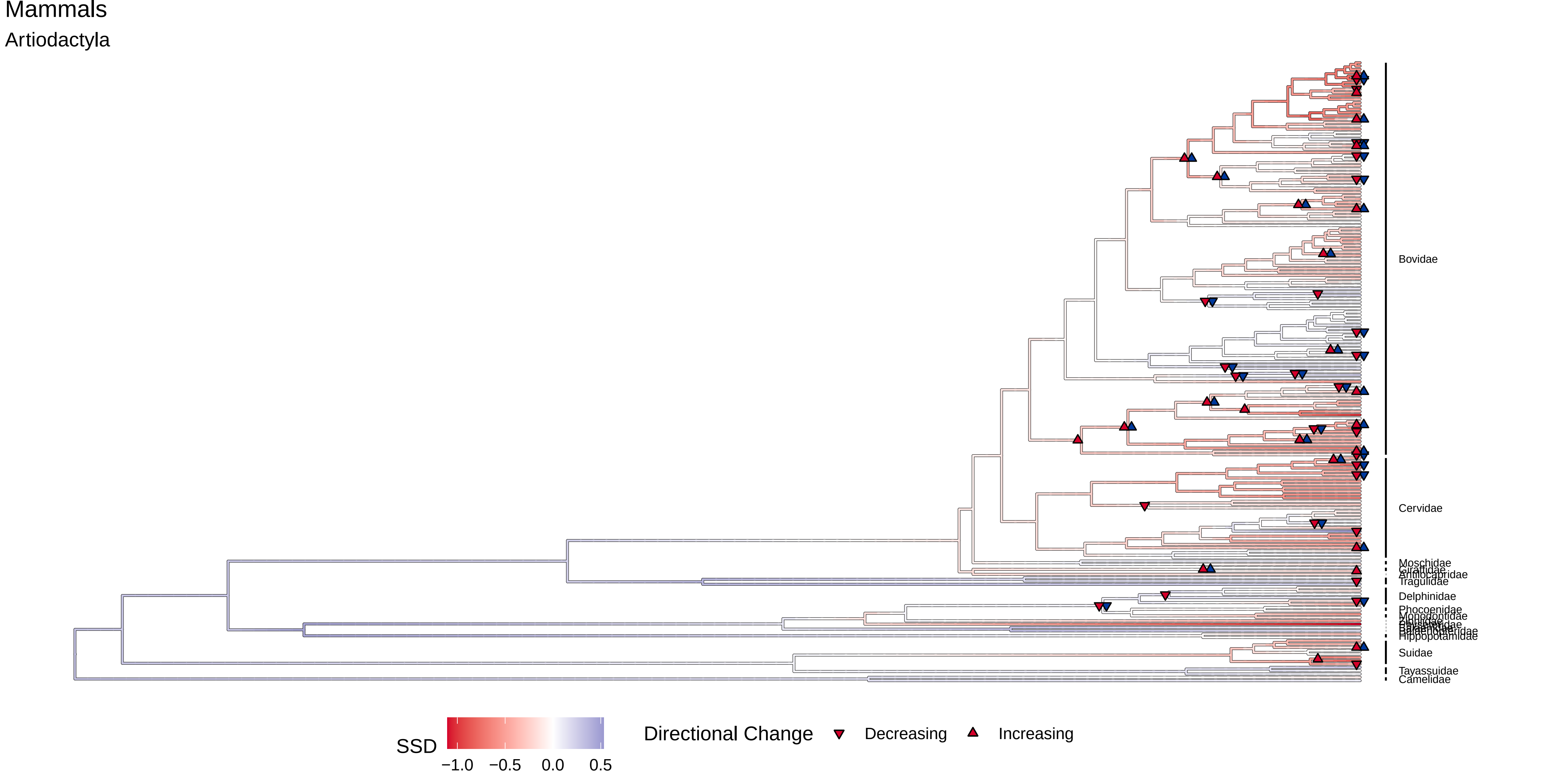
Mammals
Artiodactyla
Bovidae
Cervidae
Moschidae
Tragulidae
Delphinidae
Phocoenidae
Hippopotamidae
Suidae
Tayassuidae
Camelidae
SSD
−1.0
−0.5
0.0
0.5
Directional Change
Decreasing
Increasing

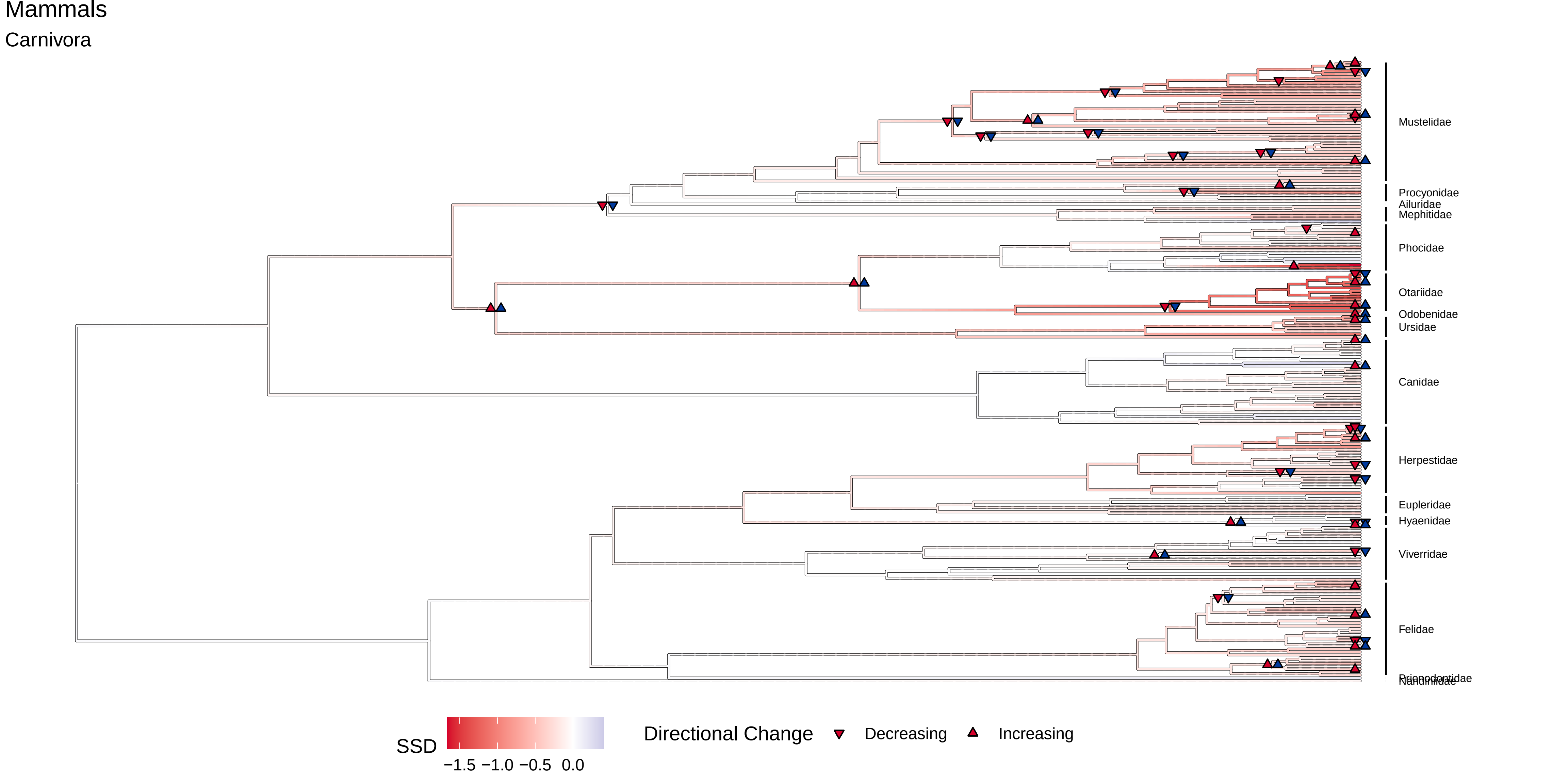

Mammals
Carnivora
Mustelidae
Procyonidae
Ailuridae
Mephitidae
Phocidae
Otariidae
Odobenidae
Ursidae
Canidae
Herpestidae
Eupleridae
Hyaenidae
Viverridae
Felidae
Prionodontidae
Nandiniidae
SSD
−1.5 −1.0 −0.5 0.0
Directional Change
Decreasing
Increasing

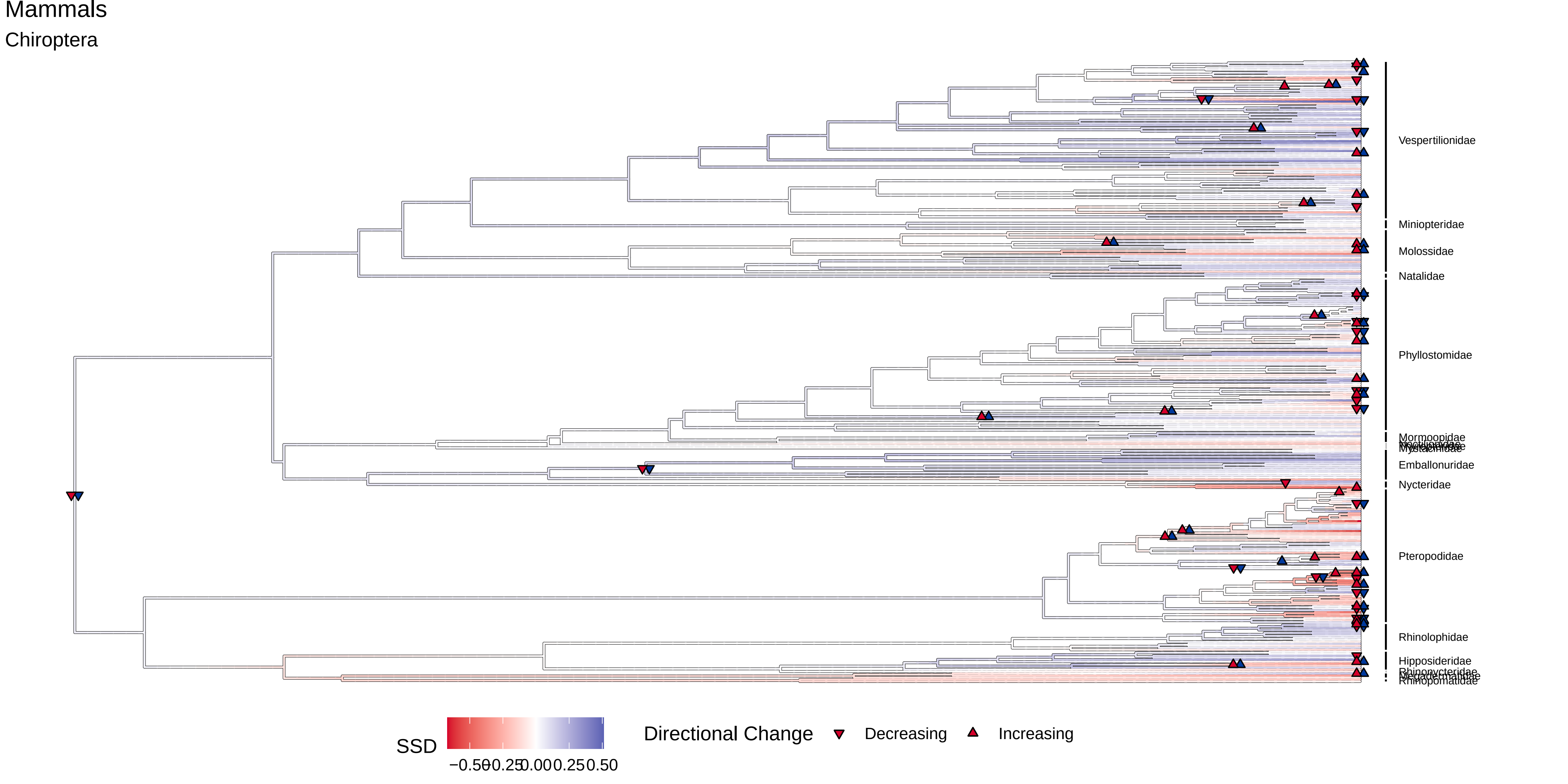

Mammals
Chiroptera
Vespertilionidae
Miniopteridae
Molossidae
Natalidae
Phyllostomidae
Mormoopidae
Emballonuridae
Nycteridae
Pteropodidae
Rhinolophidae
Hipposideridae
Rhinopomatidae
SSD
−0.50
−0.25
0.00
0.25
0.50
Directional Change
Decreasing
Increasing

Mammals

Dasyuromorphia

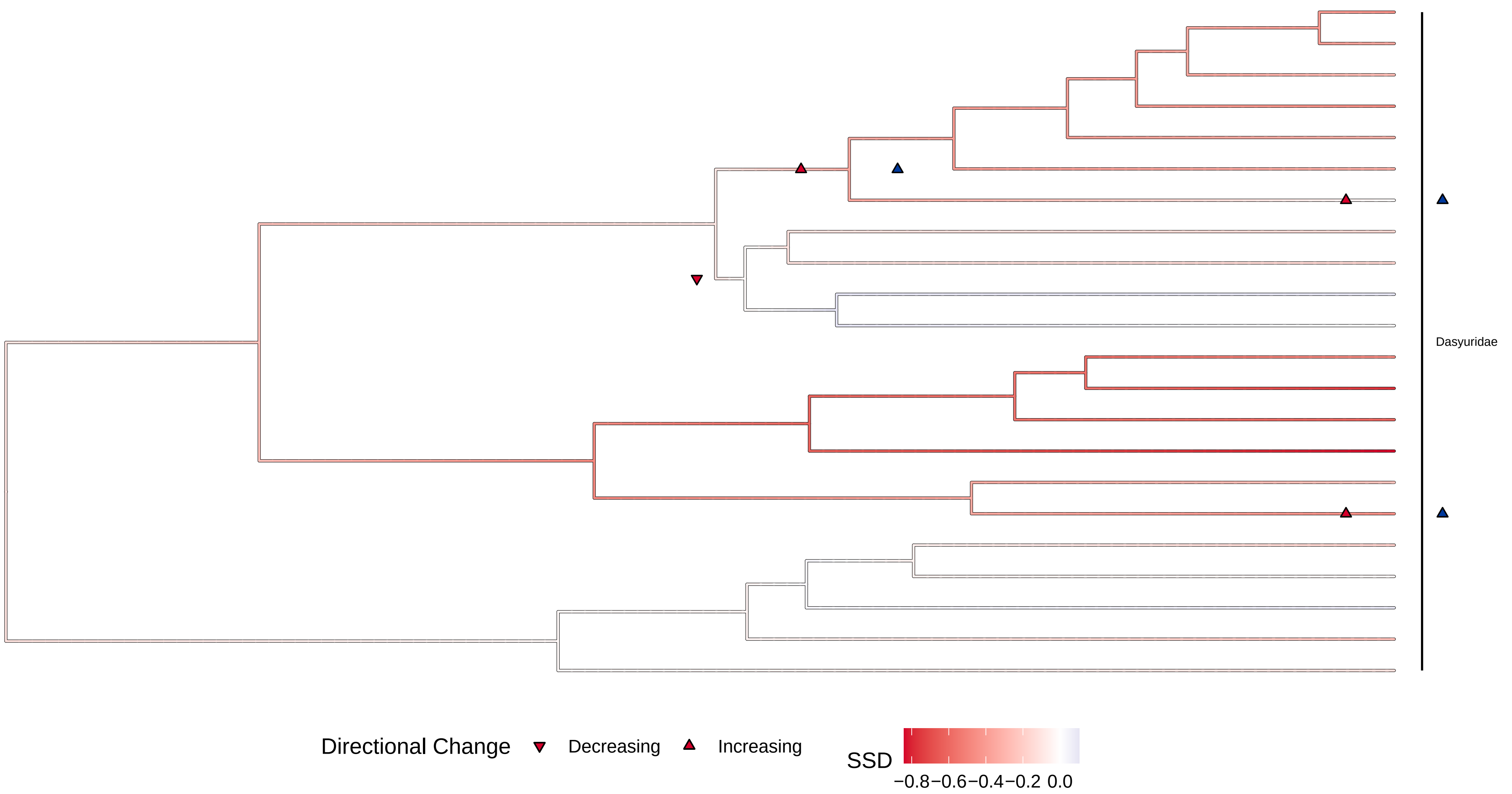

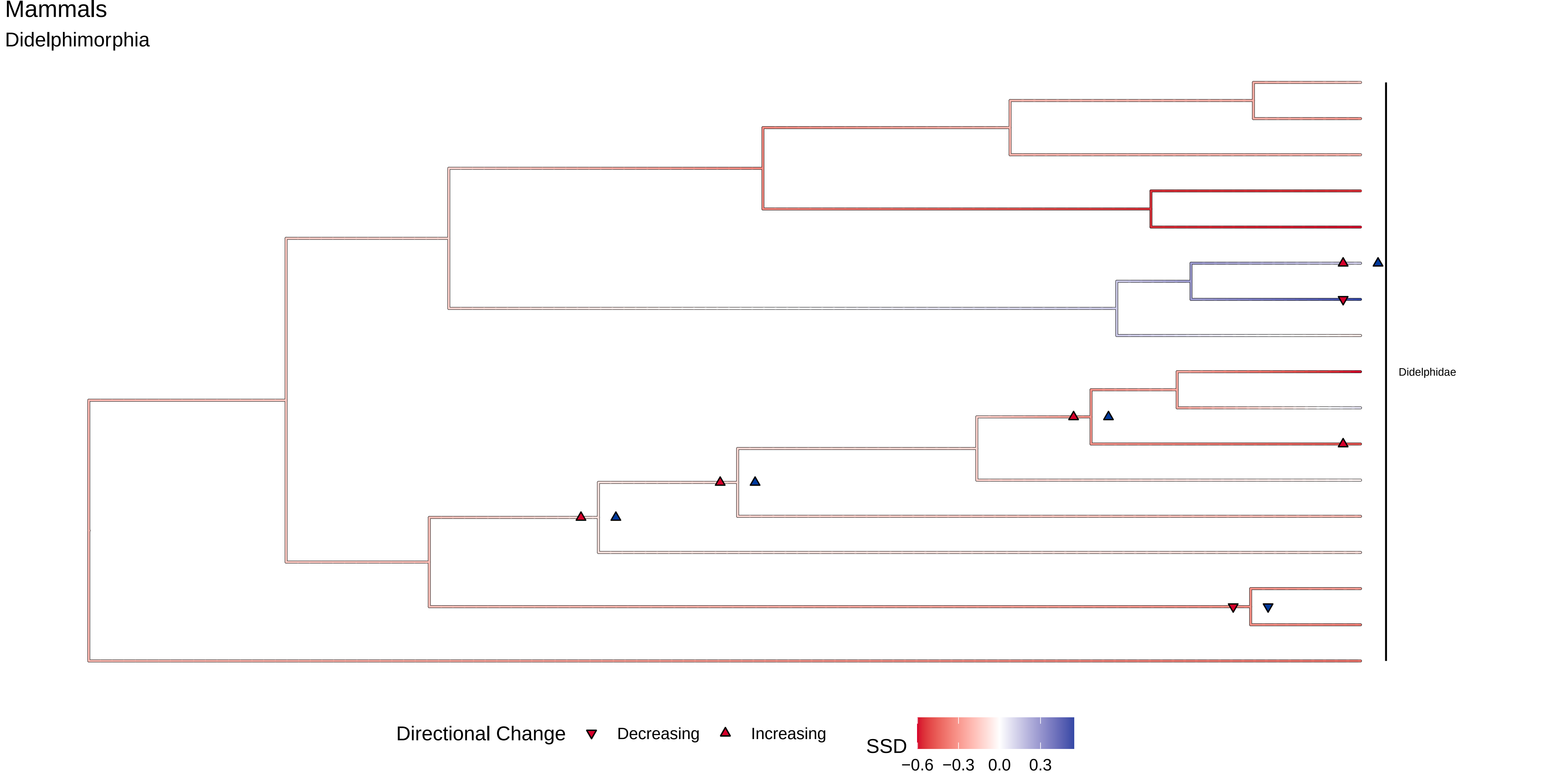
Mammals
Didelphimorphia
Didelphidae
Directional Change
Decreasing
Increasing
SSD
−0.6 −0.3 0.0 0.3

Mammals

Diprotodontia

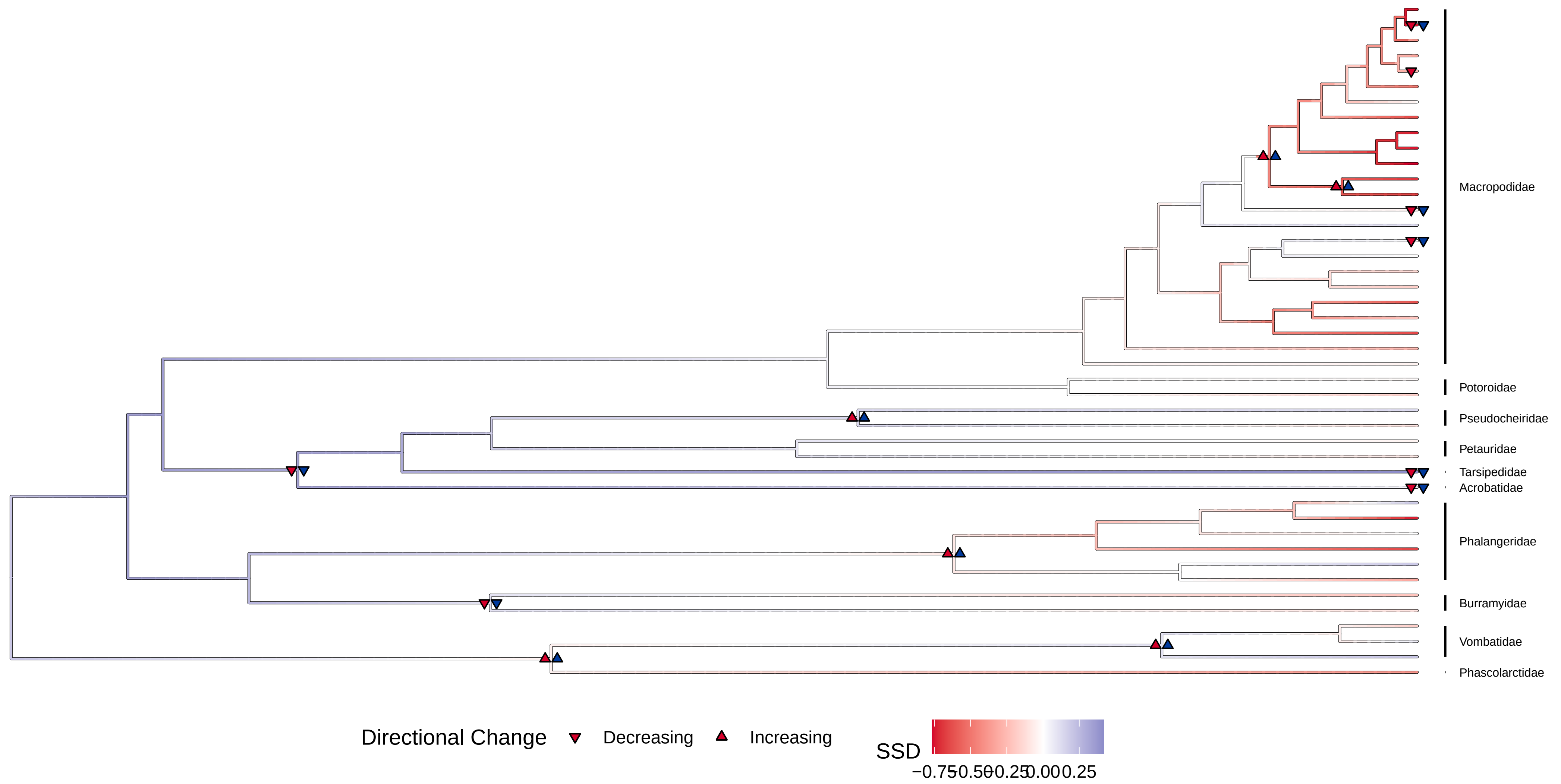

Mammals

Eulipotyphla

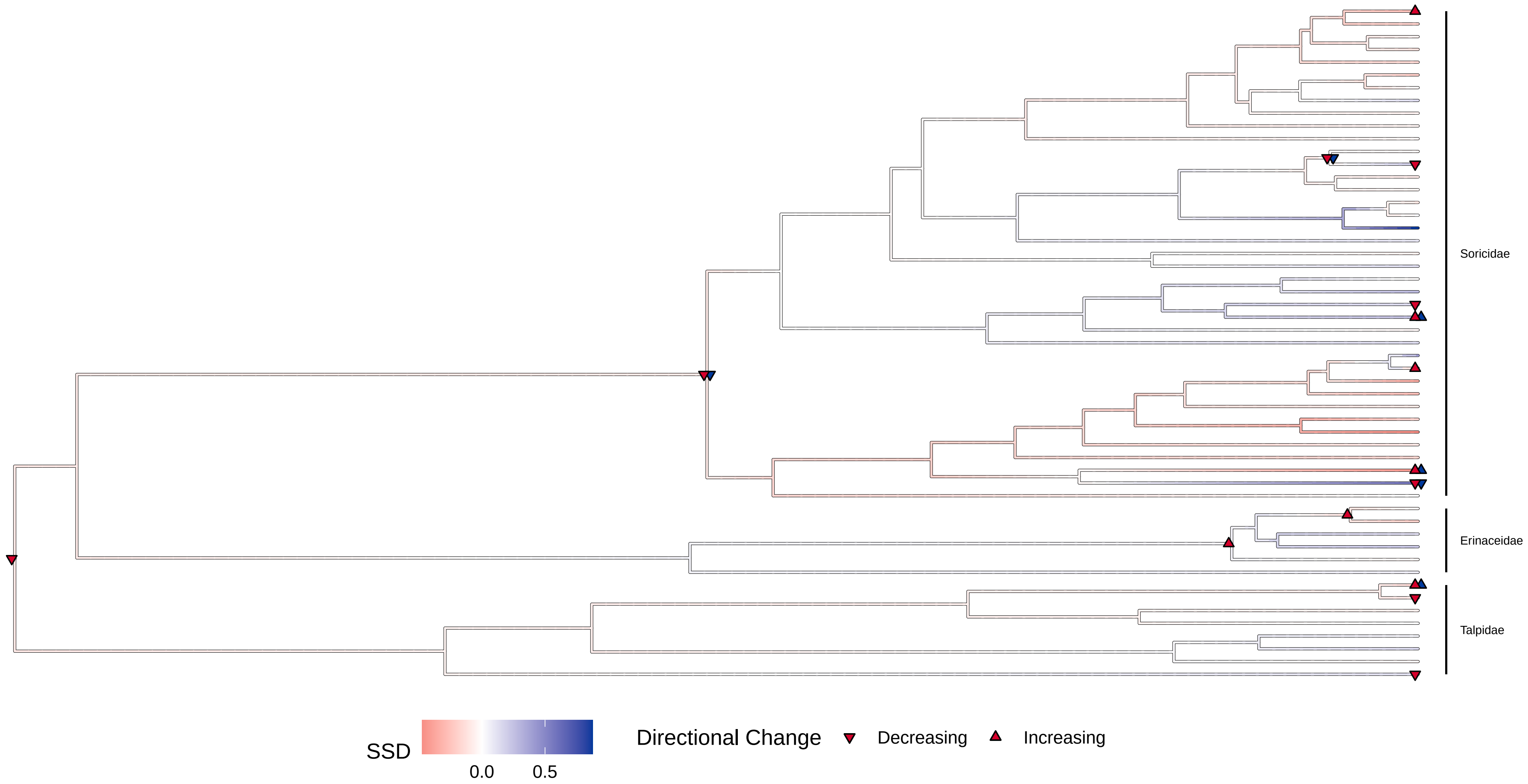

Mammals

Hyracoidea

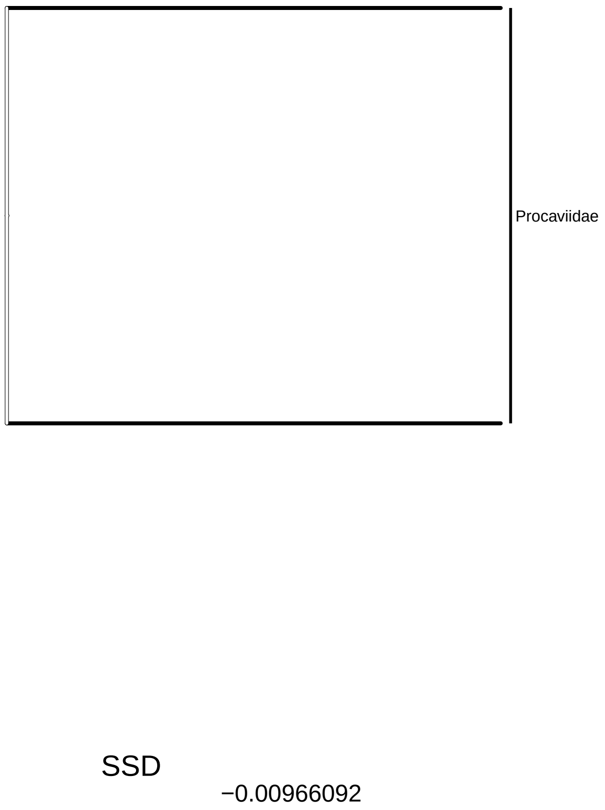

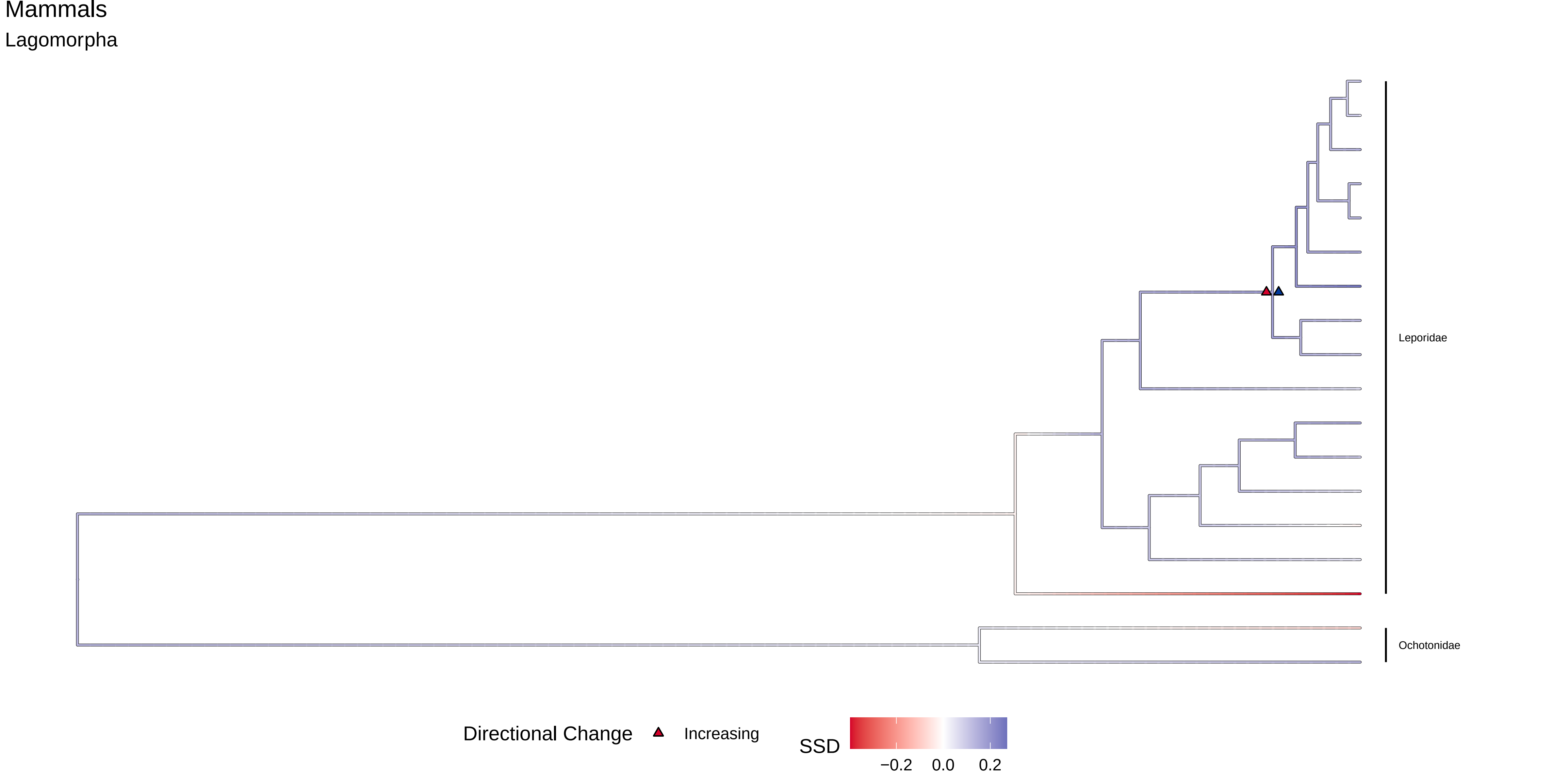
Mammals
Lagomorpha
Leporidae
Ochotonidae
Directional Change
Increasing
SSD
−0.2
0.0
0.2

Mammals

Macroscelidea

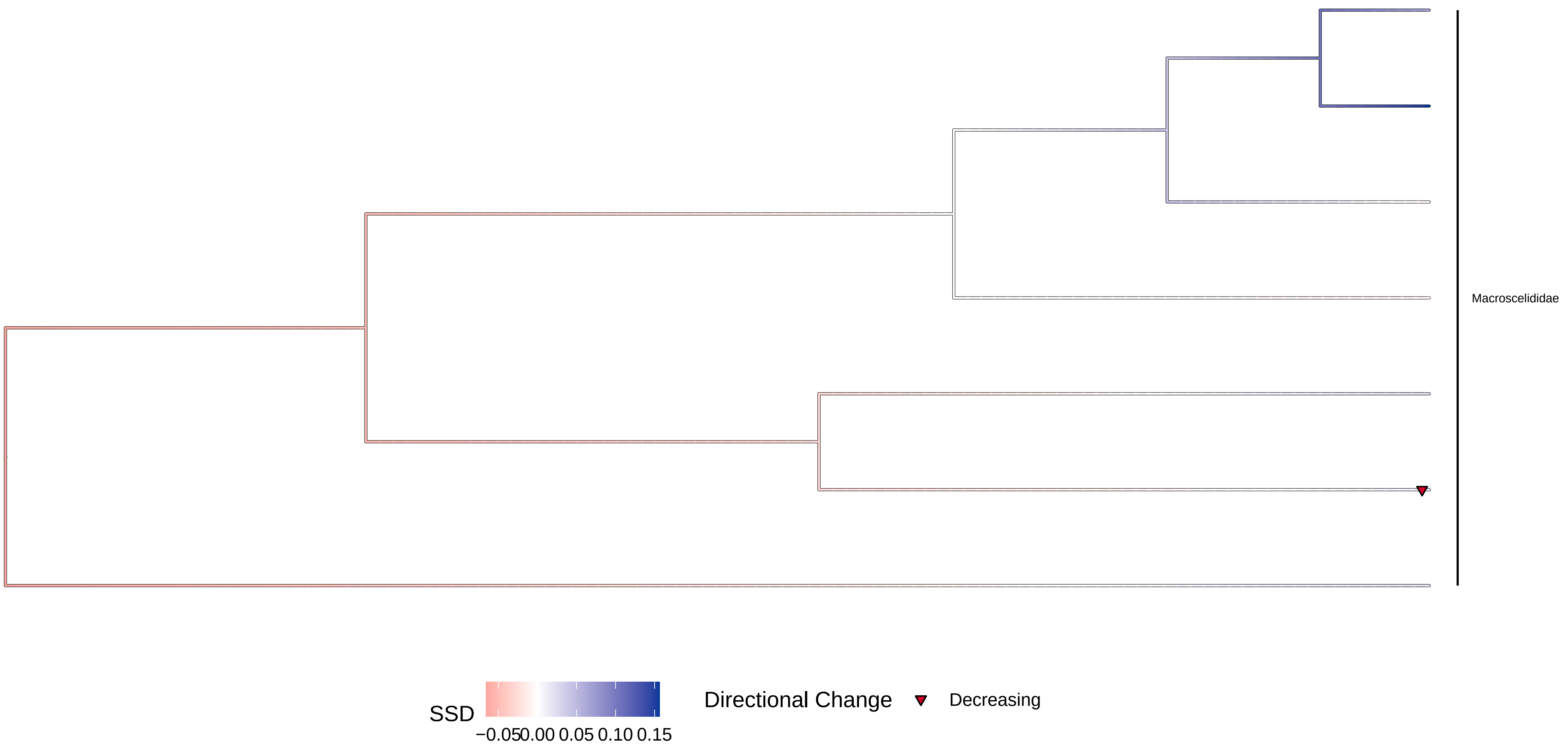

Mammals

Monotremata

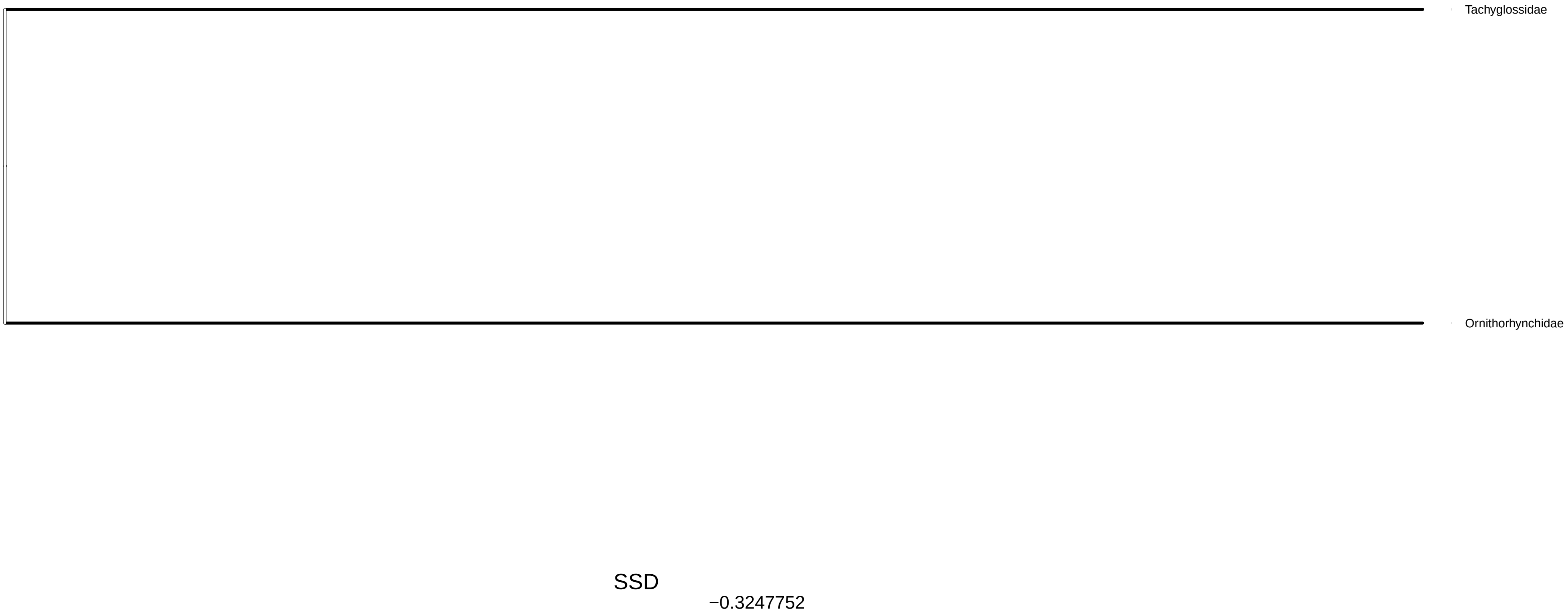

Mammals

Paucituberculata

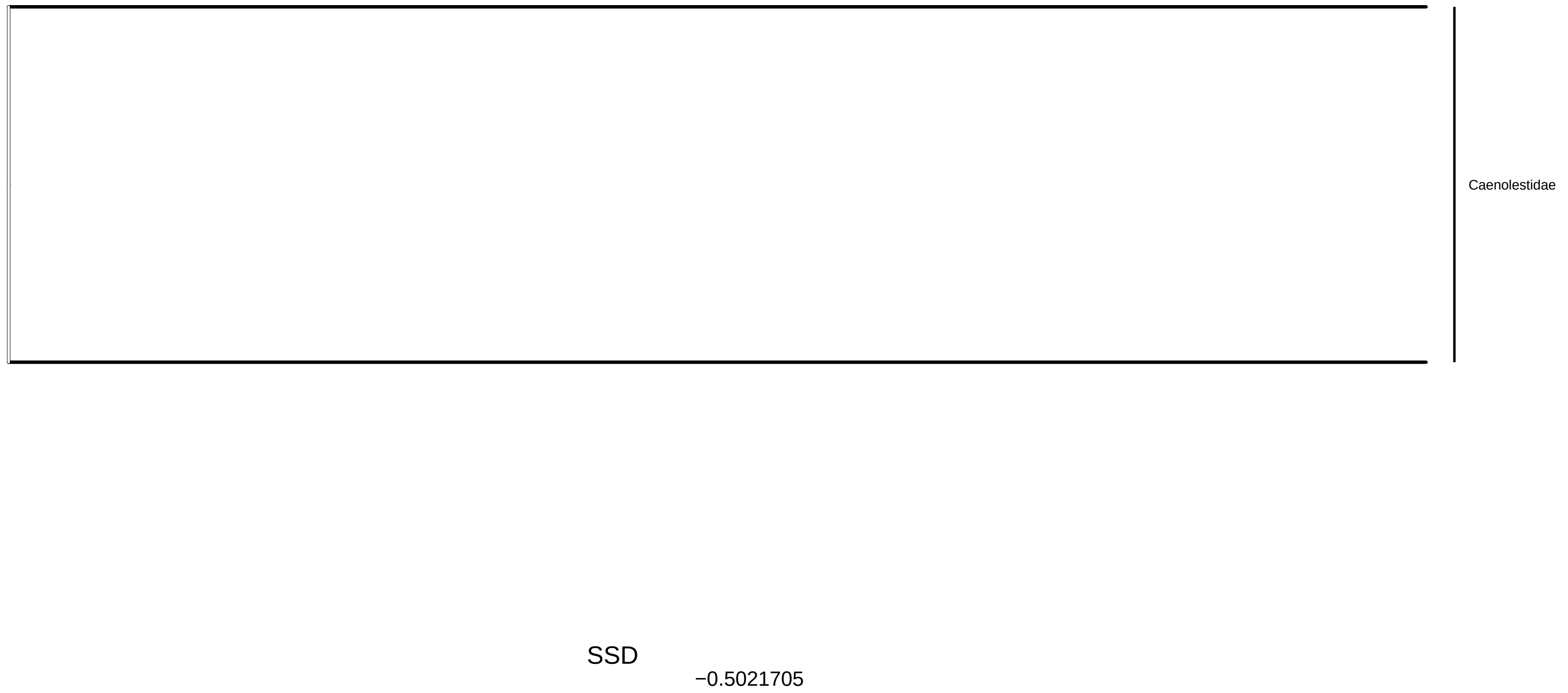

Mammals

Peramelemorphia

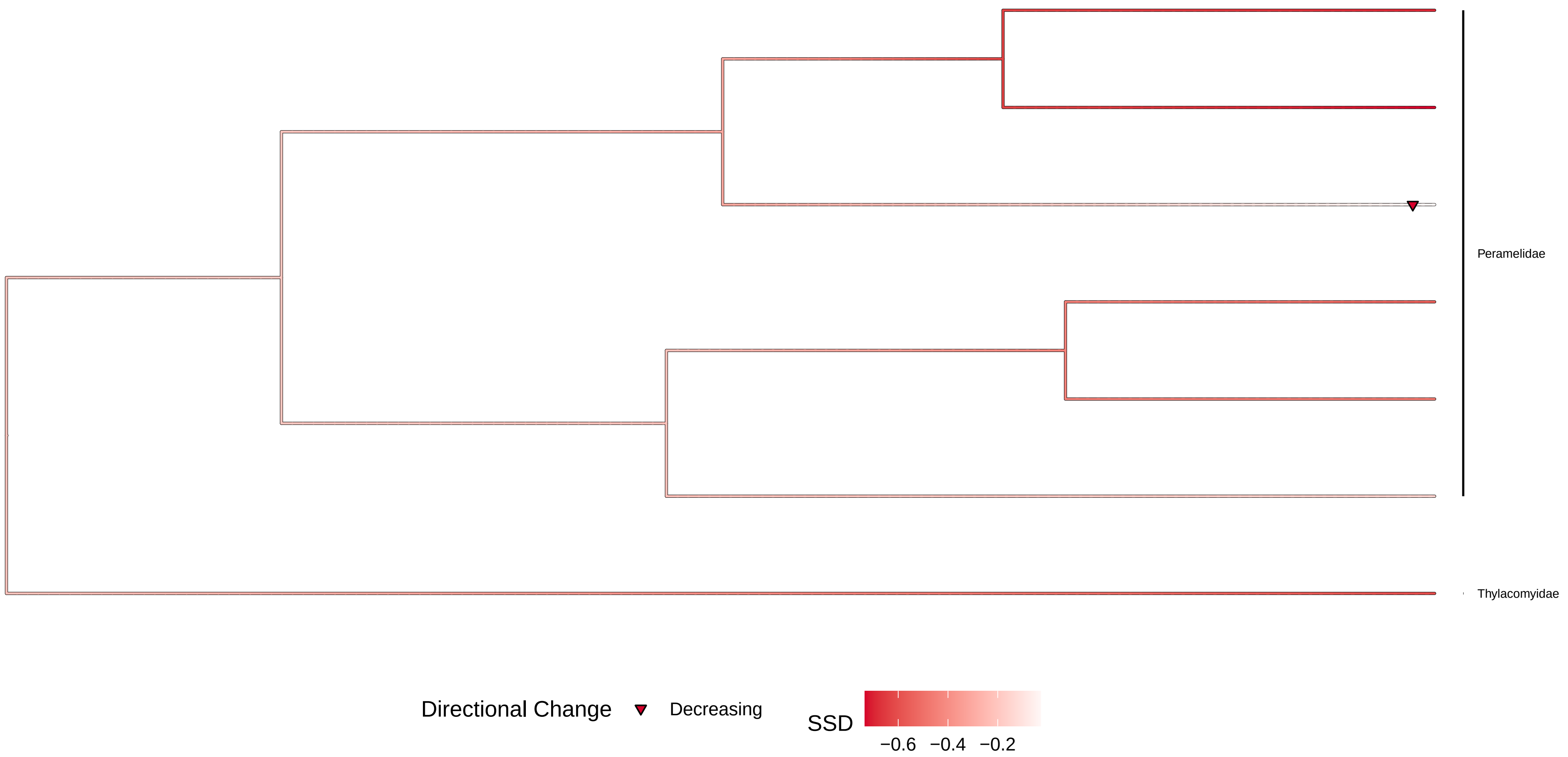

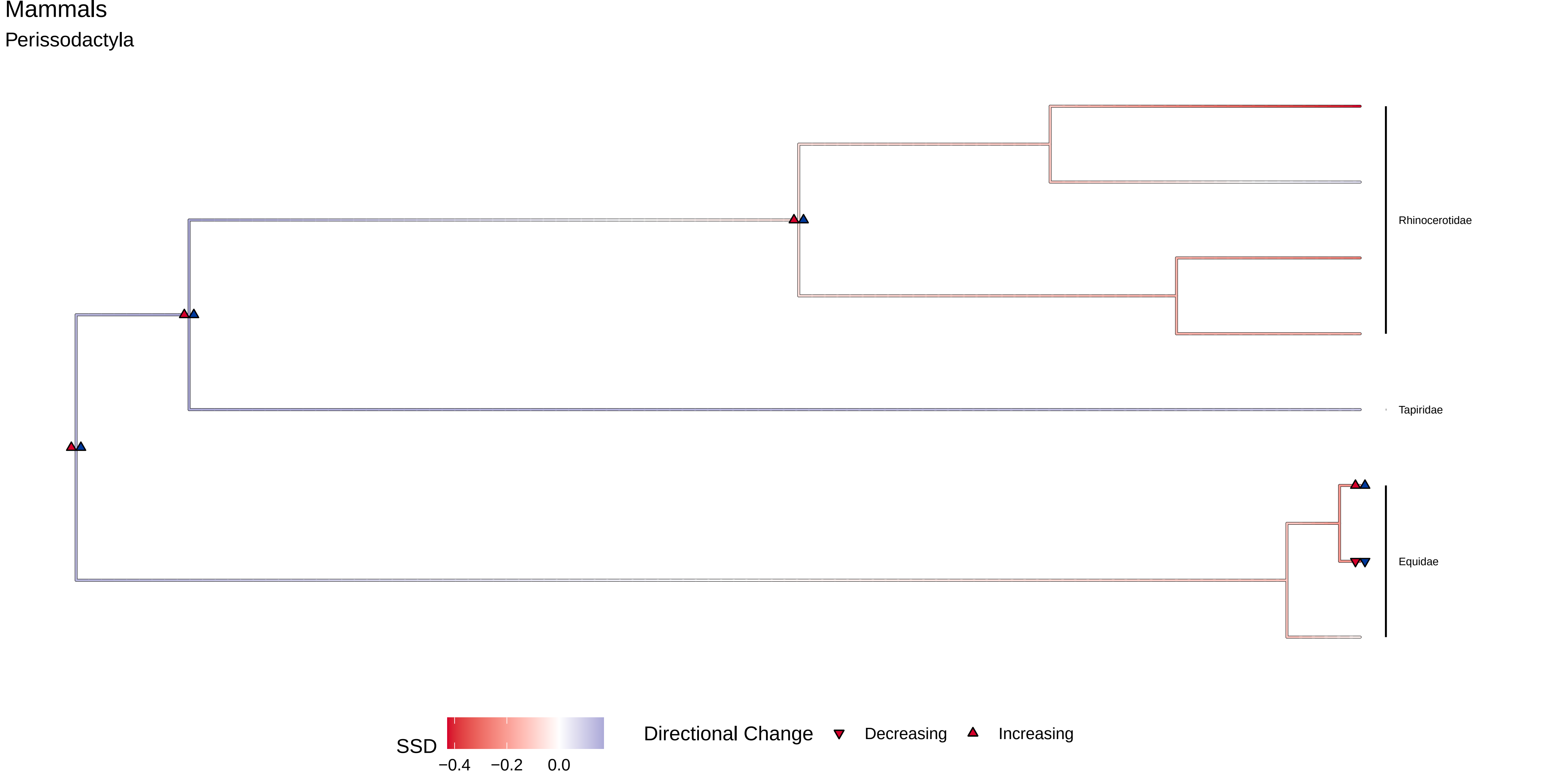

Mammals
Perissodactyla
Rhinocerotidae
Tapiridae
Equidae
SSD
−0.4
−0.2
0.0
Directional Change
Decreasing
Increasing

Mammals

Pholidota

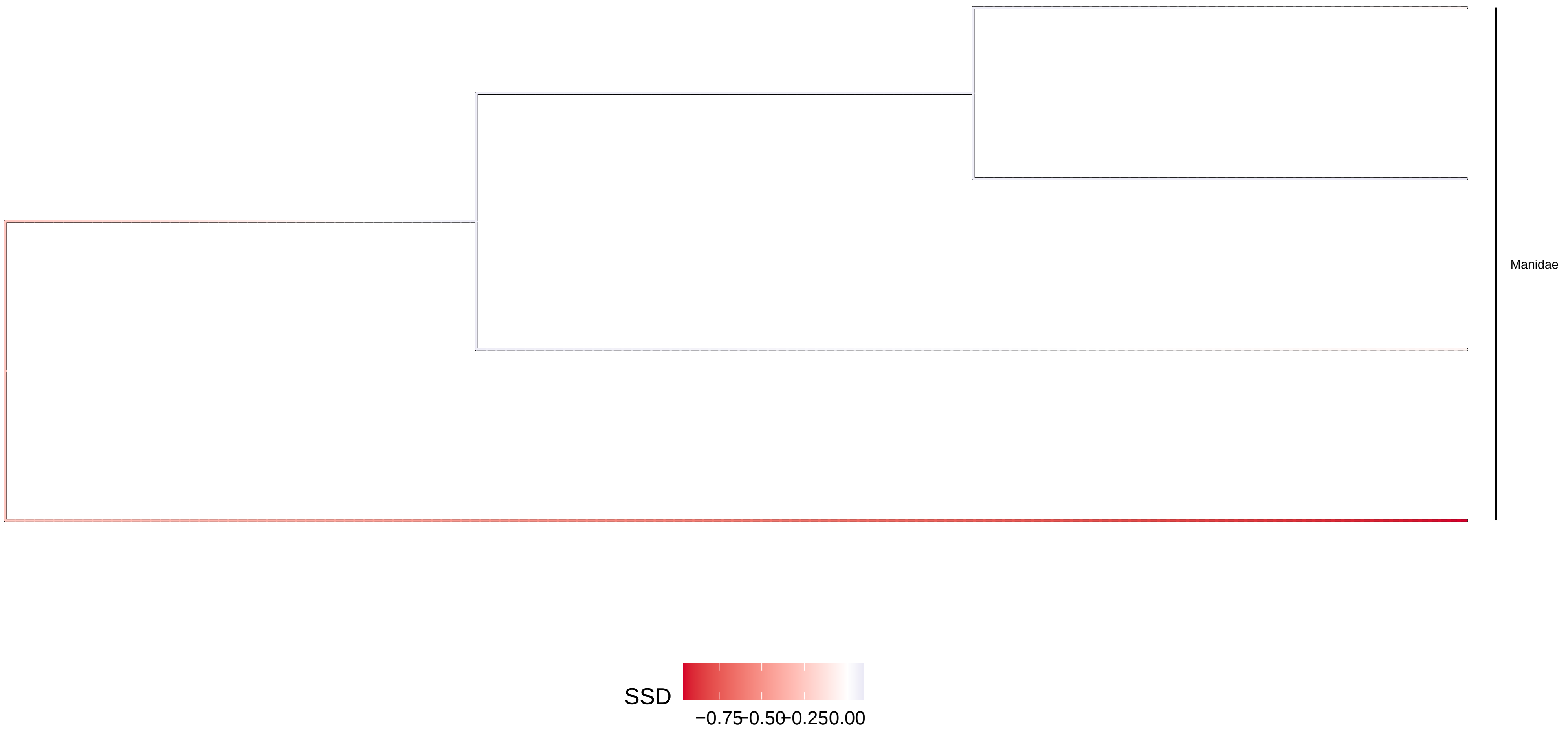

Mammals

Pilosa

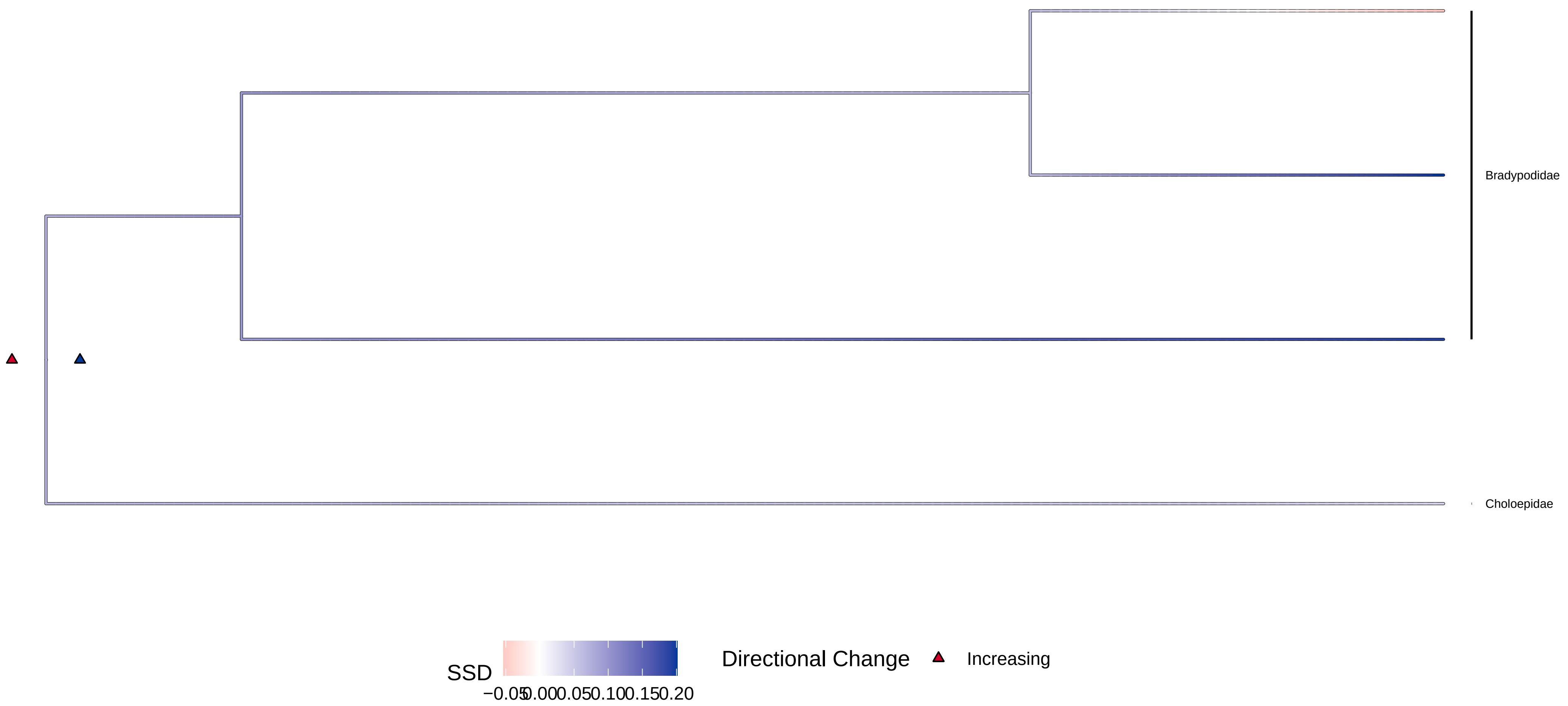

Mammals

Primates

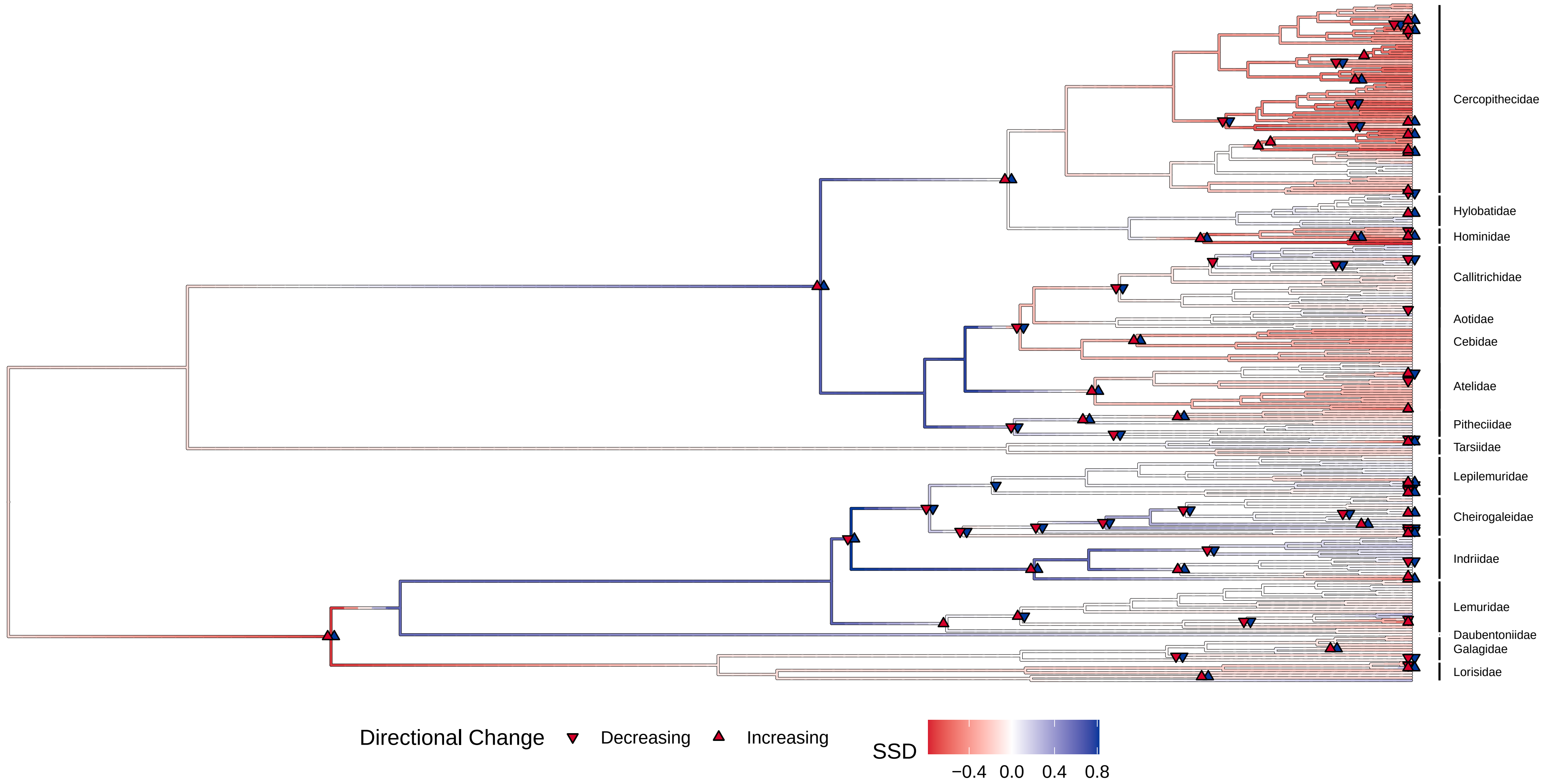

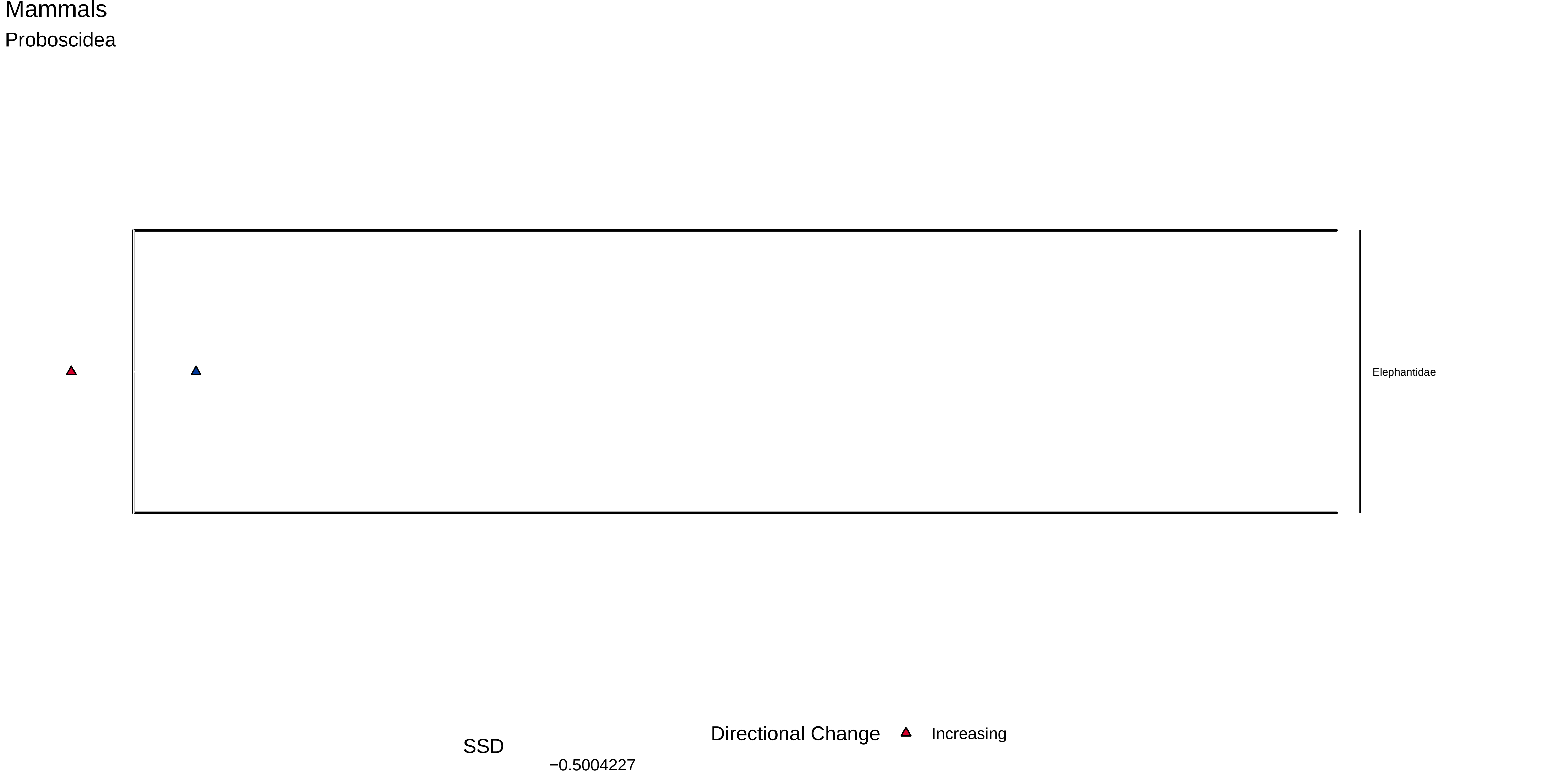

Mammals
Proboscidea
Elephantidae
SSD
−0.5004227
Directional Change
Increasing

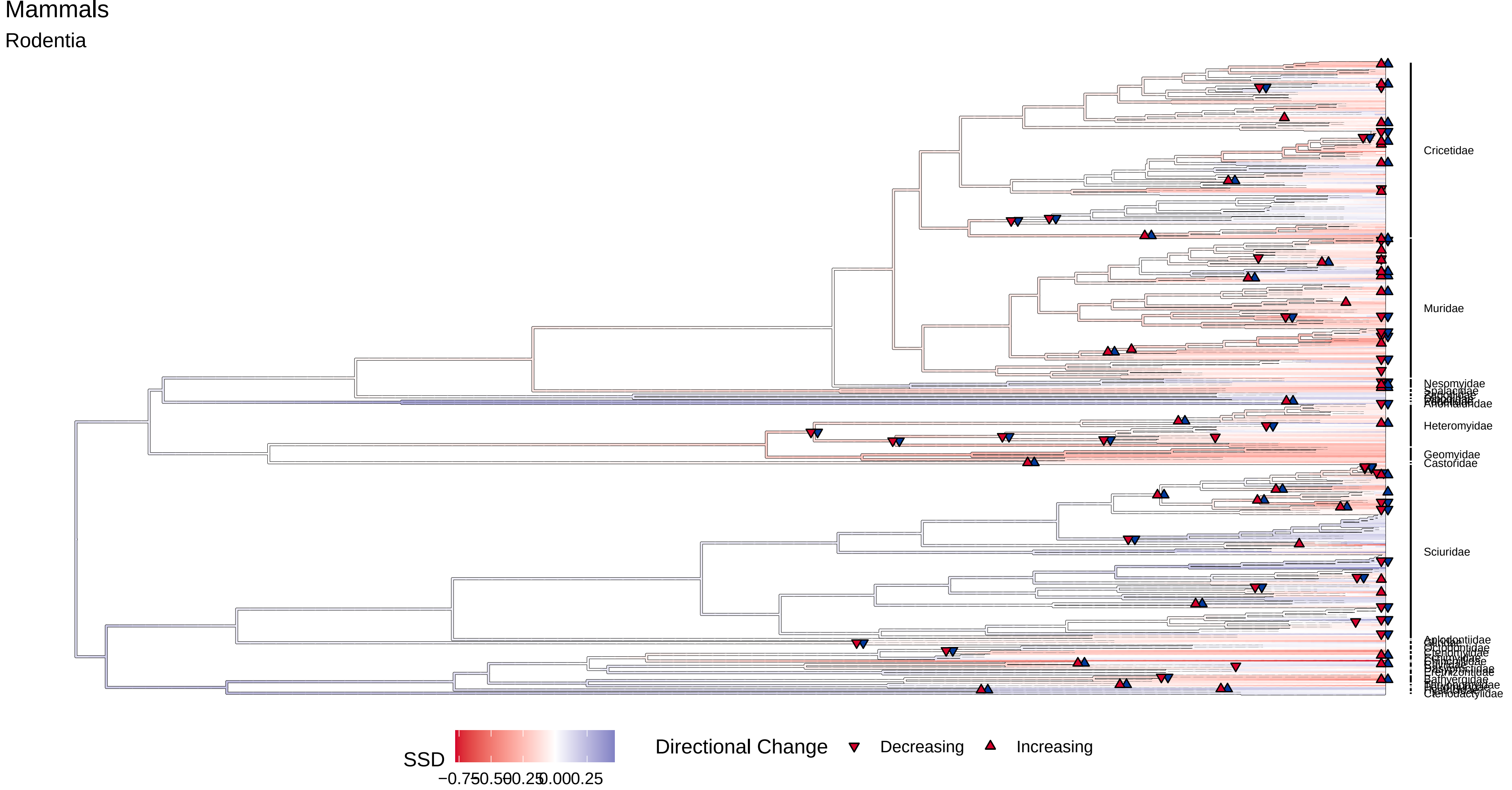
Mammals
Rodentia
Cricetidae
Muridae
Nesomyidae
Heteromyidae
Geomyidae
Castoridae
Sciuridae
Ctenodactylidae
SSD
Directional Change
Decreasing
Increasing

Birds

Accipitriformes

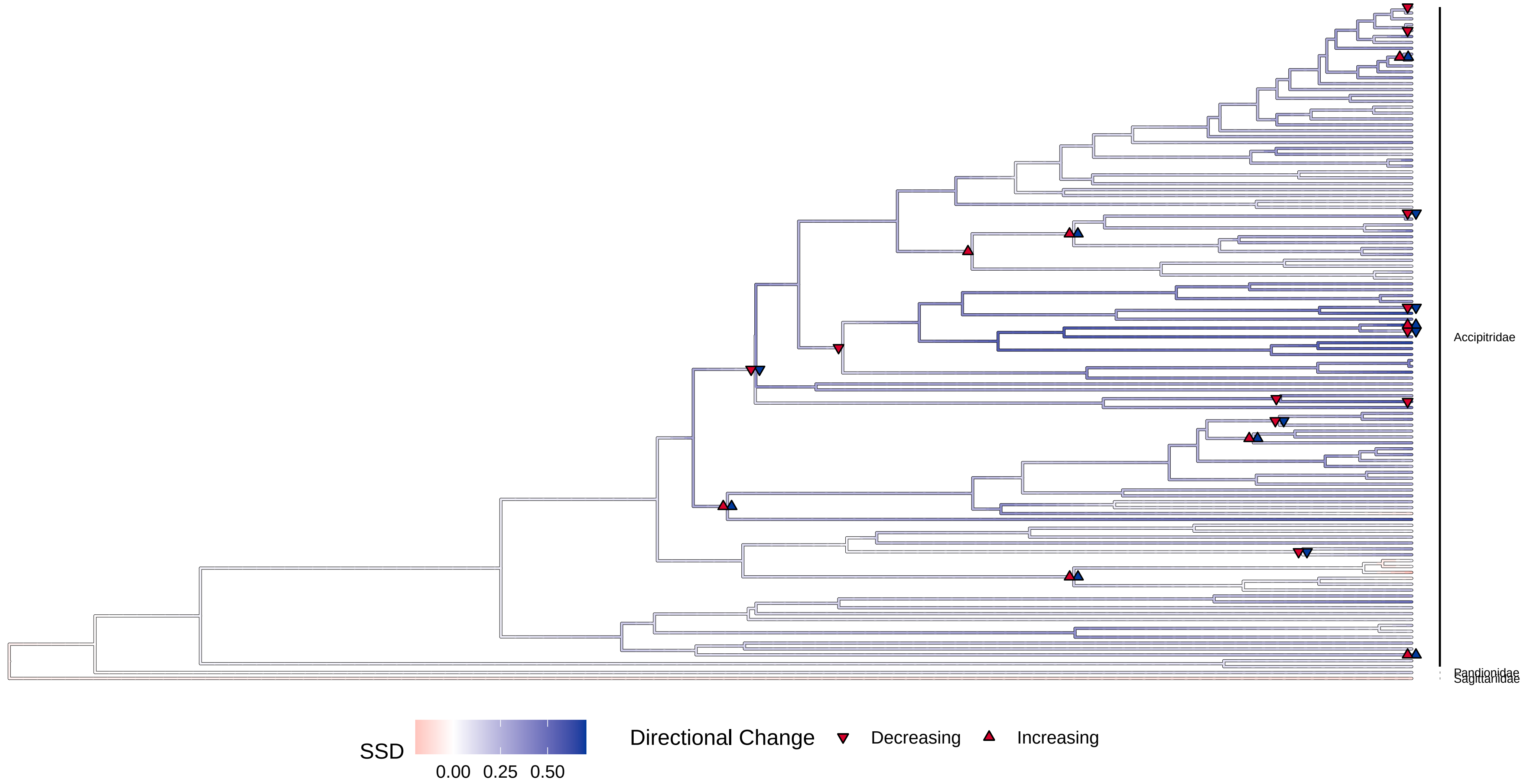

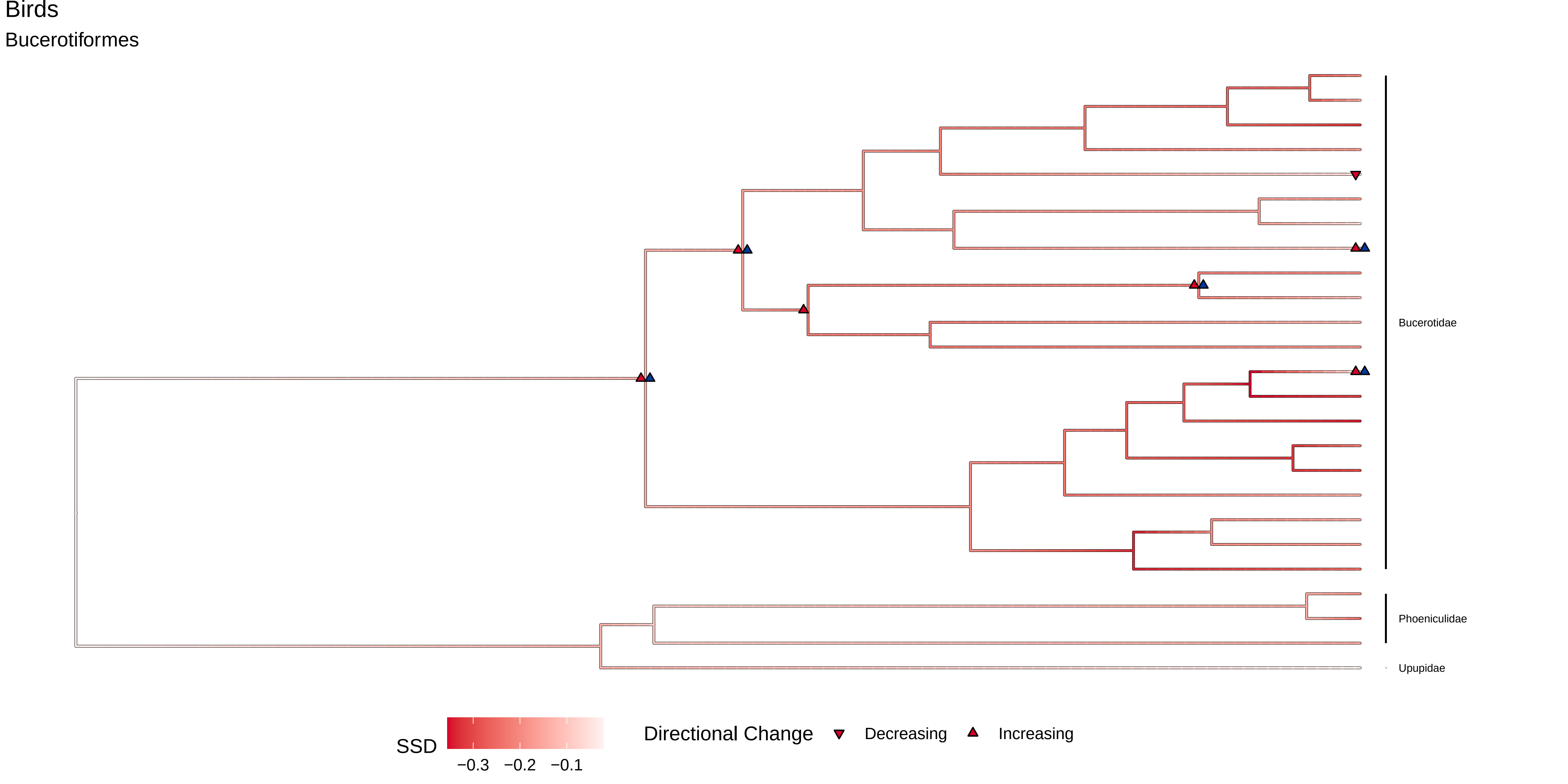
Birds
Bucerotiformes
Bucerotidae
Phoeniculidae
Upupidae
SSD
−0.3 −0.2 −0.1
Directional Change
Decreasing
Increasing

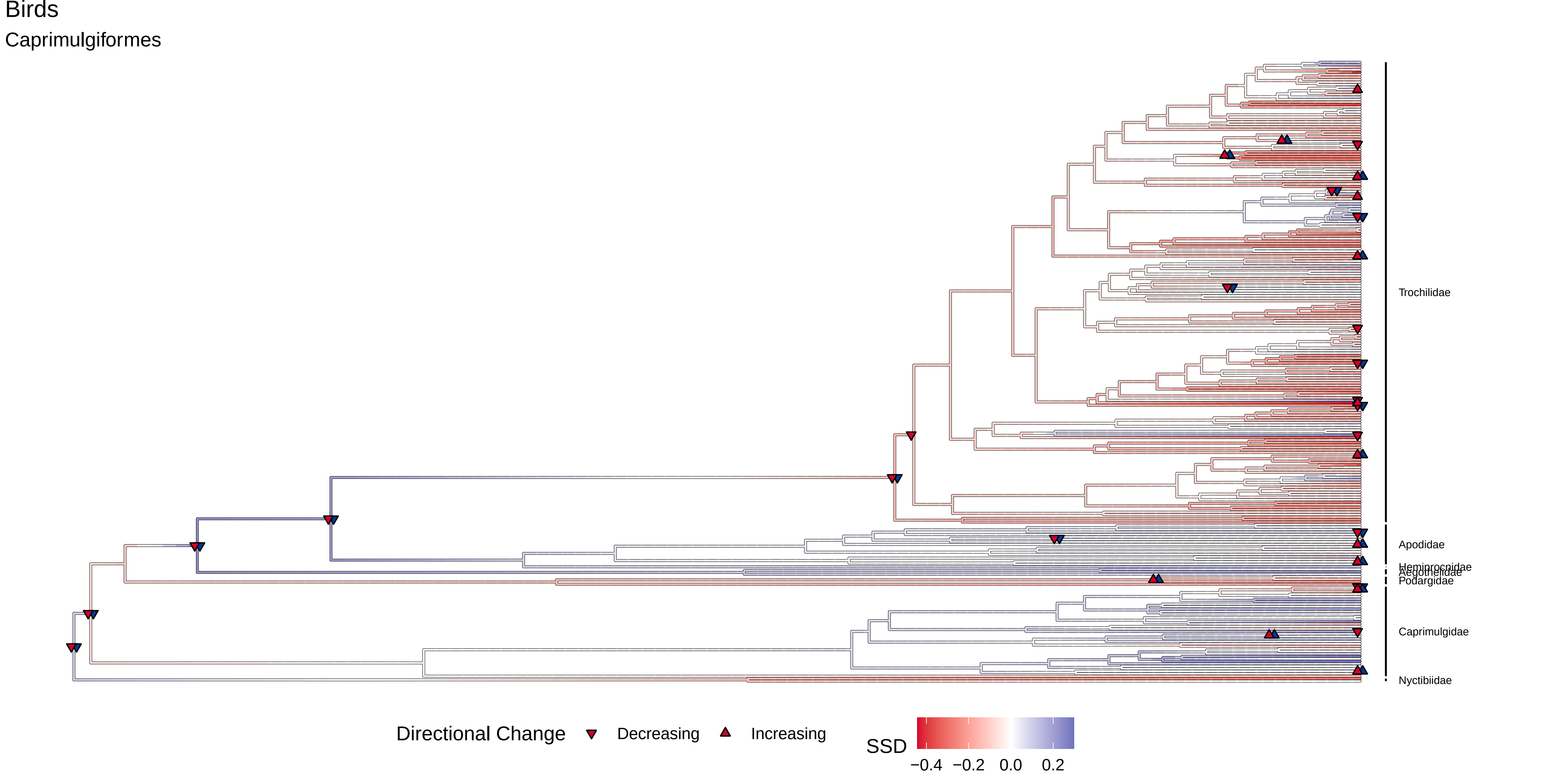

Birds
Caprimulgiformes
Trochilidae
Apodidae
Hemiprocnidae
Aegothelidae
Podargidae
Caprimulgidae
Nyctibiidae
Directional Change
Decreasing
Increasing
SSD
−0.4
−0.2
0.0
0.2

Birds

Cathartiformes

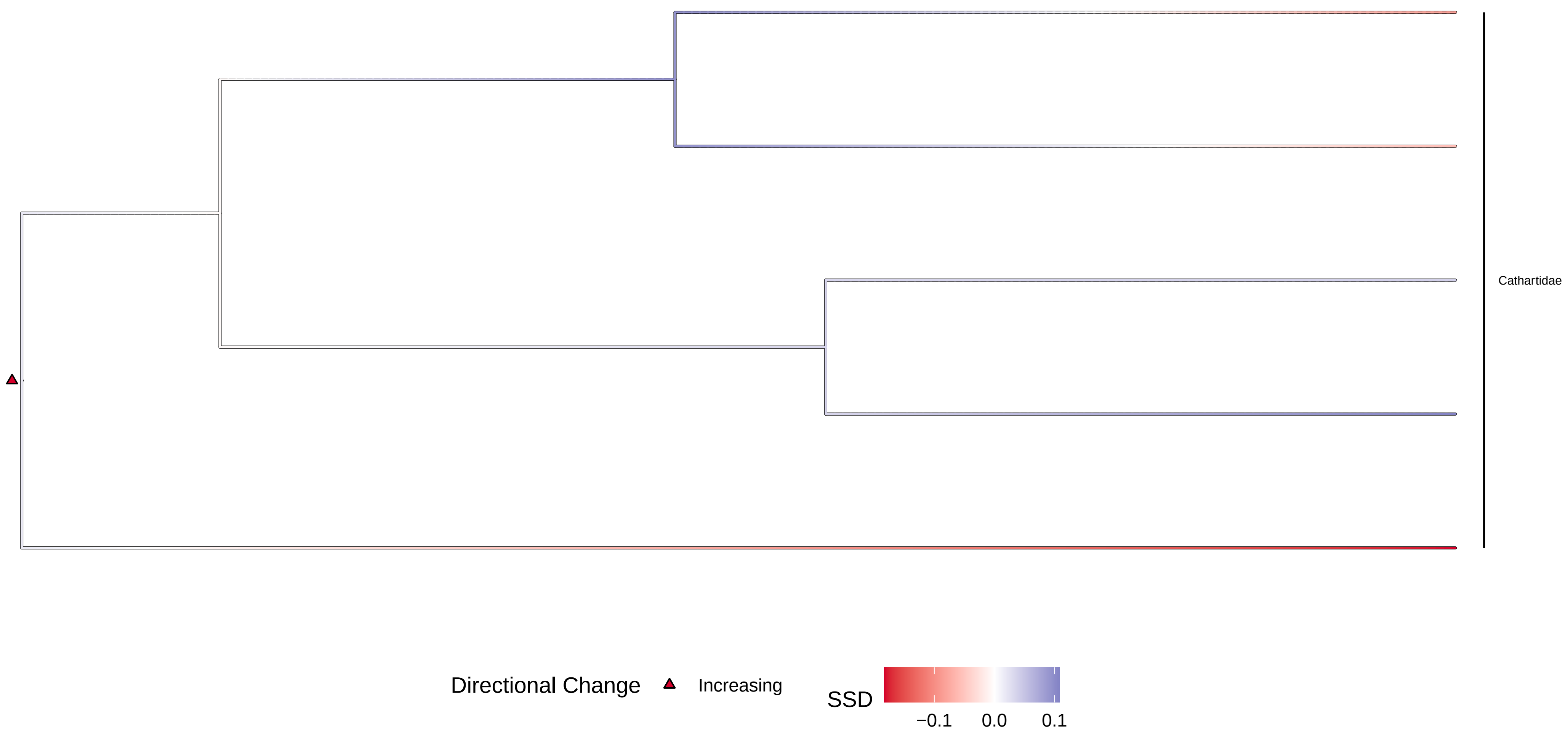

Birds

Charadriiformes

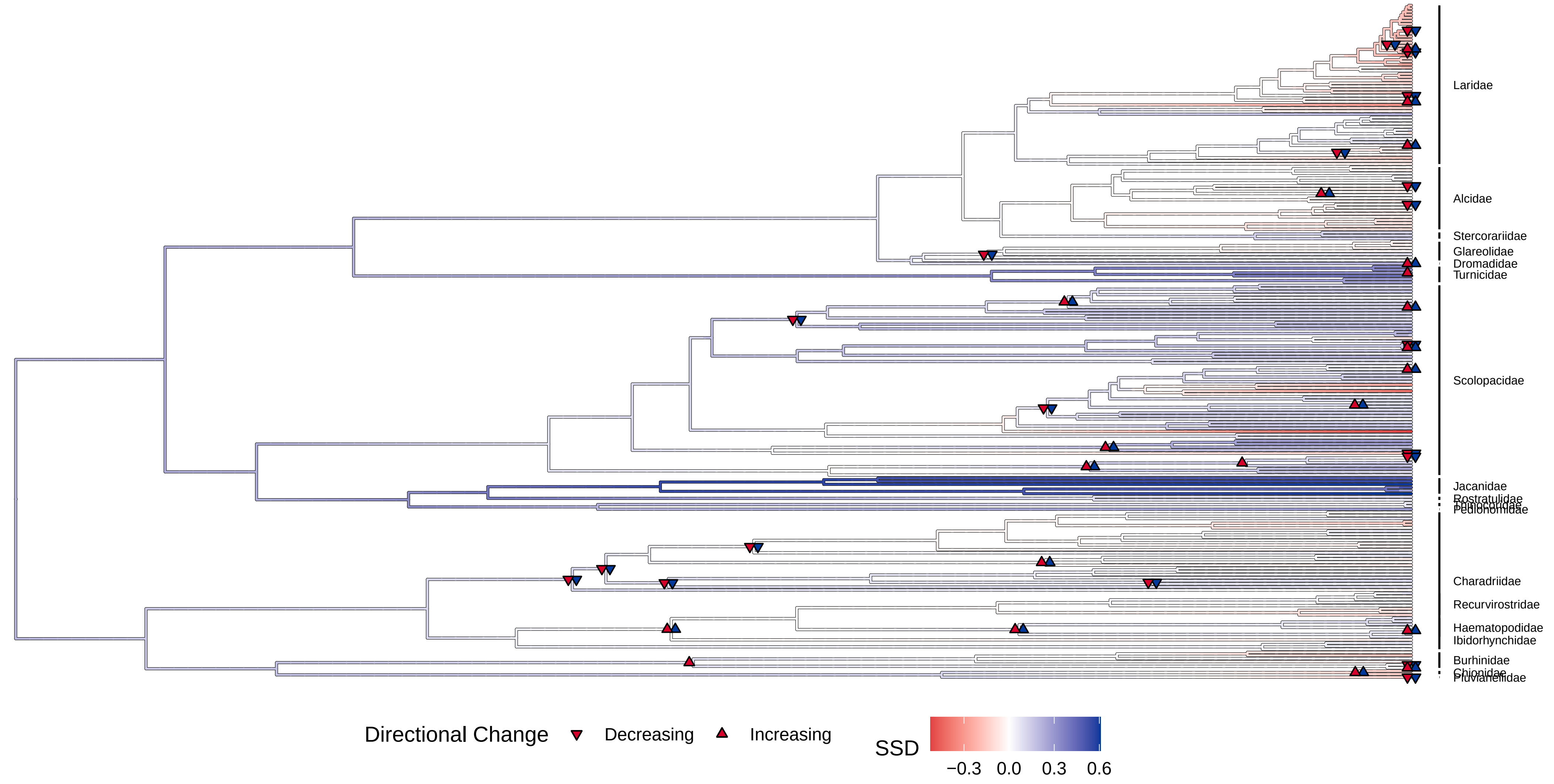

Birds

Ciconiiformes

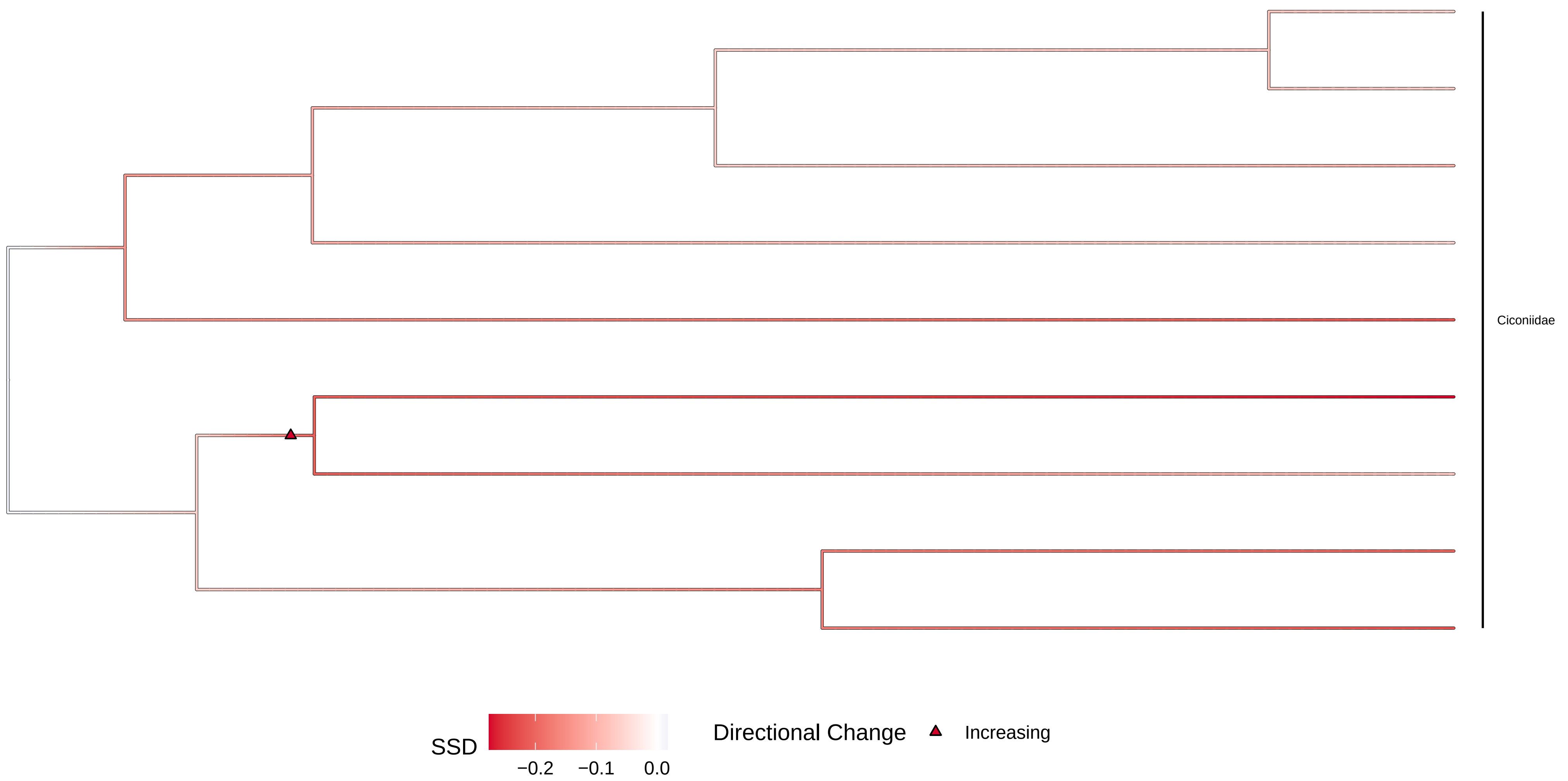

Birds

Columbiformes

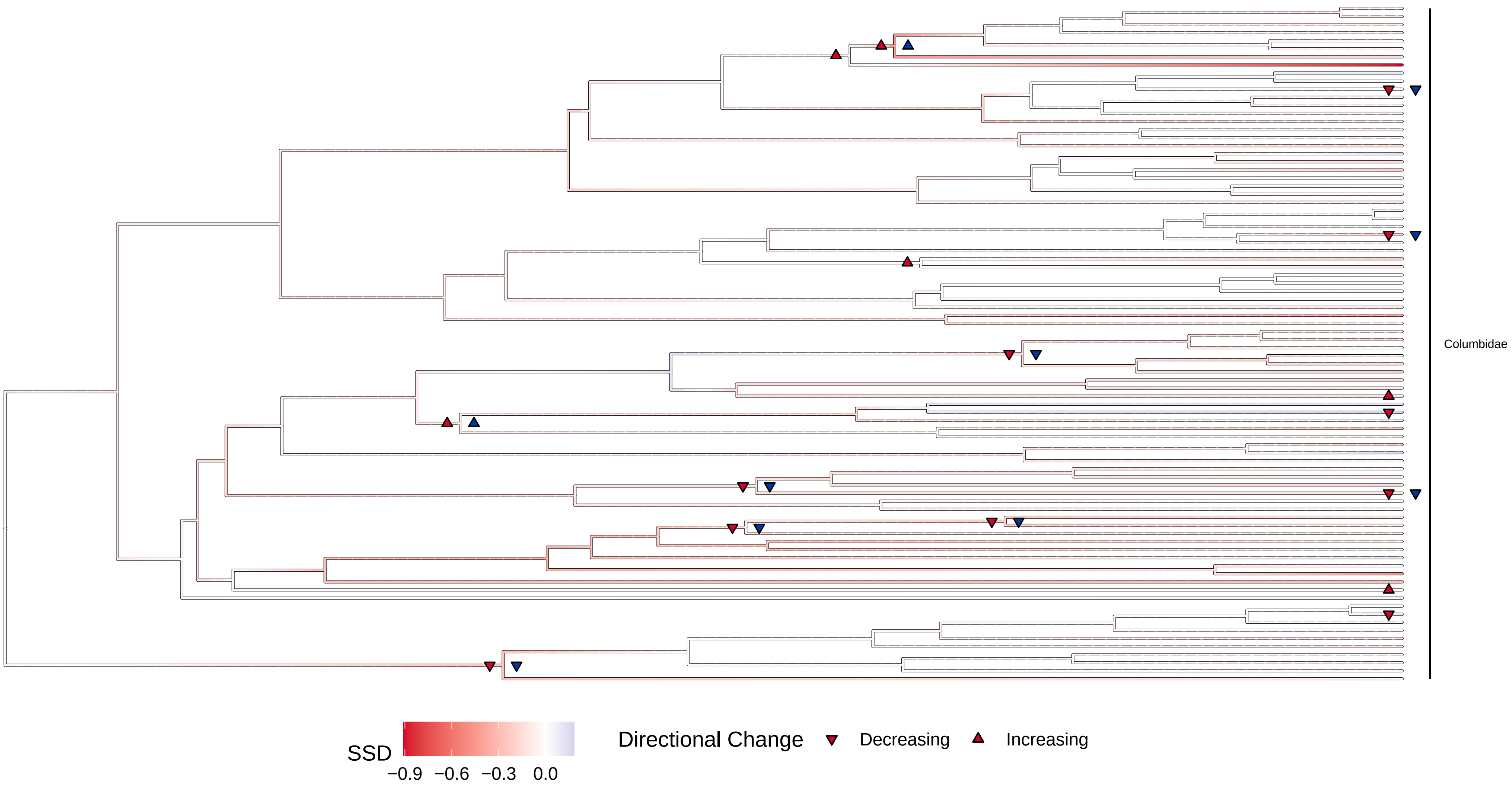

Birds

Coraciiformes

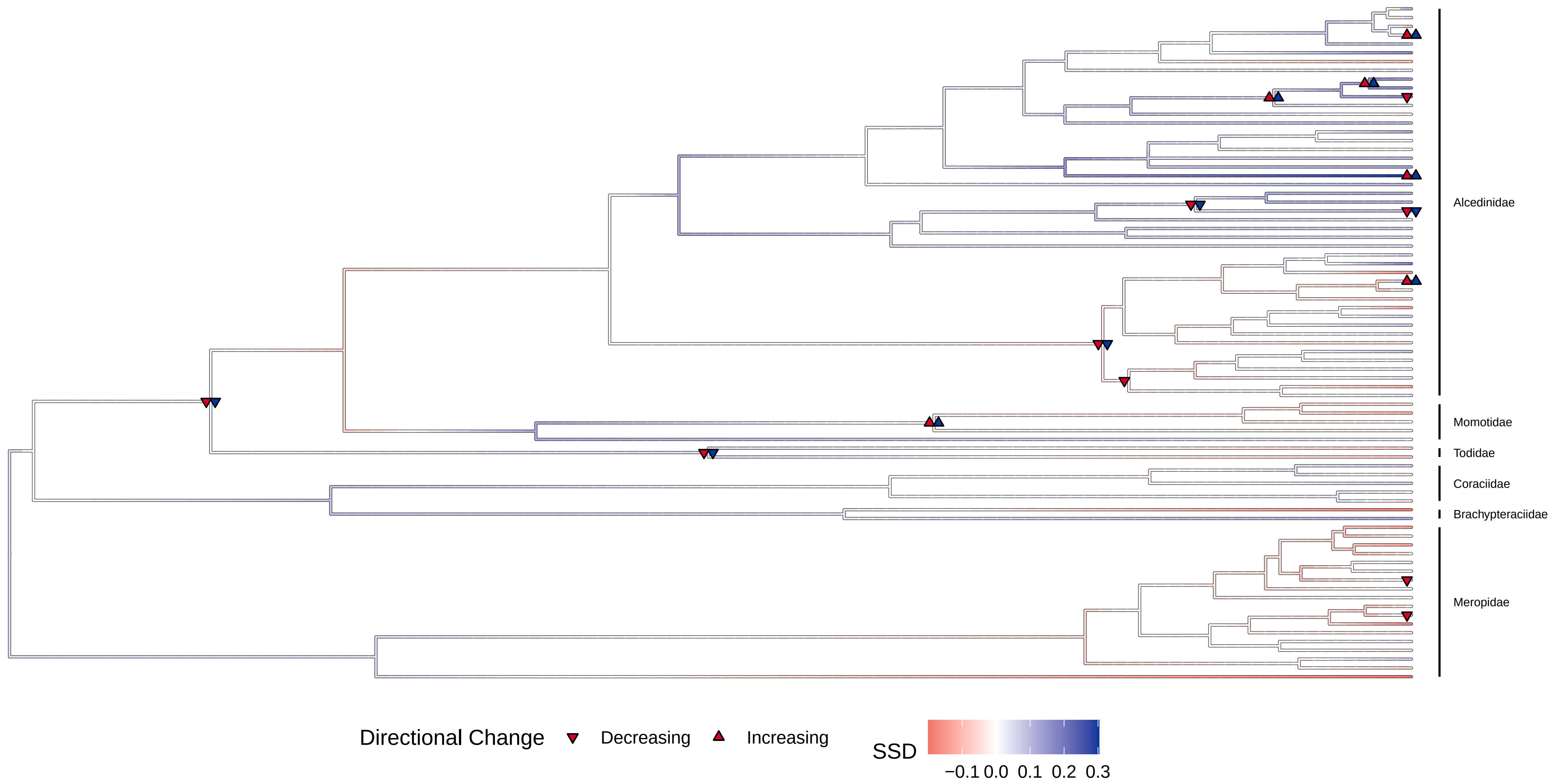

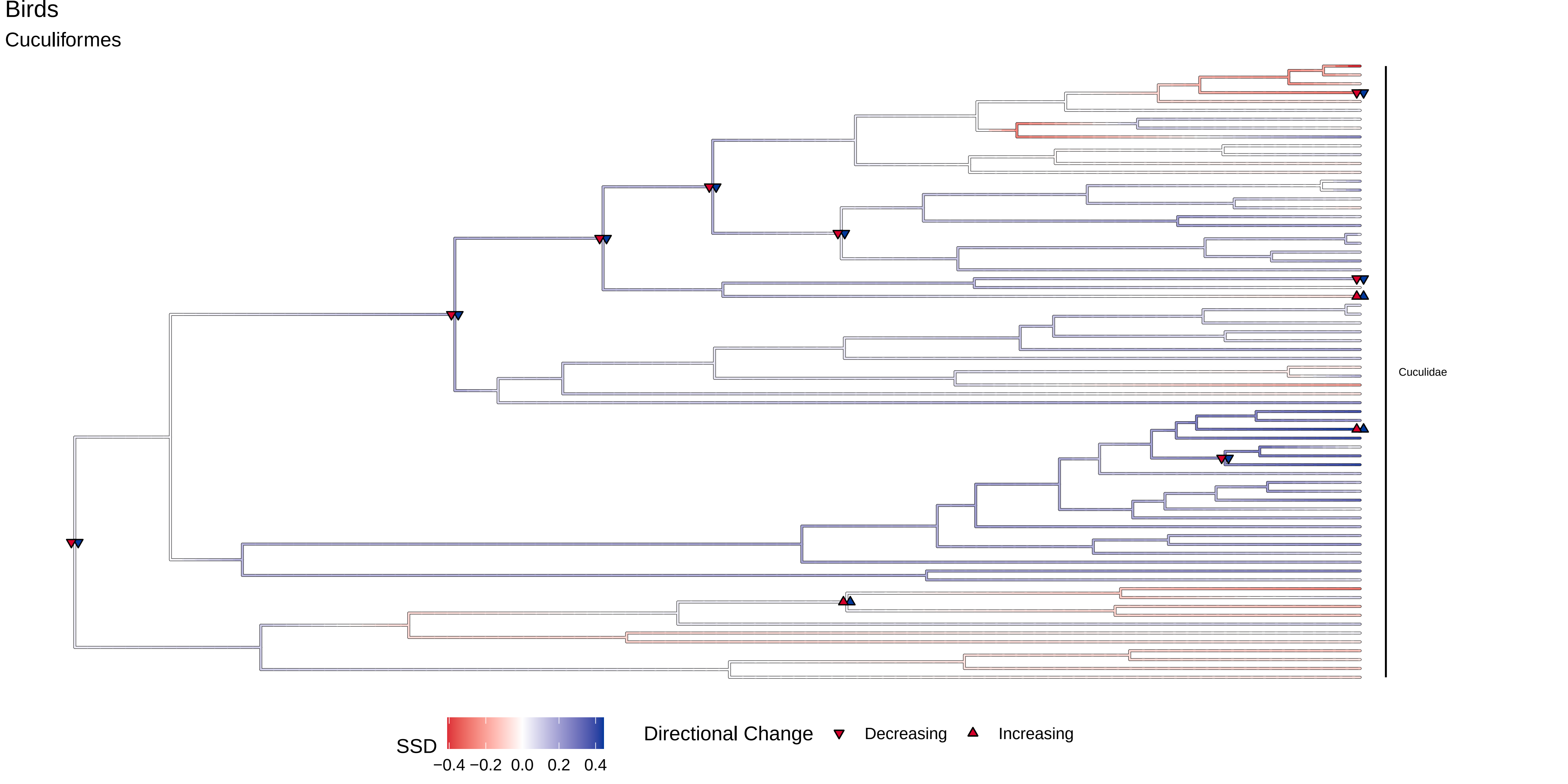

Birds
Cuculiformes
Cuculidae
SSD
−0.4 −0.2 0.0 0.2 0.4
Directional Change
Decreasing
Increasing

Birds

Falconiformes

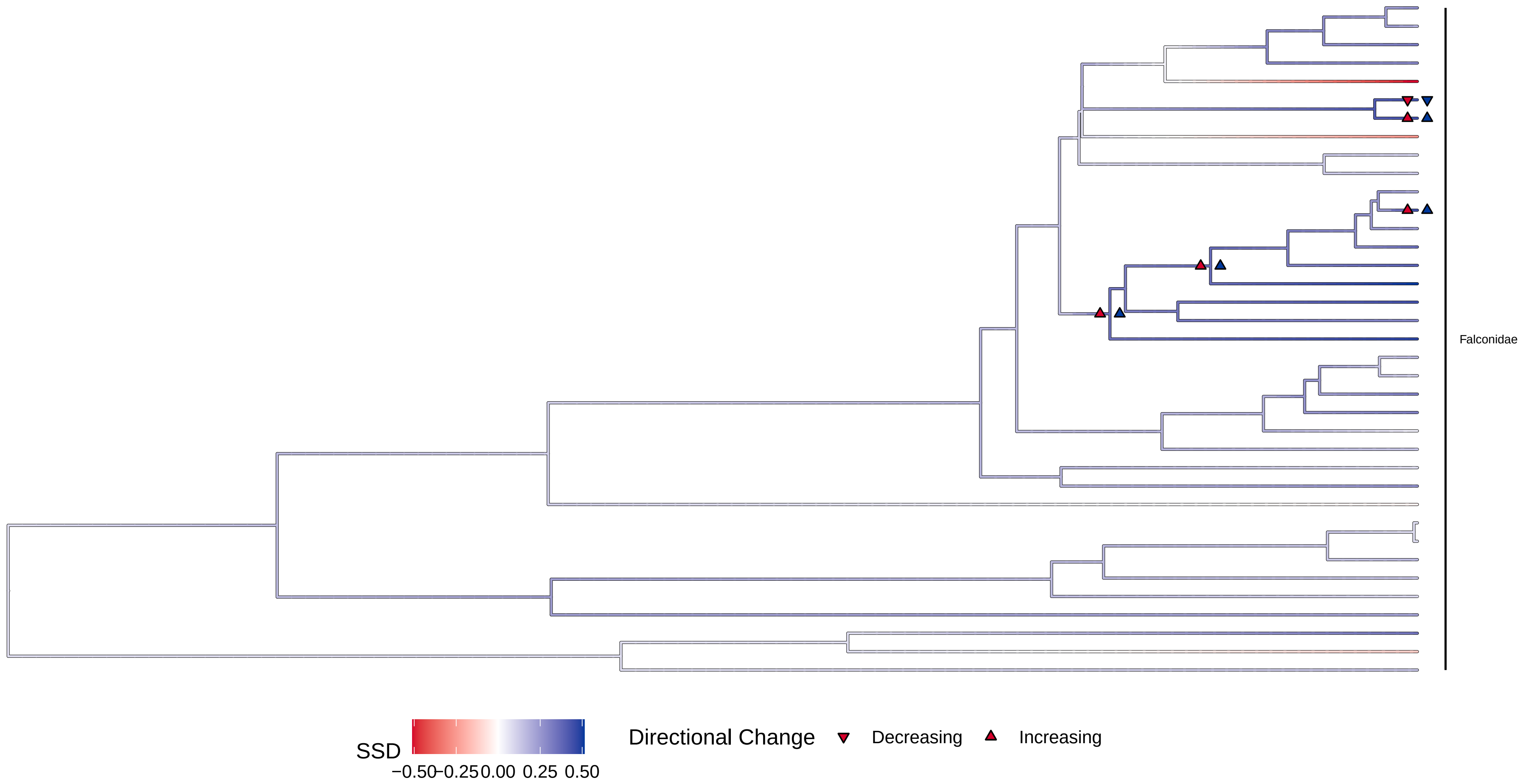

Birds

Musophagiformes

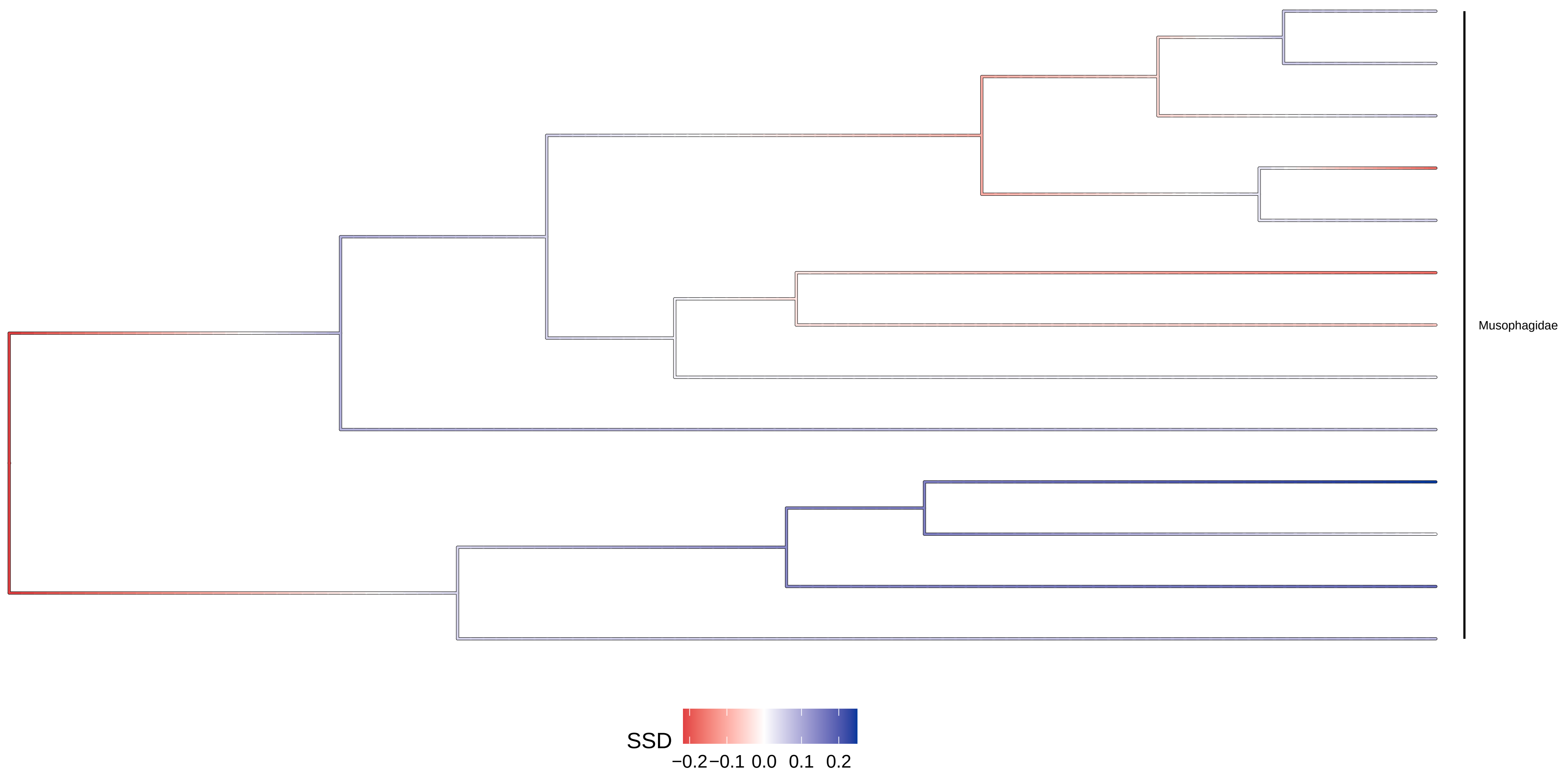

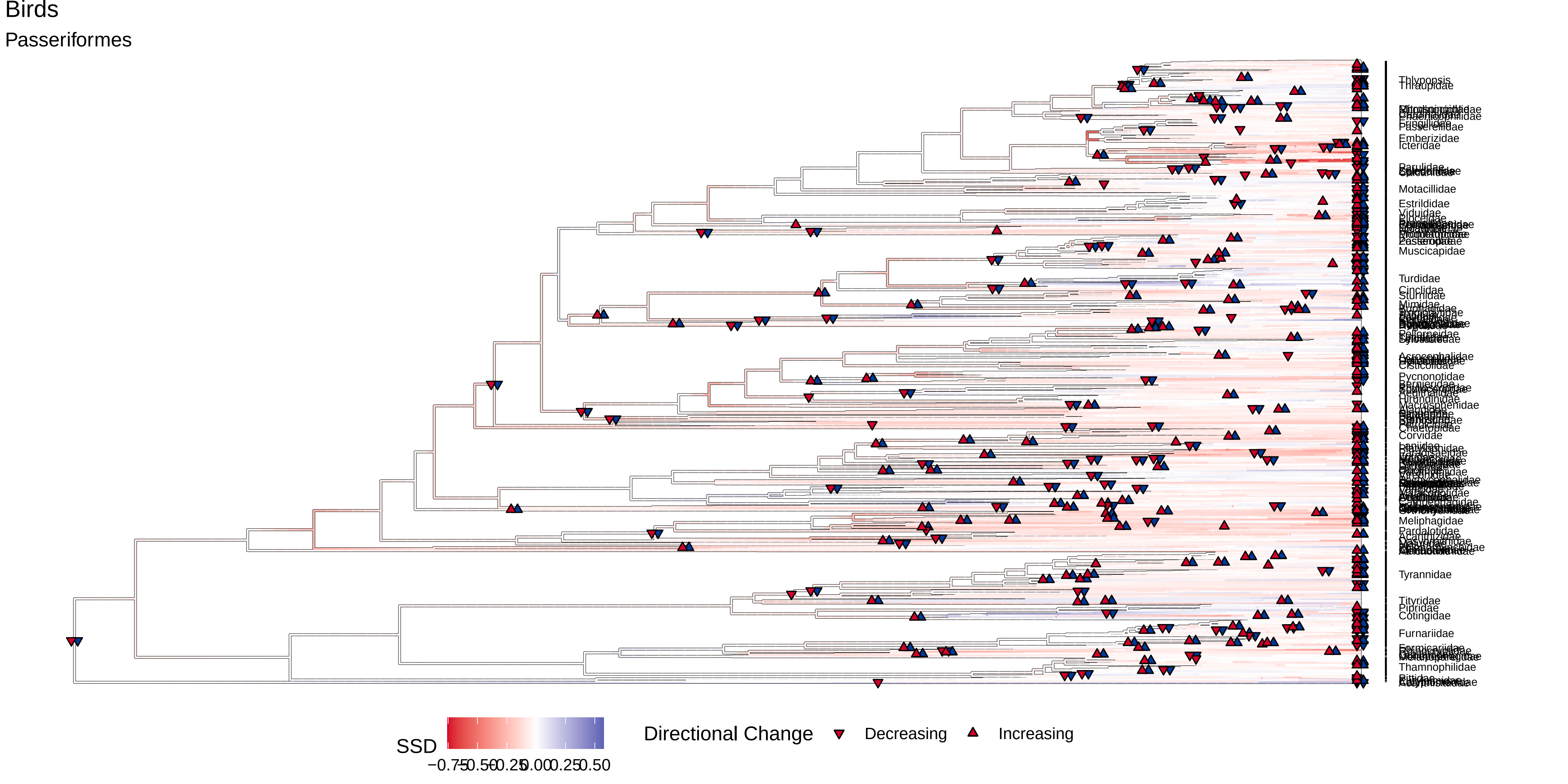
Birds
Passeriformes
Thlypopsis
Thraupidae
Passerellidae
Emberizidae
Icteridae
Parulidae
Motacillidae
Estrildidae
Viduidae
Muscicapidae
Turdidae
Cinclidae
Sturnidae
Mimidae
Acrocephalidae
Cisticolidae
Pycnonotidae
Hirundinidae
Macrosphenidae
Chaetopidae
Corvidae
Laniidae
Meliphagidae
Pardalotidae
Tyrannidae
Tityridae
Pipridae
Cotingidae
Furnariidae
Formicariidae
Thamnophilidae
Pittidae
SSD
−0.75 −0.50 −0.25 0.00 0.25 0.50
Directional Change
Decreasing
Increasing

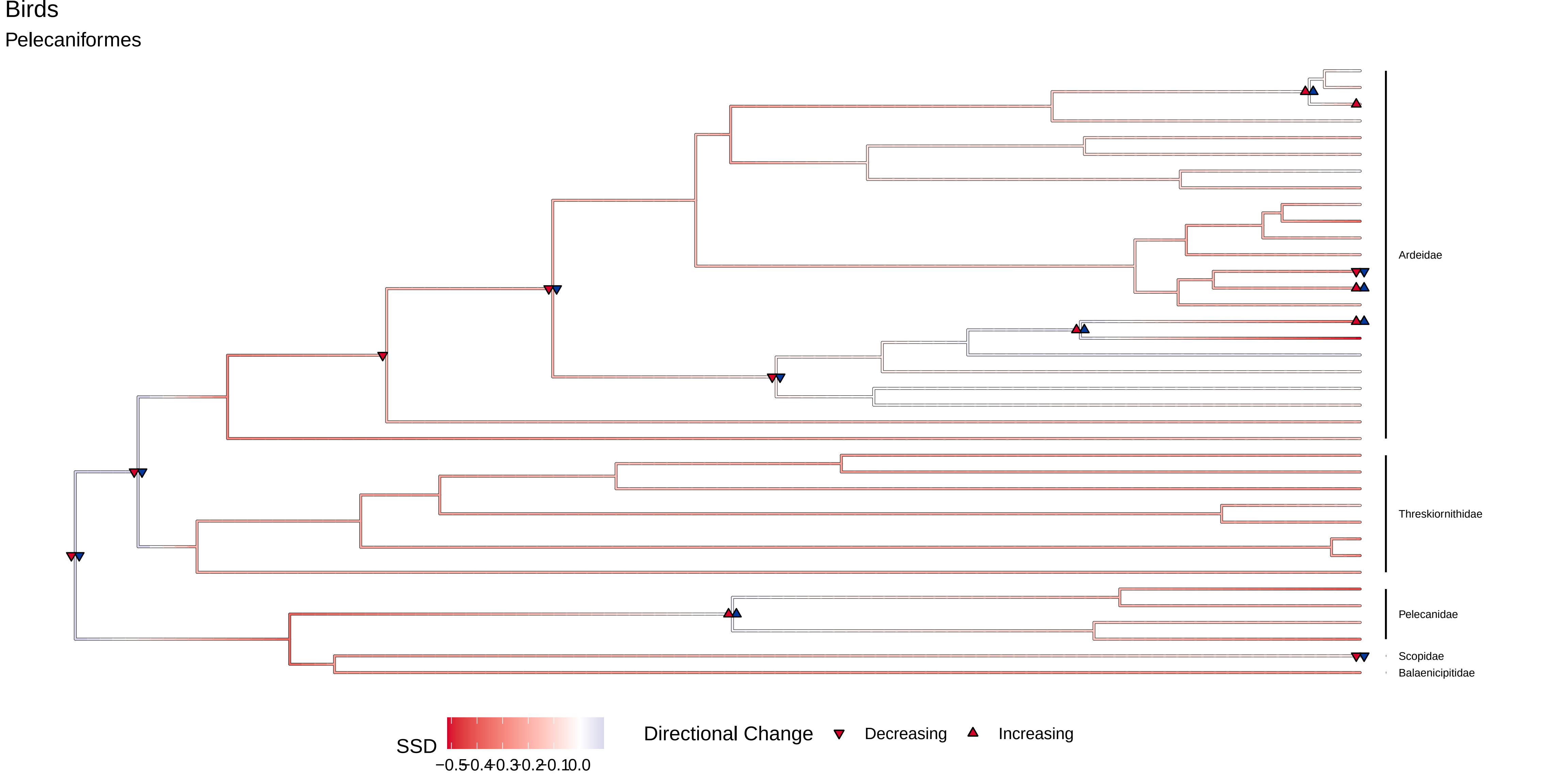
Birds
Pelecaniformes
Ardeidae
Threskiornithidae
Pelecanidae
Scopidae
Balaenicipitidae
SSD
−0.5
−0.4
−0.3
−0.2
−0.1
0.0
Directional Change
Decreasing
Increasing

Birds

Phaethontiformes

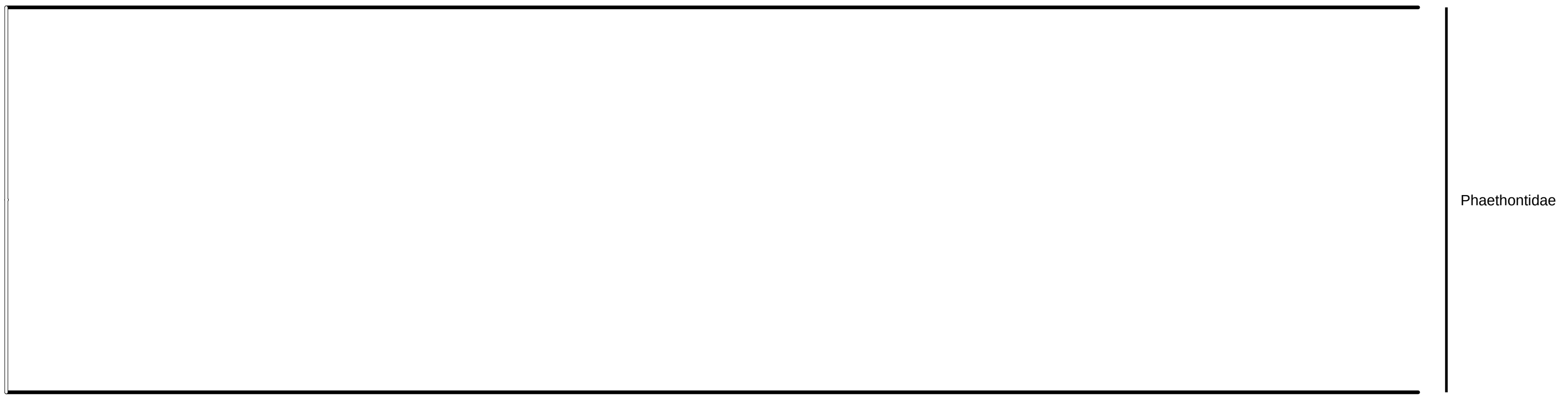

SSD

0.09991331

Birds

Phoenicopteriformes

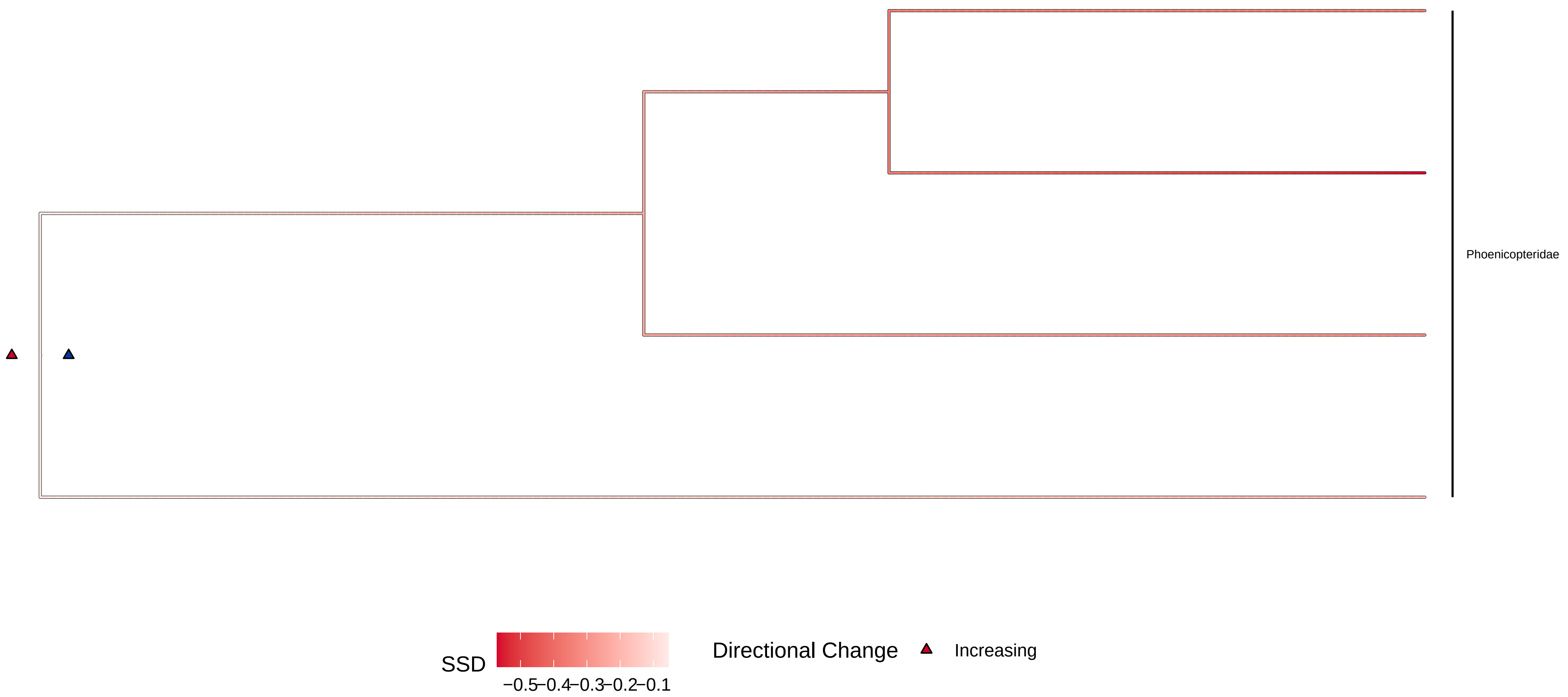

Birds

Podicipediformes

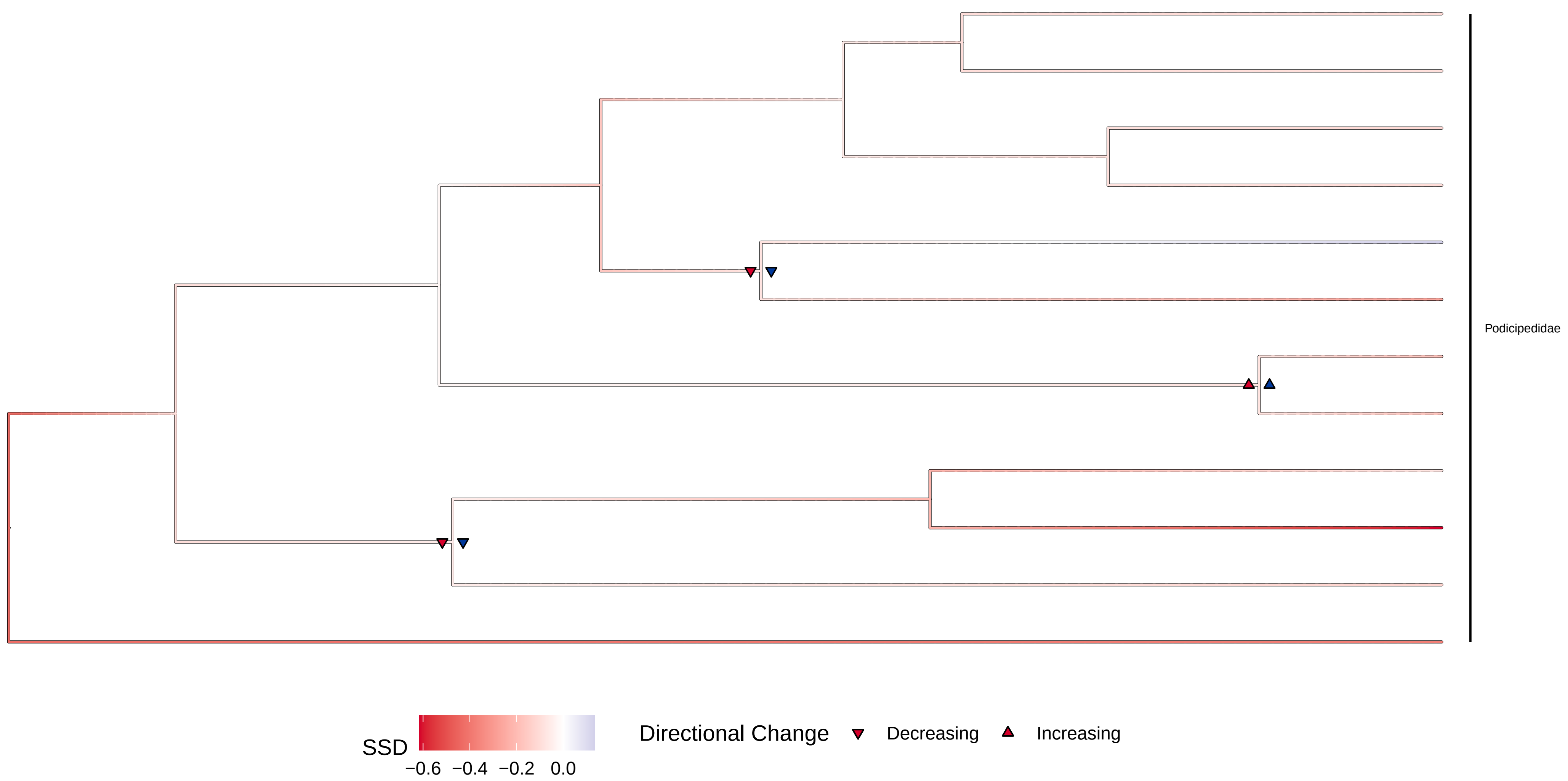

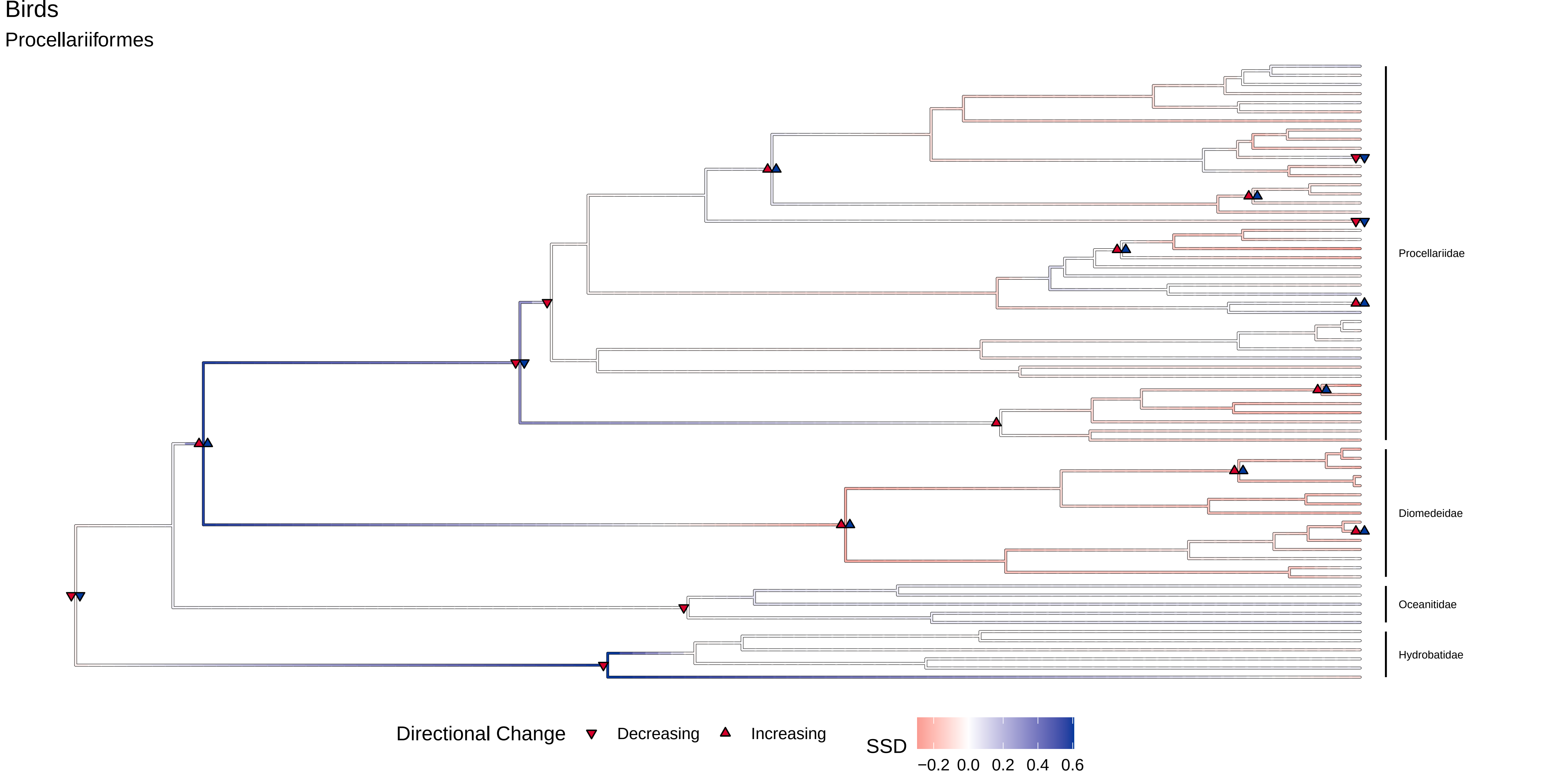

Birds
Procellariiformes
Procellariidae
Diomedeidae
Oceanitidae
Hydrobatidae
Directional Change
Decreasing
Increasing
SSD
−0.2 0.0 0.2 0.4 0.6

Birds

Psittaciformes

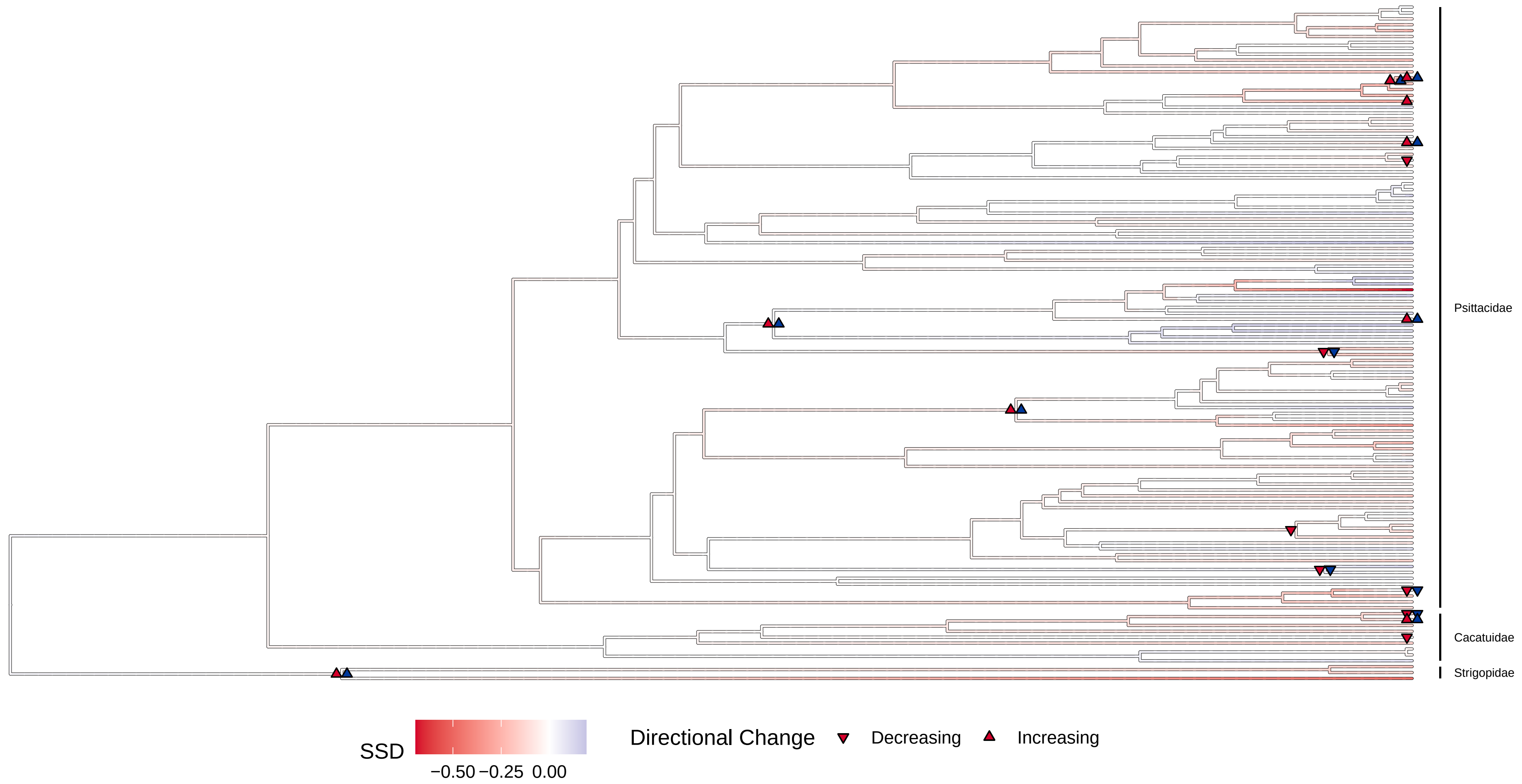

Birds

Pterocliformes

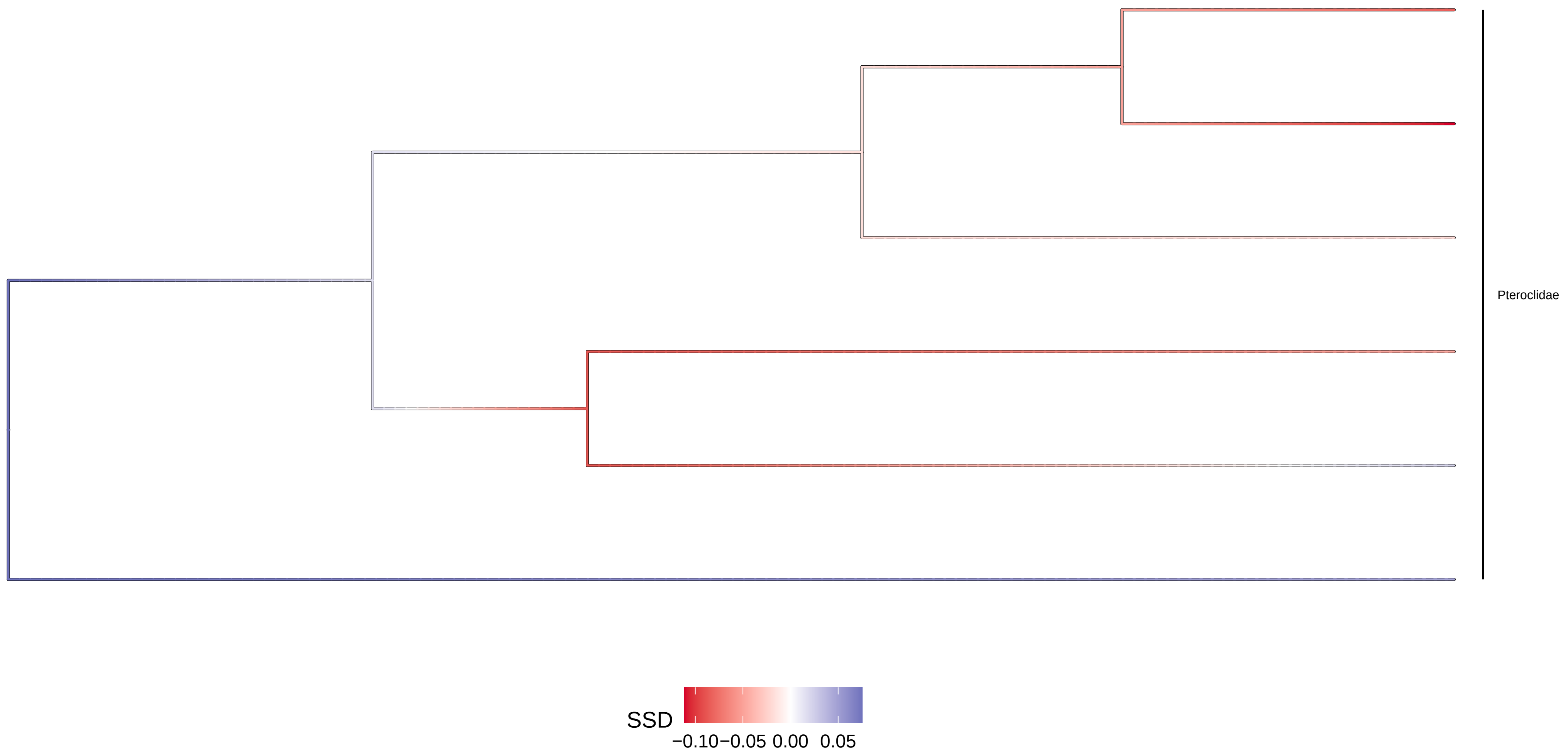

Birds

Sphenisciformes

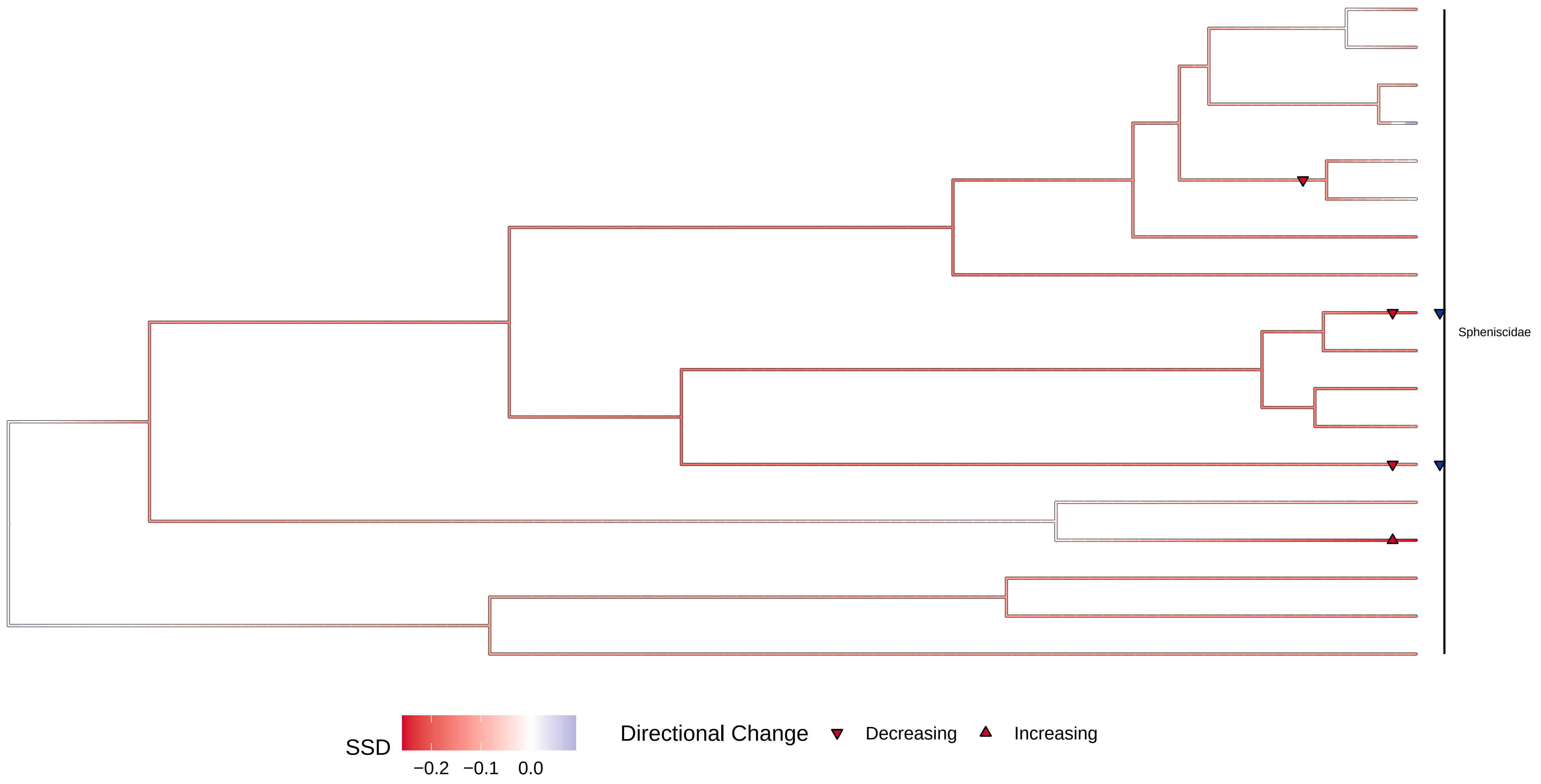

Birds

Struthioniformes

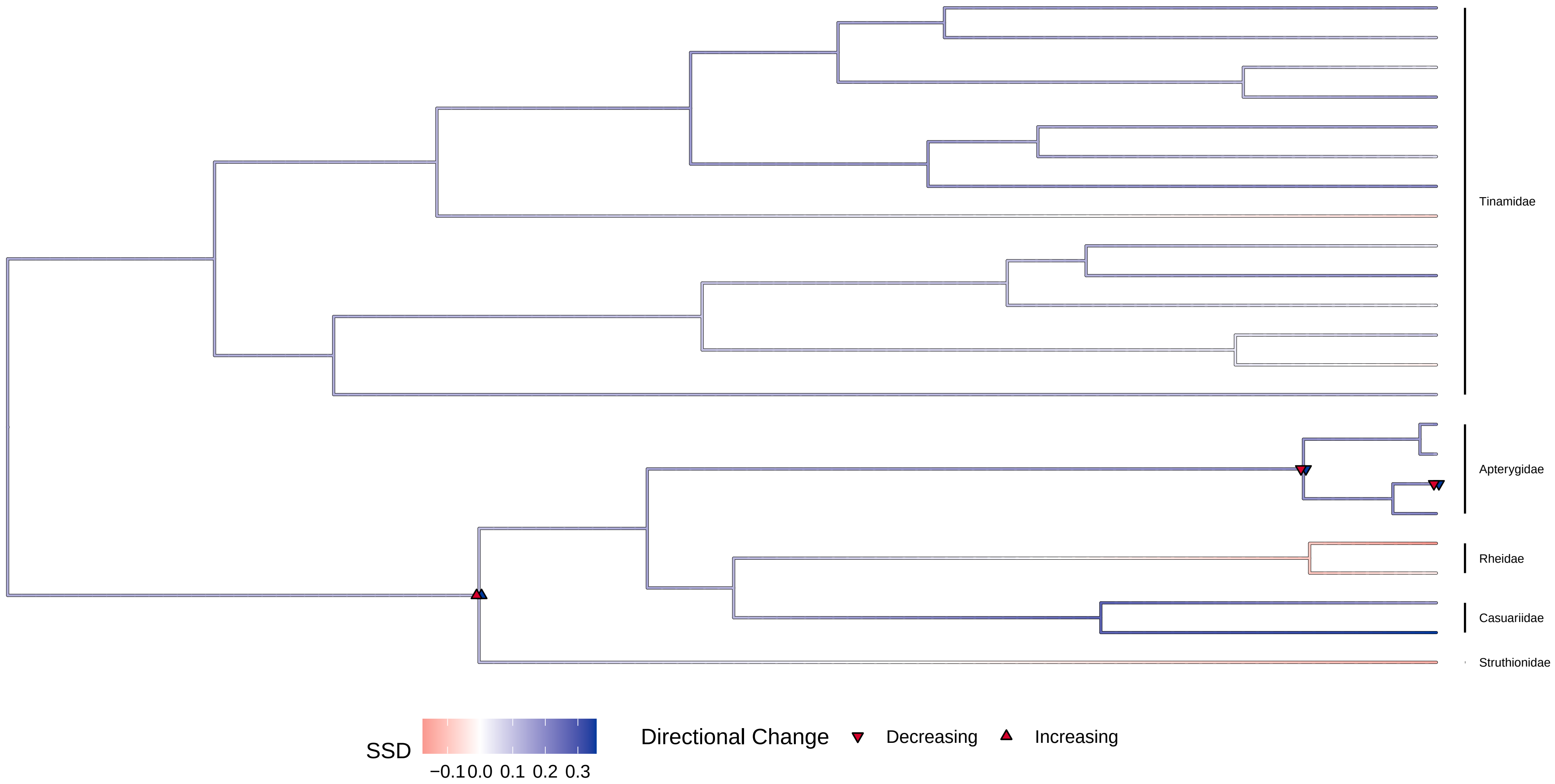

Birds

Trogoniformes

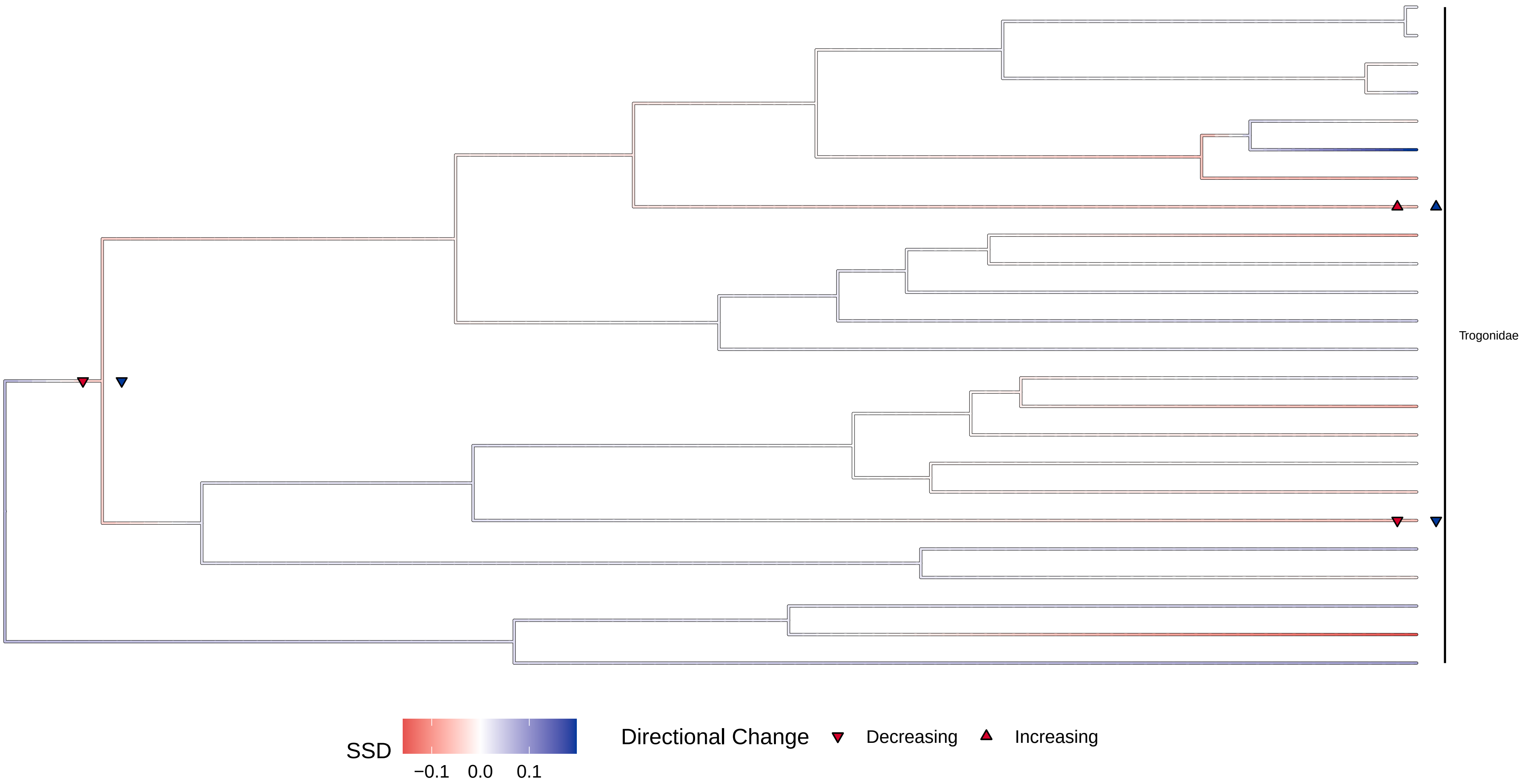

Birds

Anseriformes

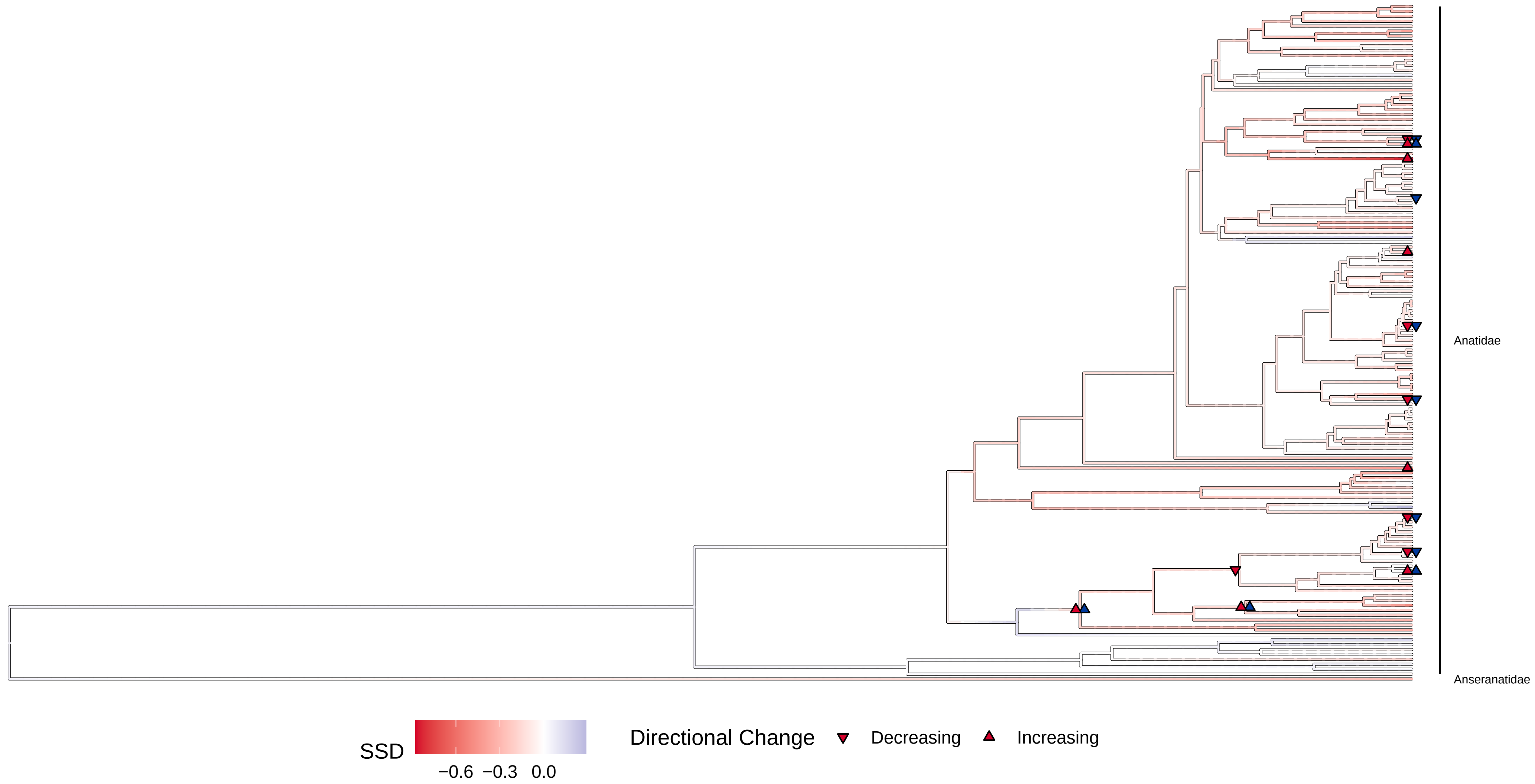

Birds

Coliiformes

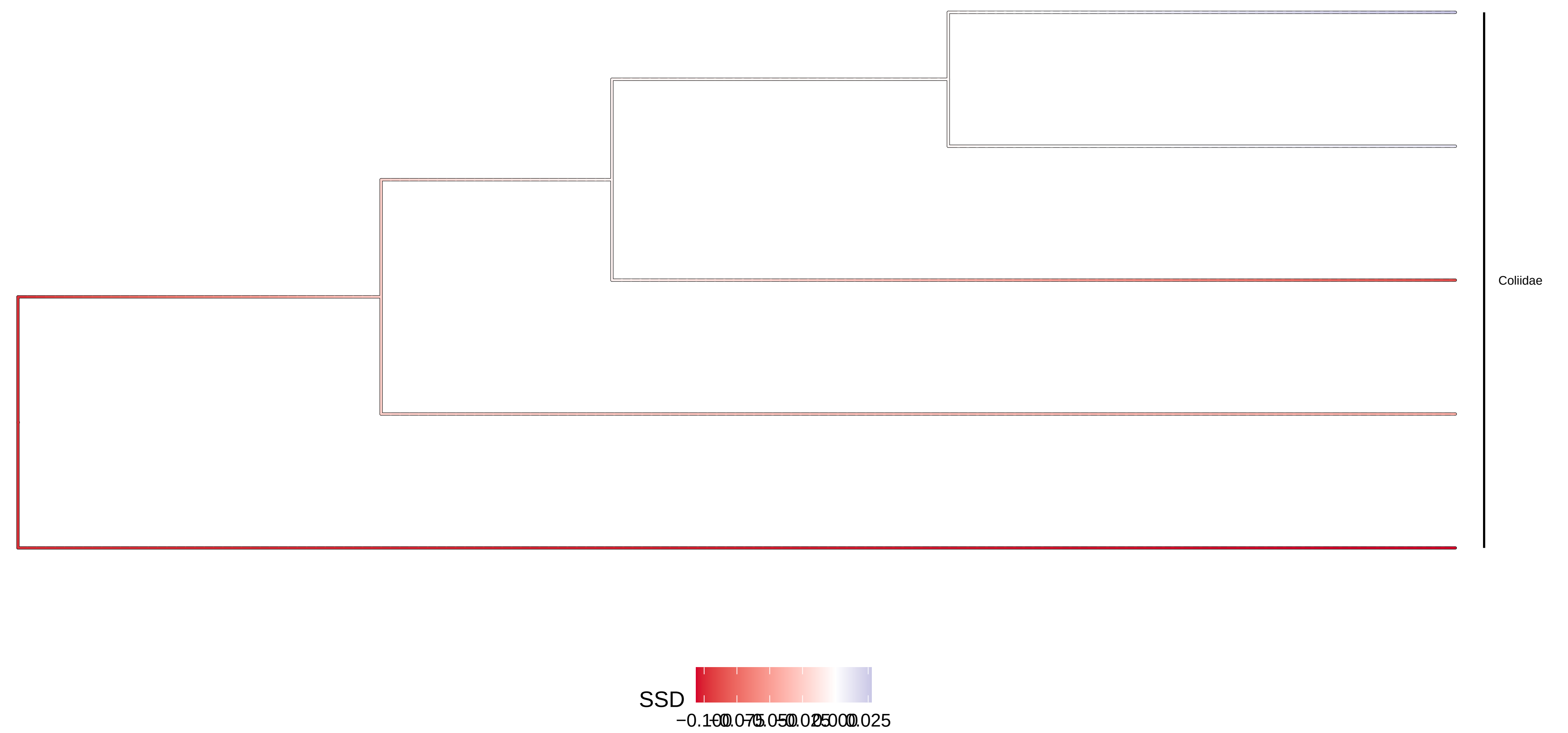

Birds

Galliformes

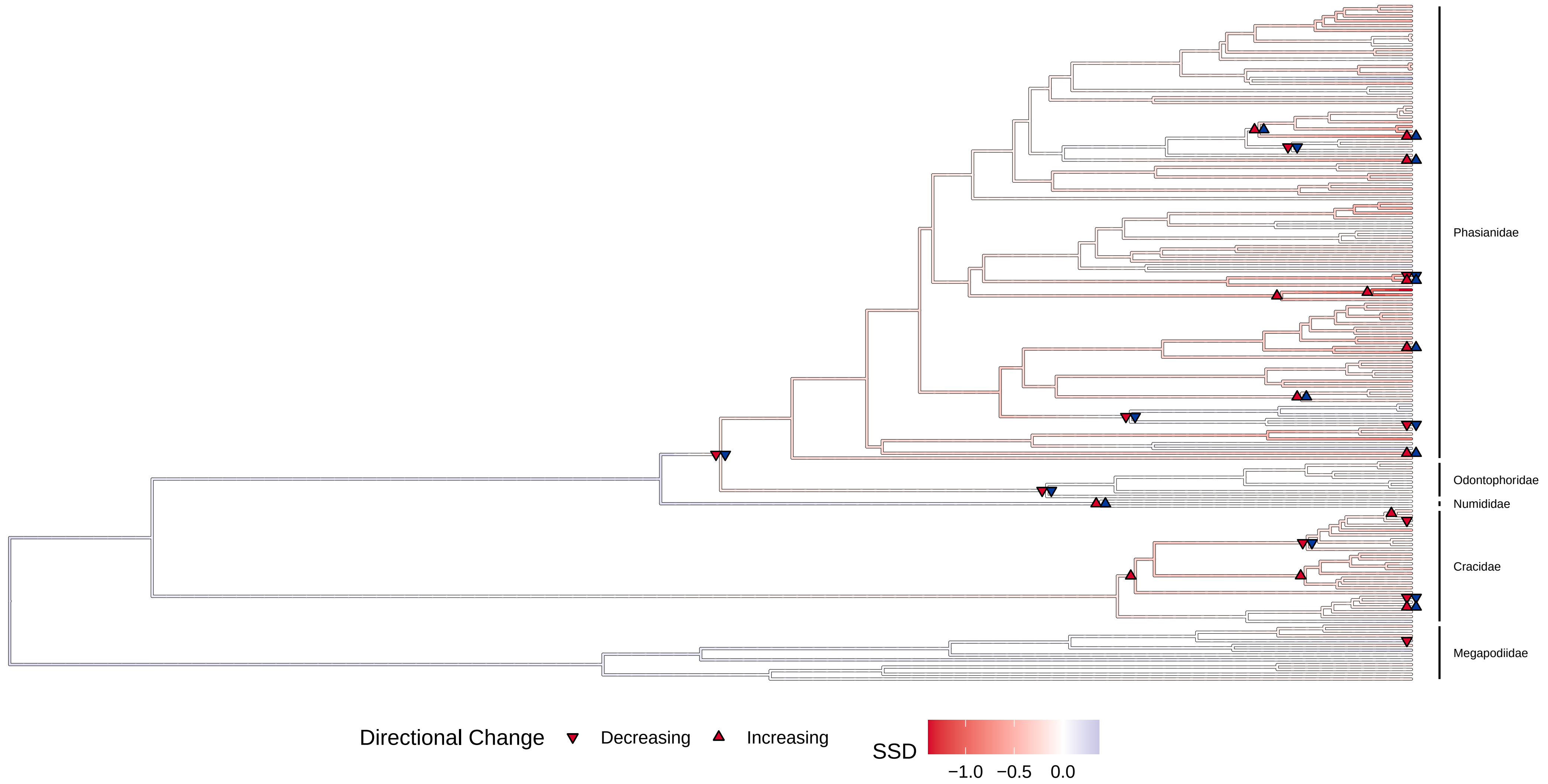

Birds

Gaviiformes

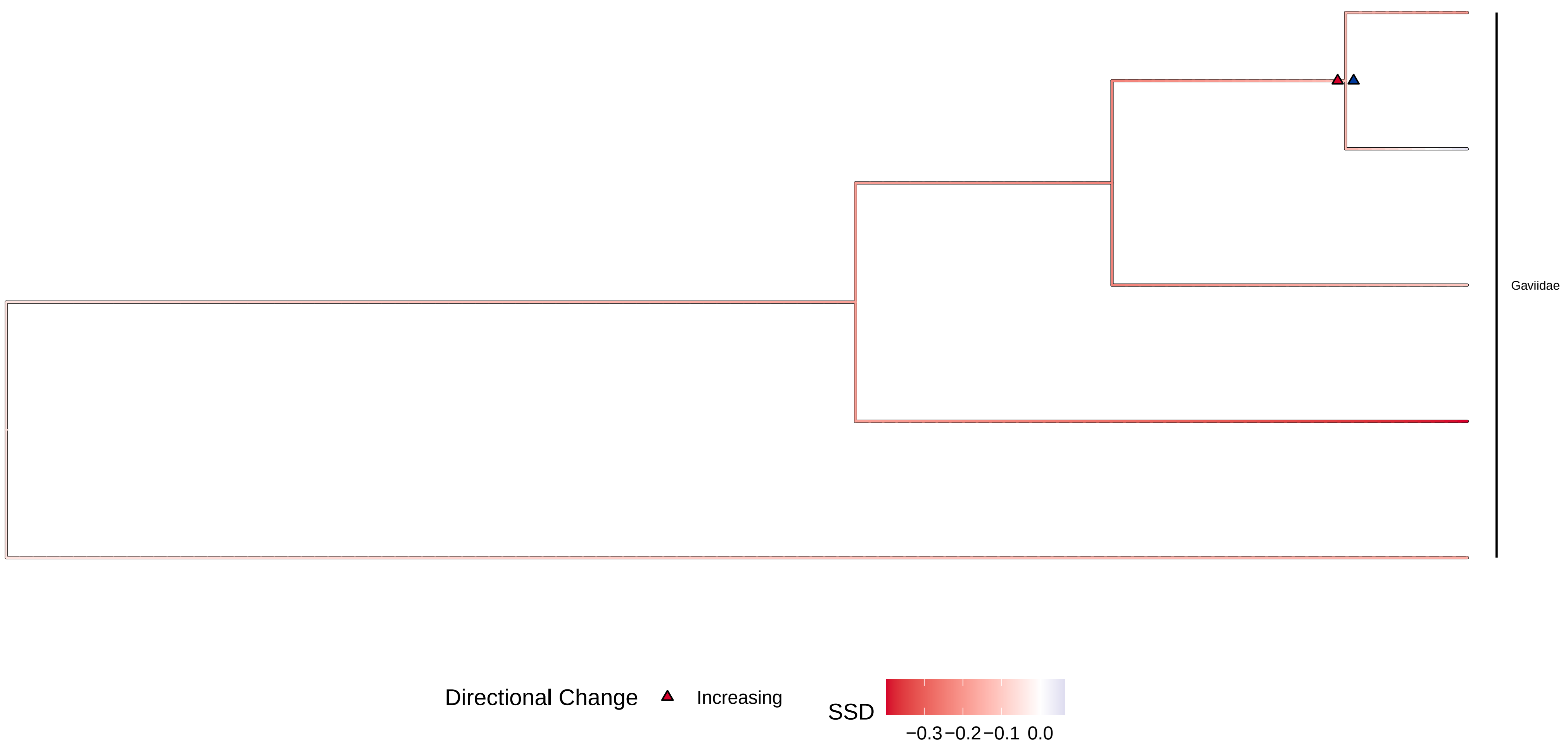

Birds

Gruiformes

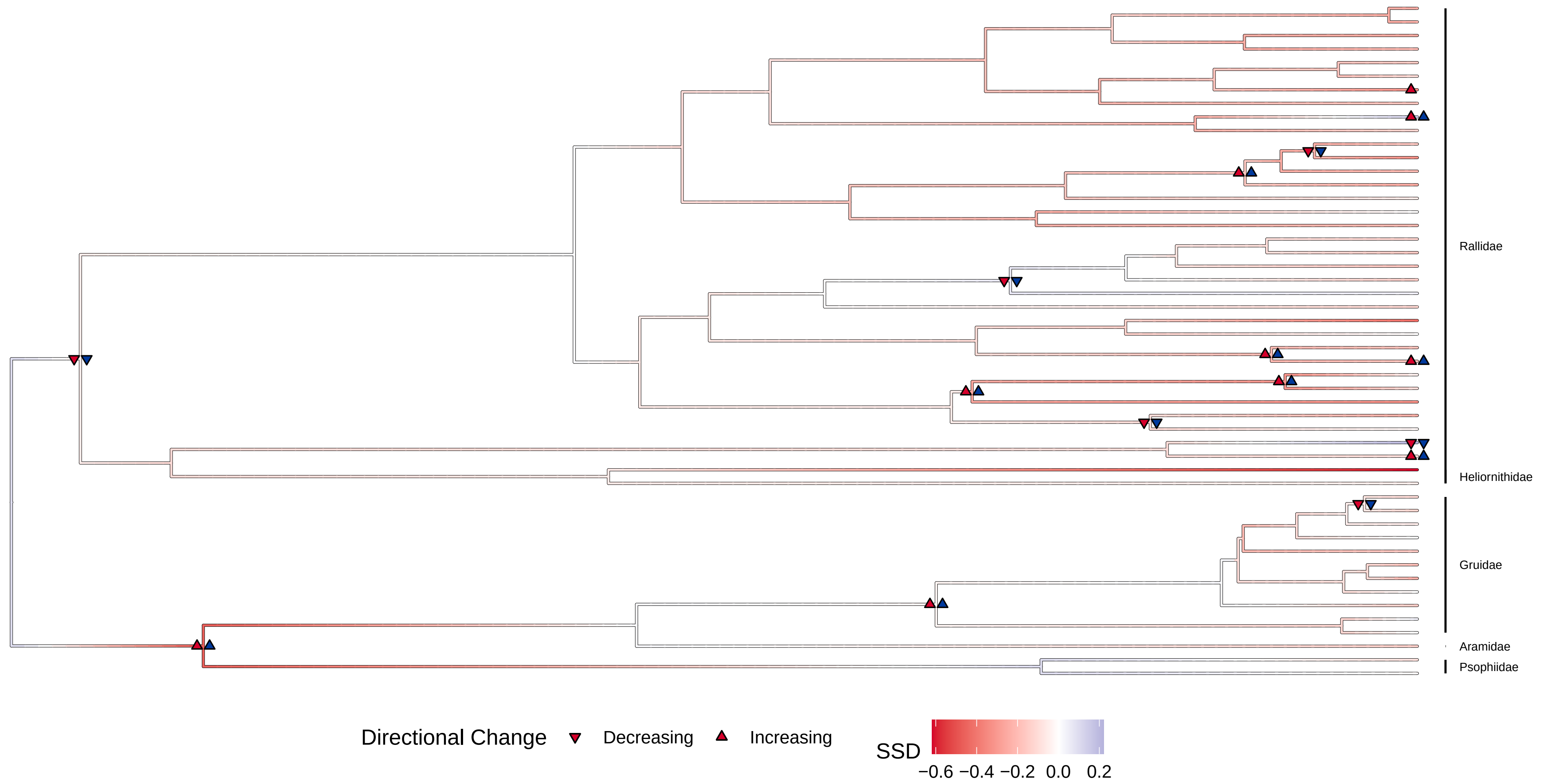

Birds

Otidiformes

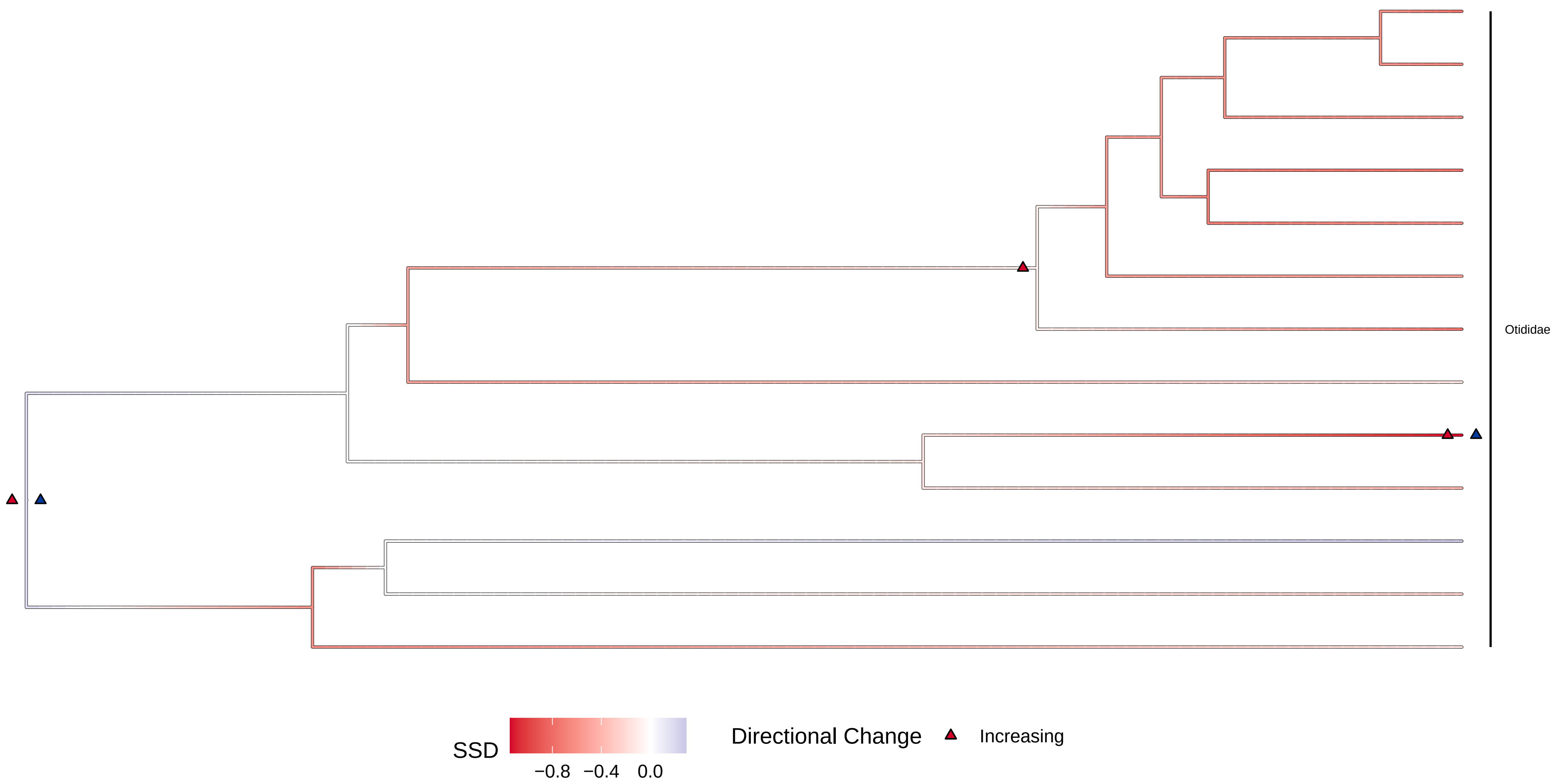

Birds

Piciformes

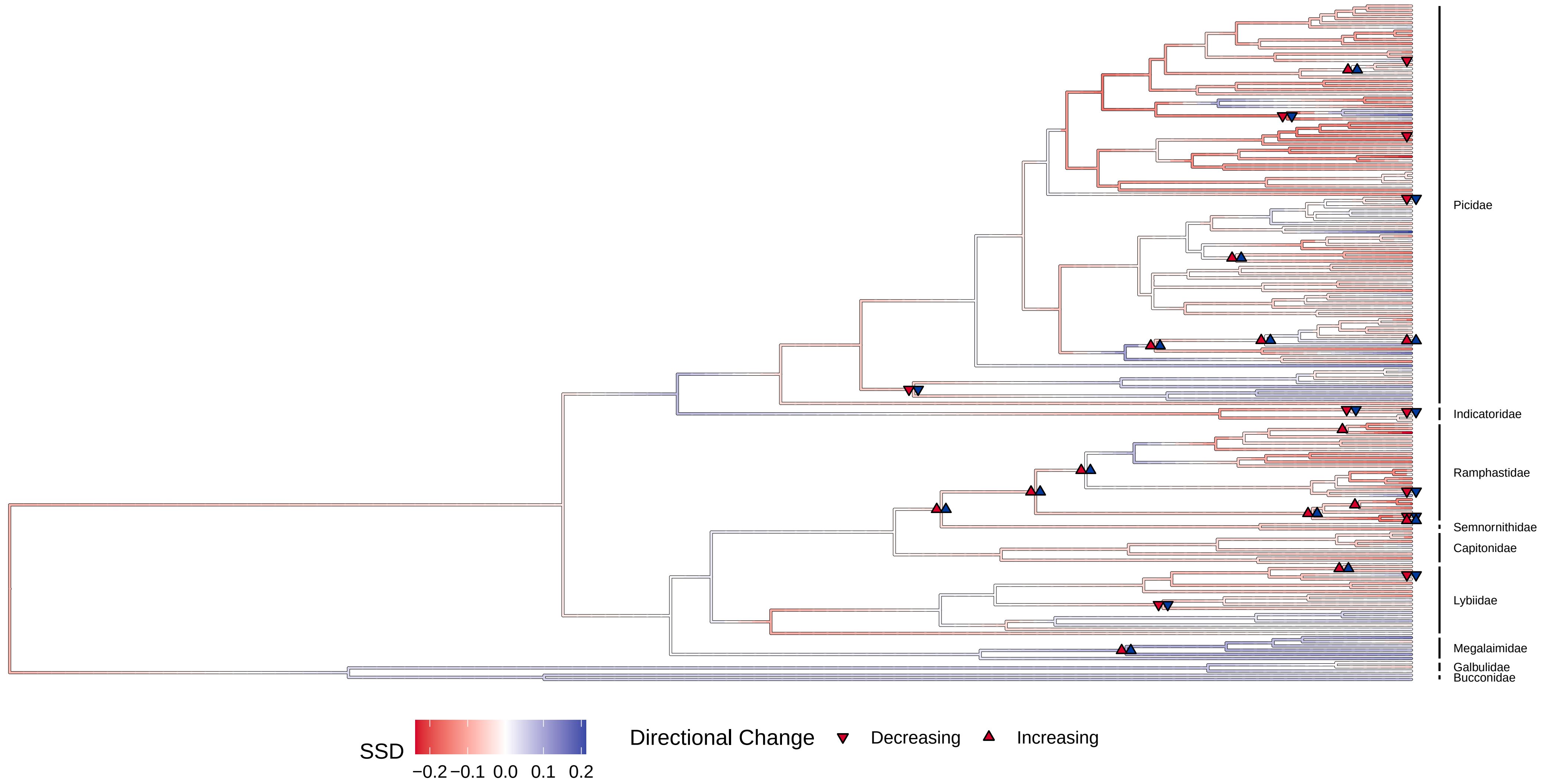

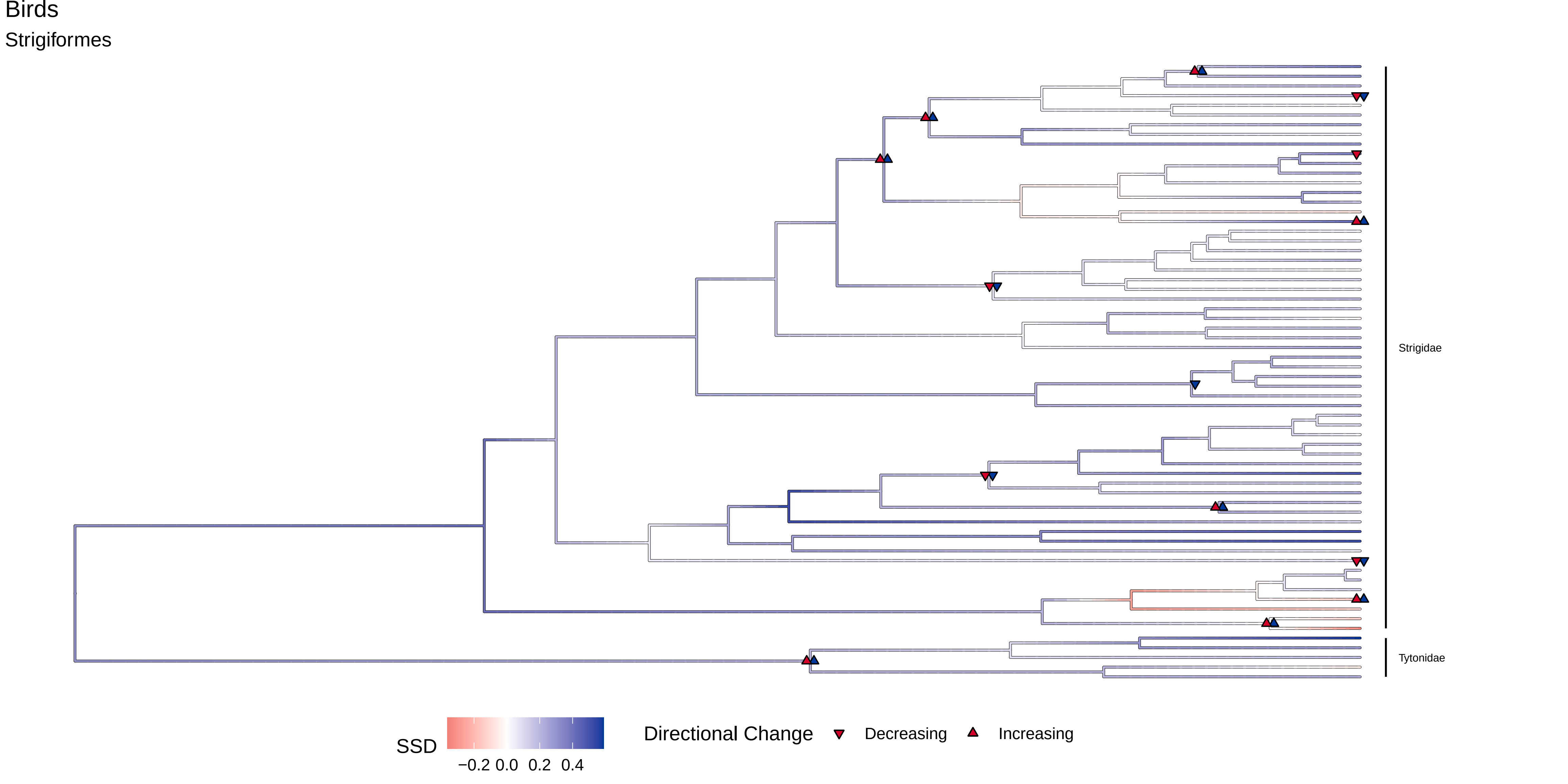

Birds
Strigiformes
Strigidae
Tytonidae
SSD
−0.2 0.0 0.2 0.4
Directional Change
Decreasing
Increasing

Birds

Suliformes

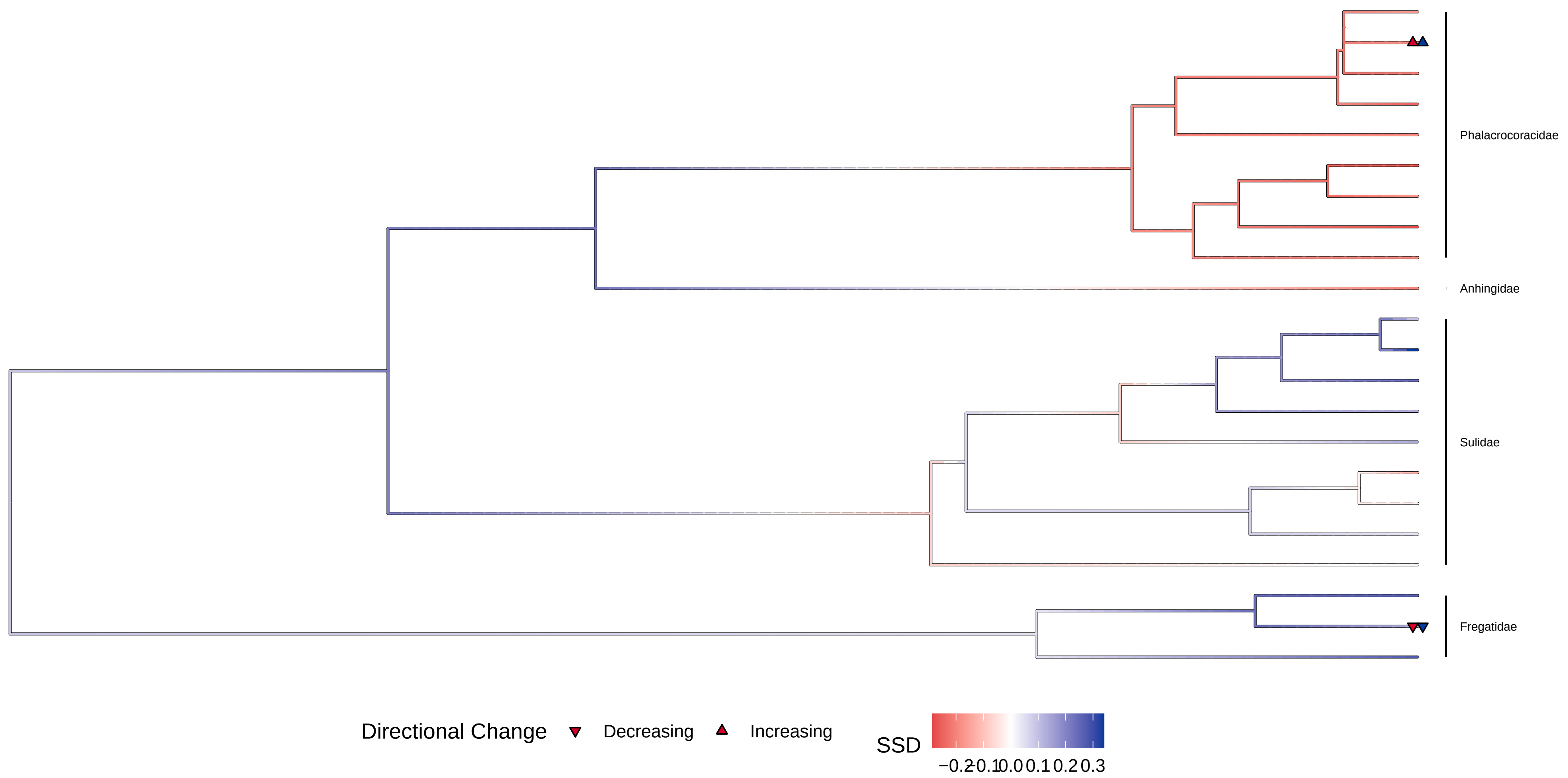

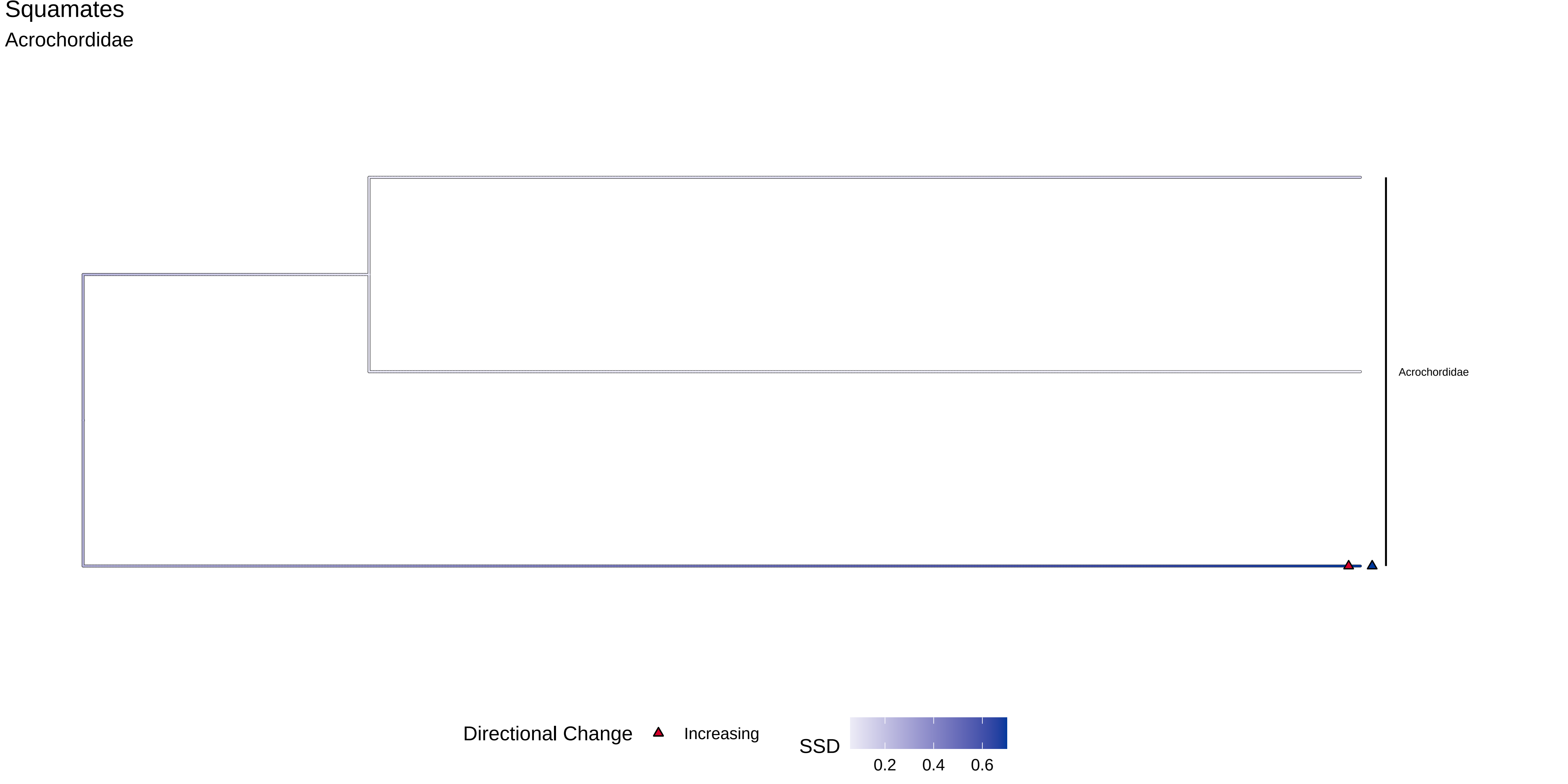
Squamates
Acrochordidae
Acrochordidae
Directional Change
Increasing
SSD
0.2
0.4
0.6

Squamates

Agamidae

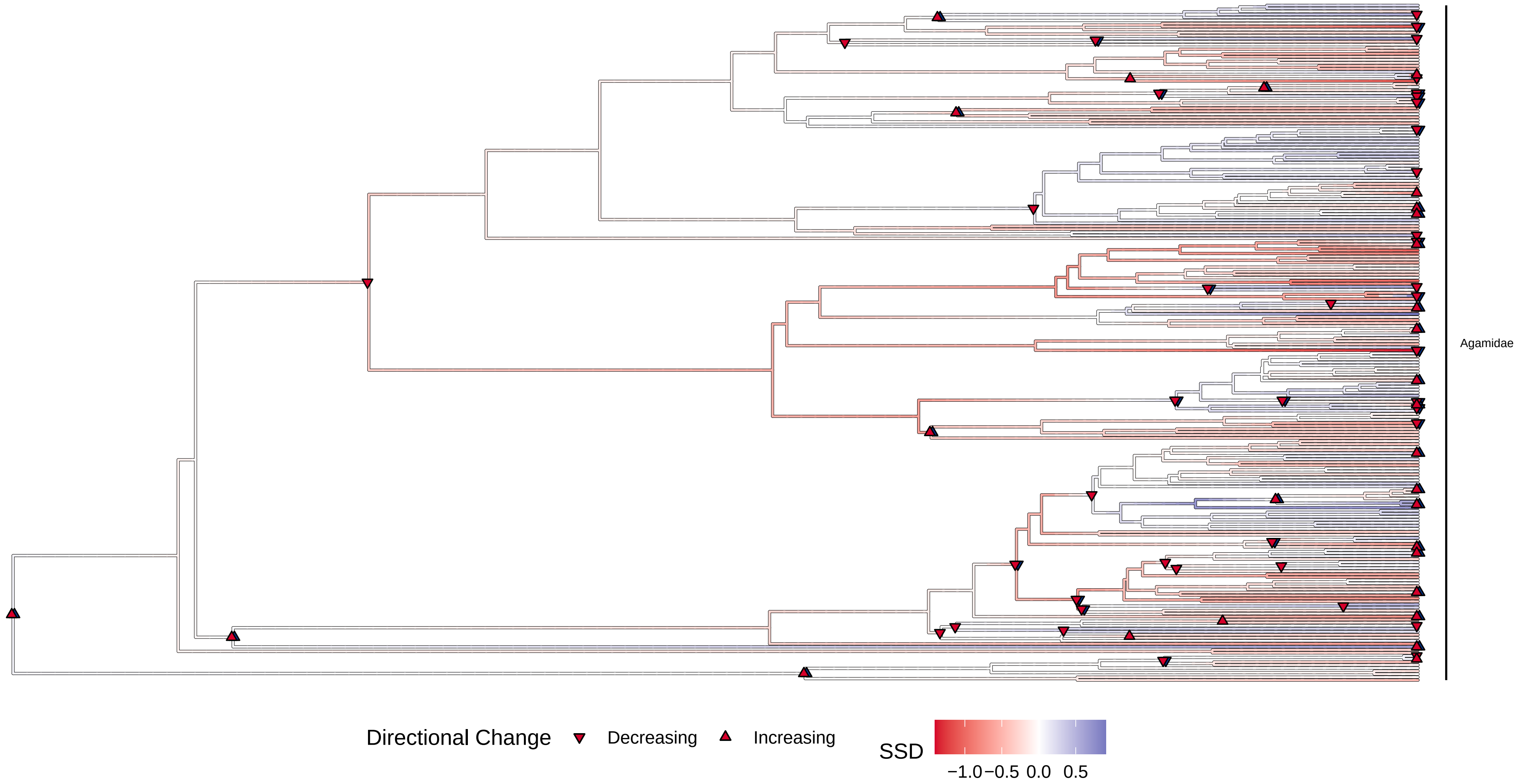

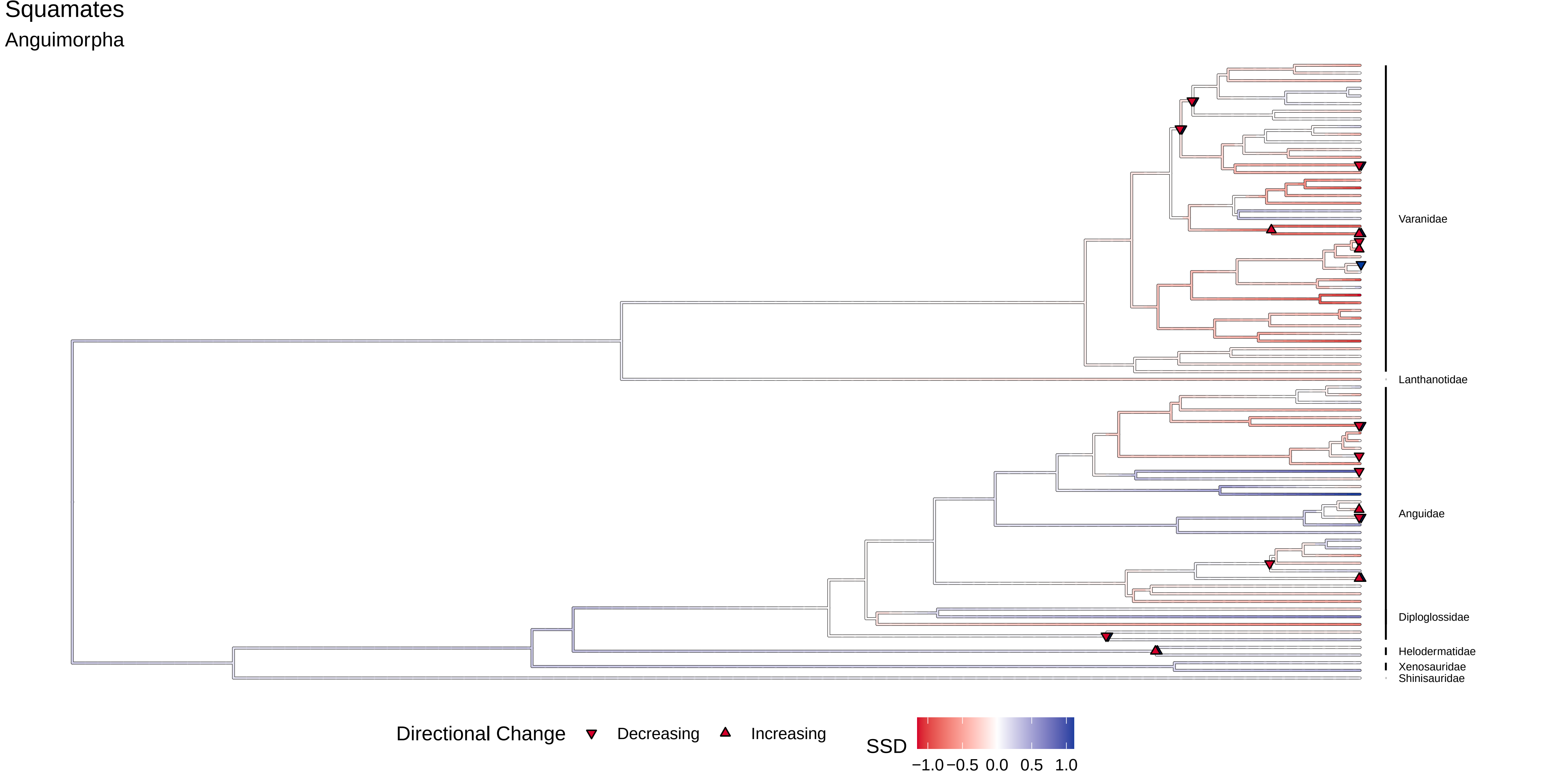

Squamates
Anguimorpha
Varanidae
Lanthanotidae
Anguidae
Diploglossidae
Helodermatidae
Xenosauridae
Shinisauridae
Directional Change
Decreasing
Increasing
SSD
−1.0
−0.5
0.0
0.5
1.0

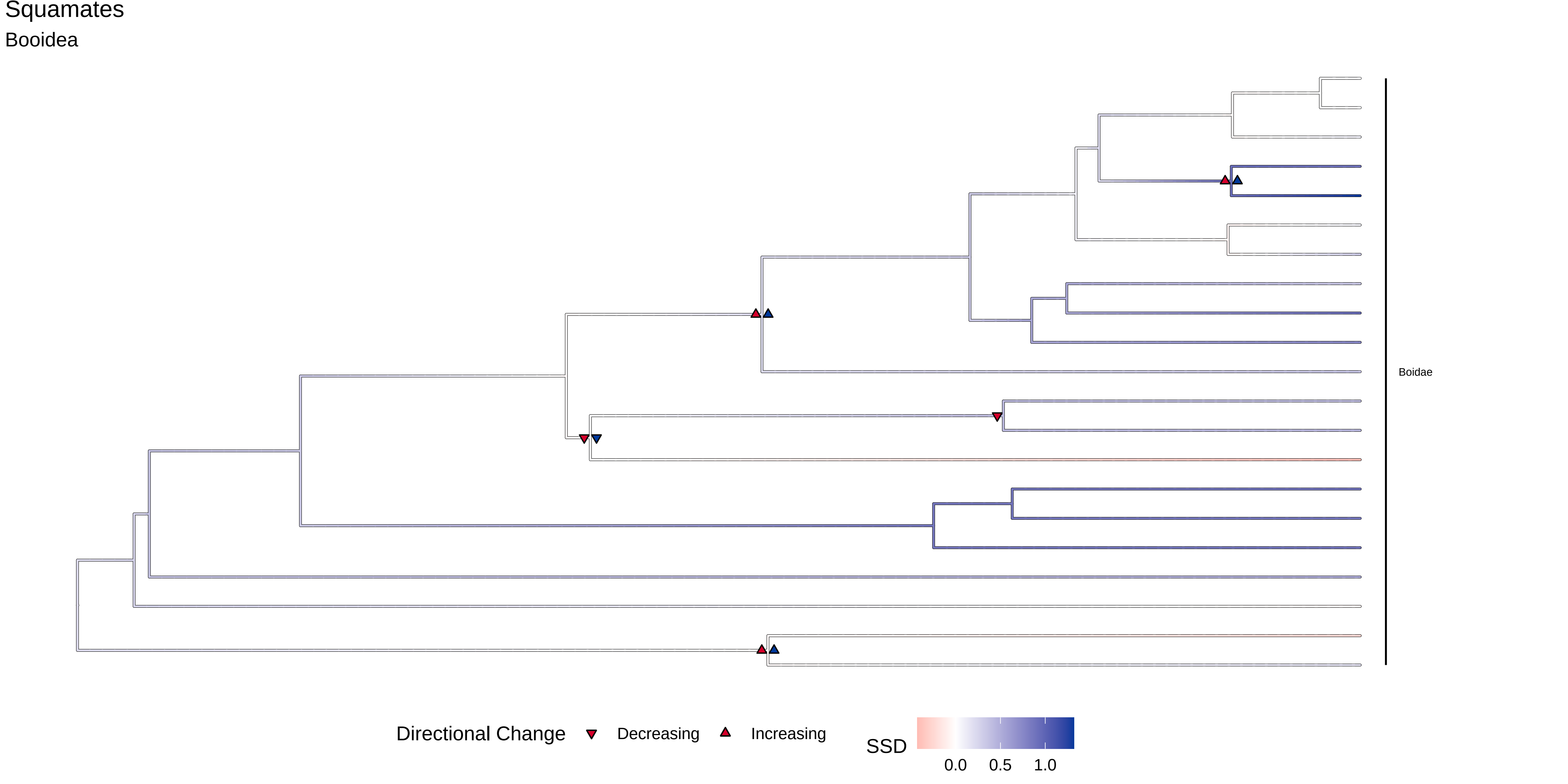

Squamates
Booidea
Boidae
Directional Change
Decreasing
Increasing
SSD
0.0
0.5
1.0

Squamates

Carphodactylidae

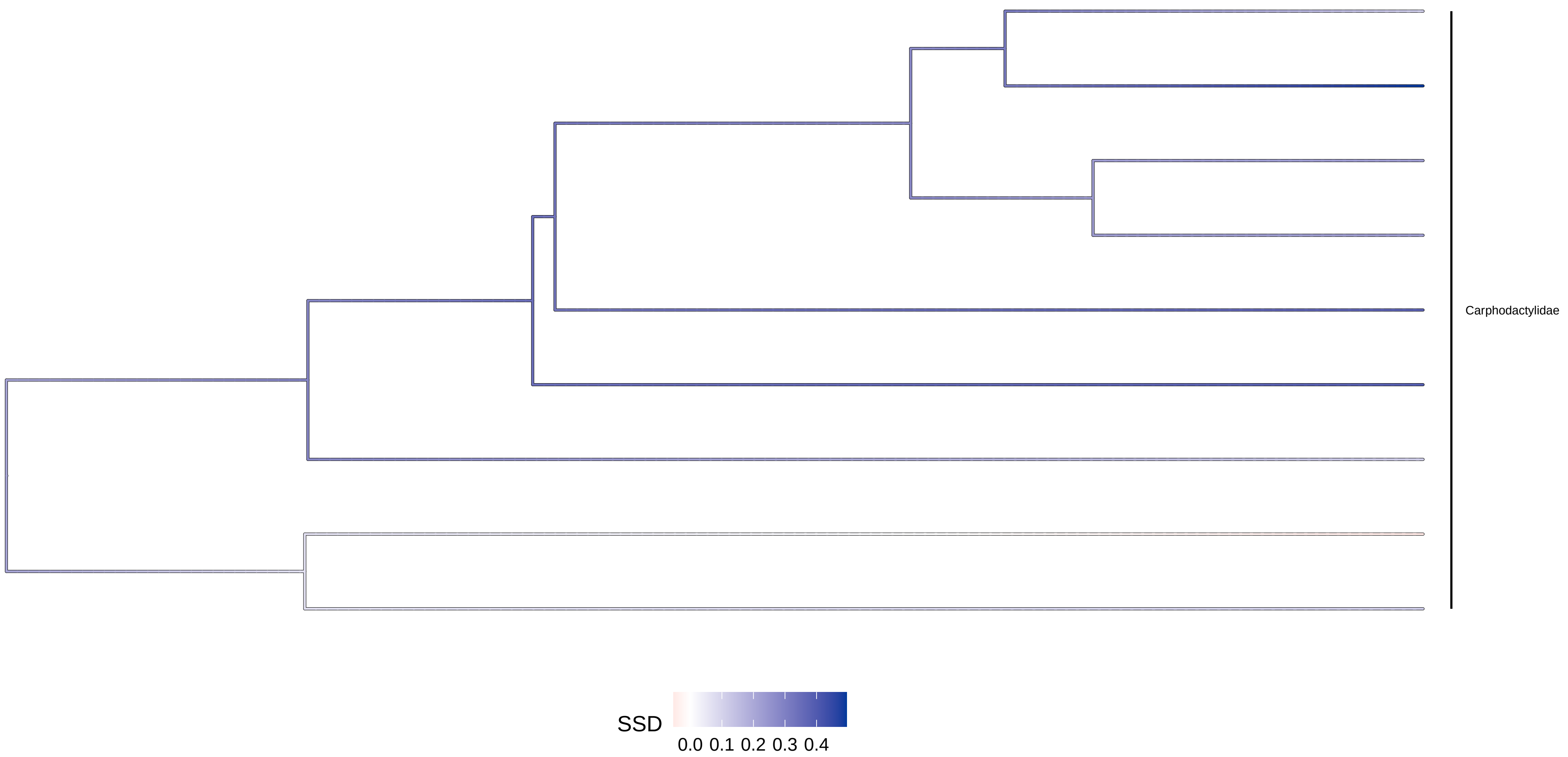

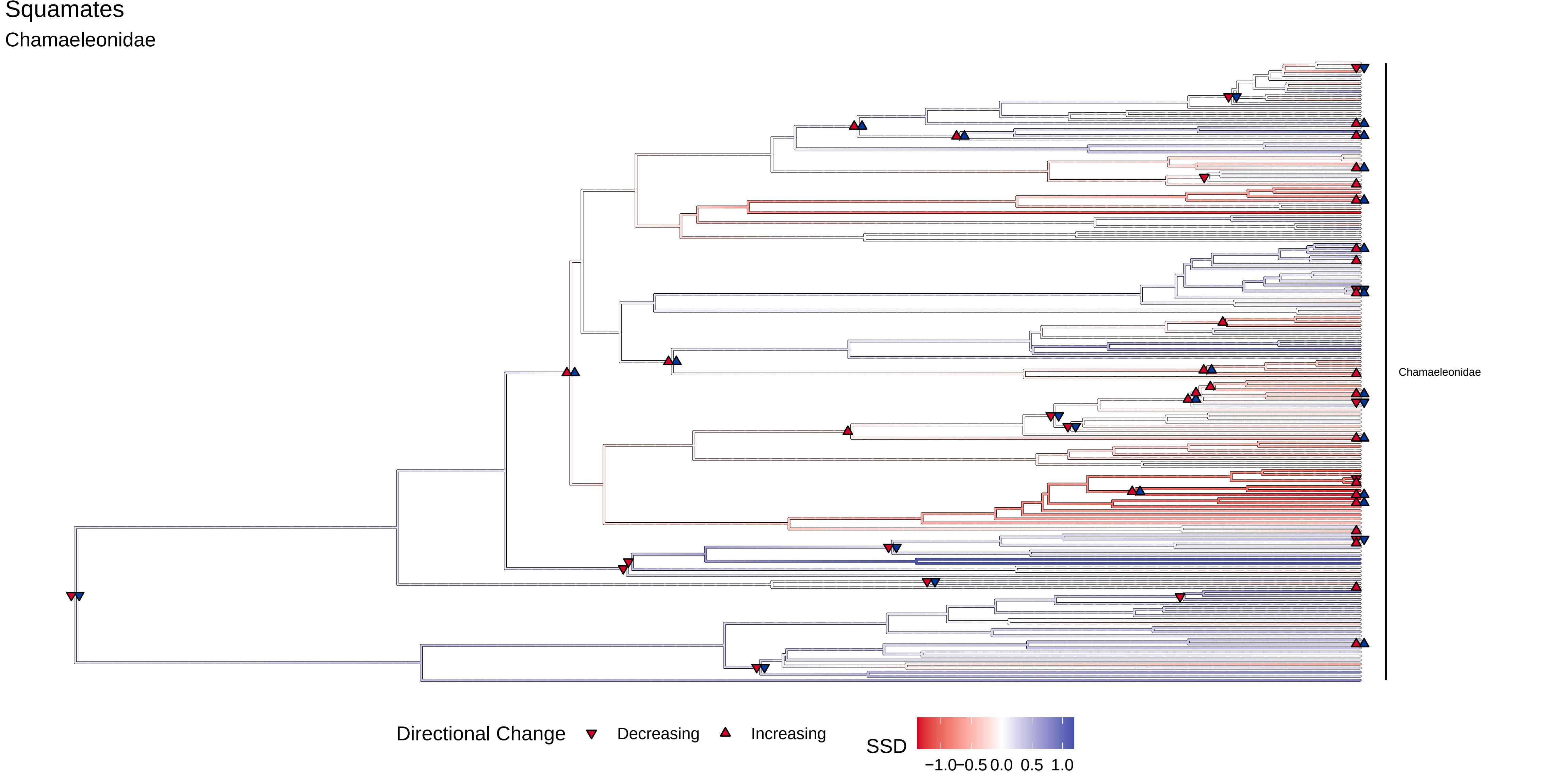

Squamates
Chamaeleonidae
Chamaeleonidae
Directional Change
Decreasing
Increasing
SSD
−1.0
−0.5
0.0
0.5
1.0

Squamates

Colubroidea

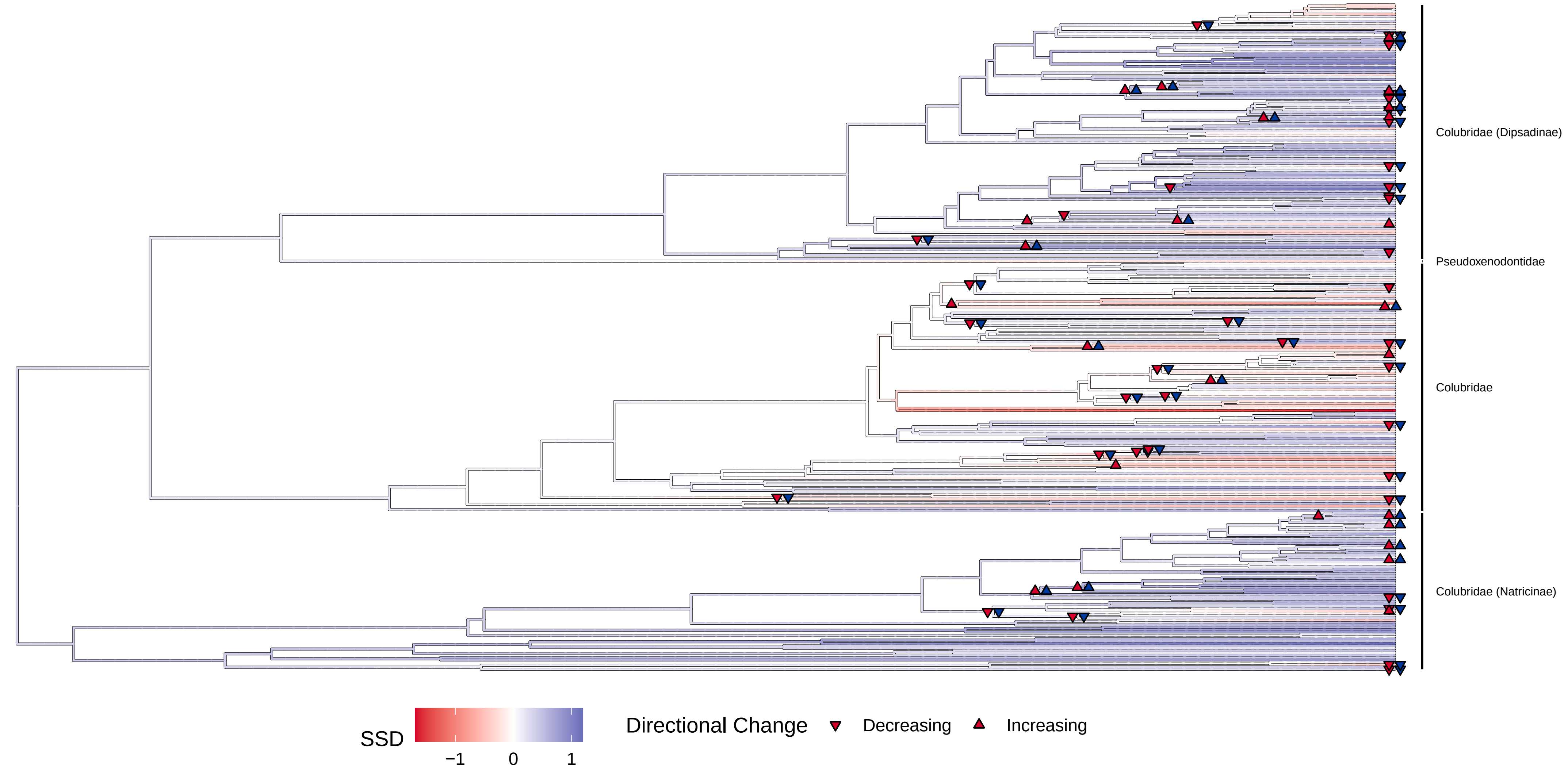

Squamates

Cordylidae

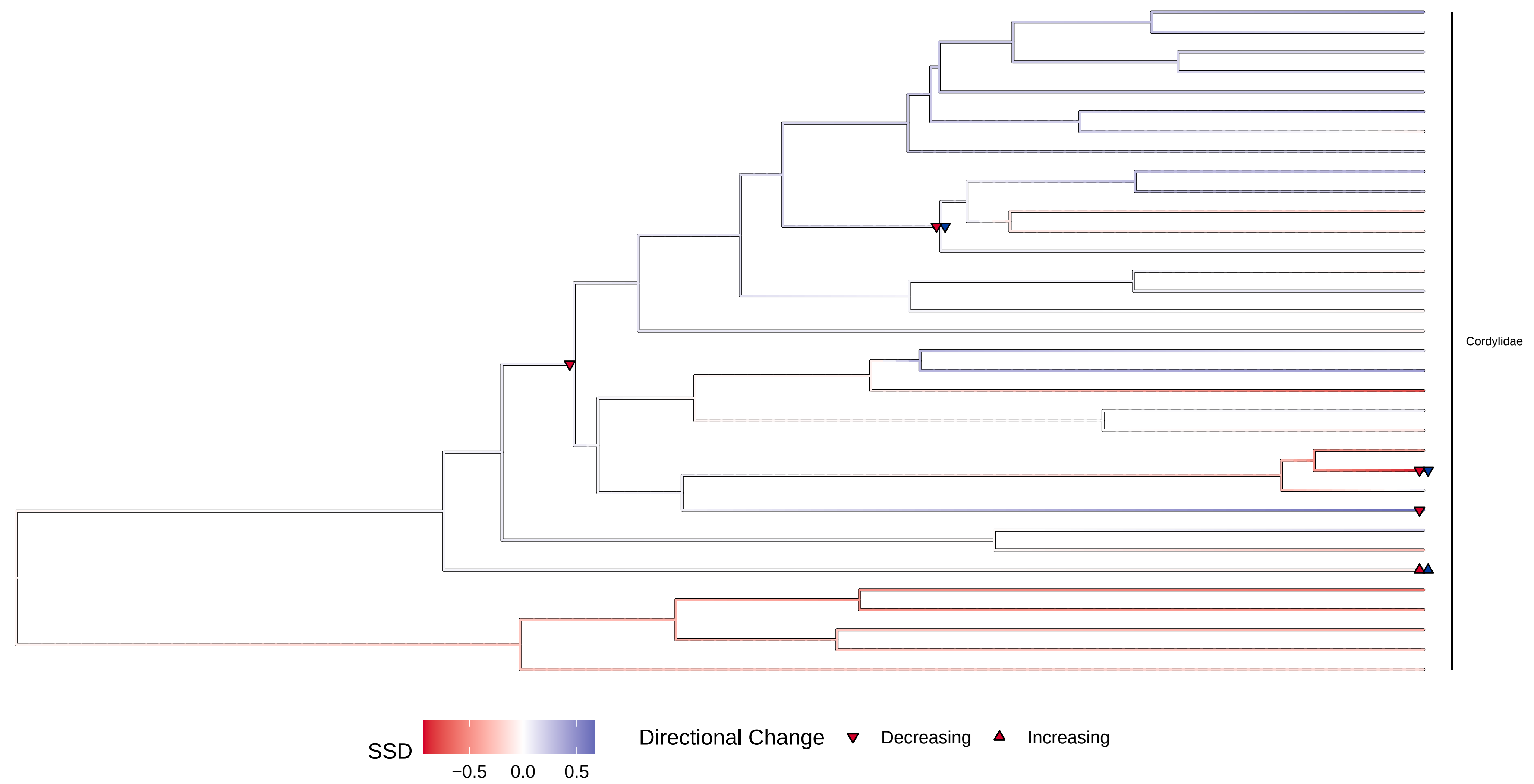

Squamates

Corytophanidae

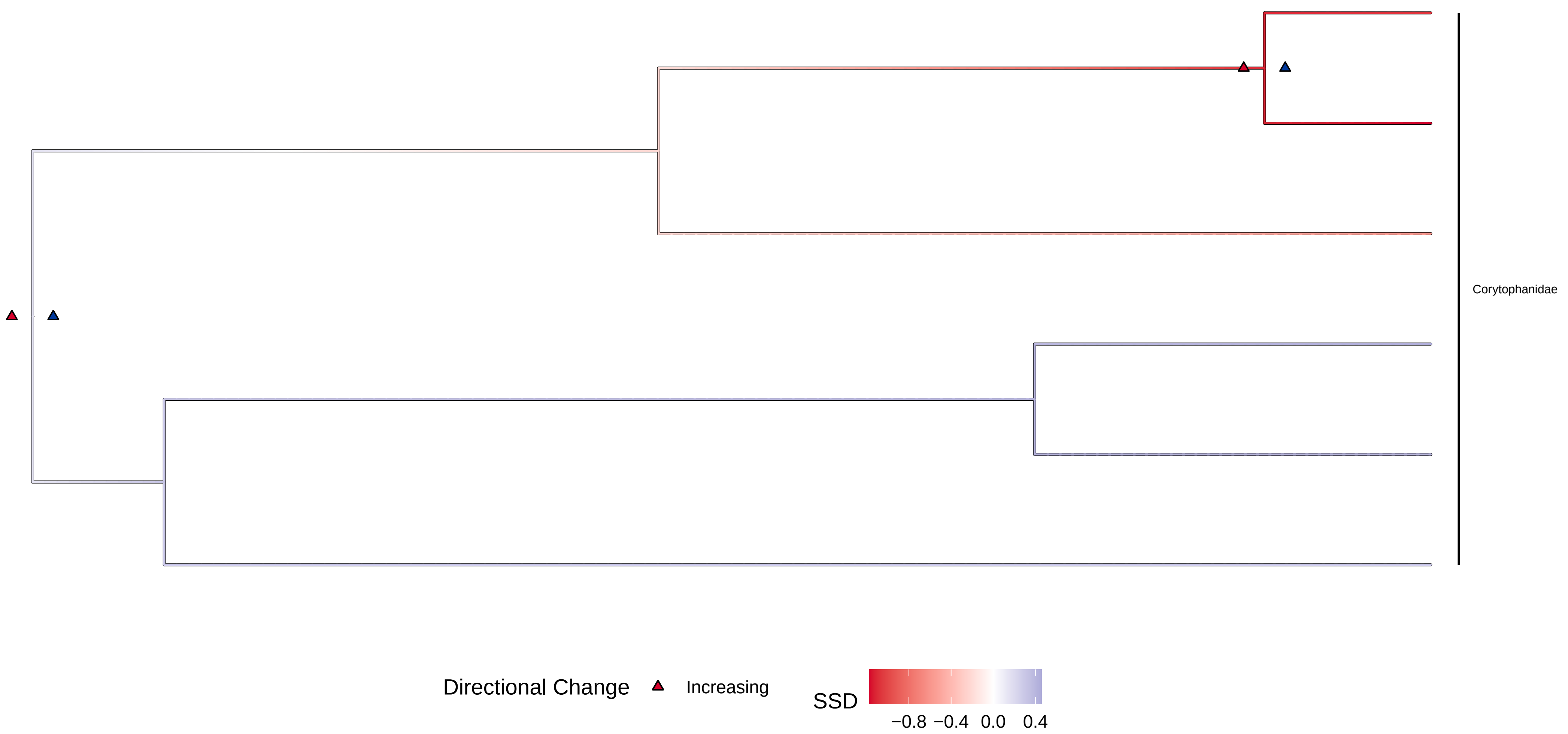

Squamates

Crotaphytidae

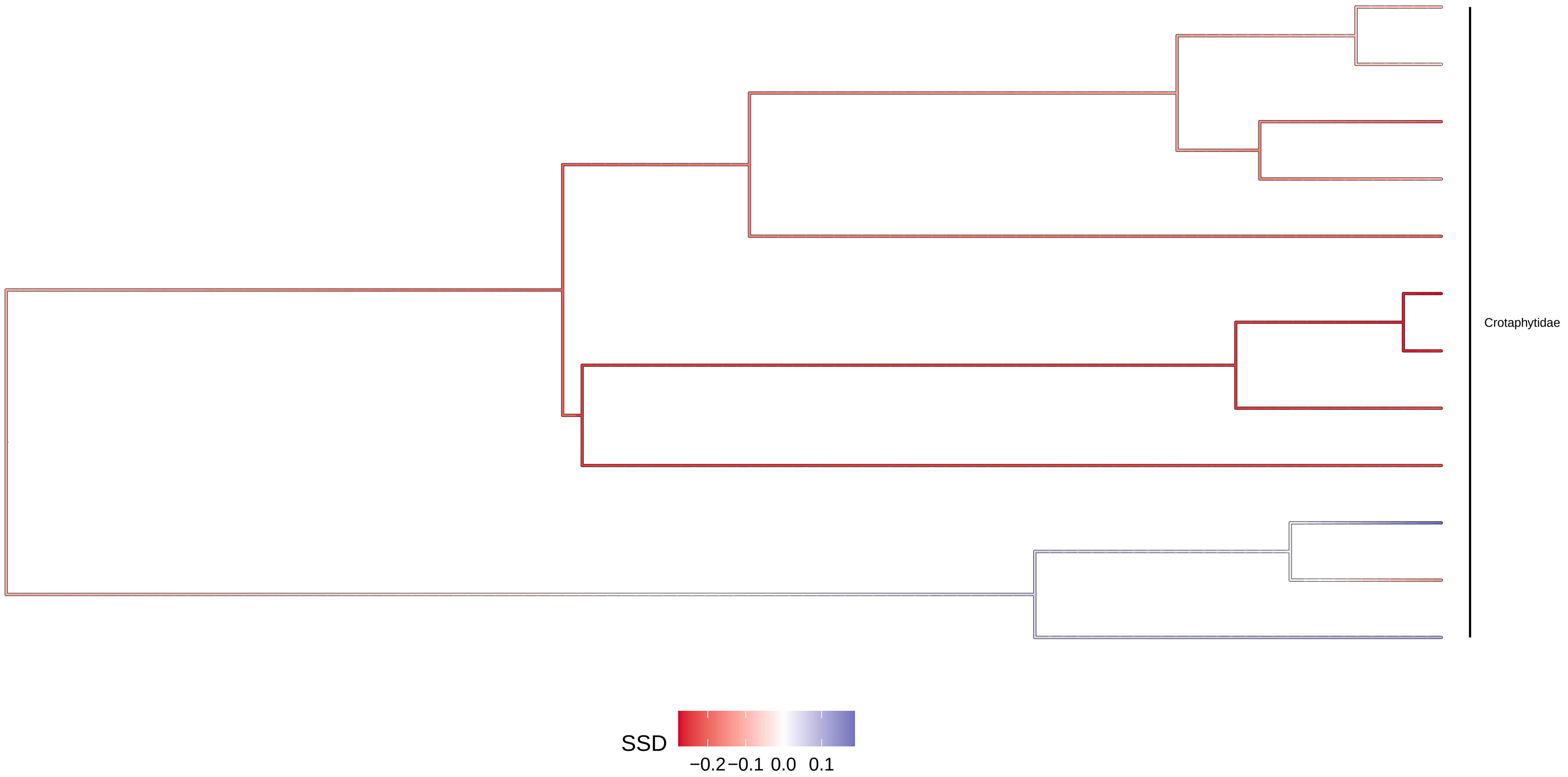

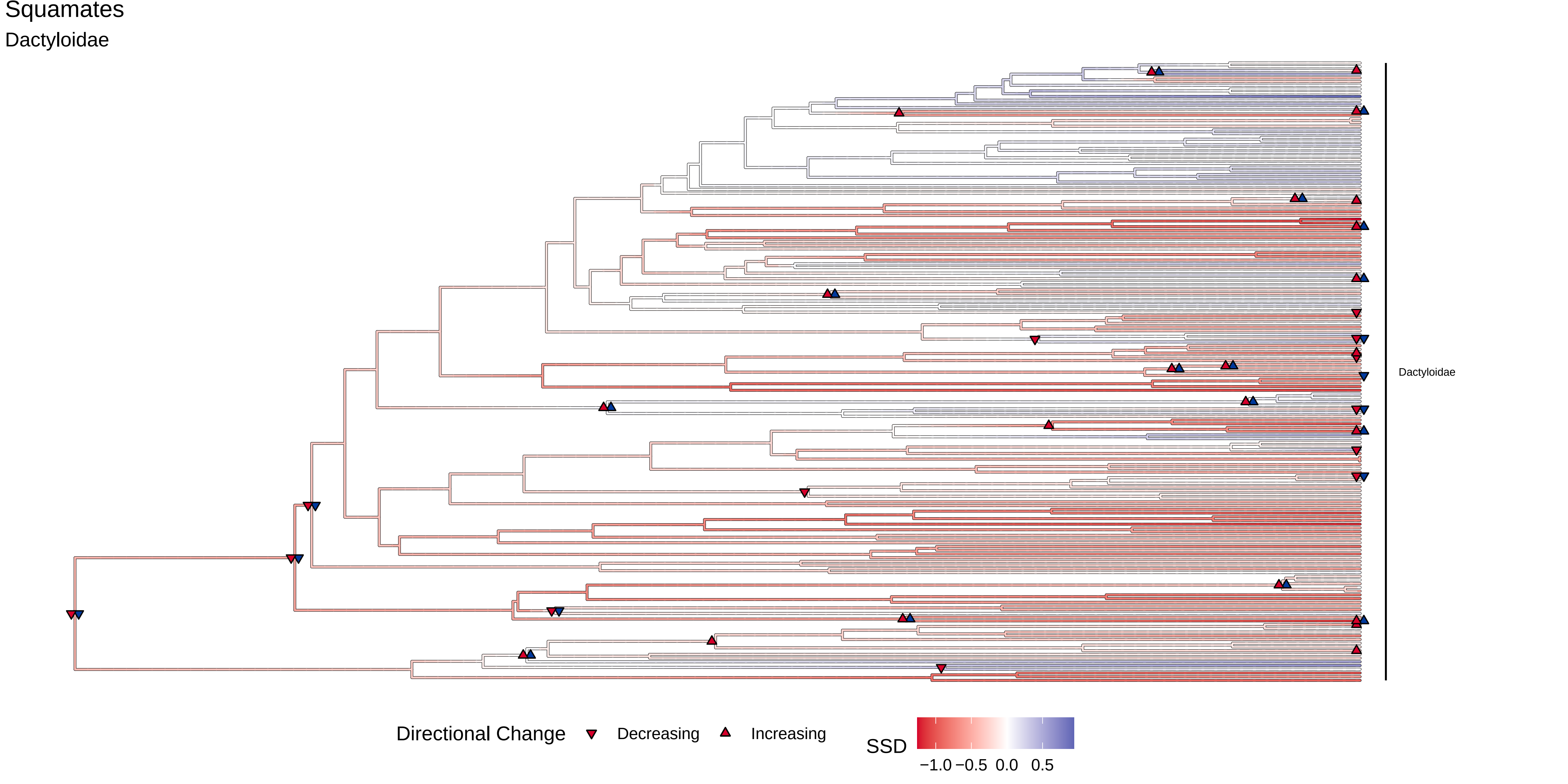
Squamates
Dactyloidae
Dactyloidae
Directional Change
Decreasing
Increasing
SSD
−1.0 −0.5 0.0 0.5

Squamates

Diplodactylidae

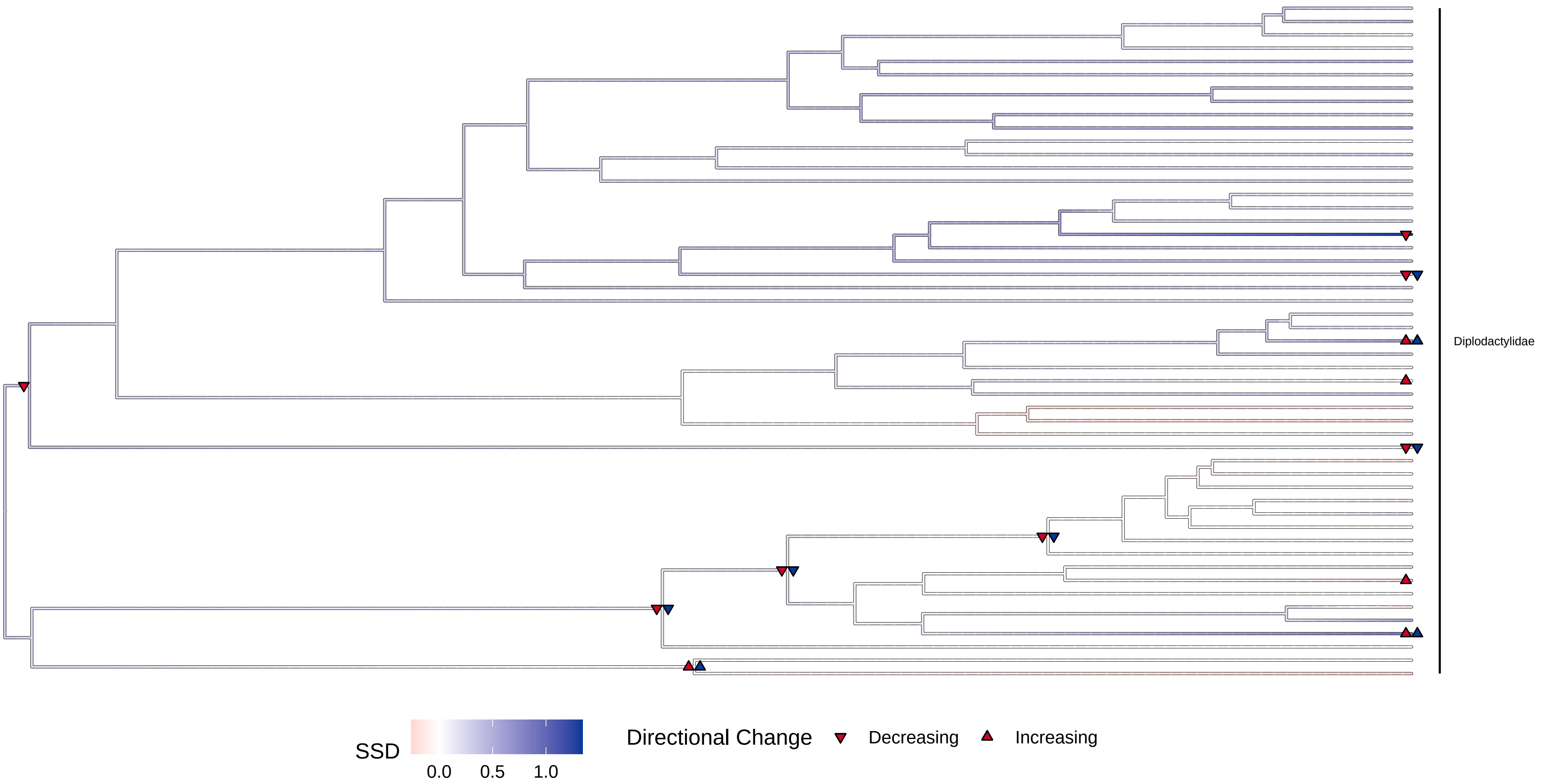

Squamates

Elapoidea

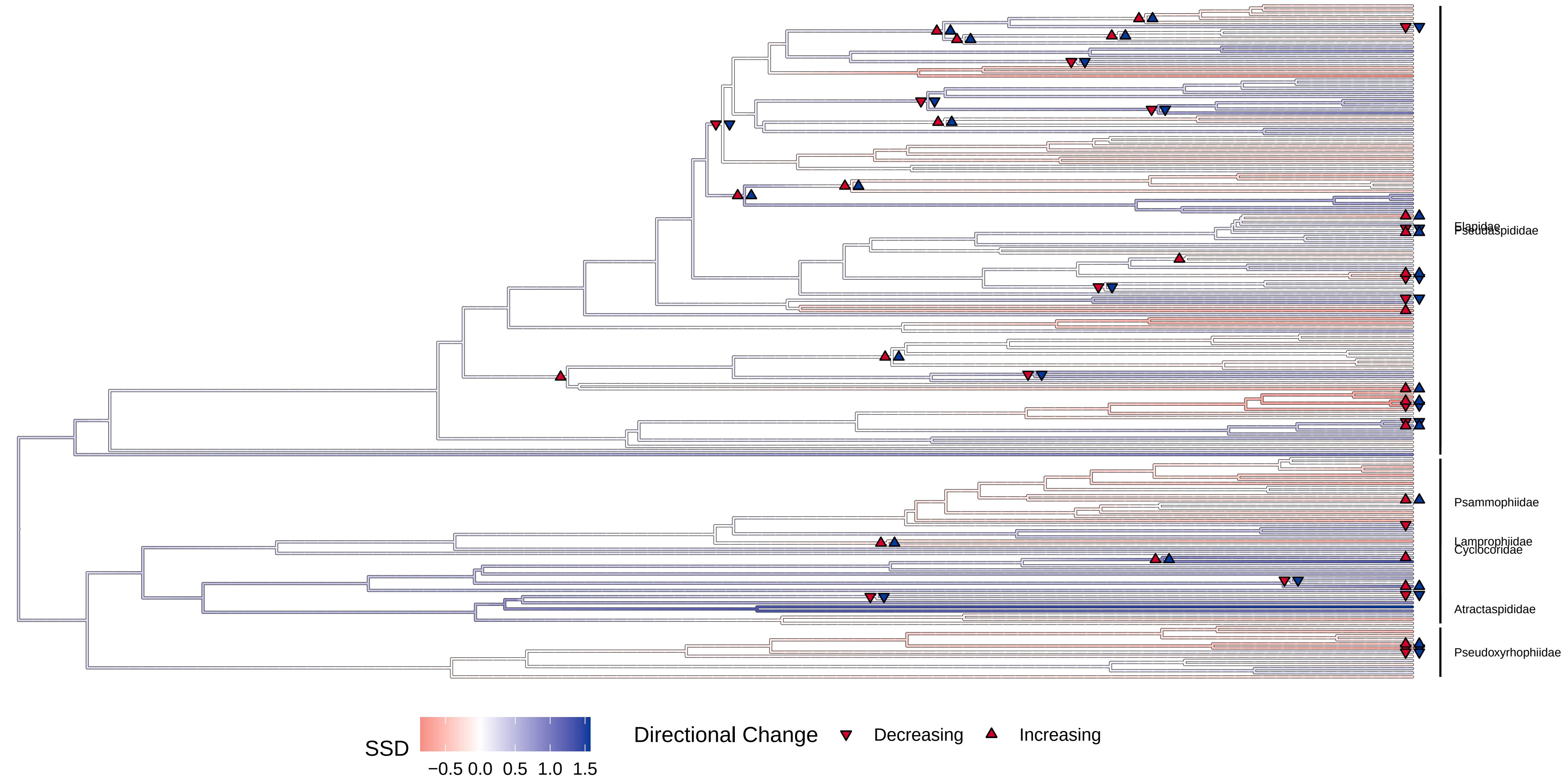

Squamates

Eublepharidae

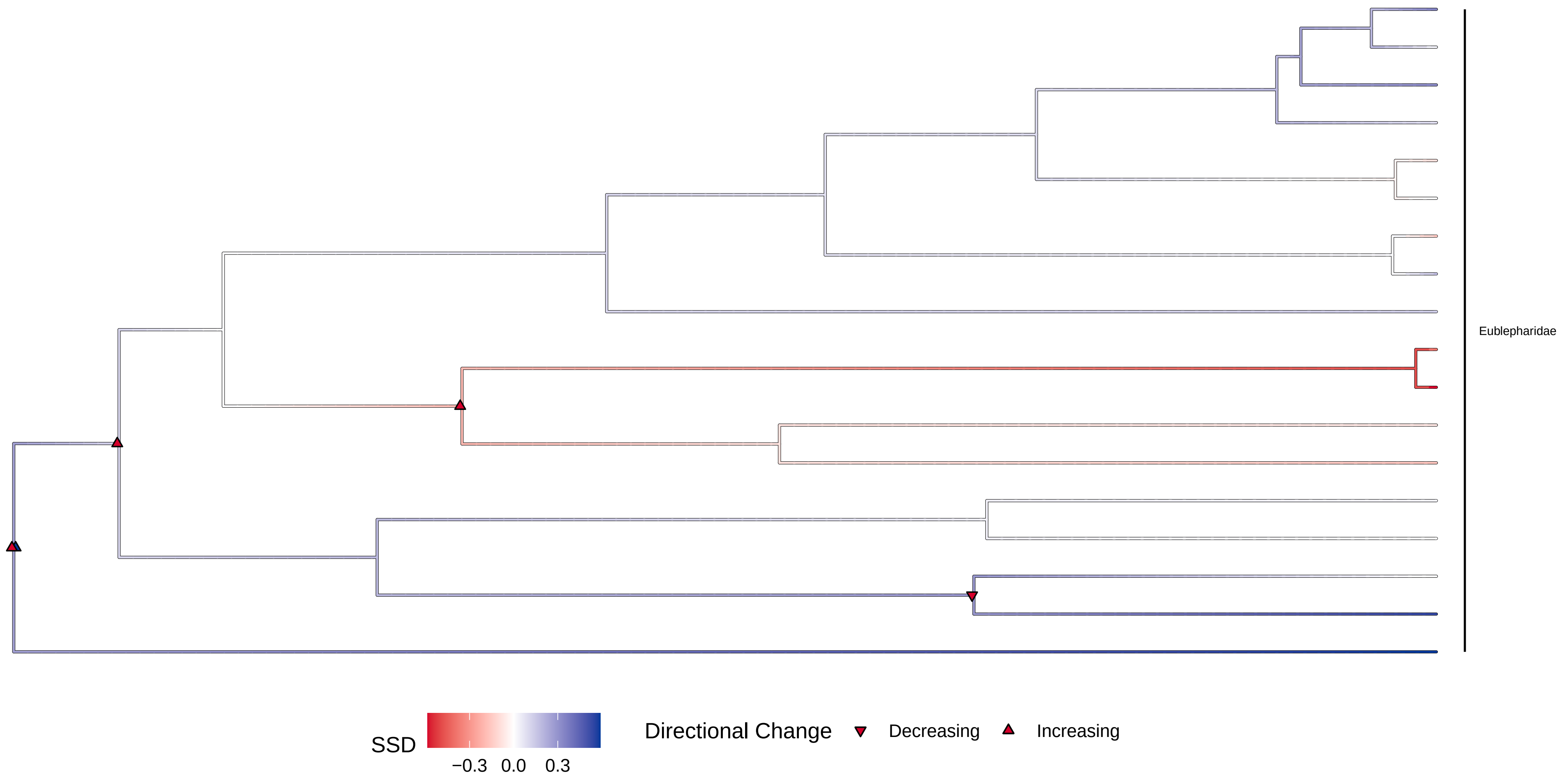

Squamates

Gekkonidae

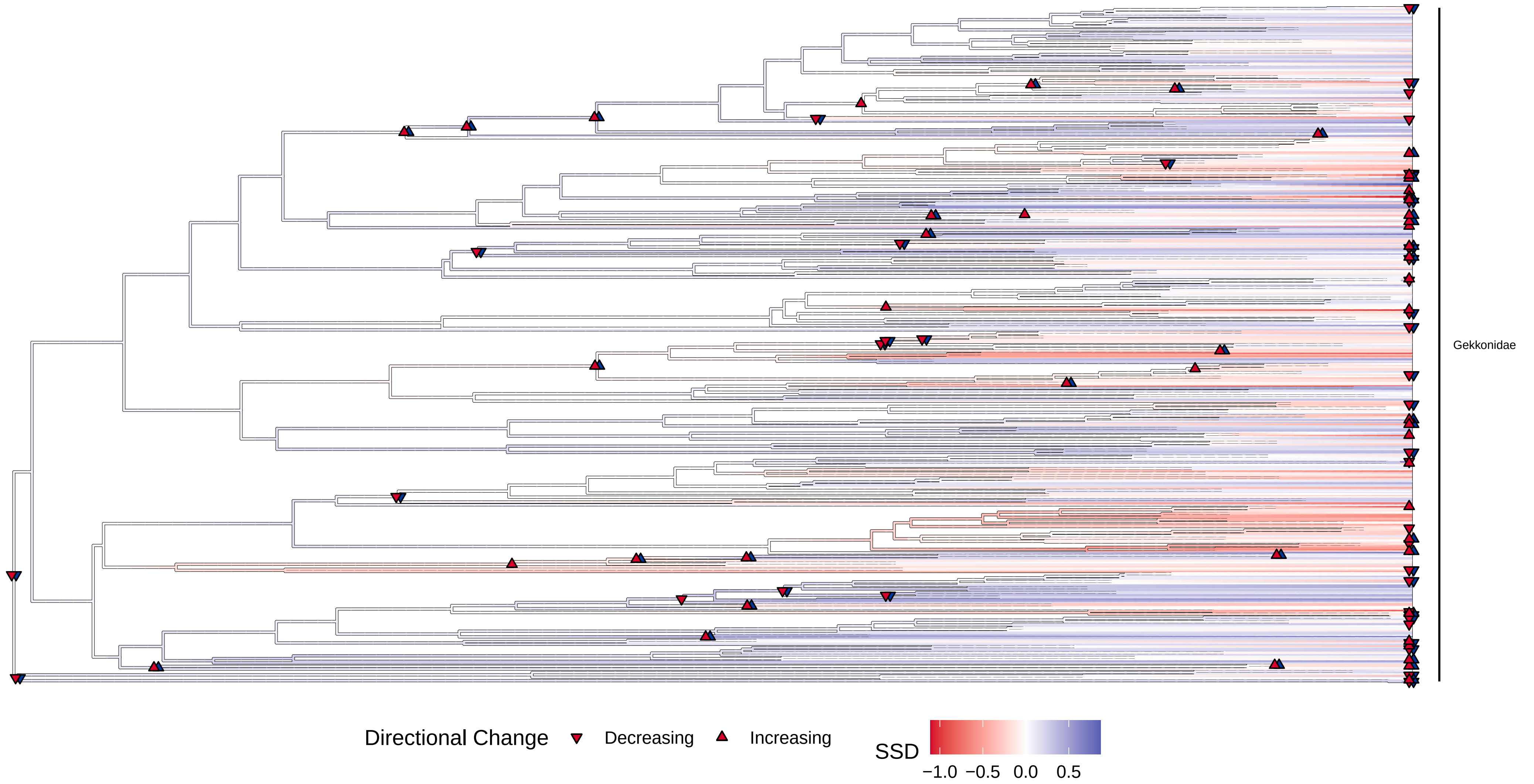

Squamates

Gerrhosauridae

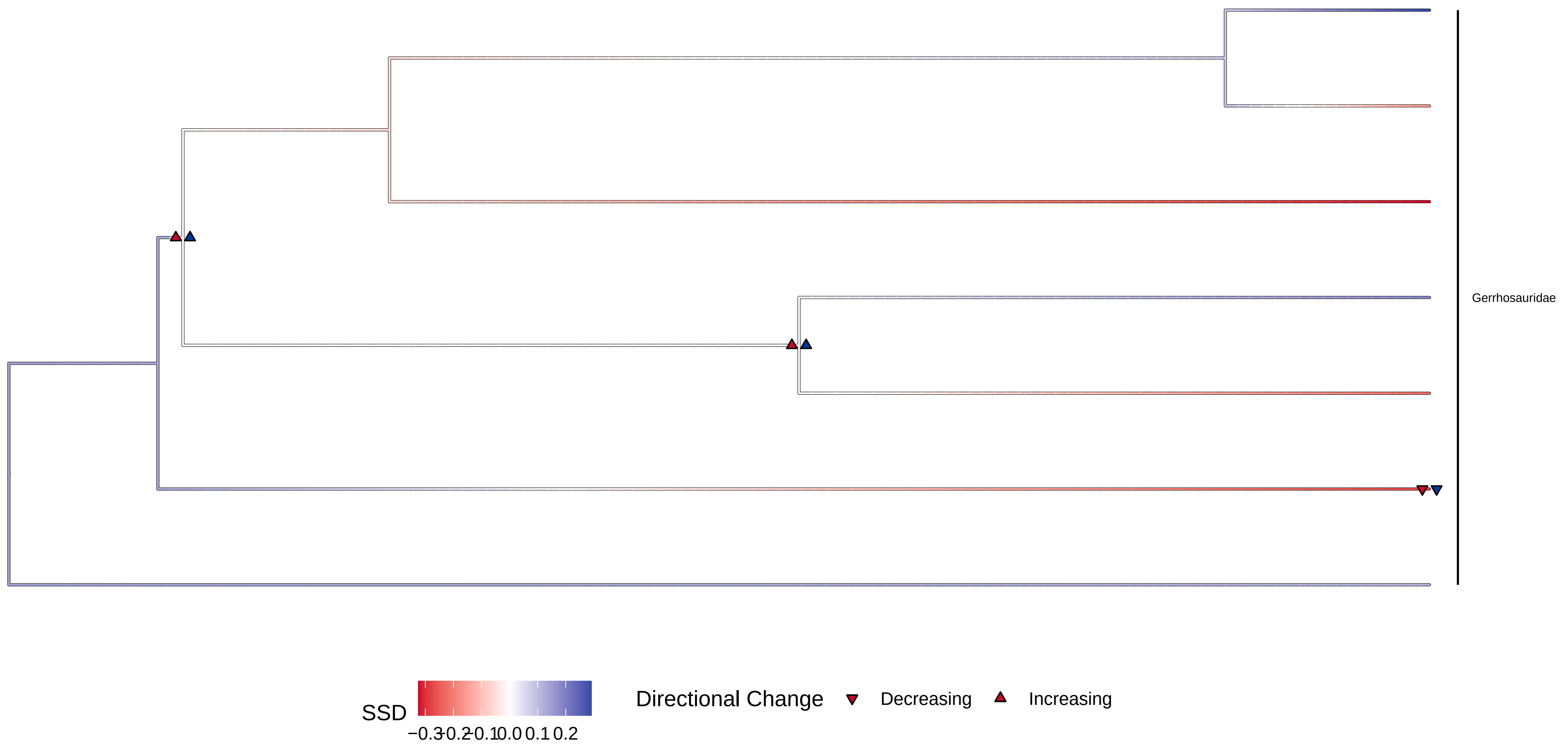

Squamates

Homalopsidae

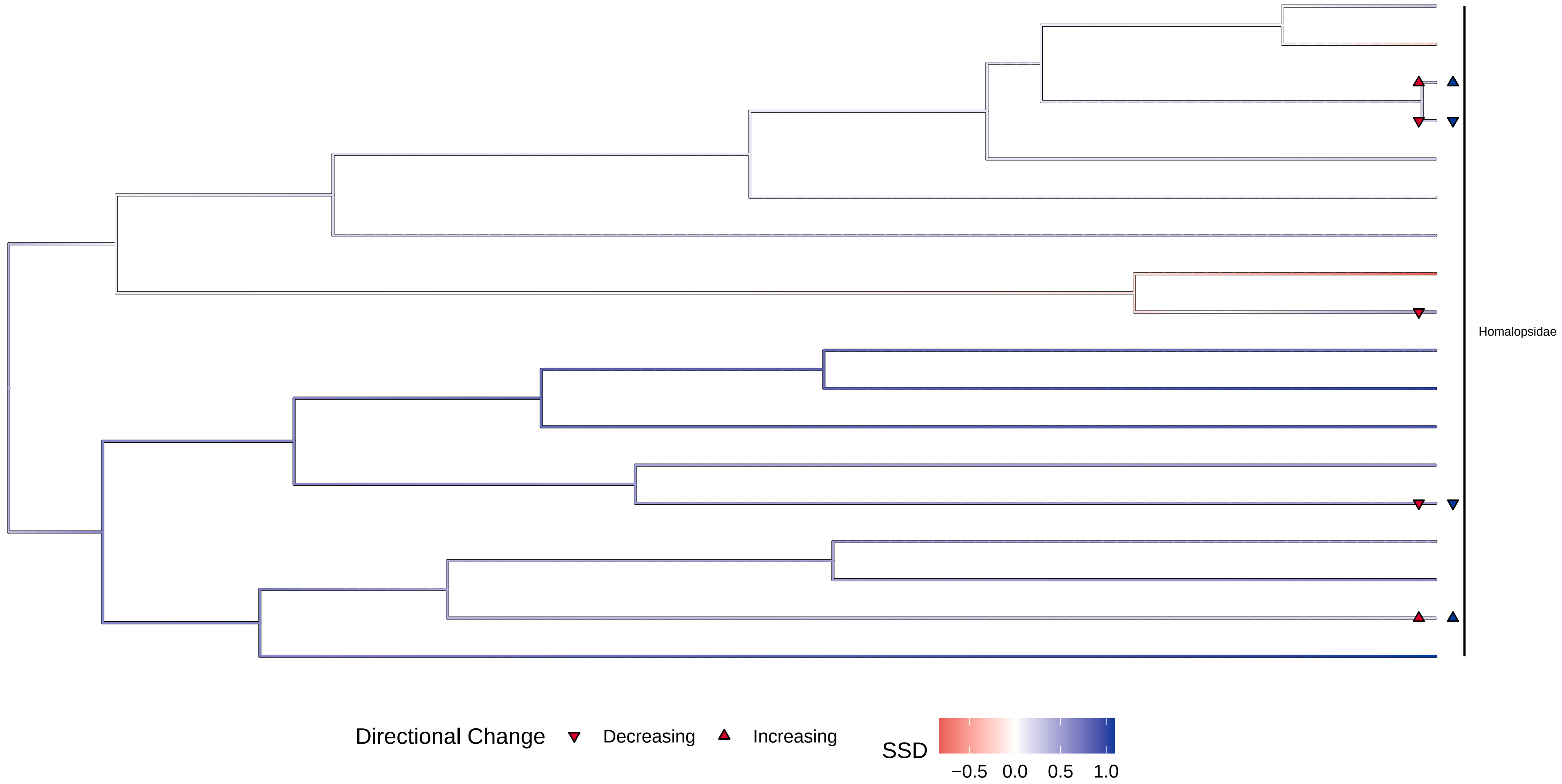

Squamates

Hoplocercidae

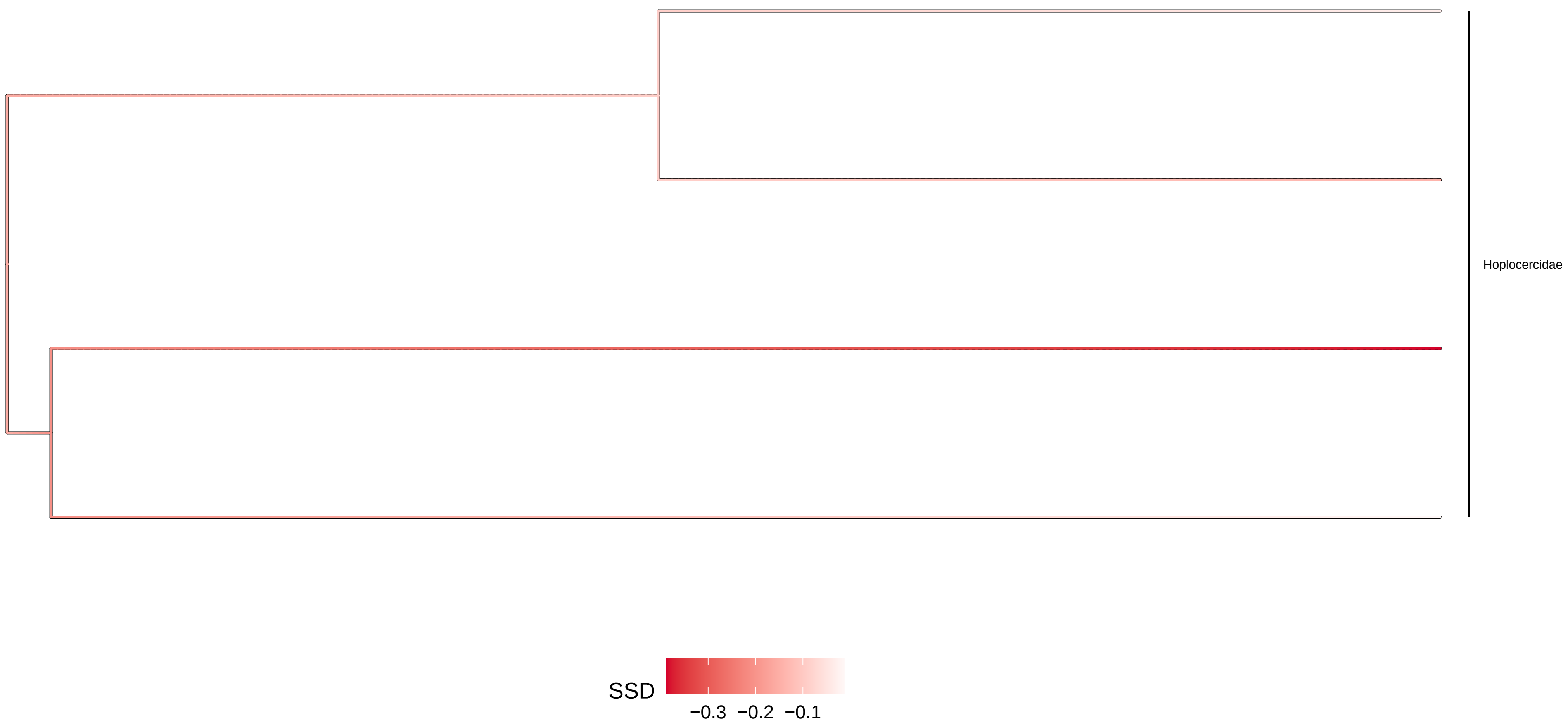

Squamates

Iguanidae

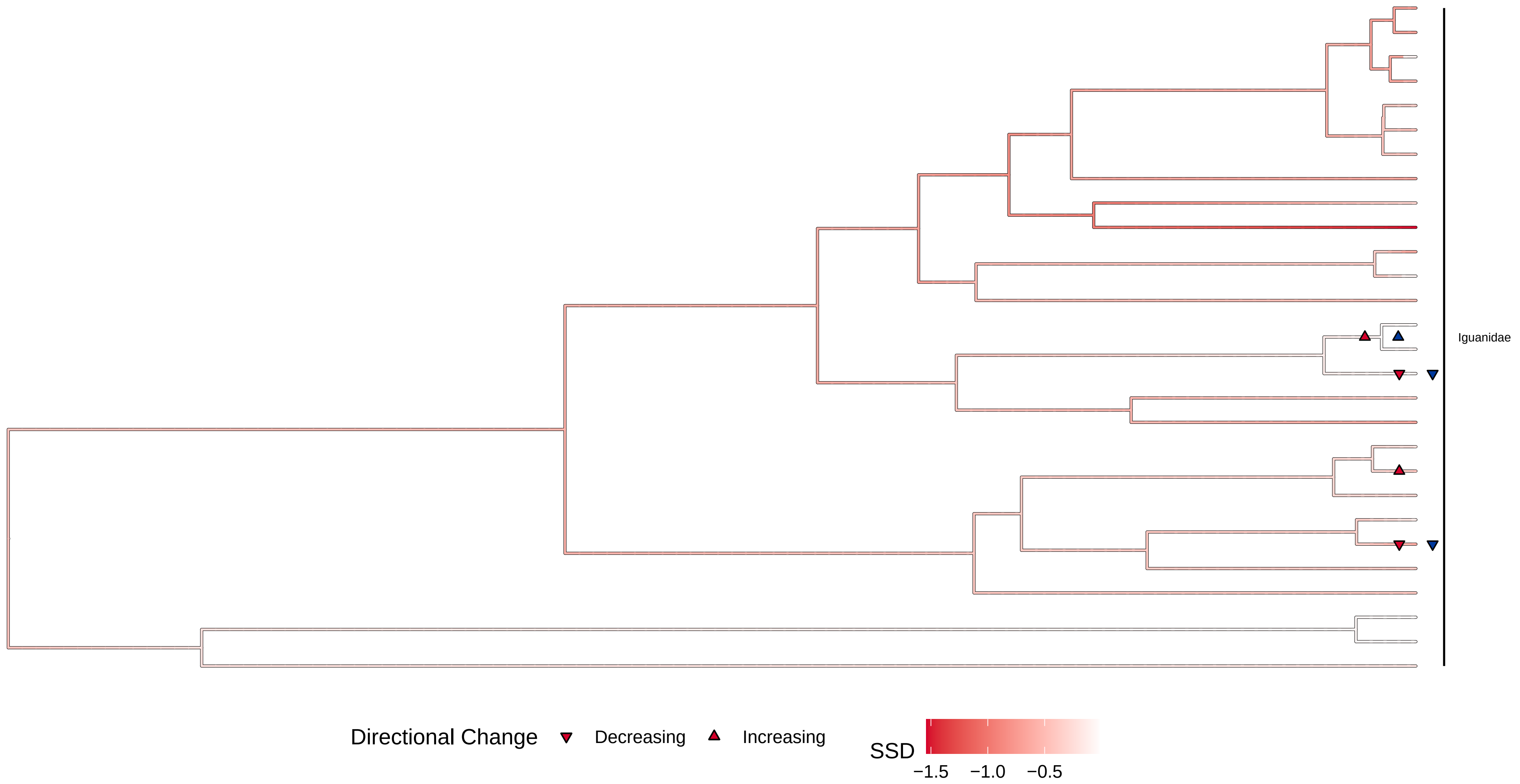

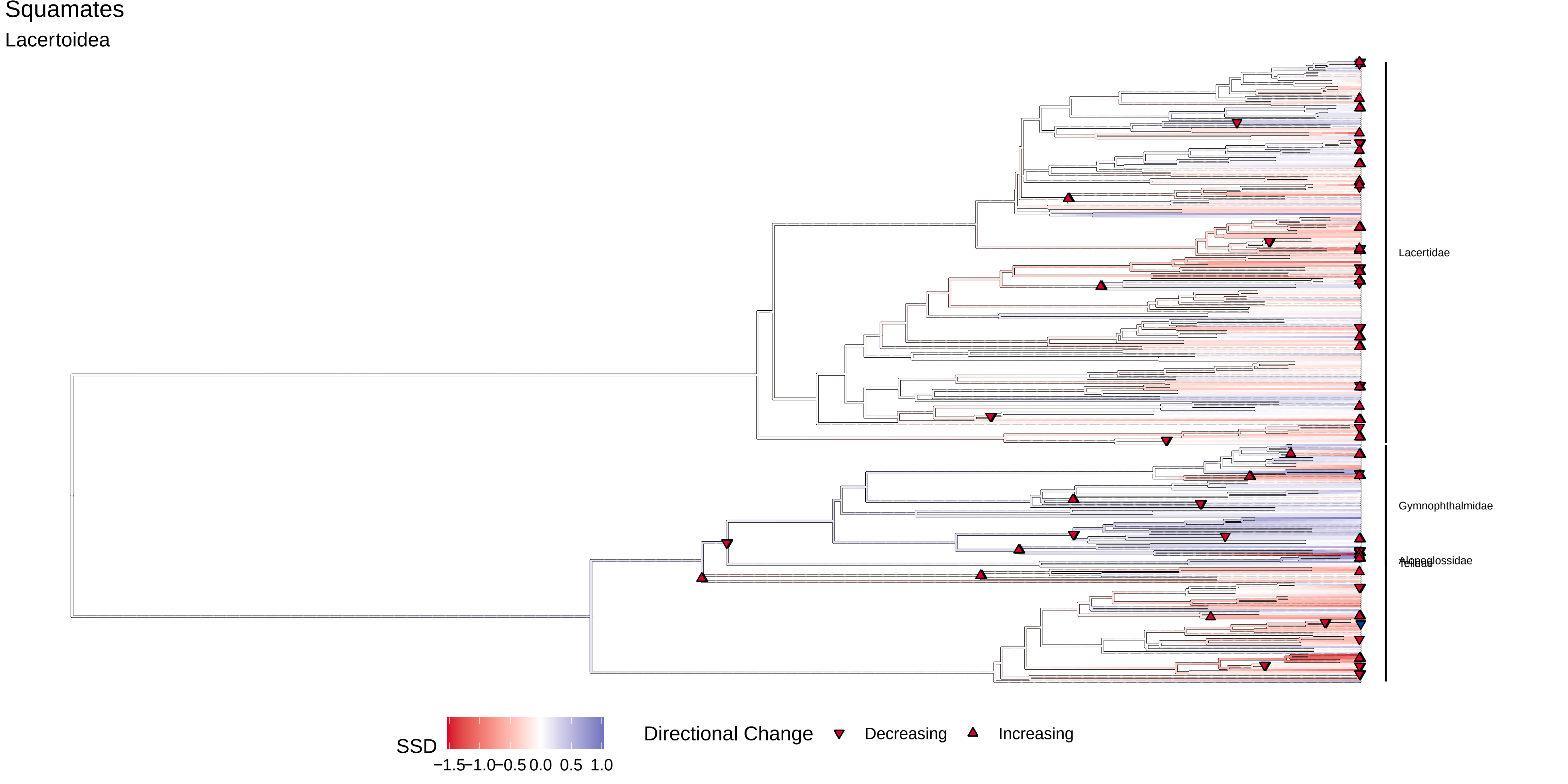
Squamates
Lacertoidea
Lacertidae
Gymnophthalmidae
Alopoglossidae
Teiidae
SSD
−1.5
−1.0
−0.5
0.0
0.5
1.0
Directional Change
Decreasing
Increasing

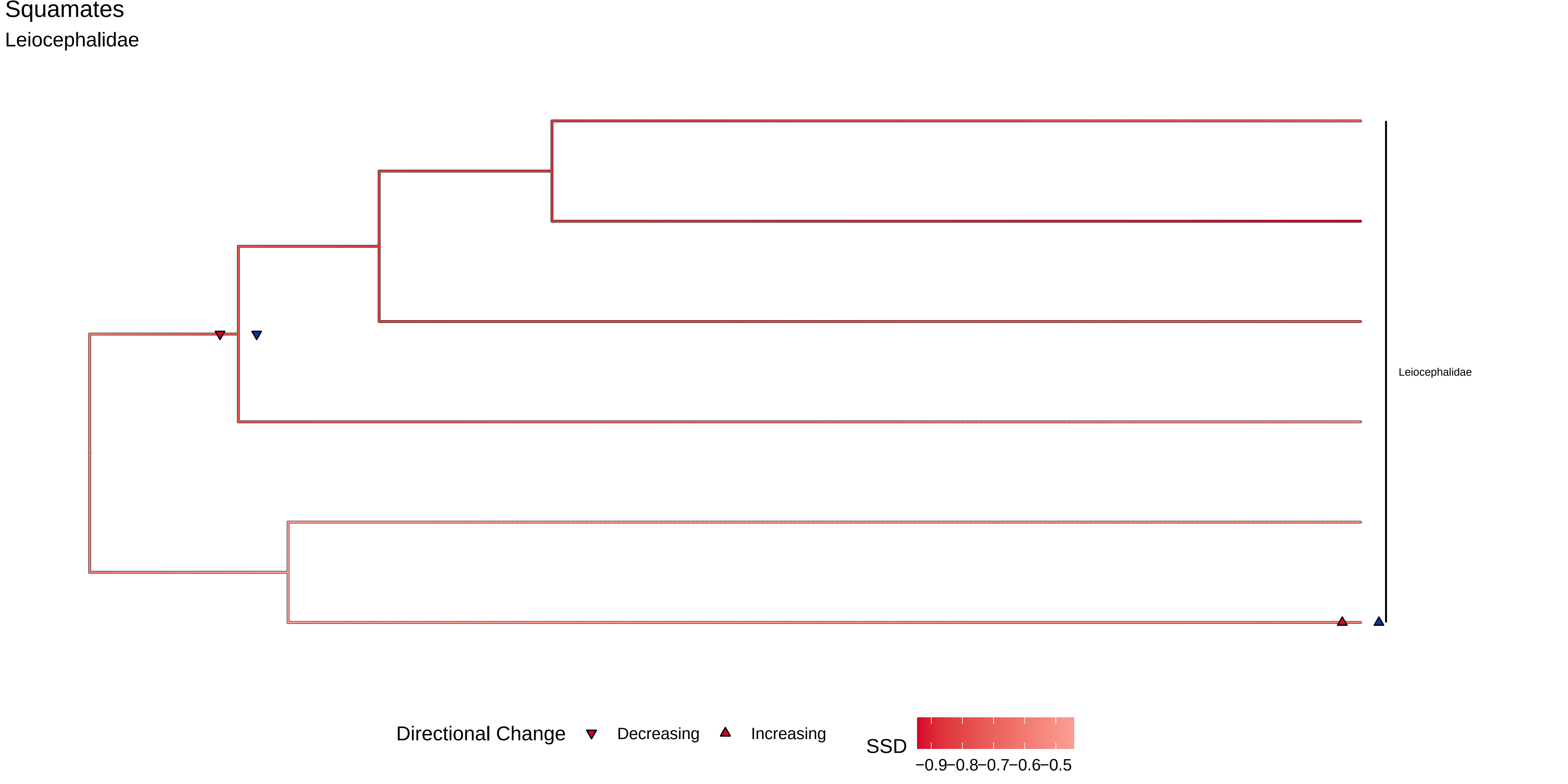
Squamates
Leiocephalidae
Leiocephalidae
Directional Change
Decreasing
Increasing
SSD
−0.9−0.8−0.7−0.6−0.5

Squamates

Leiosauridae

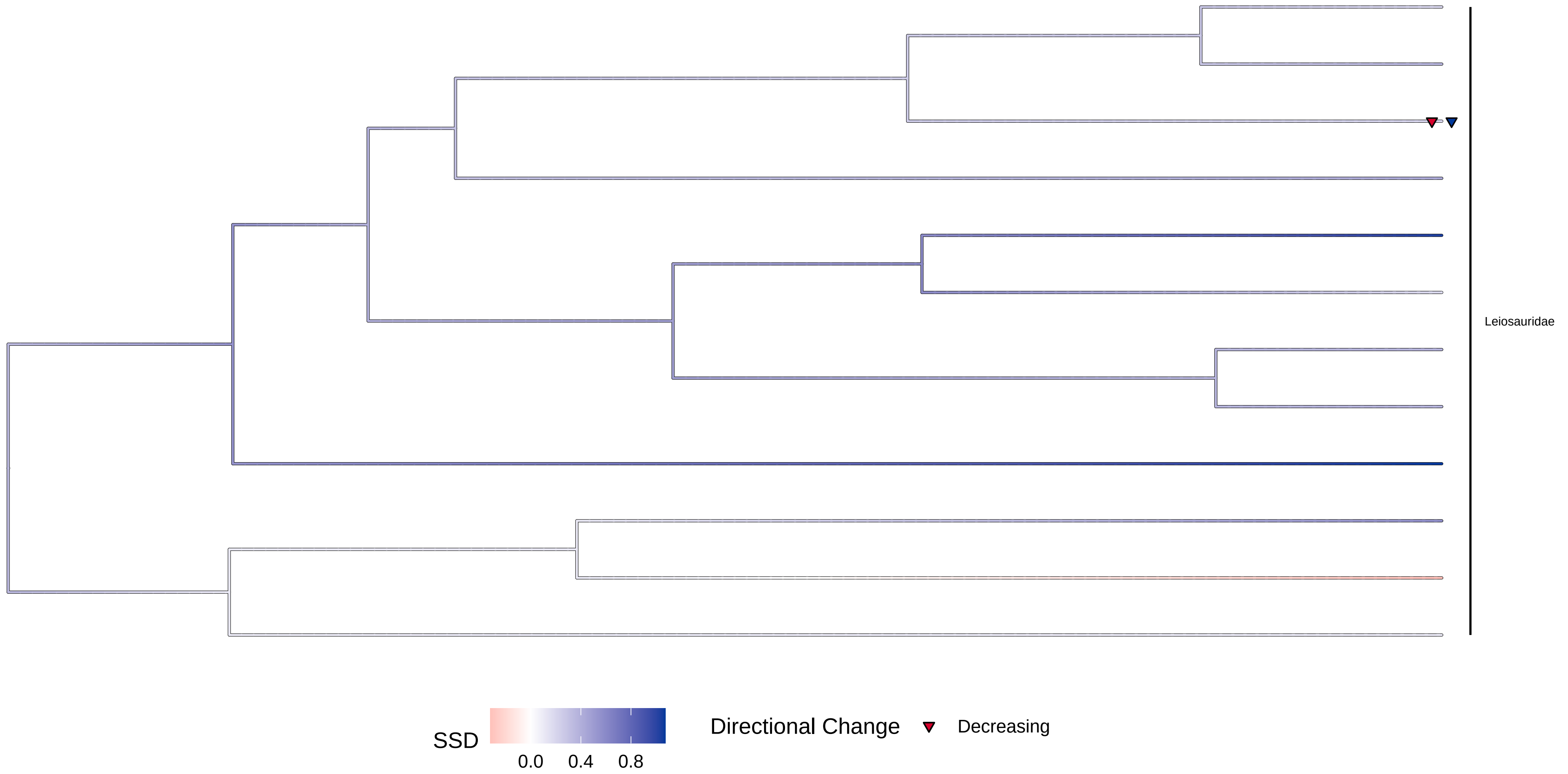

Squamates

Liolaemidae

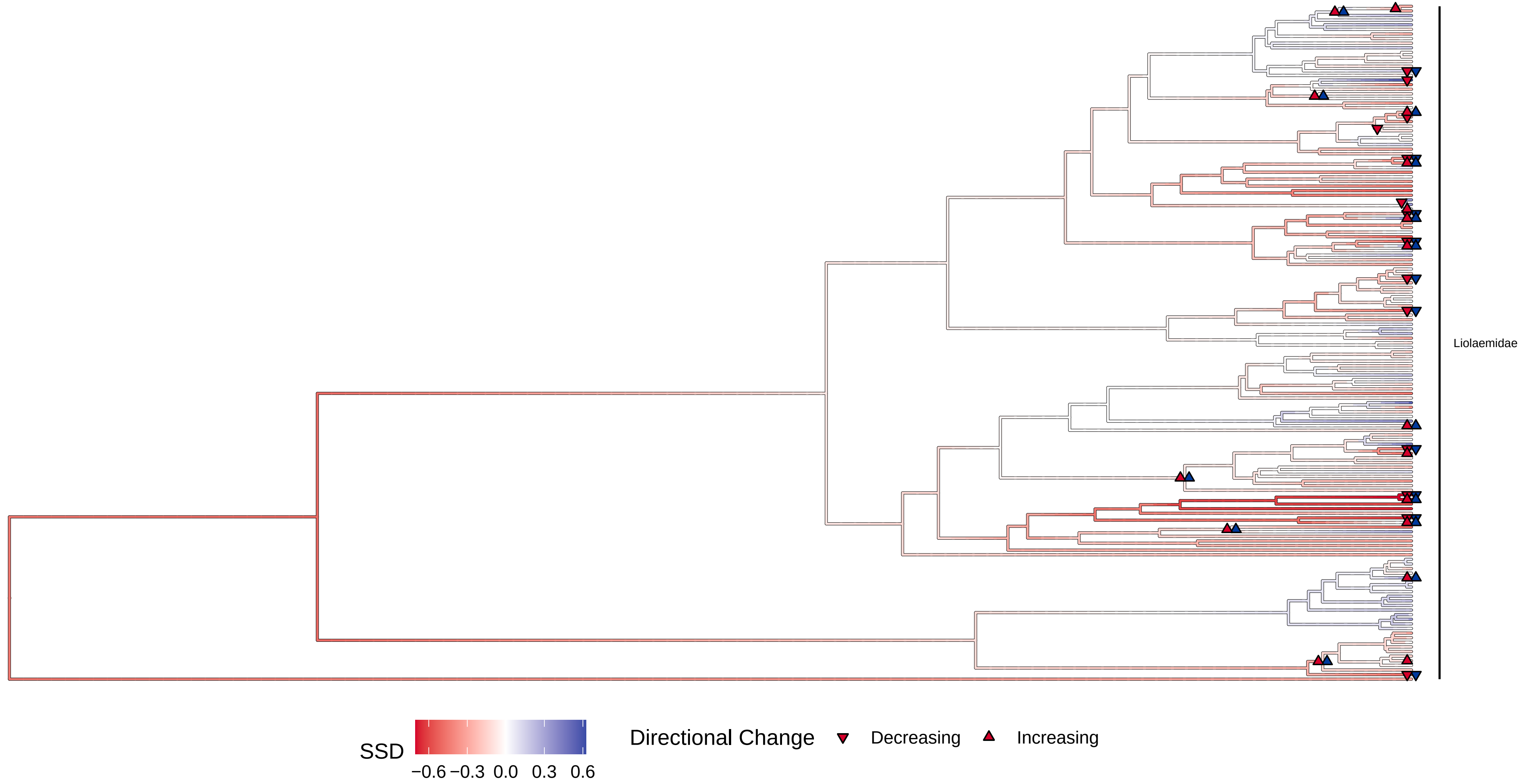

Squamates

Opluridae

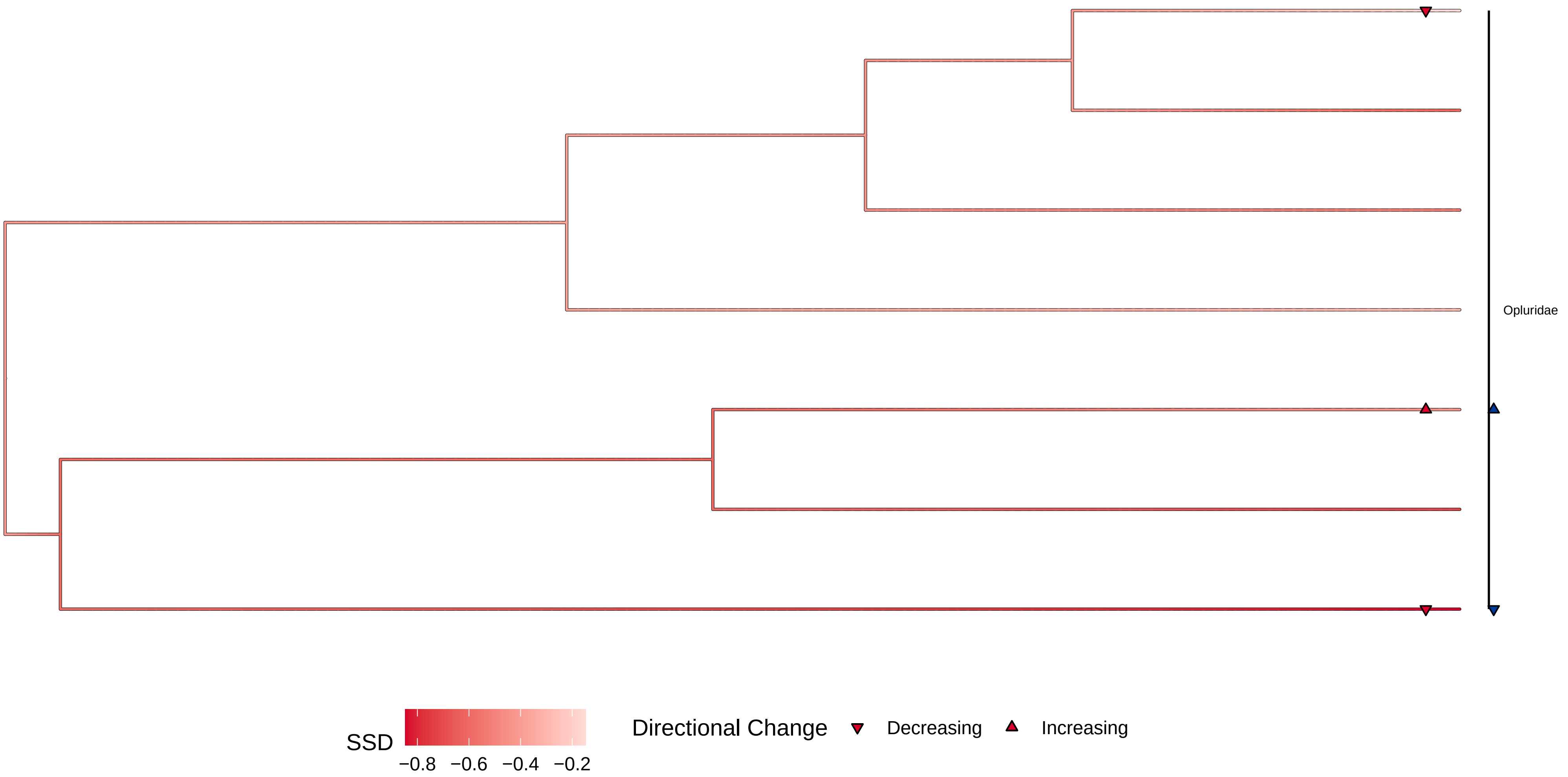

Squamates

Pareidae

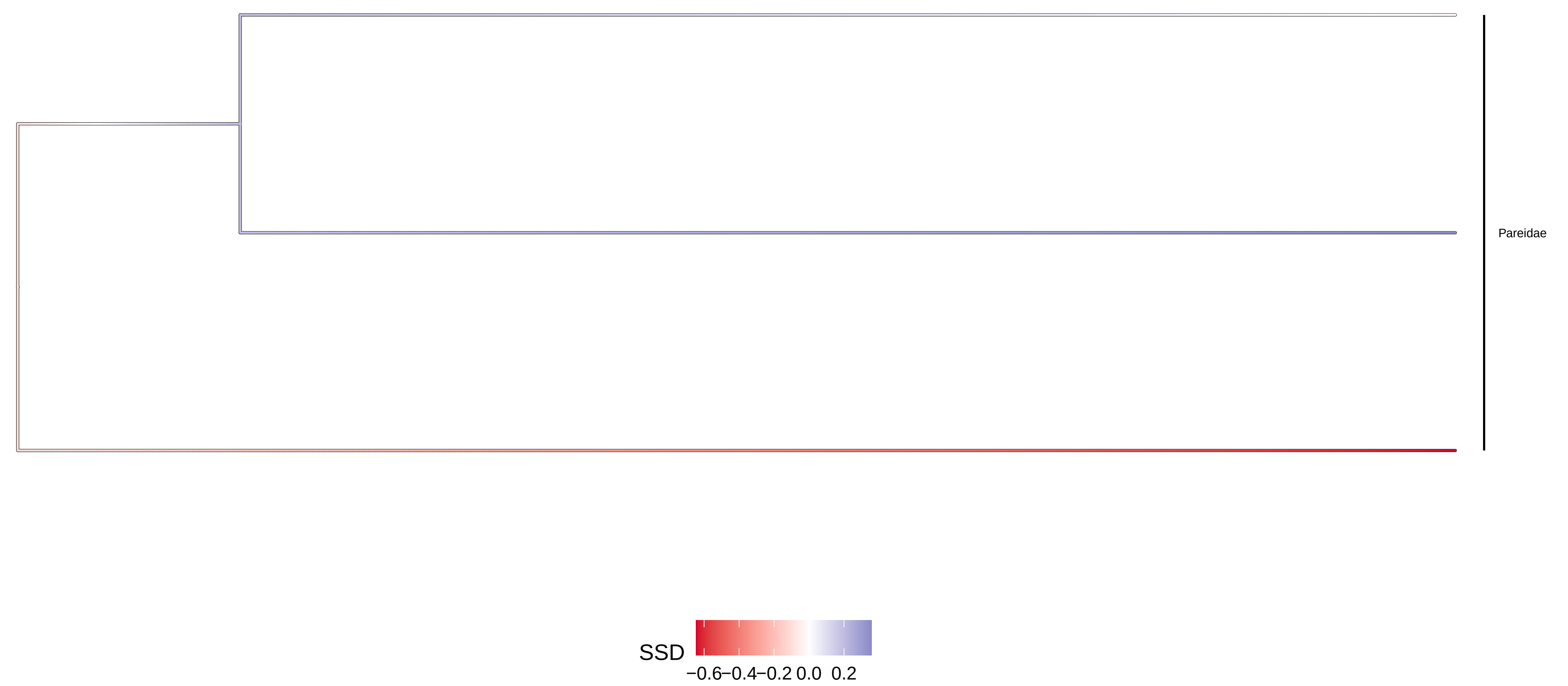

Squamates

Phrynosomatidae

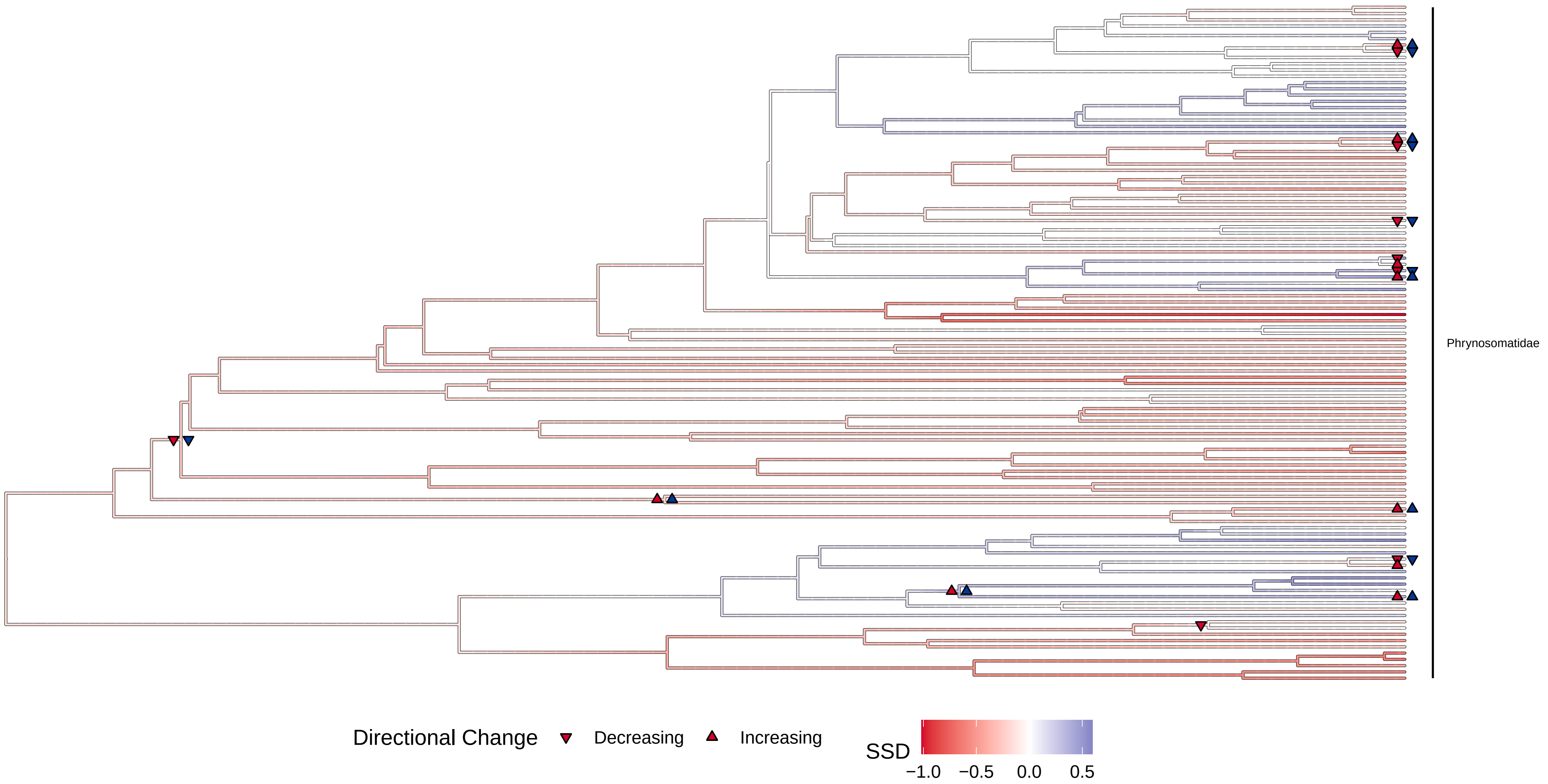

Squamates

Phyllodactylidae

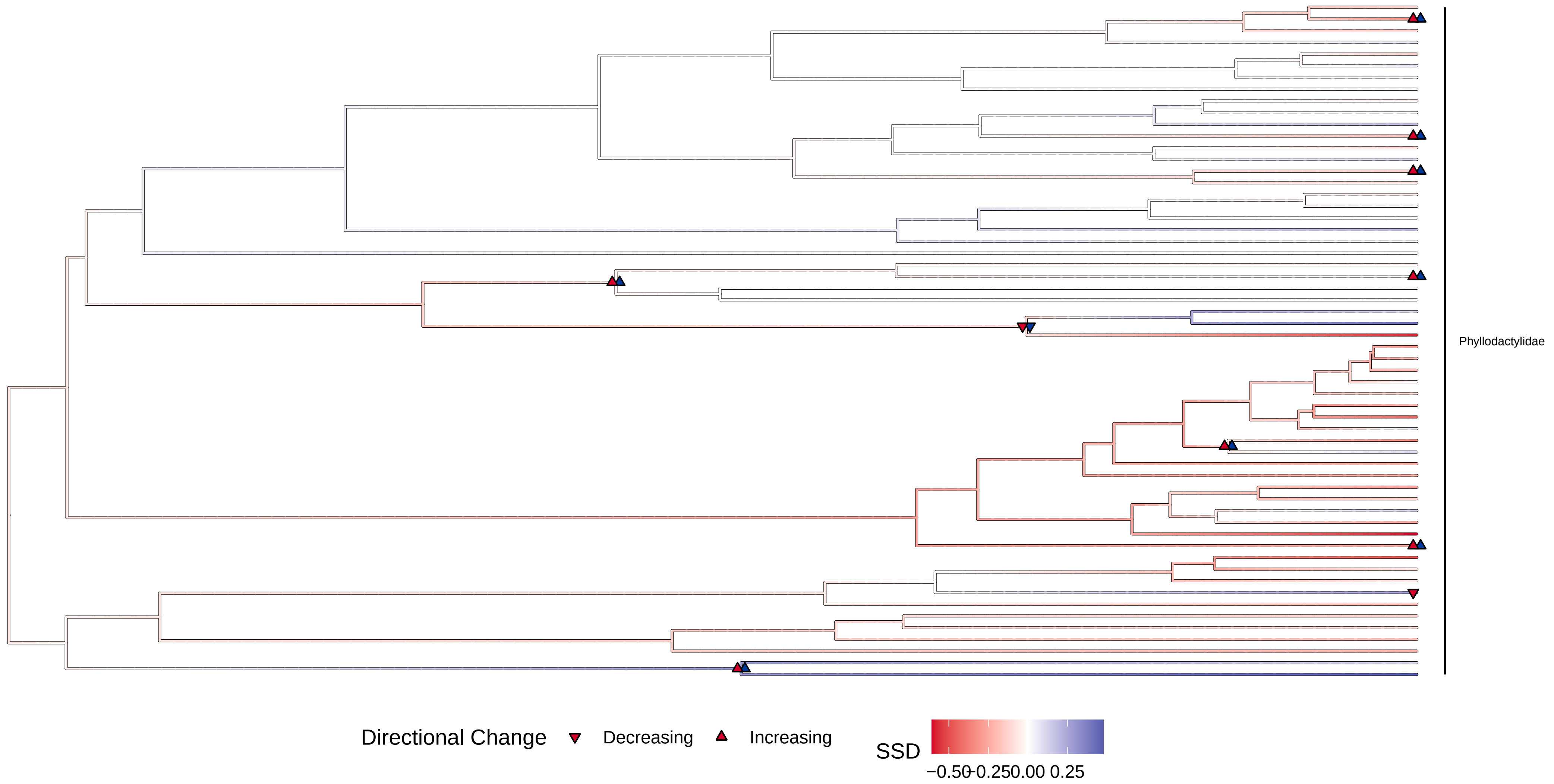

Squamates

Polychrotidae

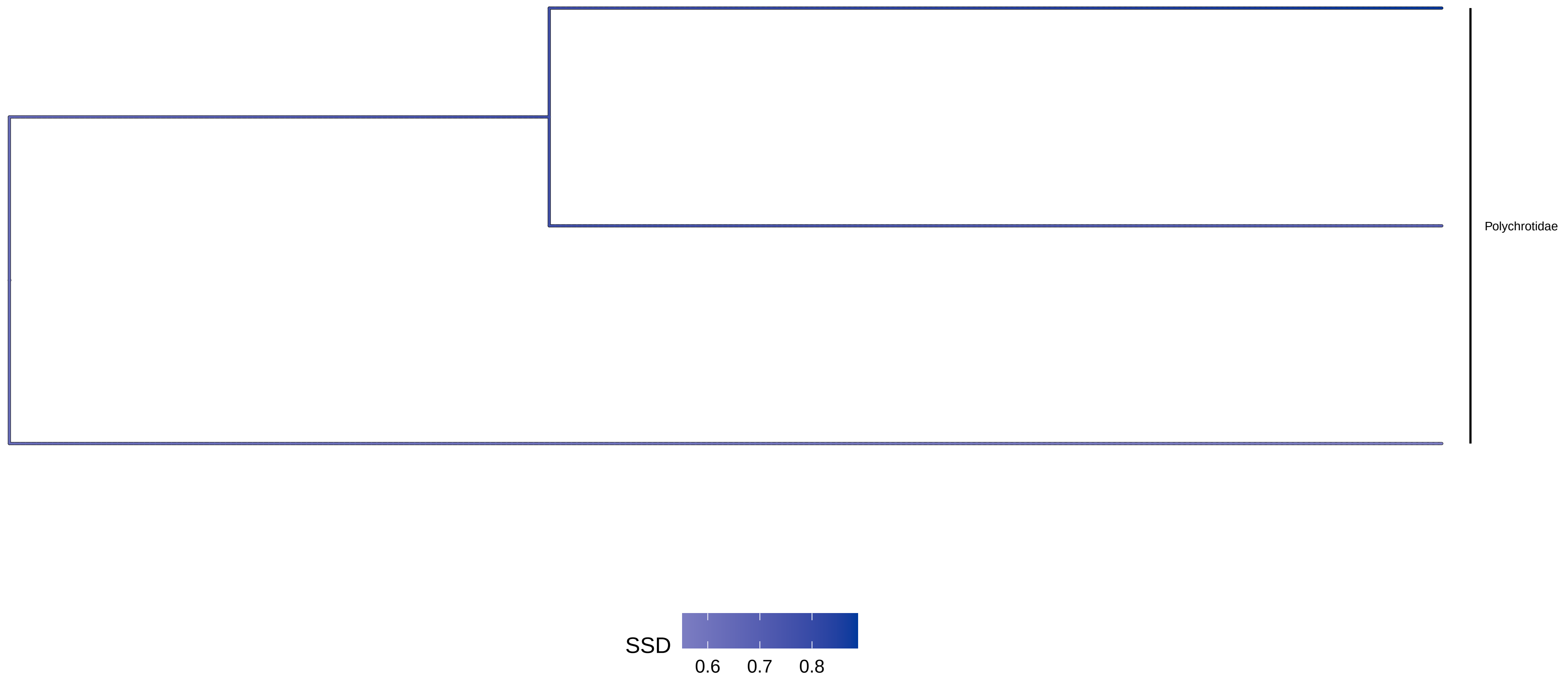

Squamates

Pygopodidae

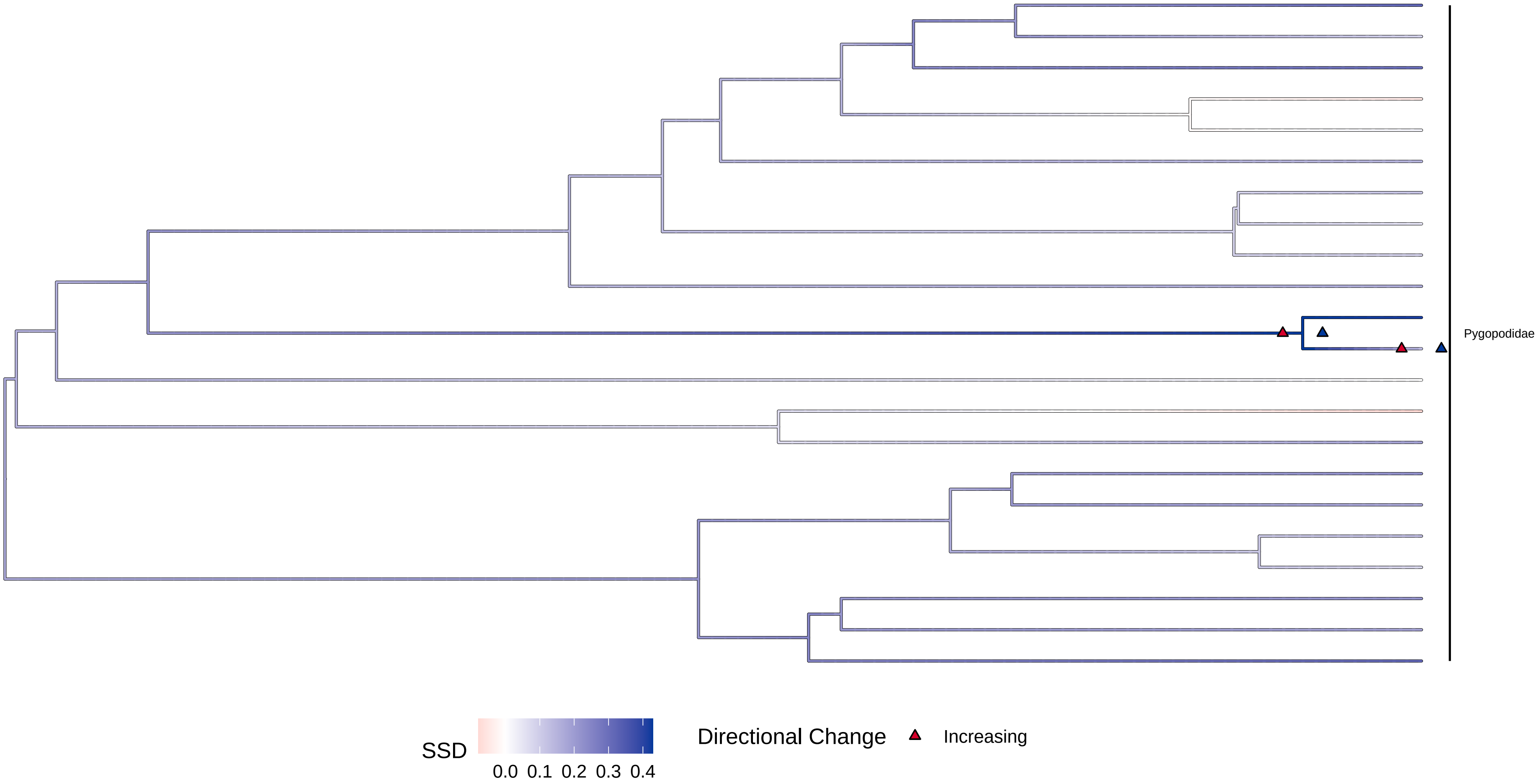

Squamates

Pythonoidea

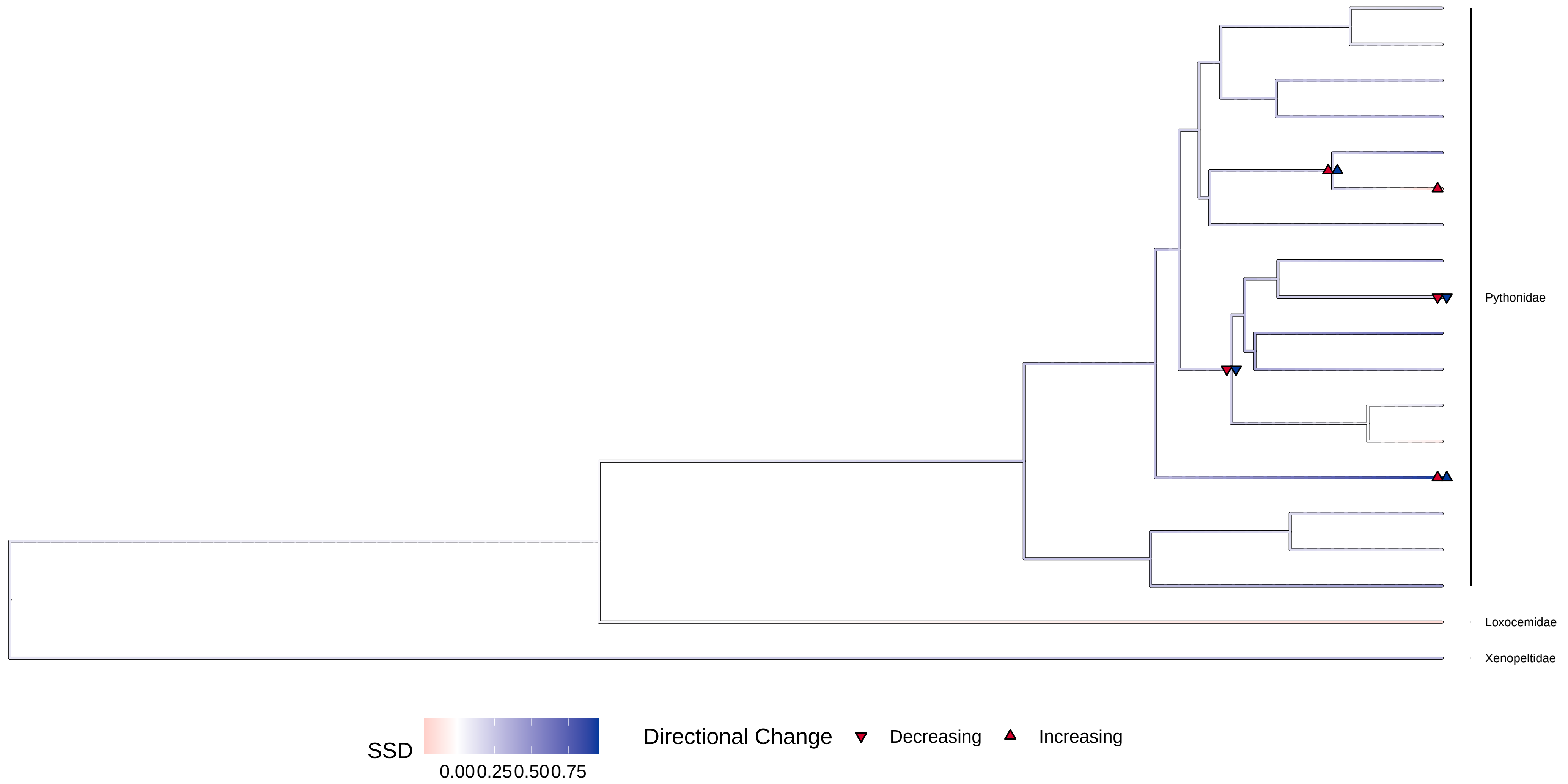

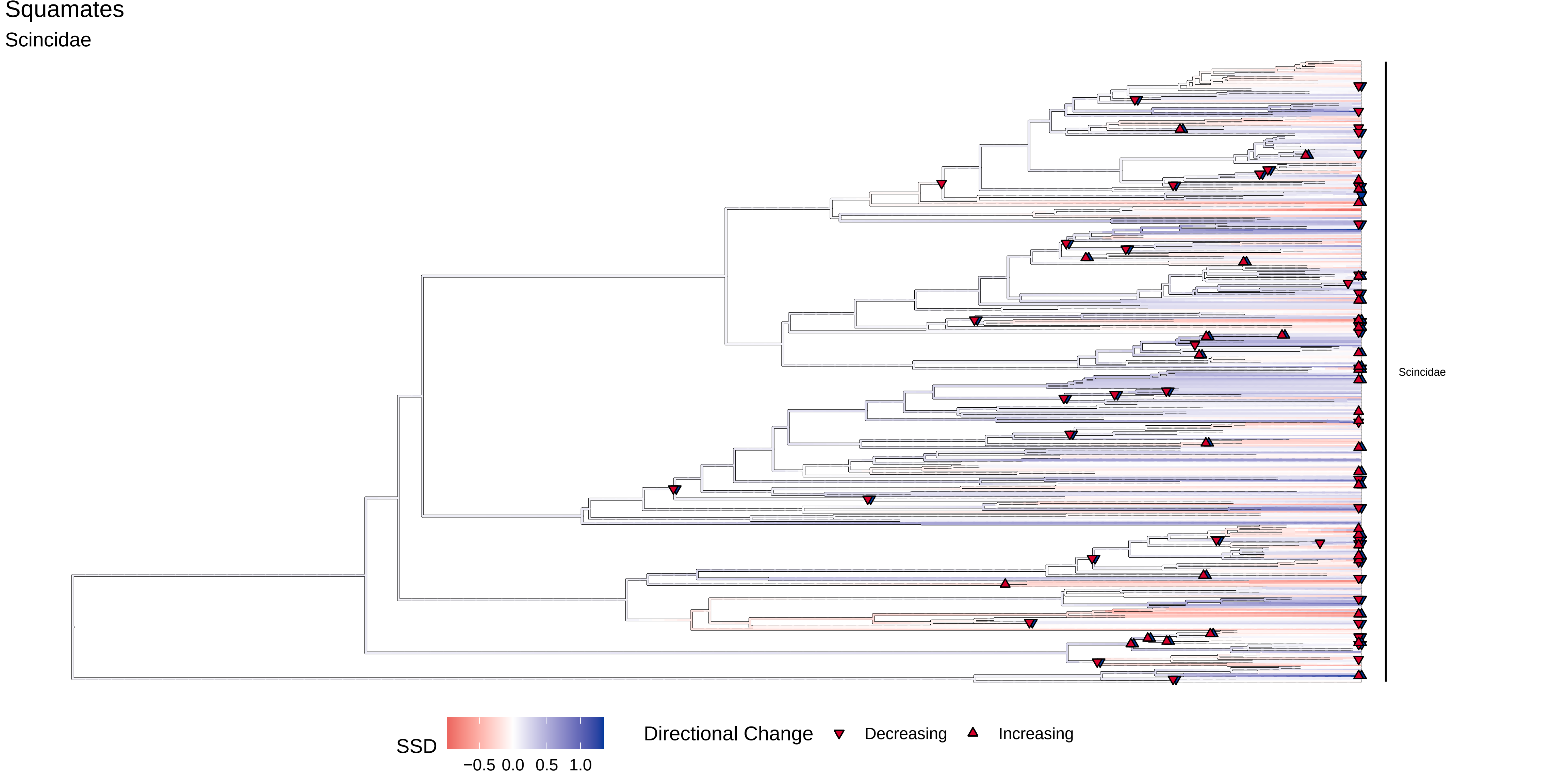

Squamates
Scincidae
Scincidae
SSD
−0.5 0.0 0.5 1.0
Directional Change
Decreasing
Increasing

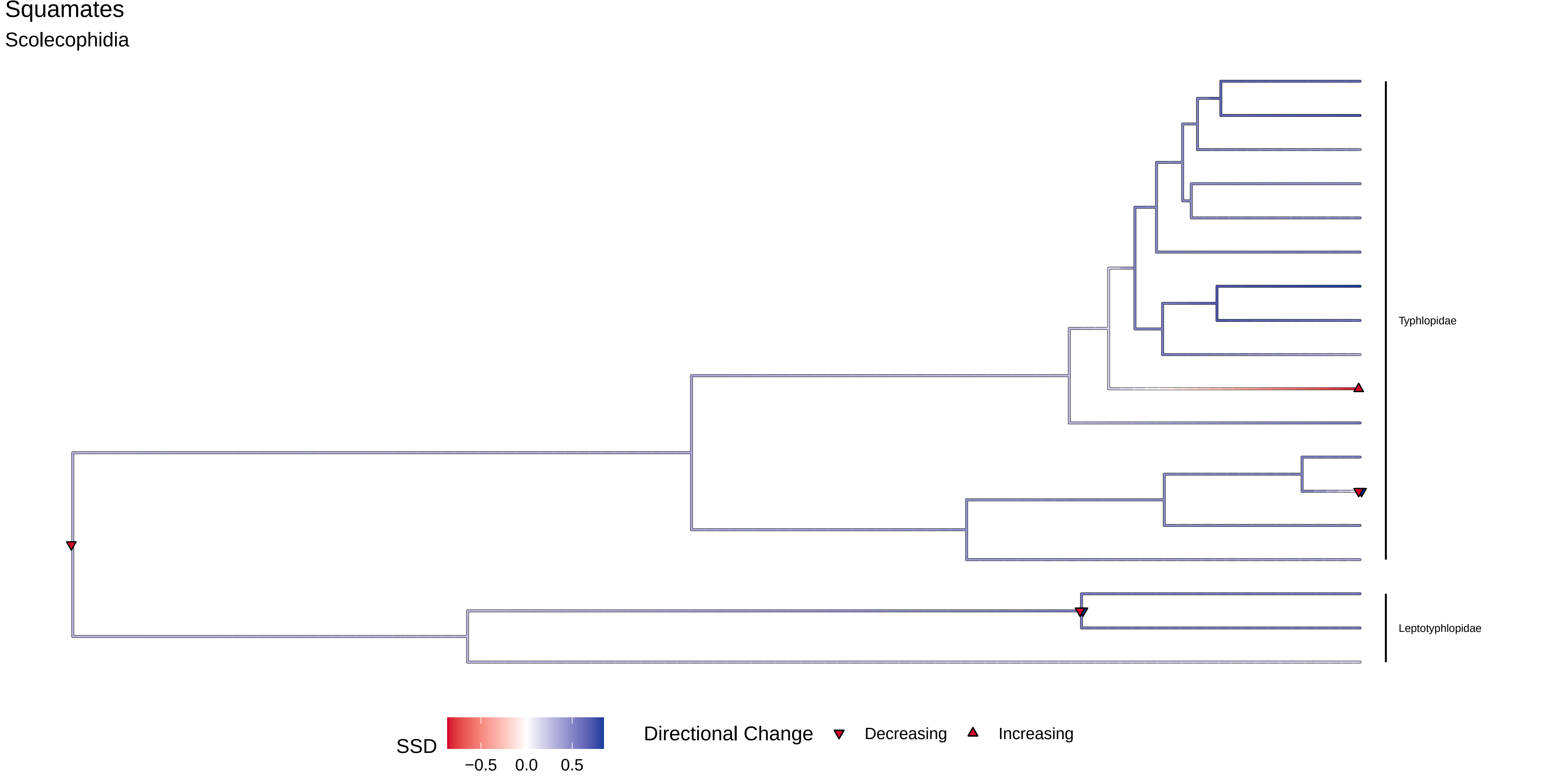
Squamates
Scolecophidia
Typhlopidae
Leptotyphlopidae
SSD
−0.5
0.0
0.5
Directional Change
Decreasing
Increasing

Squamates

Sphaerodactylidae

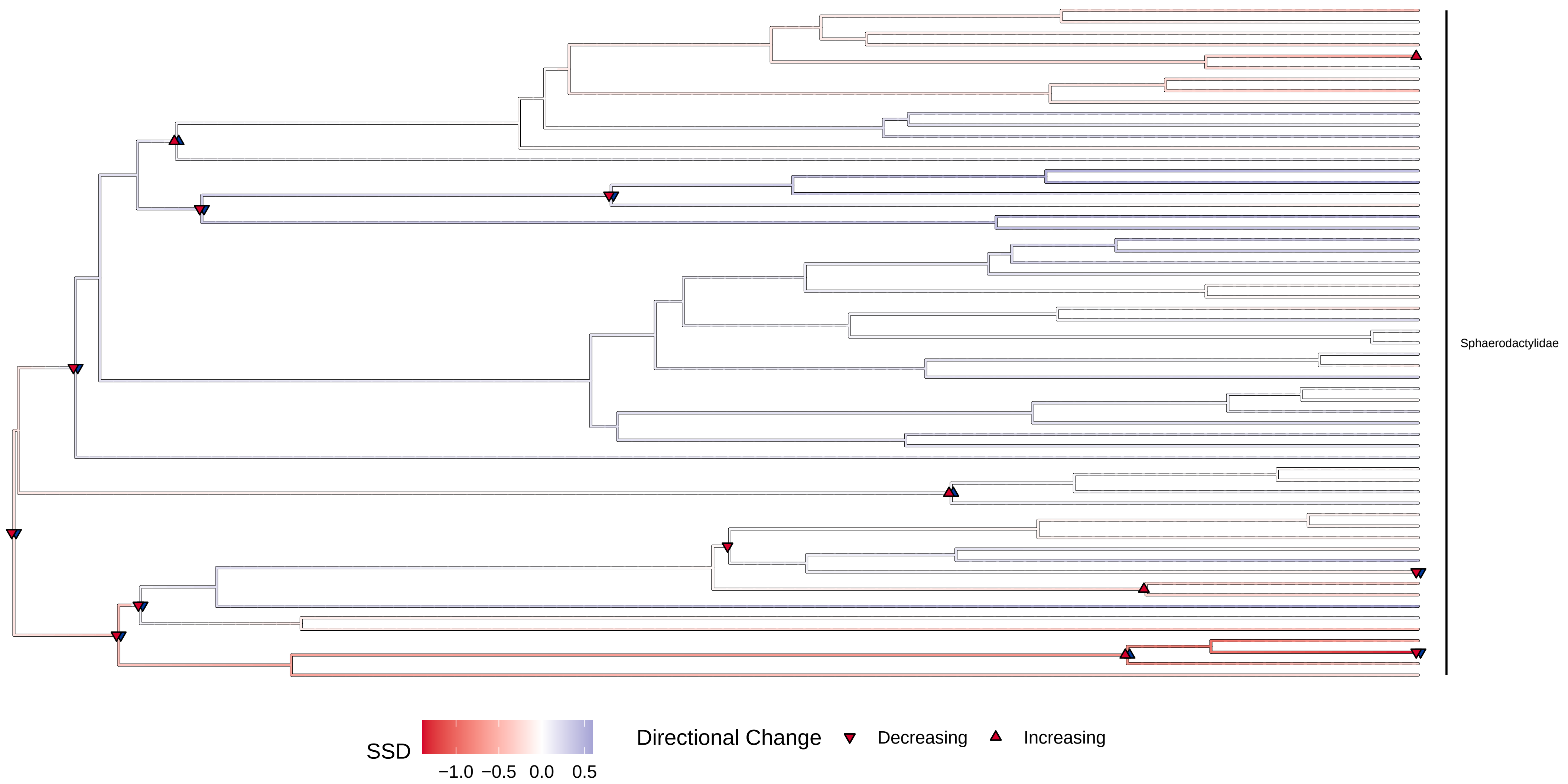

Squamates

Tropidophiidae

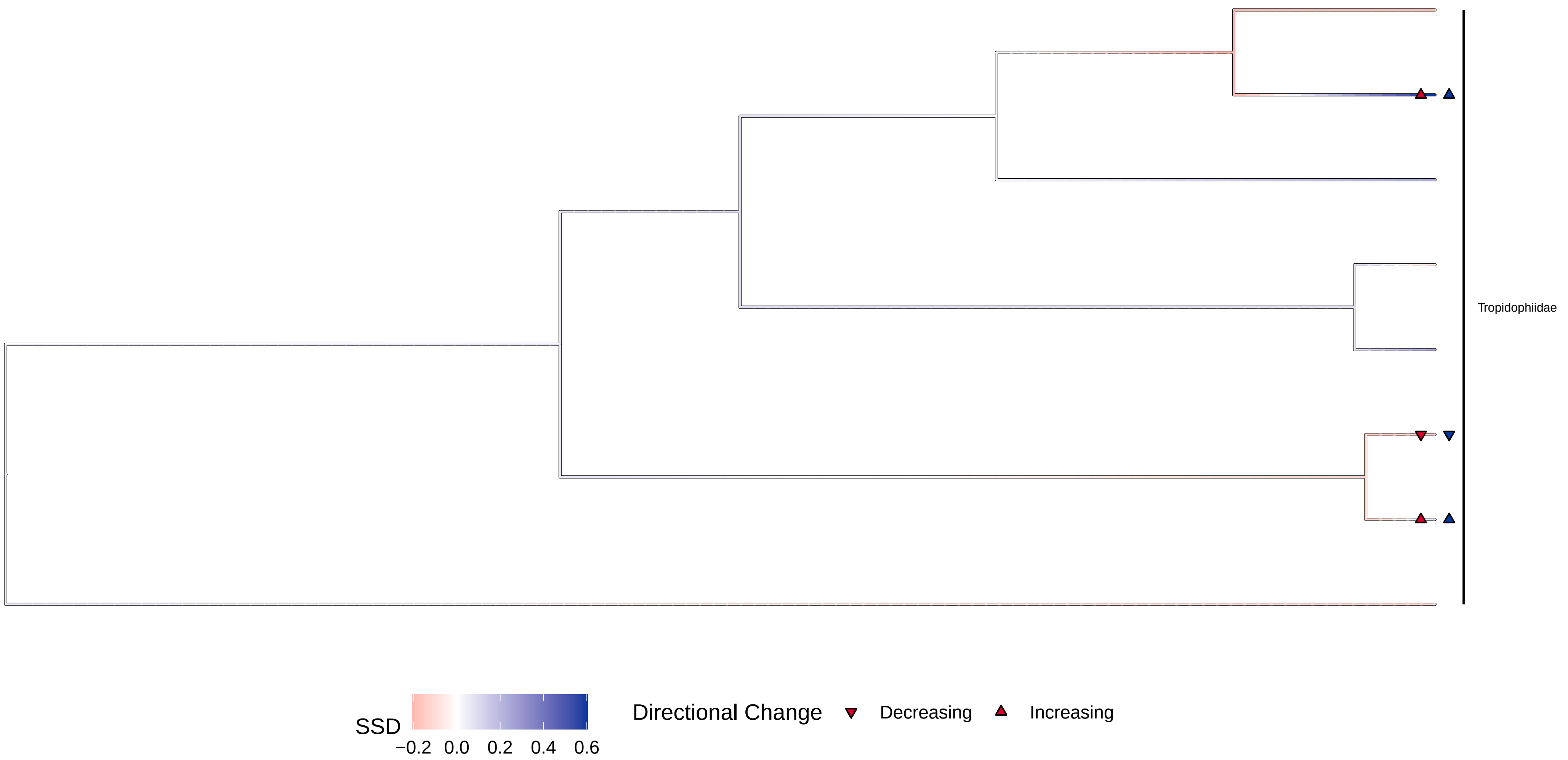

Squamates

Tropiduridae

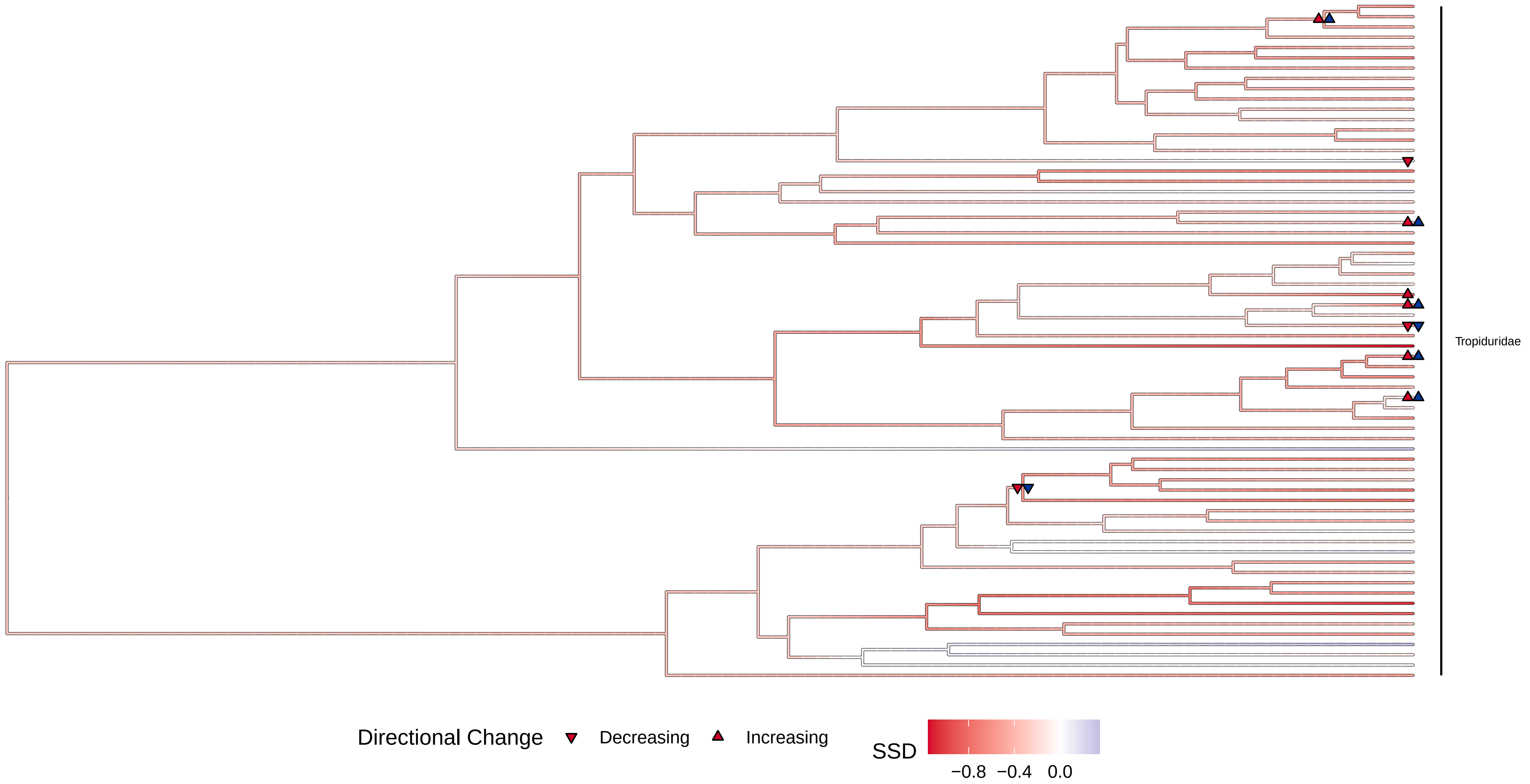

Squamates

Uropeltoidea

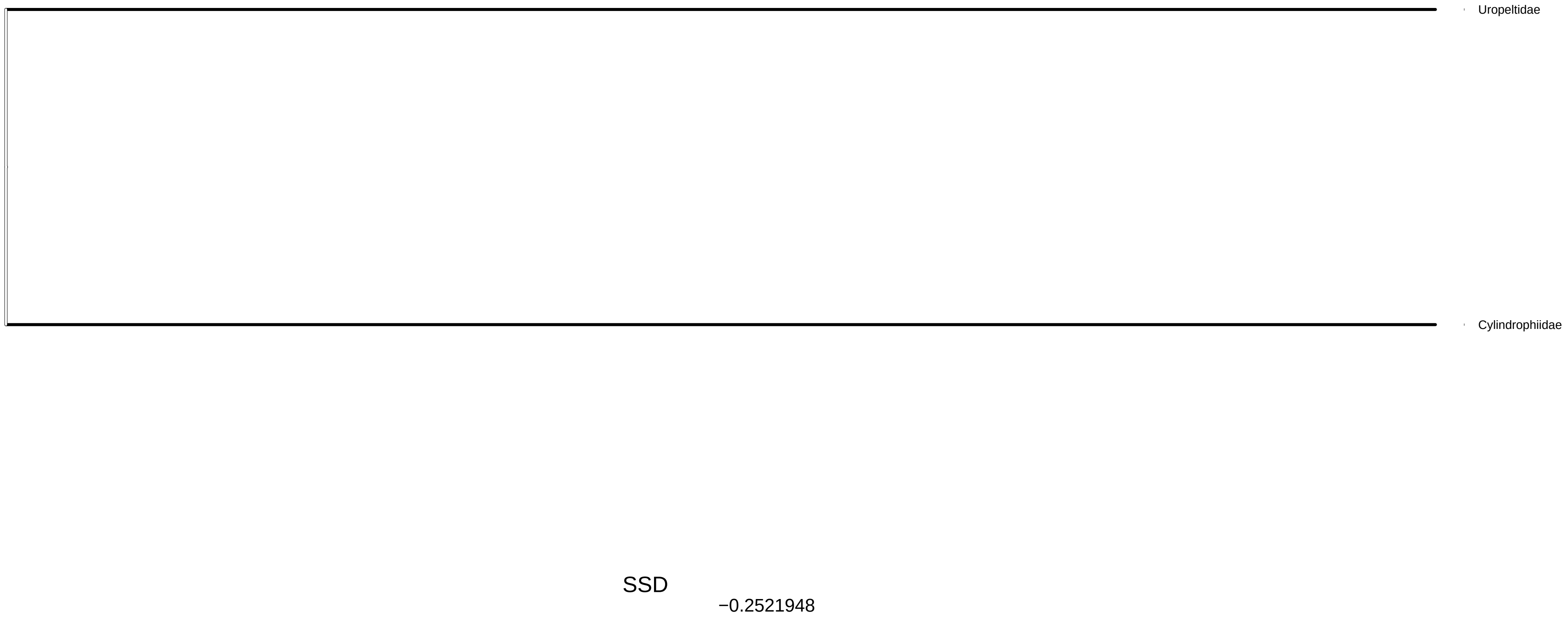

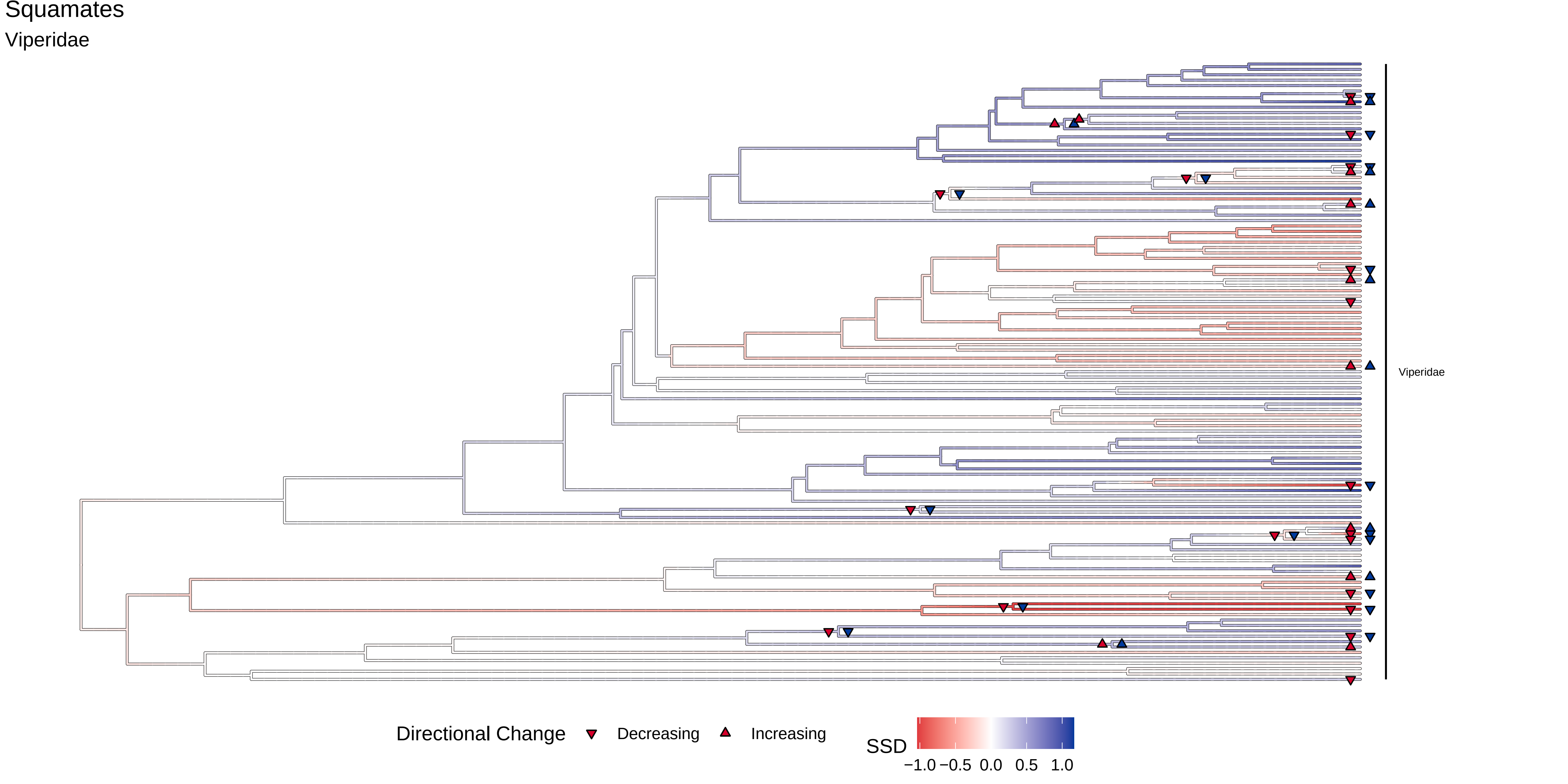

Squamates
Viperidae
Viperidae
Directional Change
Decreasing
Increasing
SSD
−1.0 −0.5 0.0 0.5 1.0

Squamates

Xantusiidae

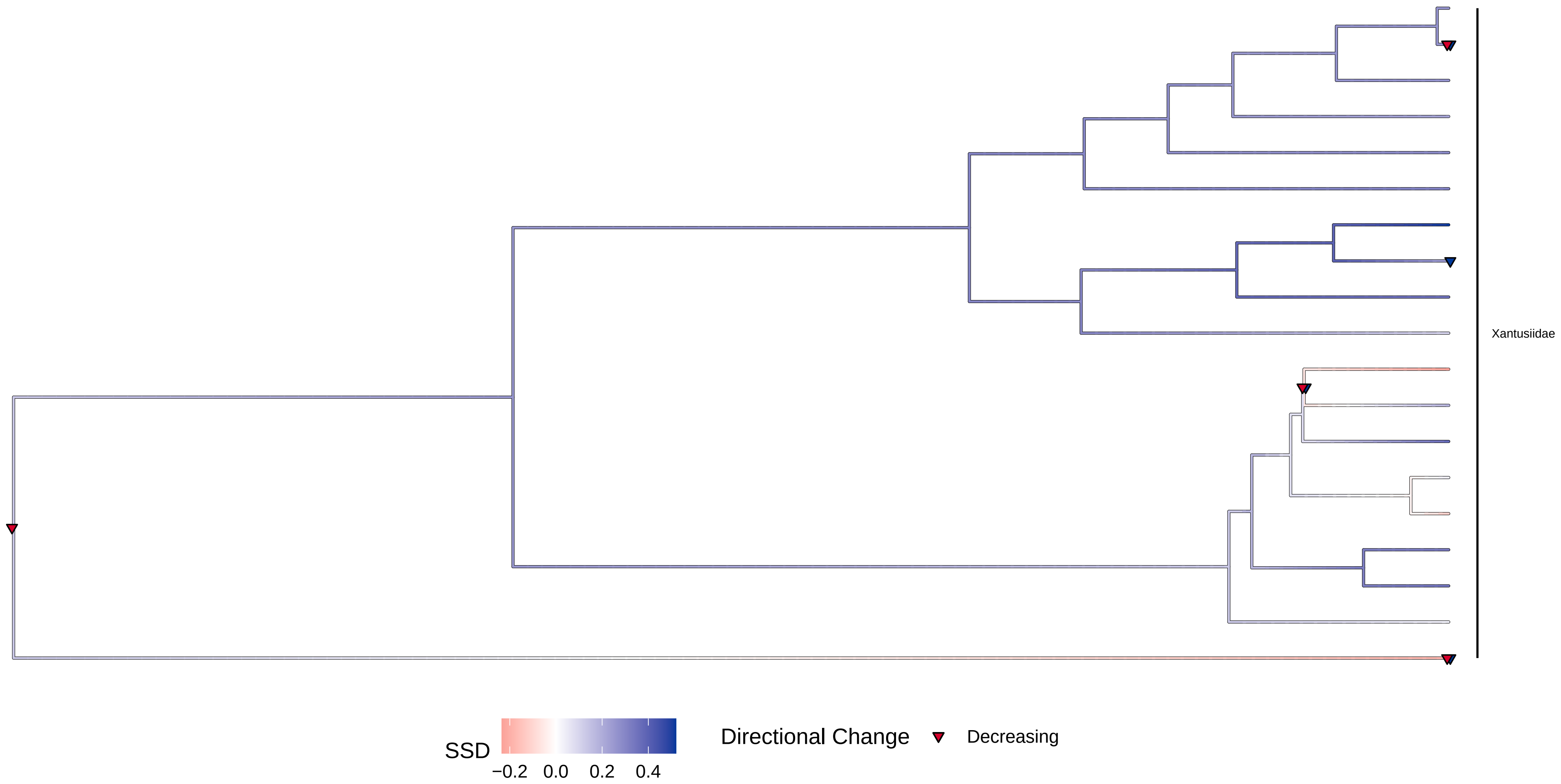

Squamates

Xenodermidae

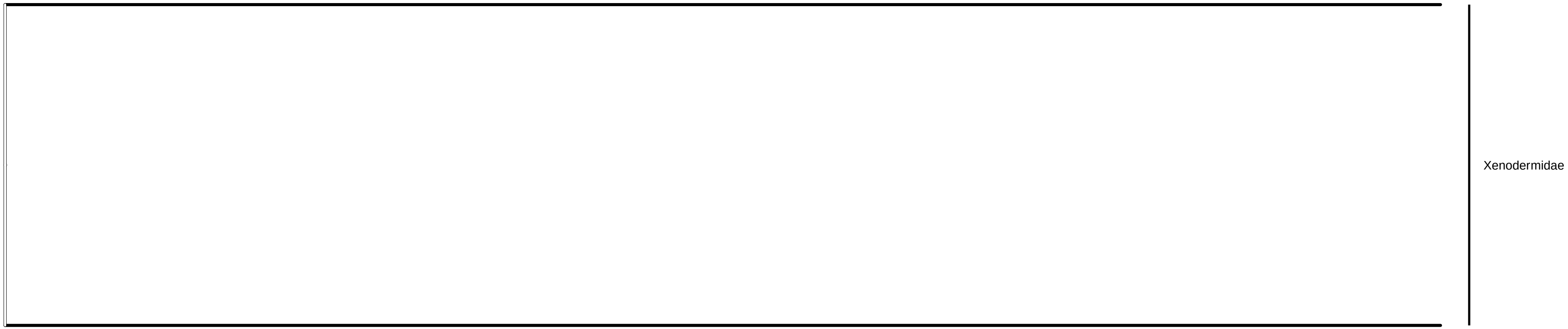

SSD

0.02471352

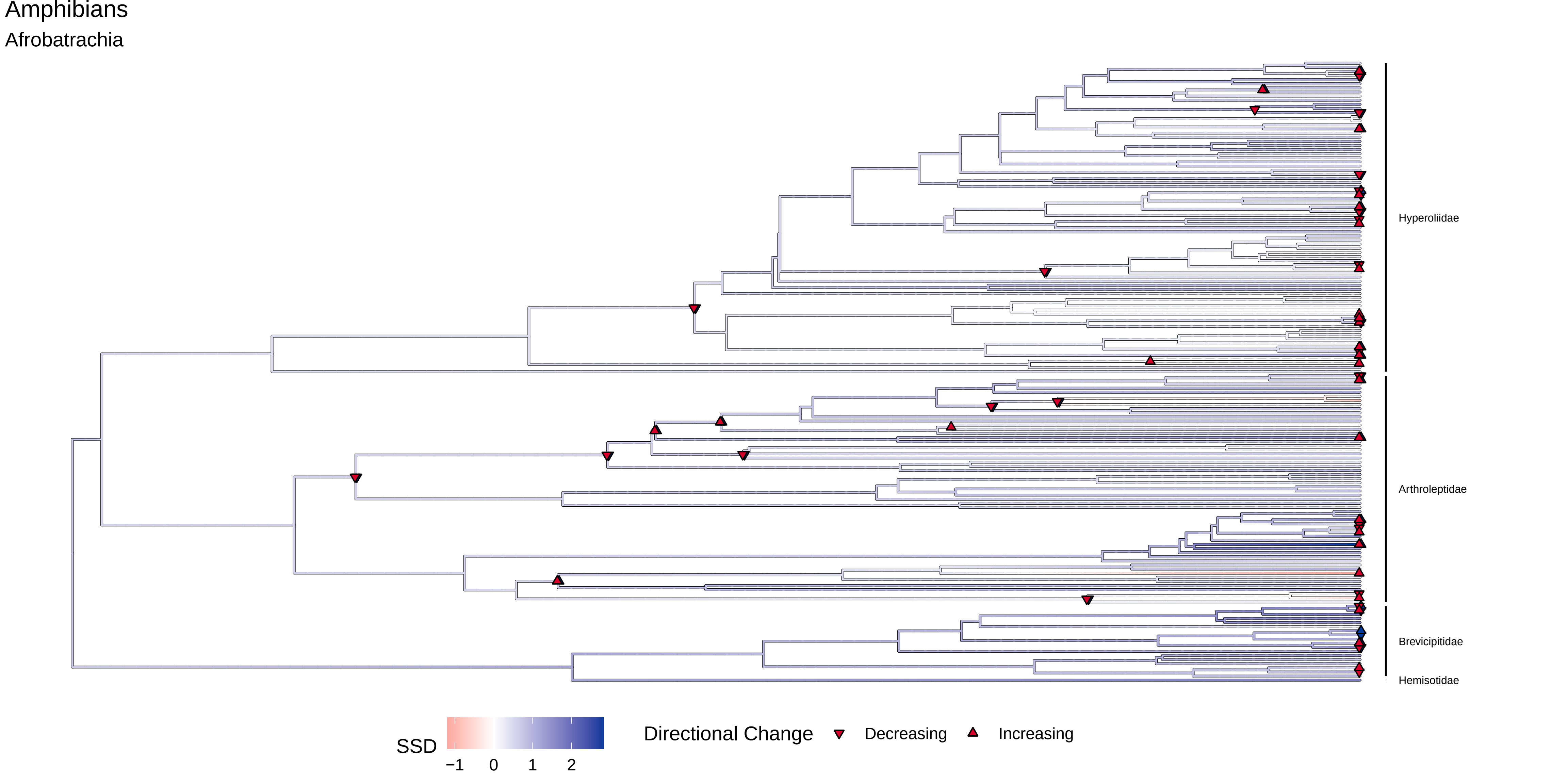

Amphibians
Afrobatrachia
Hyperoliidae
Arthroleptidae
Brevicipitidae
Hemisotidae
SSD
−1
0
1
2
Directional Change
Decreasing
Increasing

Amphibians

Alsodidae

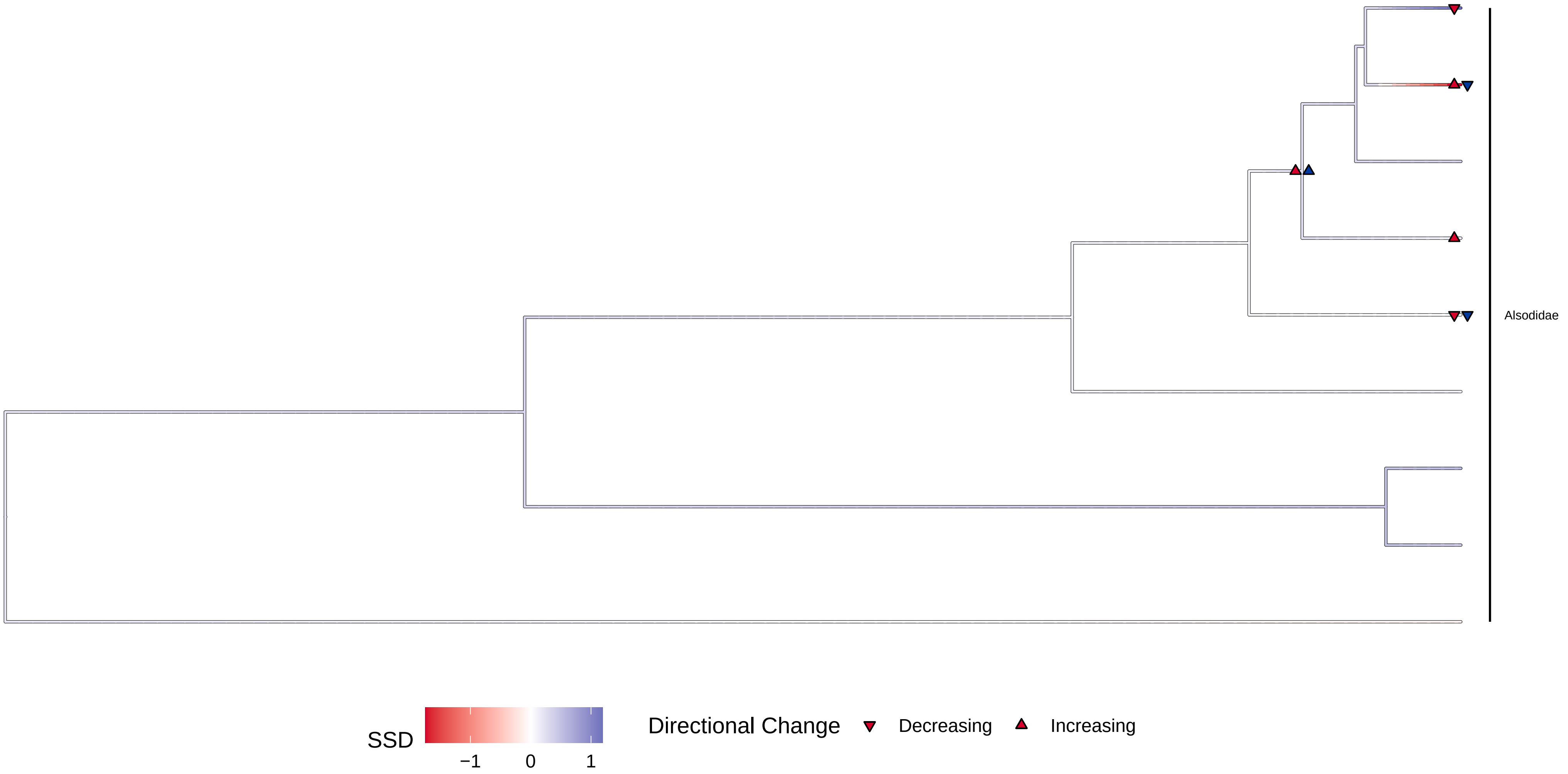

Amphibians

Ambystomatidae

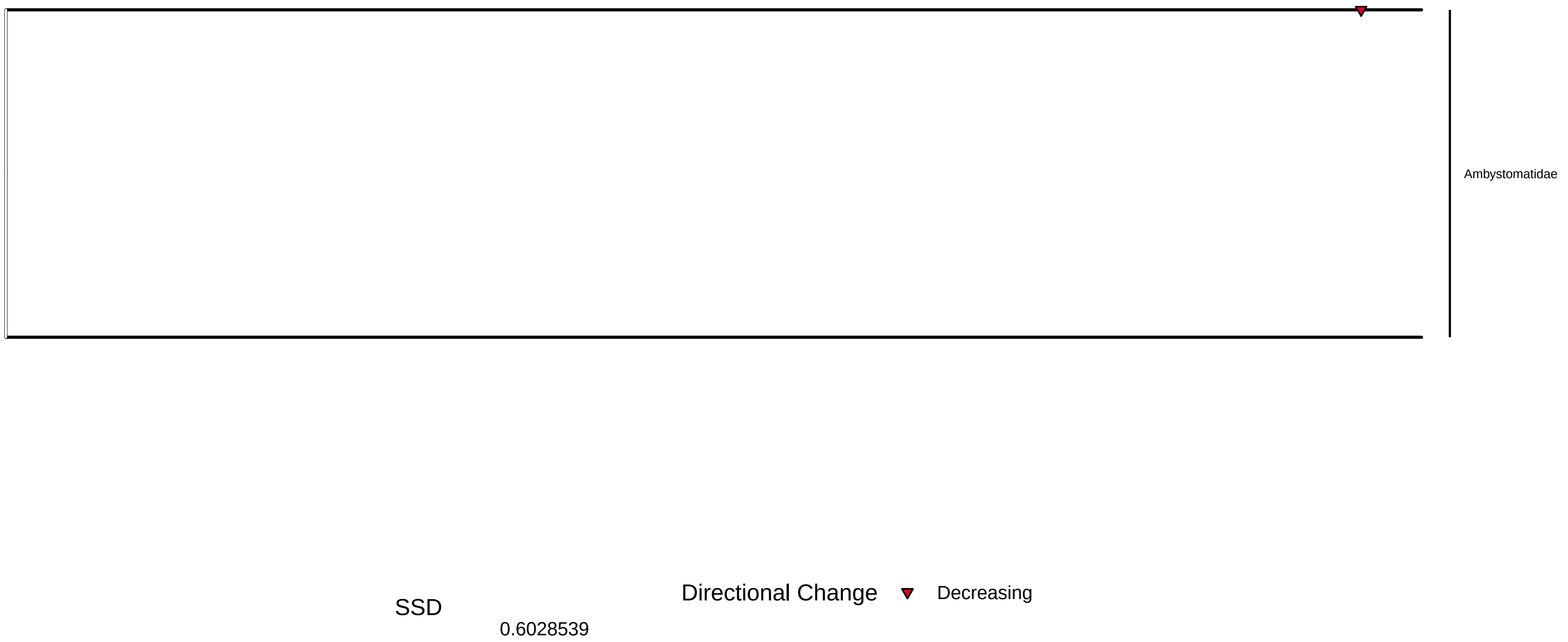

Amphibians

Batrachylidae

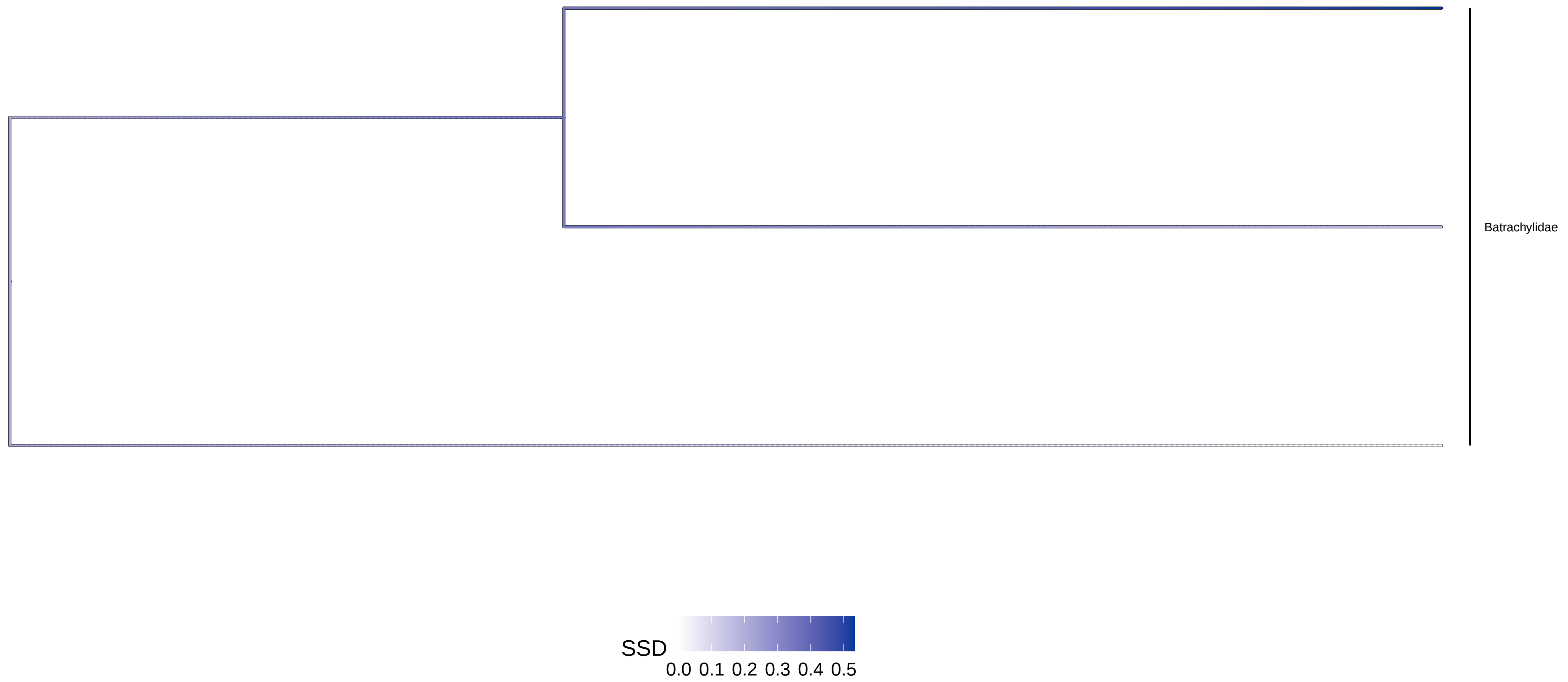

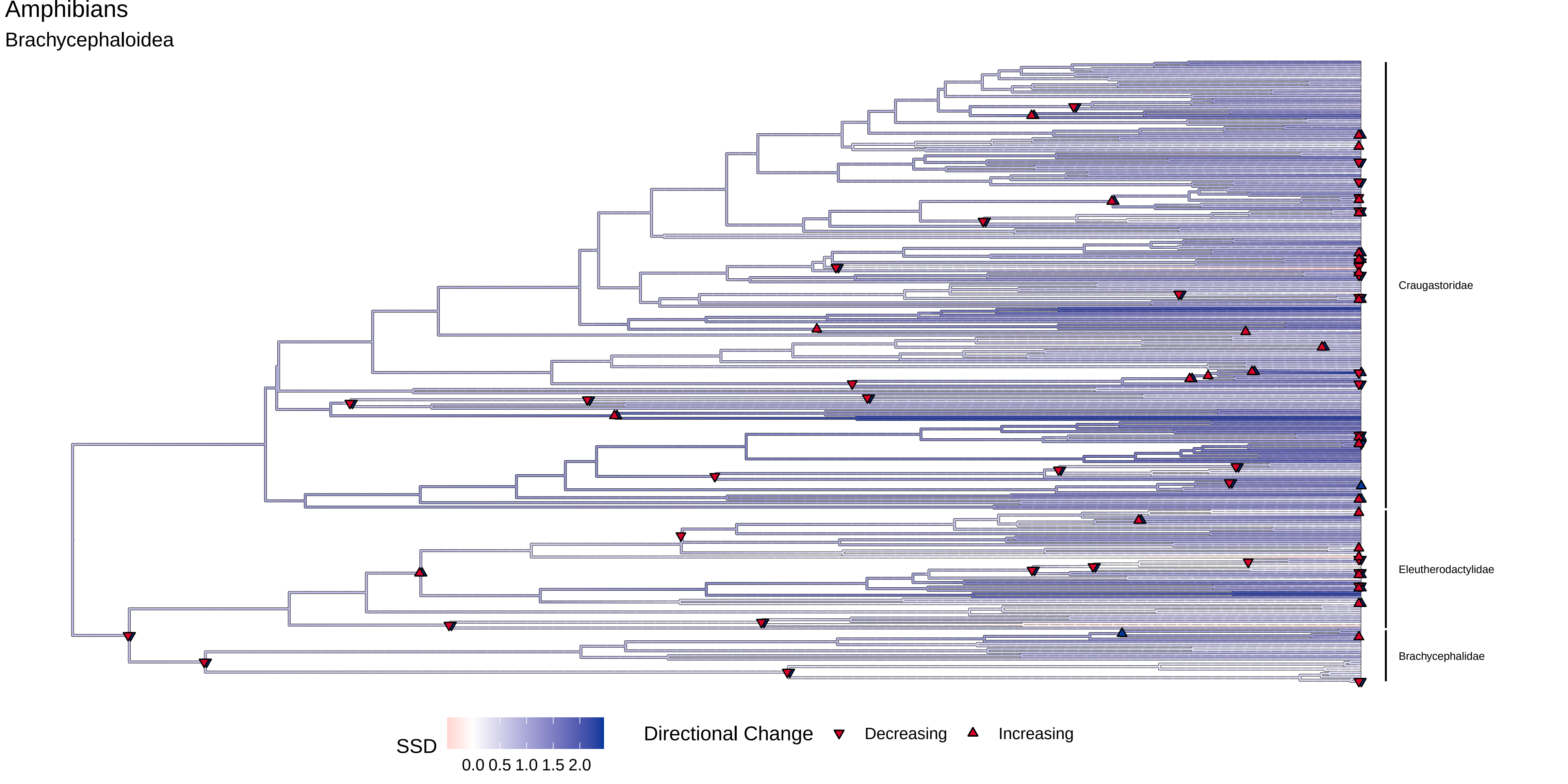

Amphibians
Brachycephaloidea
Craugastoridae
Eleutherodactylidae
Brachycephalidae
SSD
0.0 0.5 1.0 1.5 2.0
Directional Change
Decreasing
Increasing

Amphibians

Bufonidae

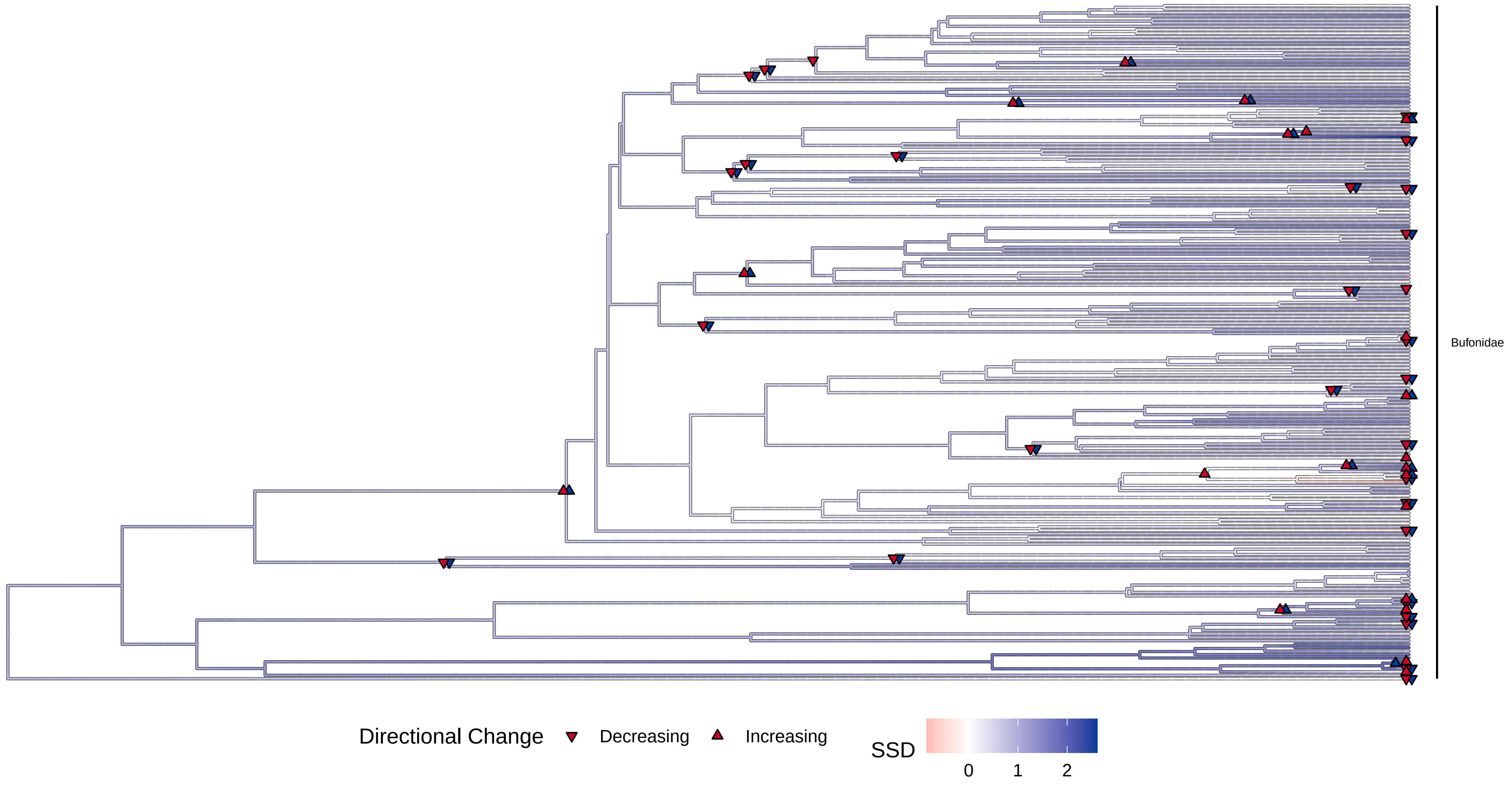

Amphibians

Centrolenidae

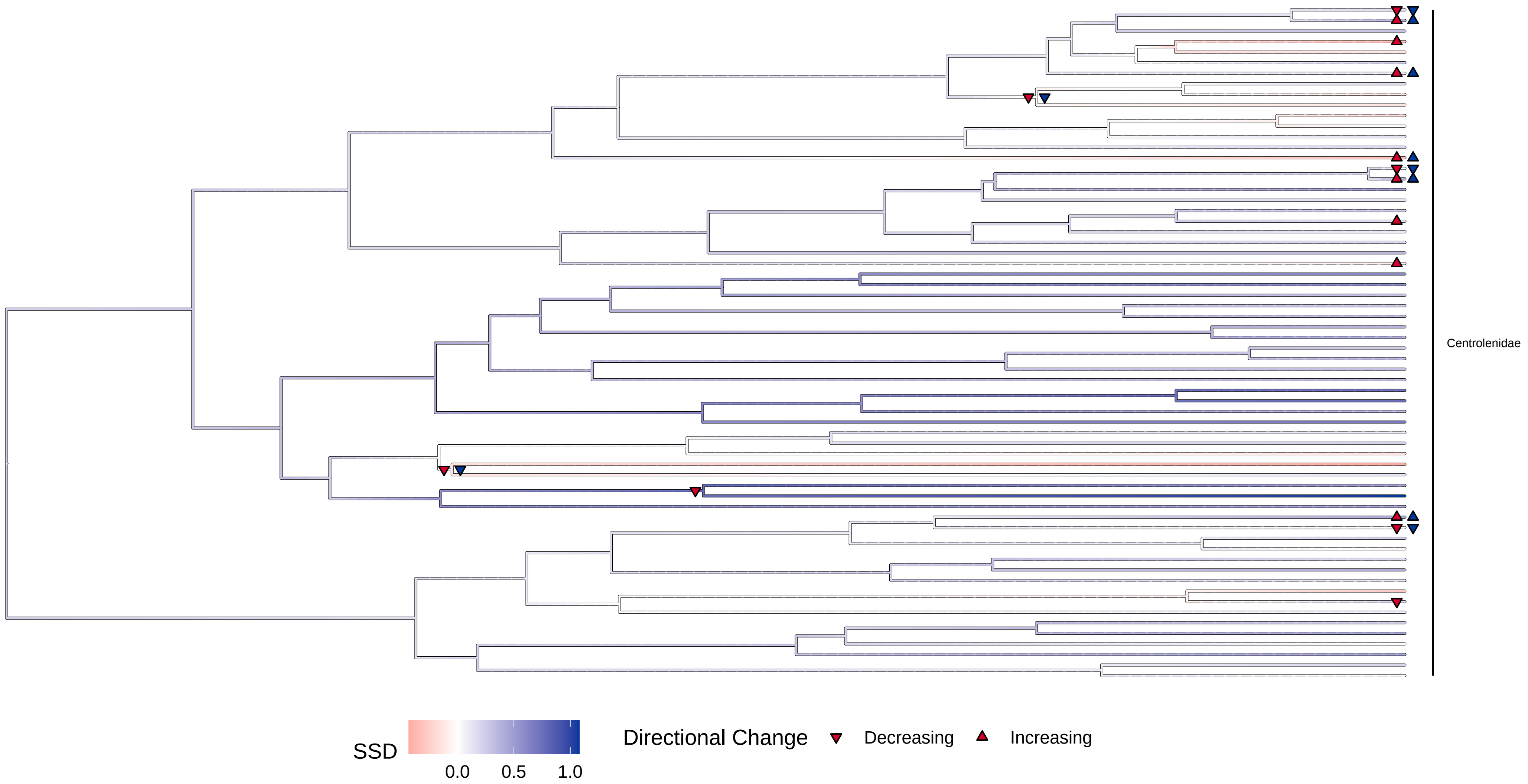

Amphibians

Ceratobatrachidae

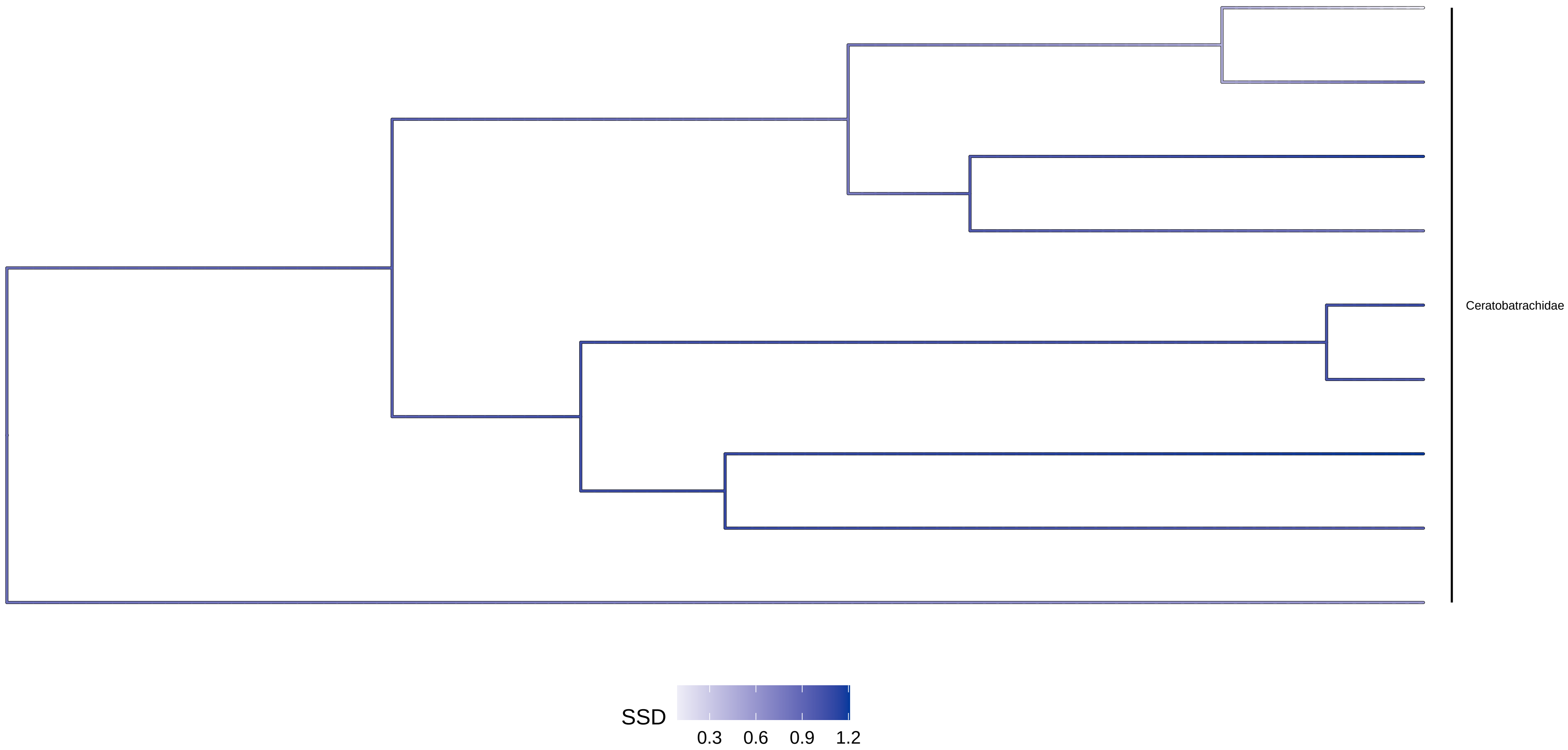

Amphibians

Ceratophryidae

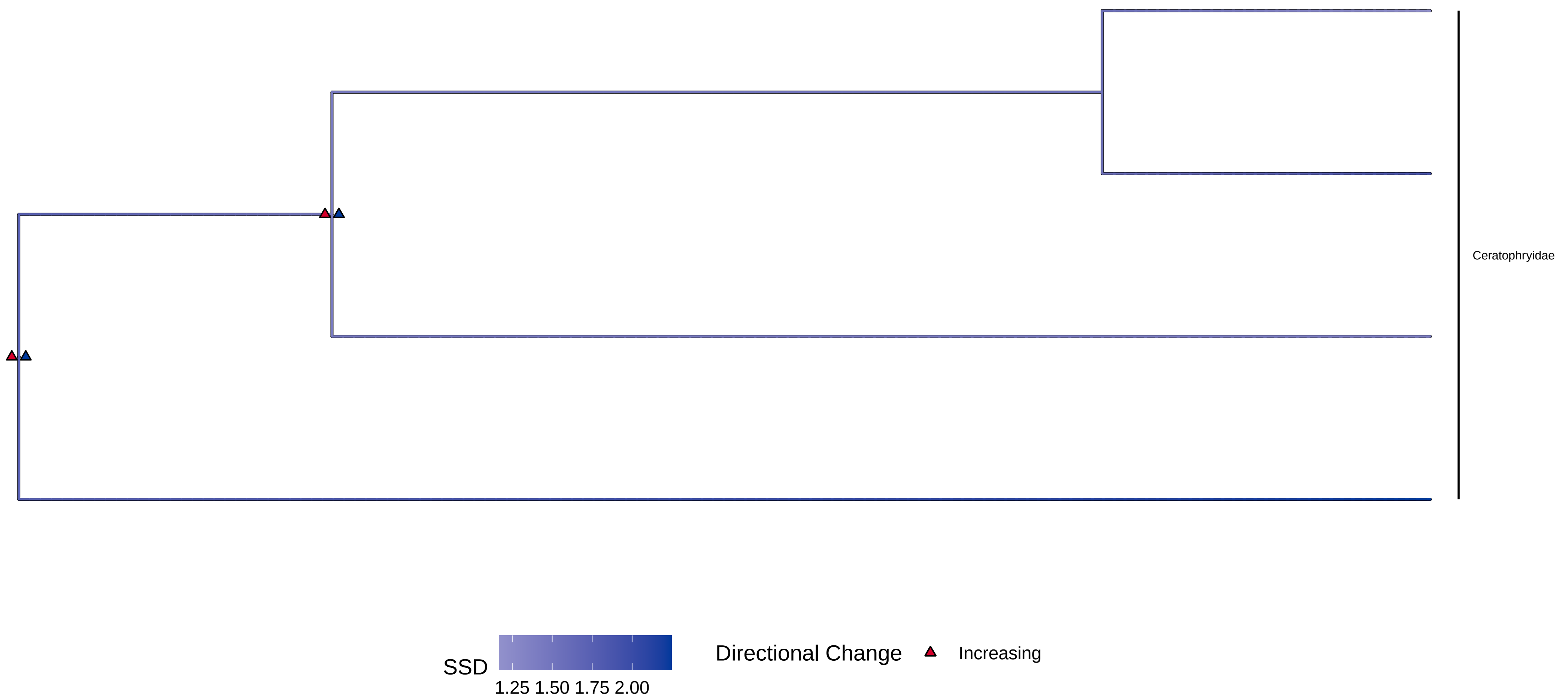

Amphibians

Conrauidae

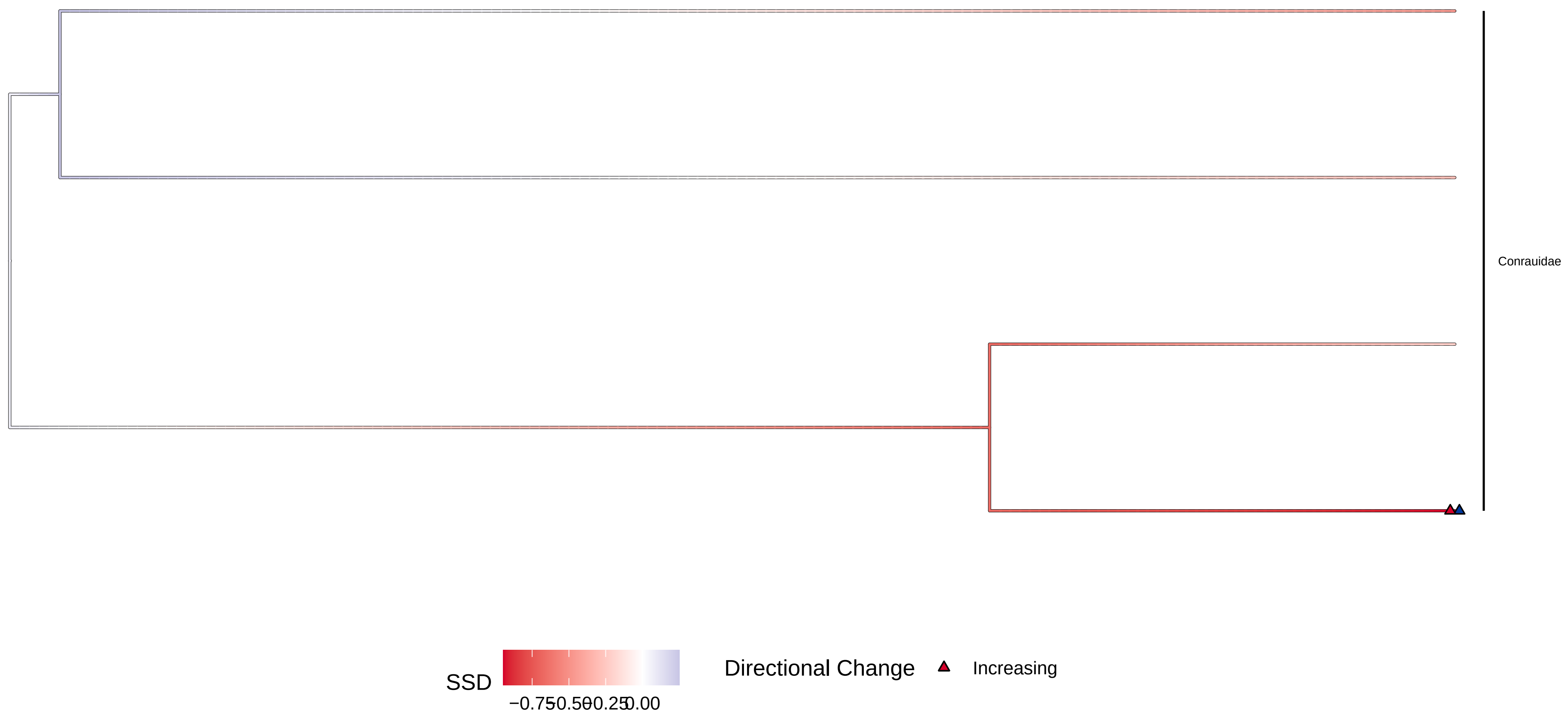

Amphibians

Cryptobranchoidea

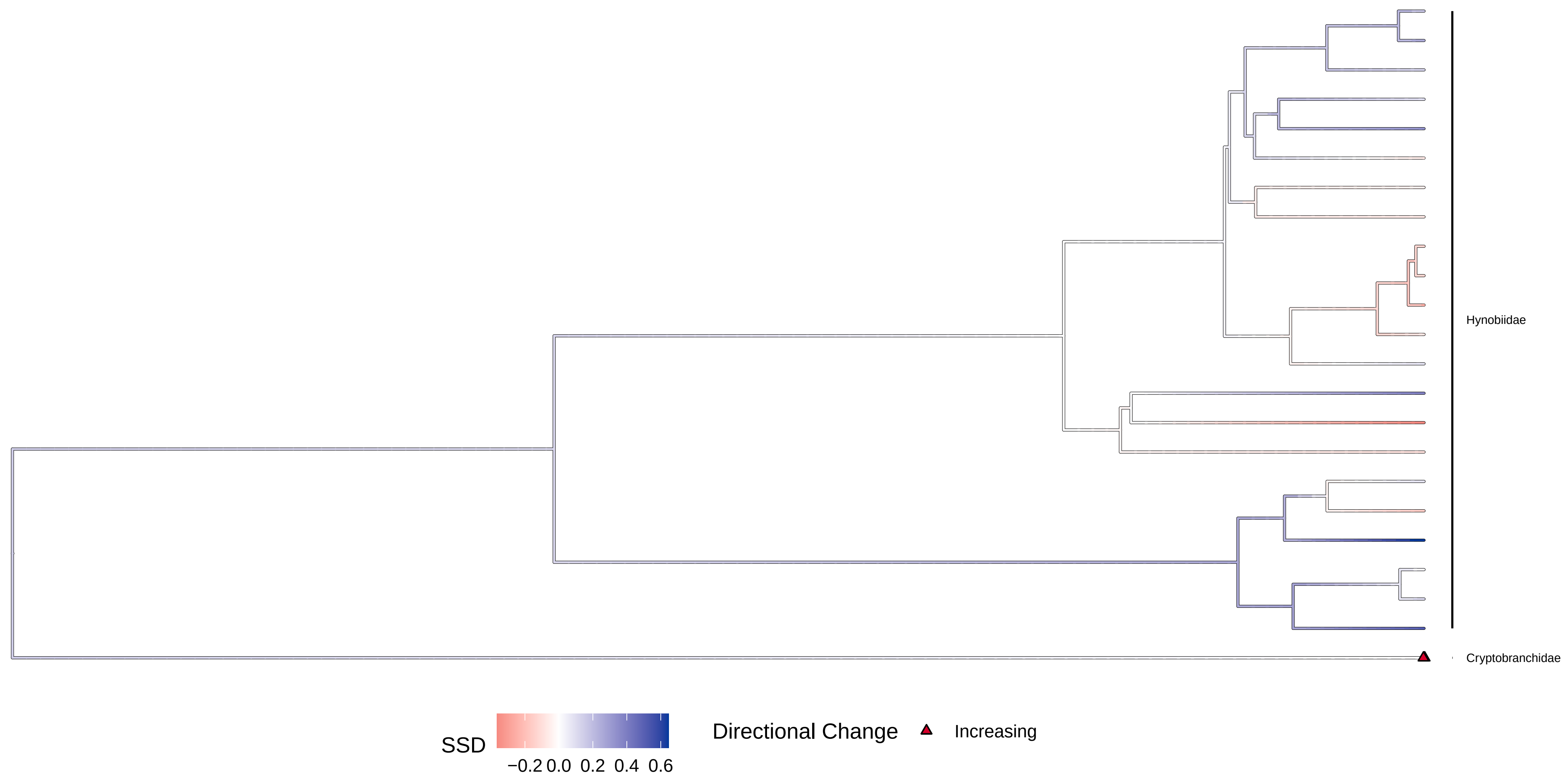

Amphibians

Cycloramphidae

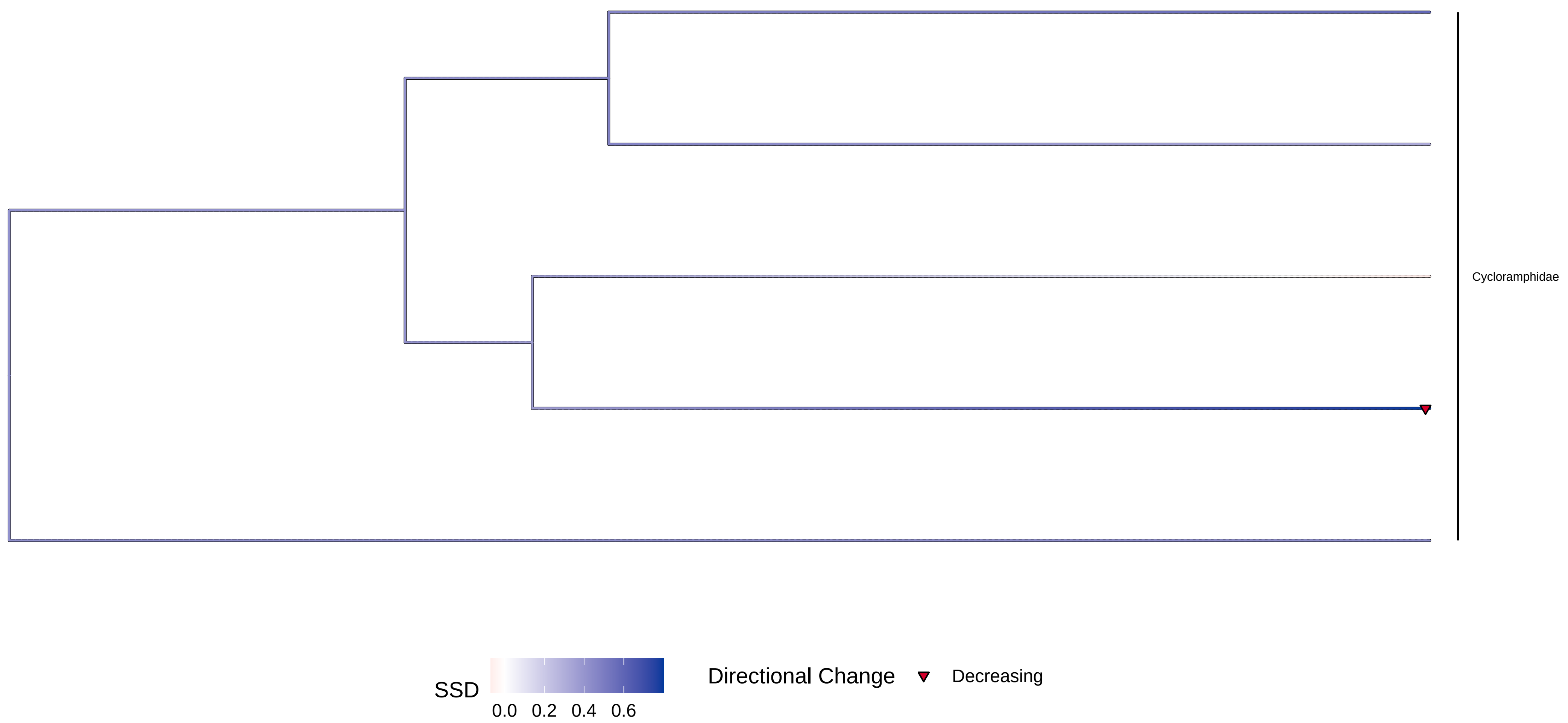

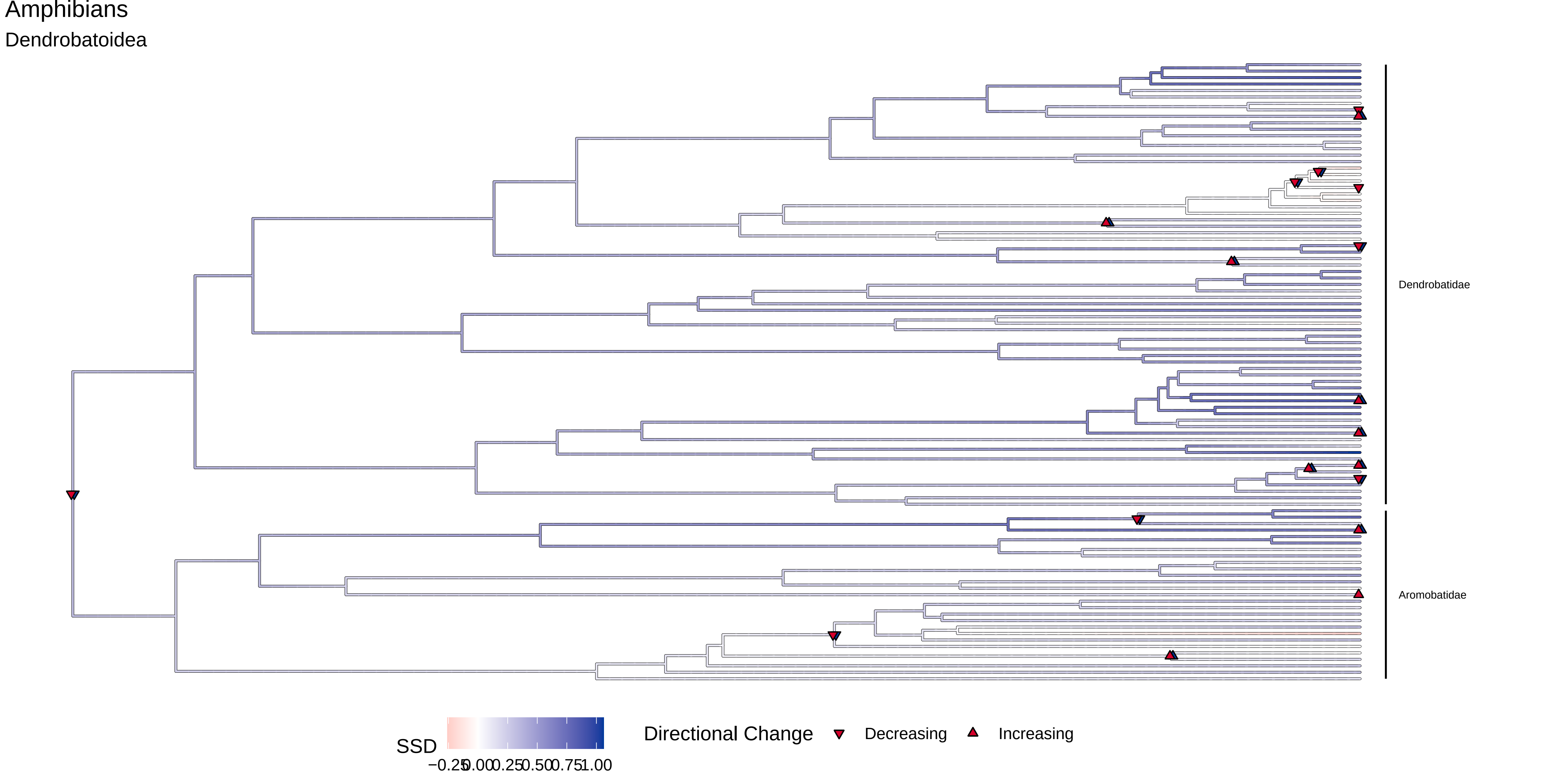
Amphibians
Dendrobatoidea
Dendrobatidae
Aromobatidae
SSD
−0.25
0.00
0.25
0.50
0.75
1.00
Directional Change
Decreasing
Increasing

Amphibians

Dicroglossidae

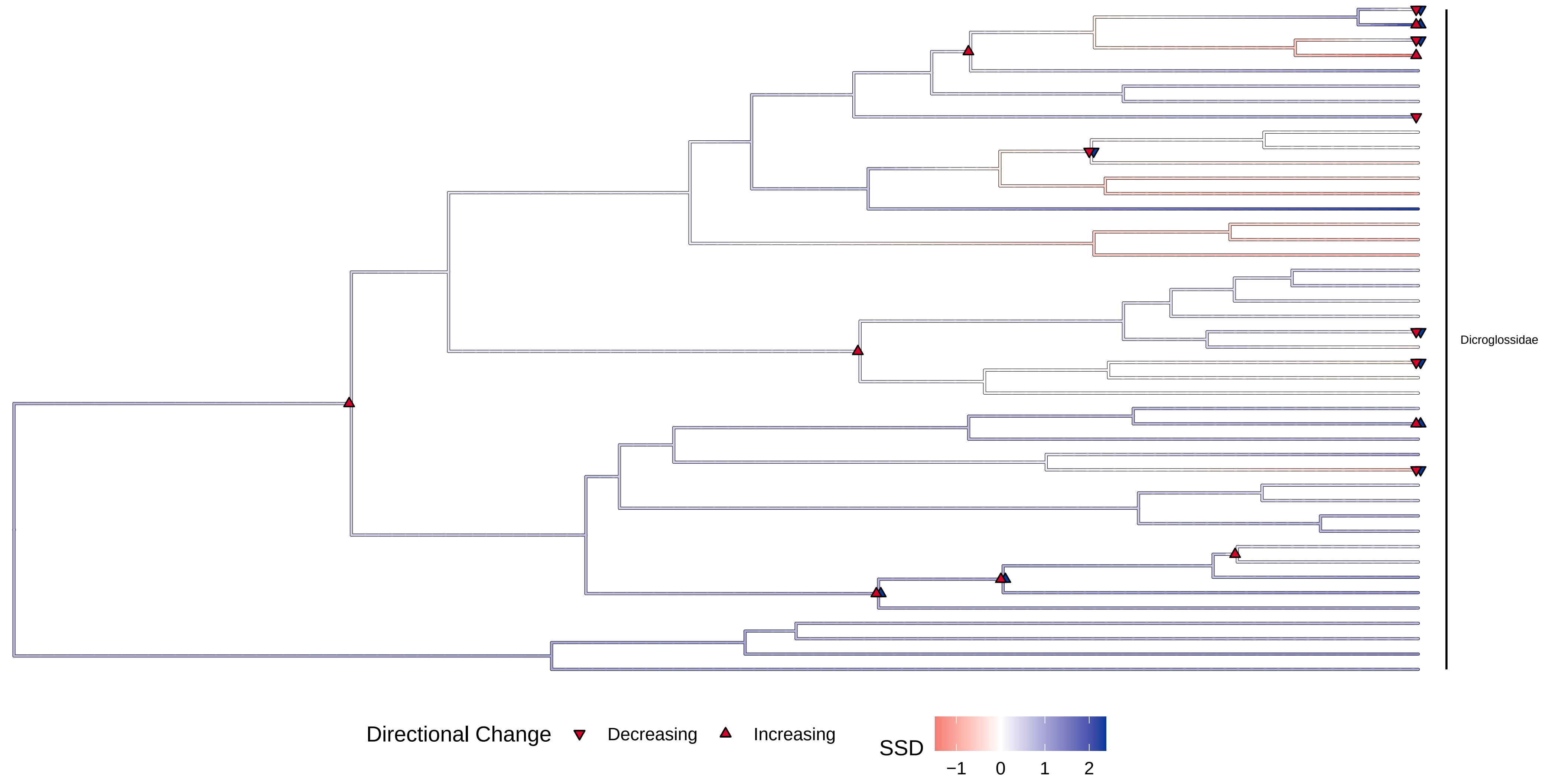

Amphibians

Discoglossoidea

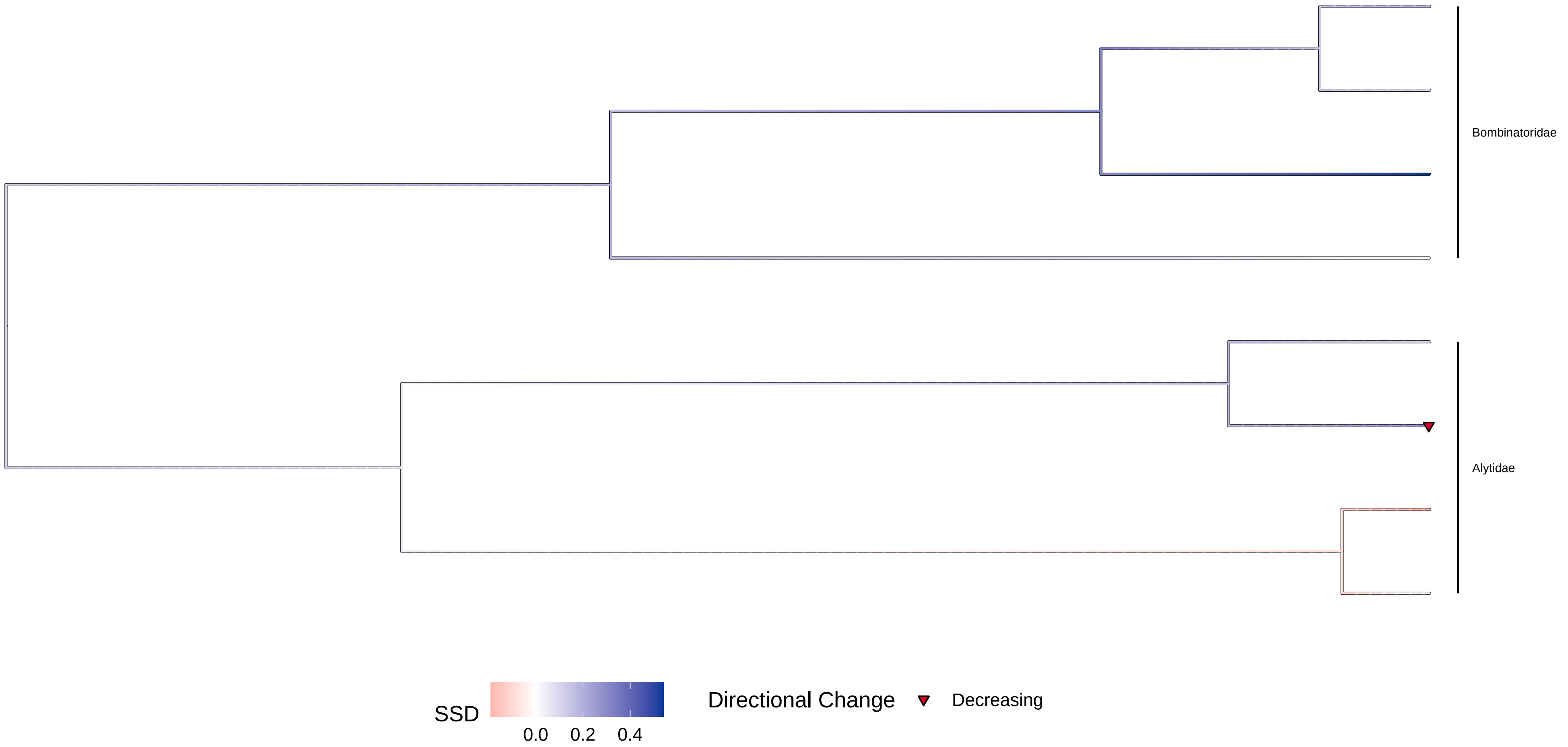

Amphibians

Gymnophiona

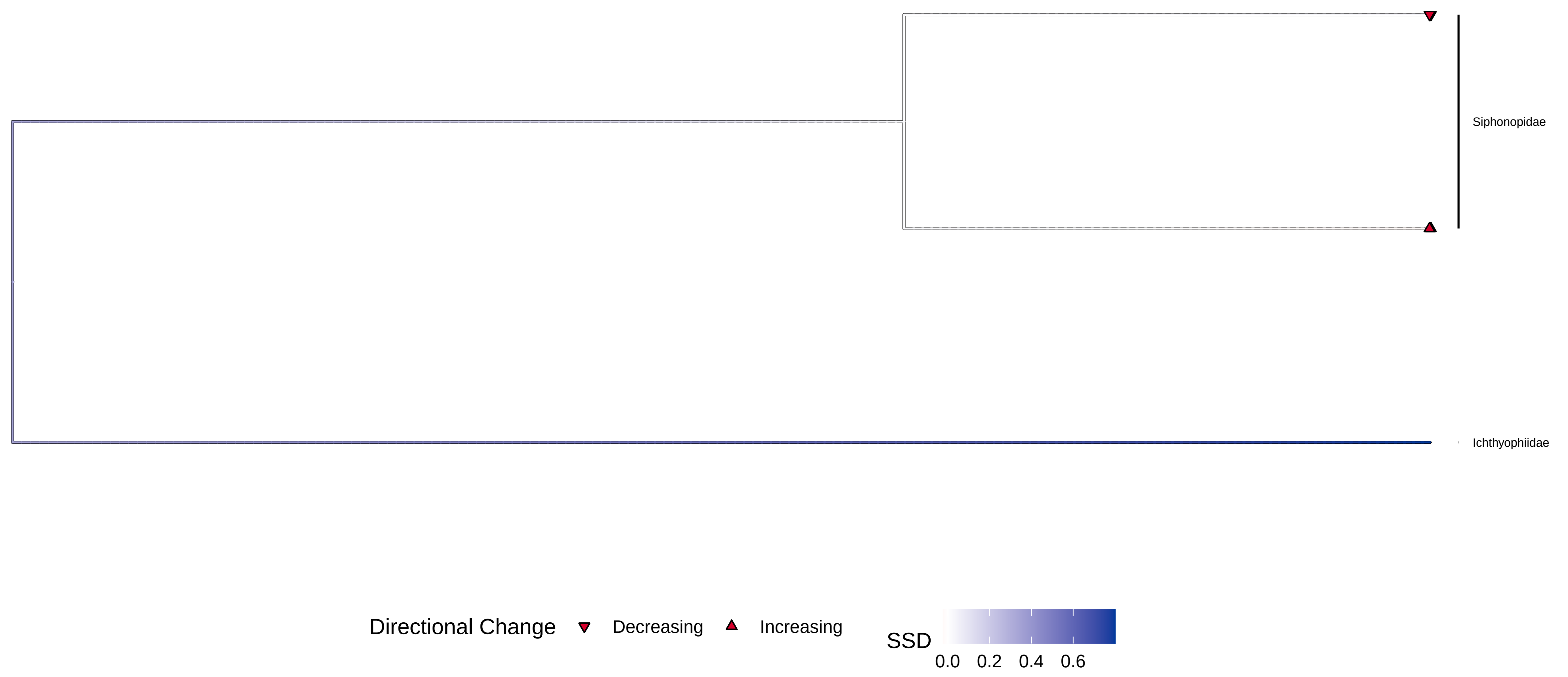

Amphibians

Heleophrynidae

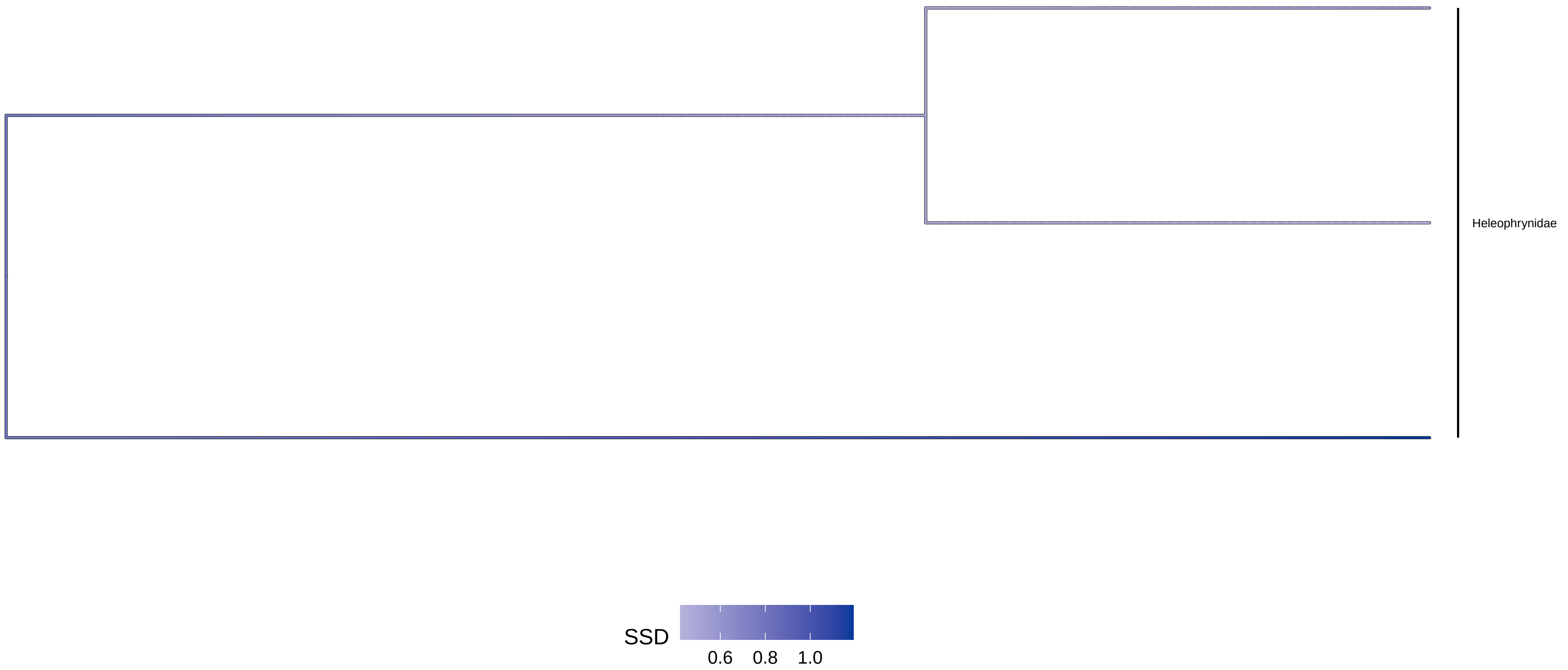

Amphibians

Hemiphractidae

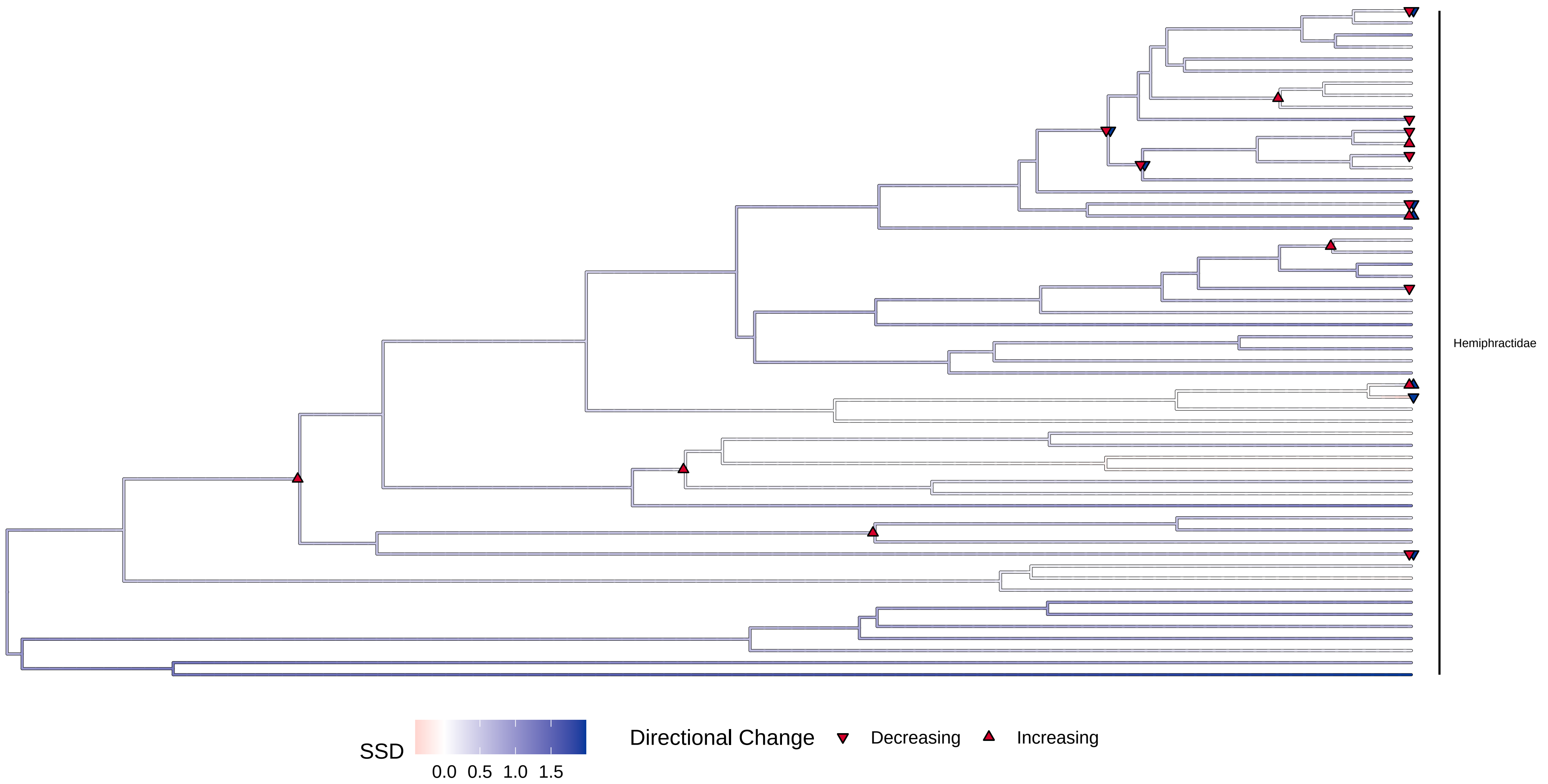

Amphibians

Hylodidae

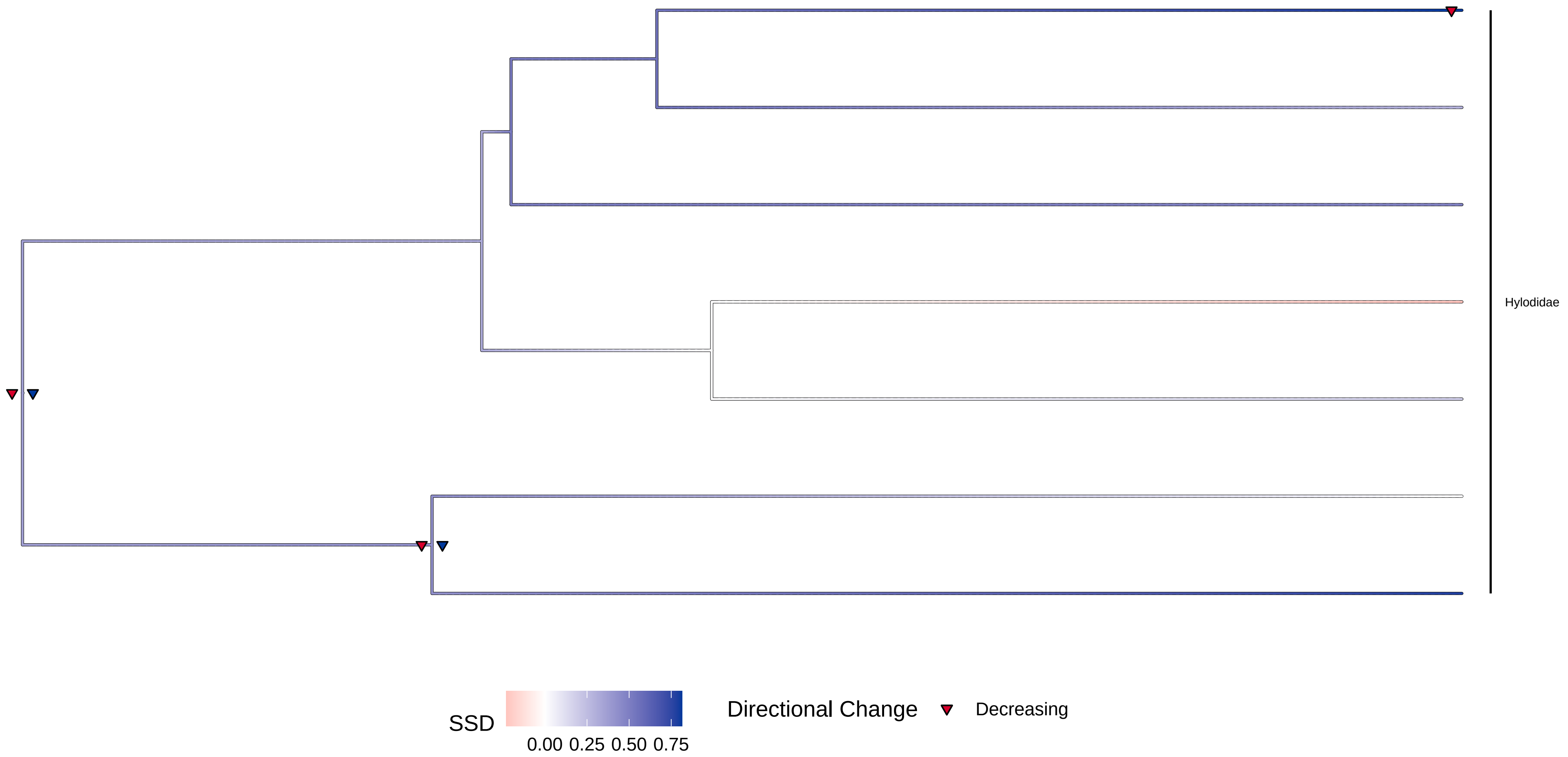

Amphibians

Leiopelmatoidaea

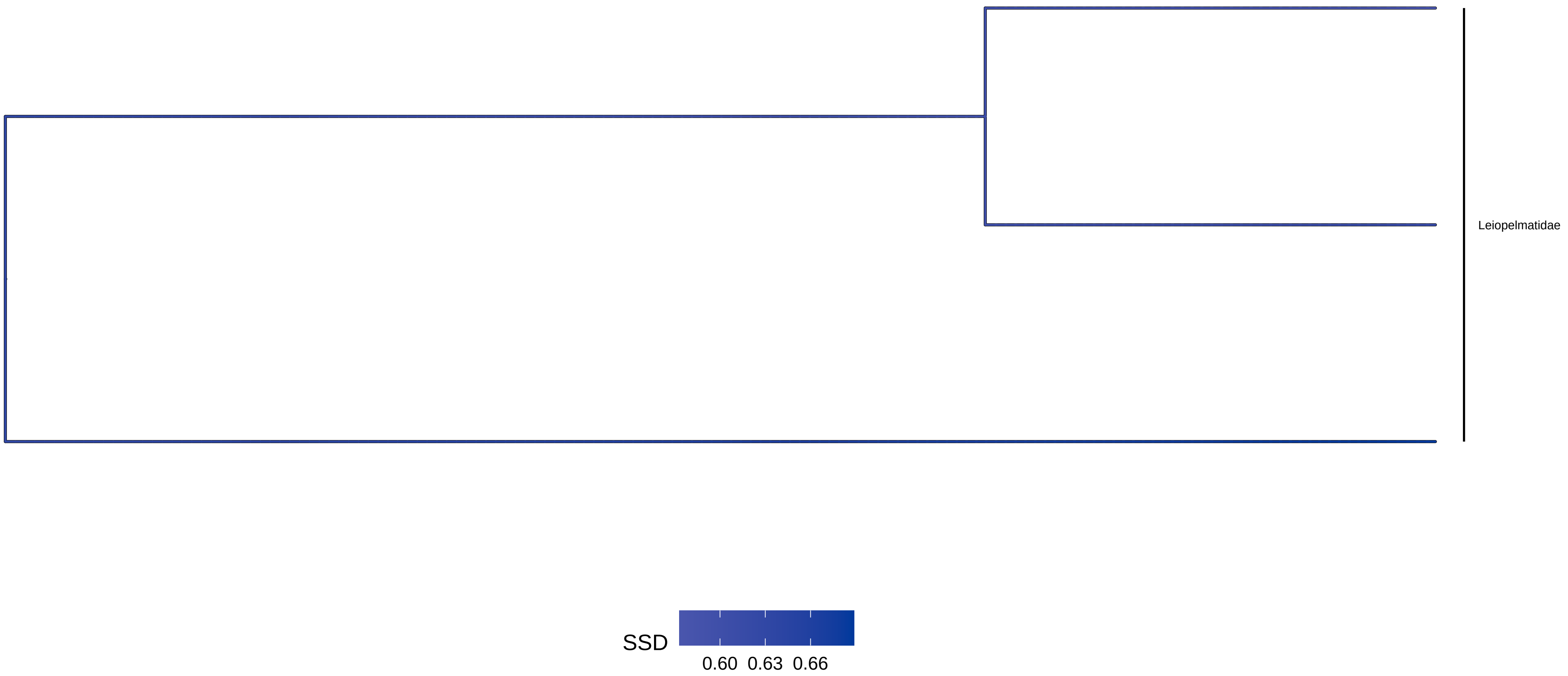

Amphibians

Leiopelmatoidea

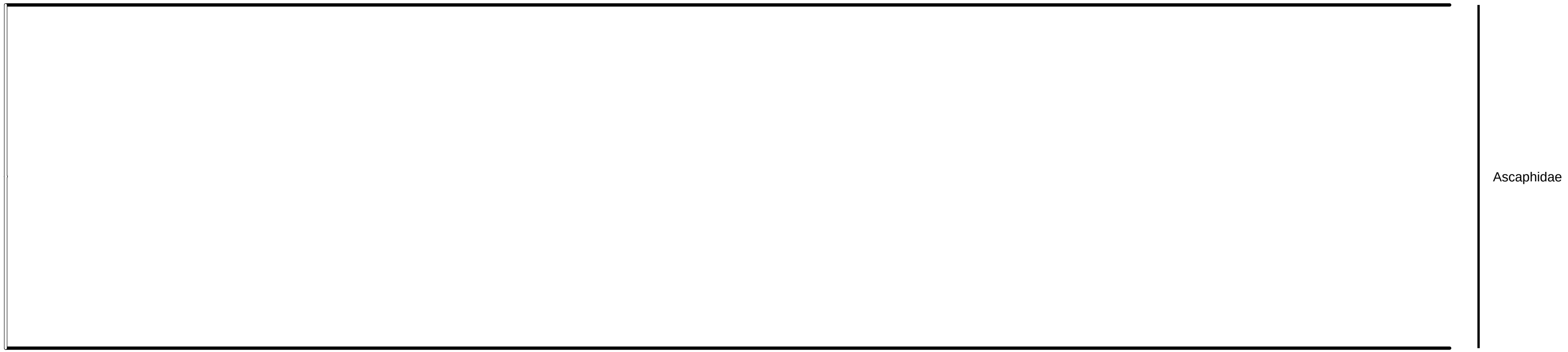

SSD

0.4923092

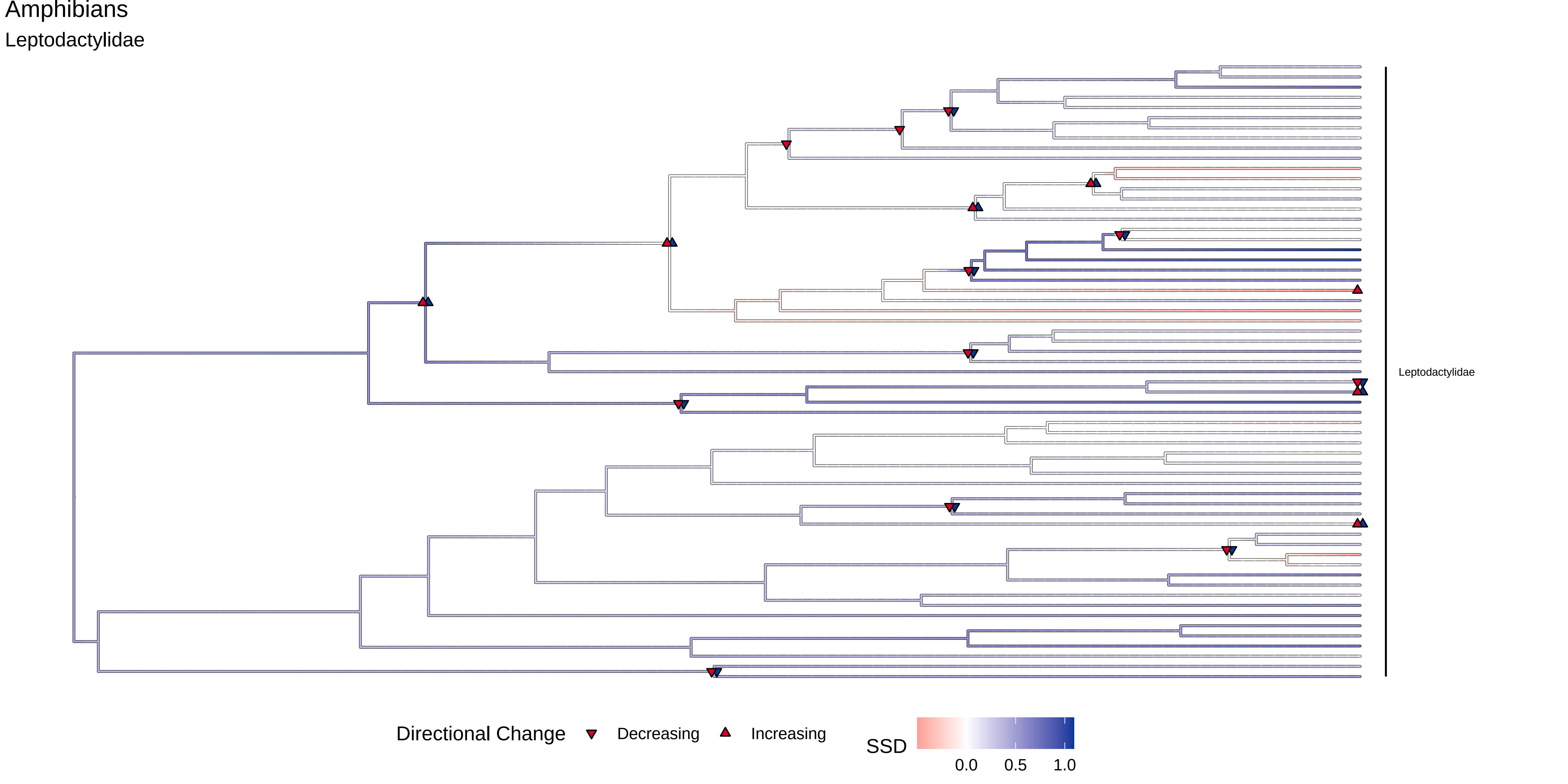

Amphibians
Leptodactylidae
Leptodactylidae
Directional Change
Decreasing
Increasing
SSD
0.0
0.5
1.0

Amphibians

Mantellidae

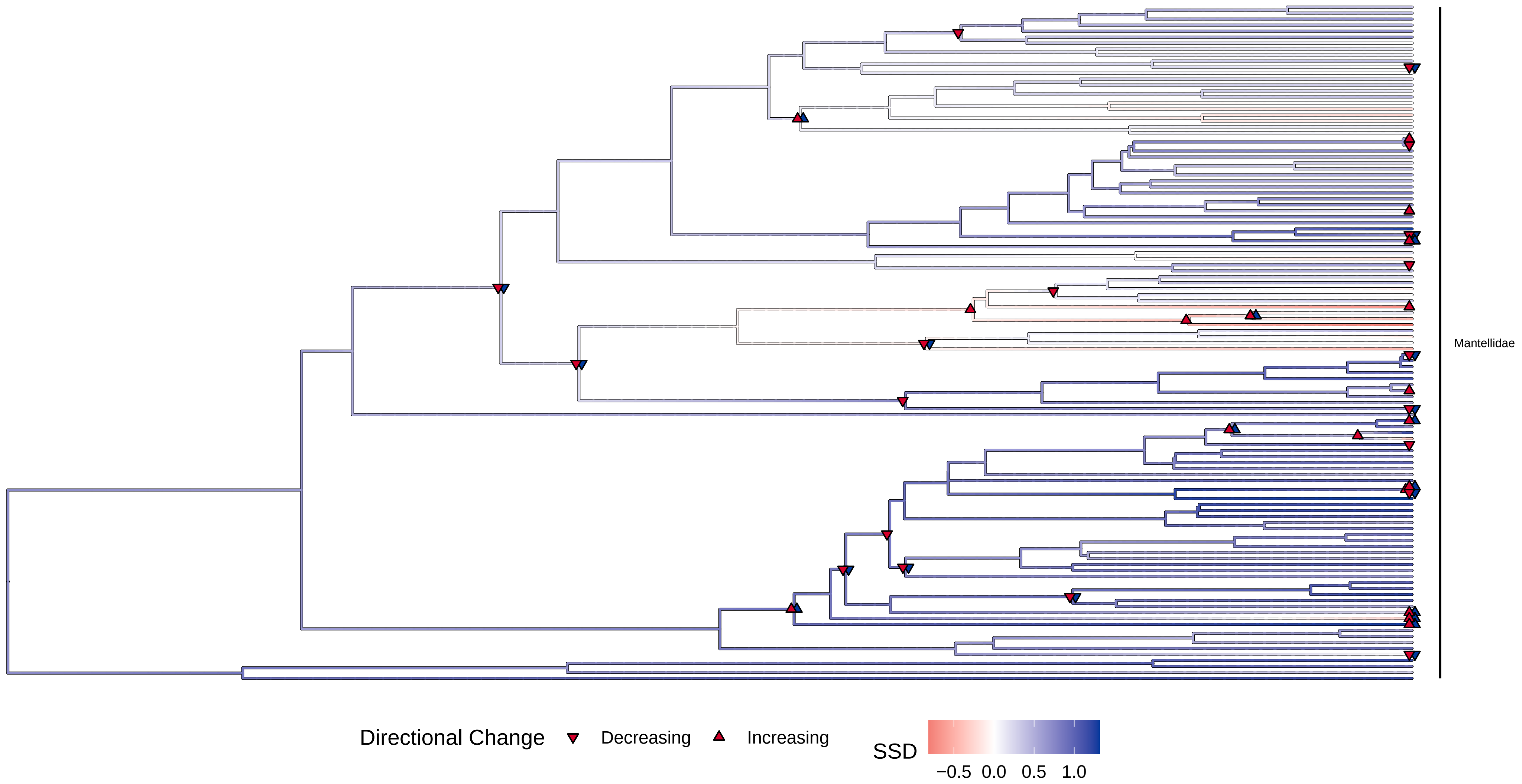

Amphibians

Micrixalidae

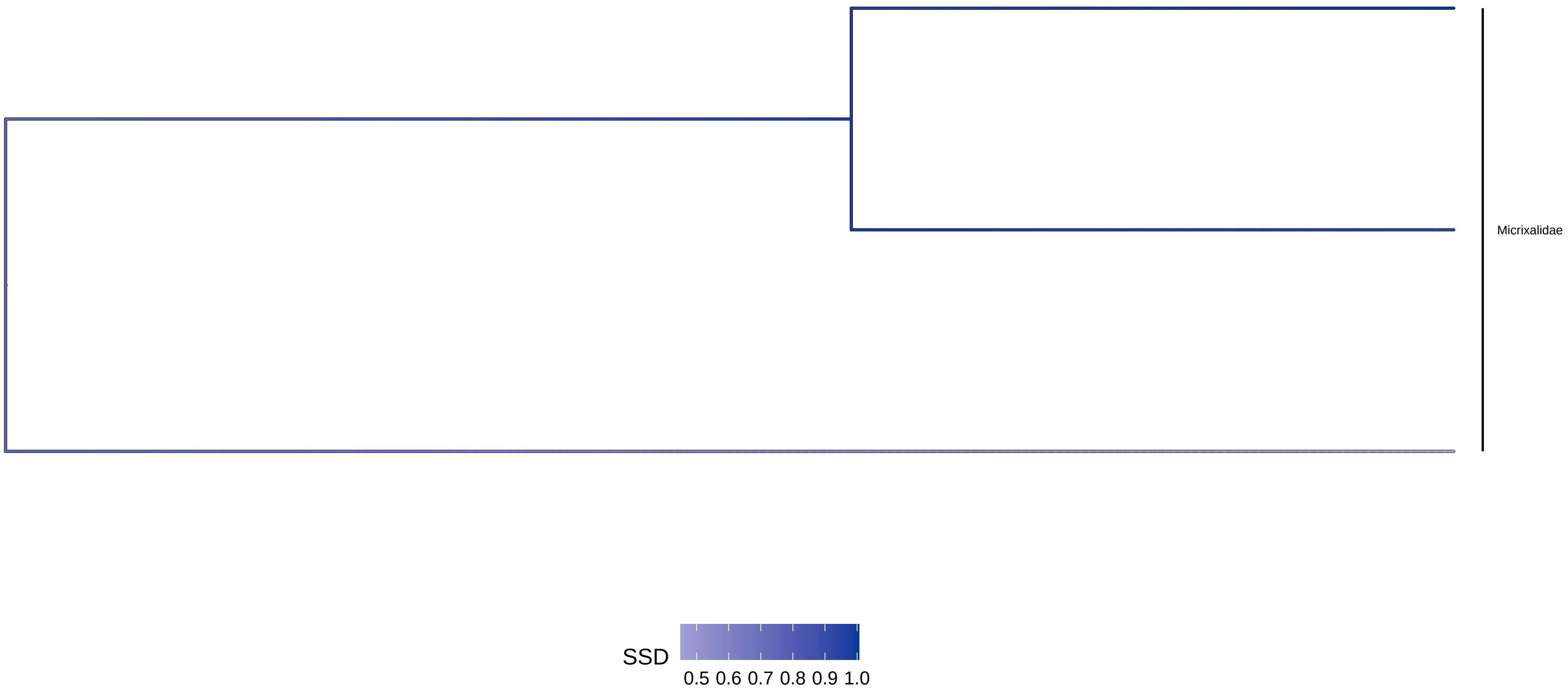

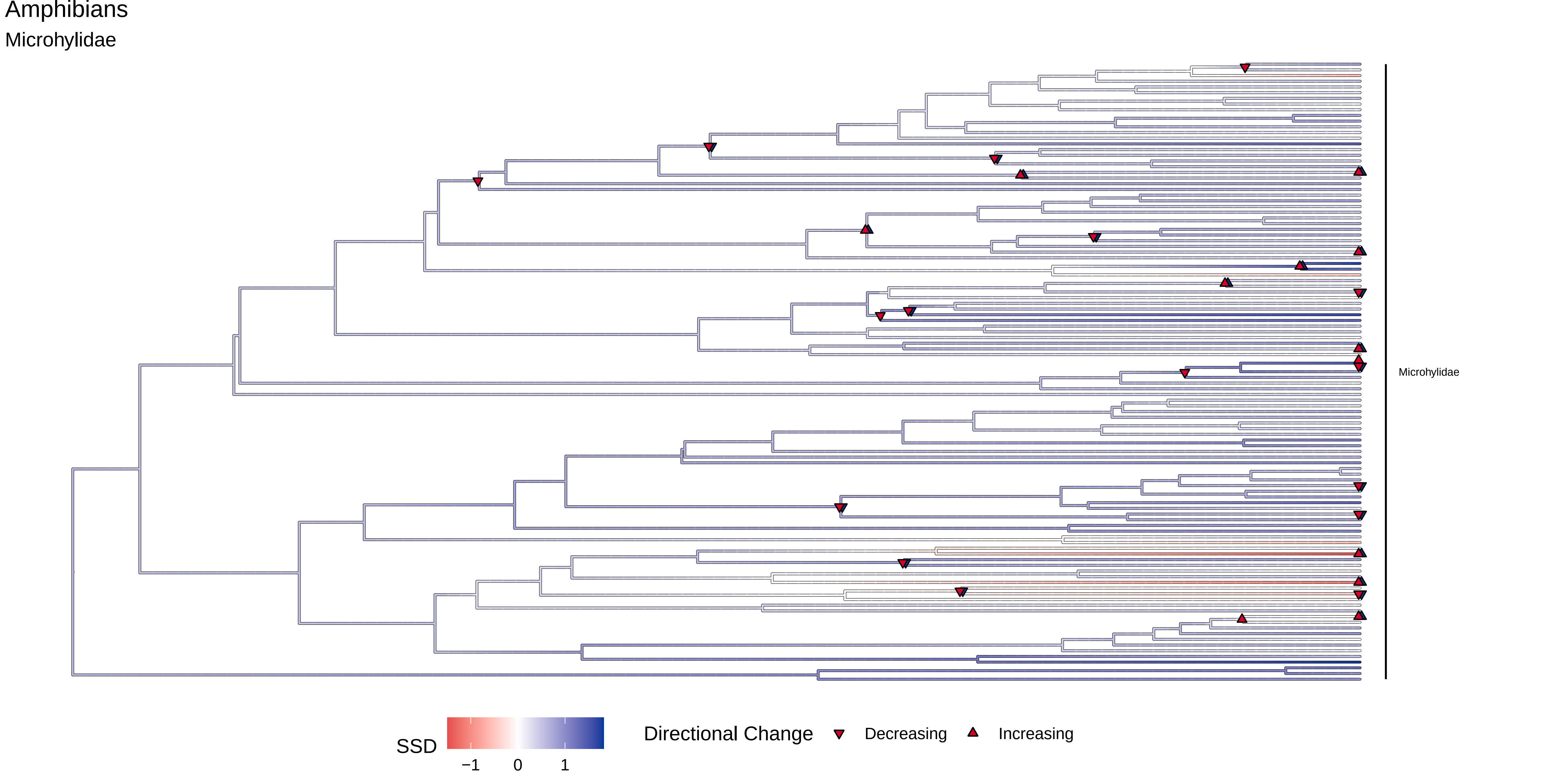
Amphibians
Microhylidae
Microhylidae
SSD
−1
0
1
Directional Change
Decreasing
Increasing

Amphibians

Myobatrachoidea

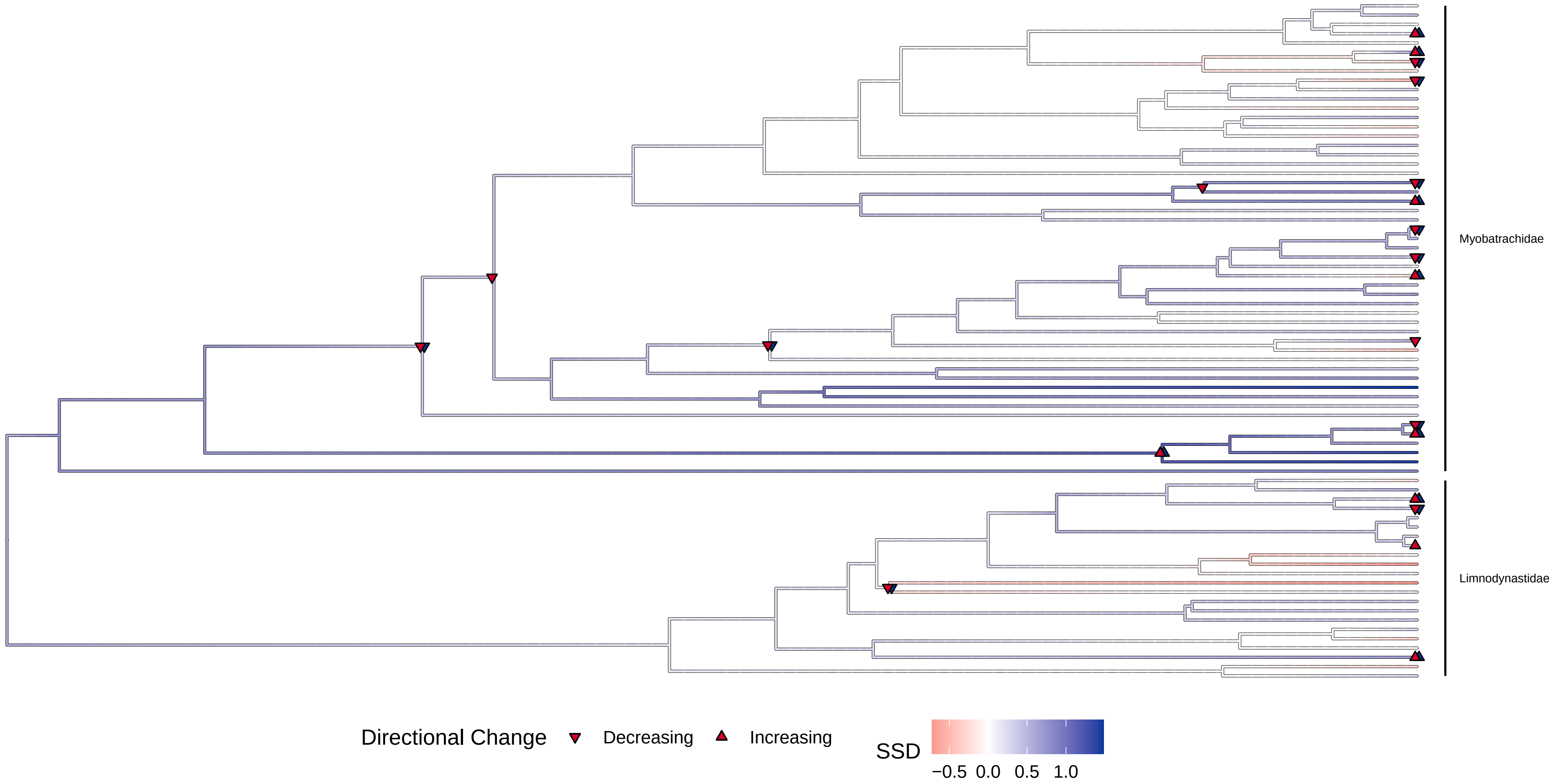

Amphibians

Nyctibatrachidae

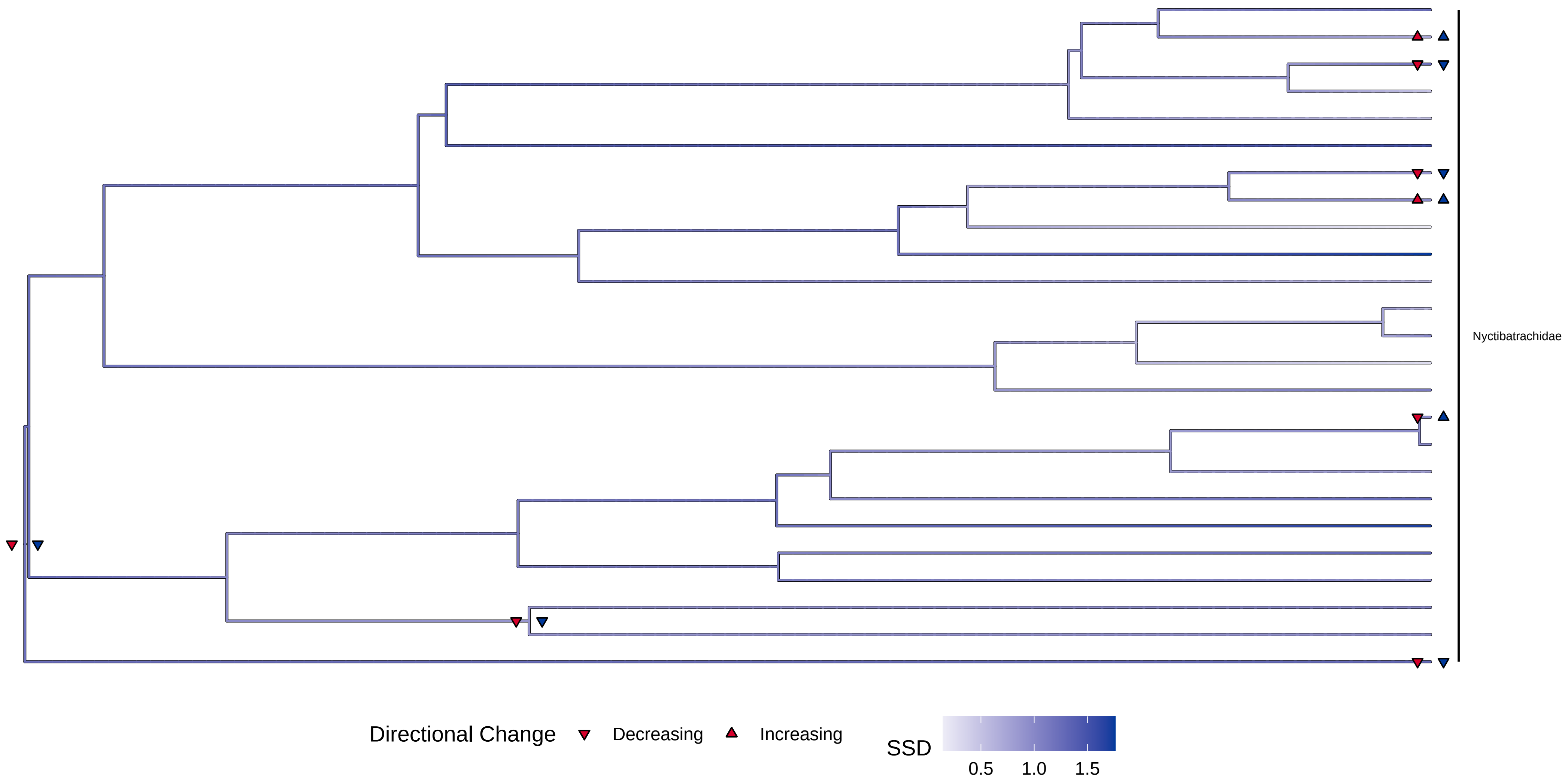

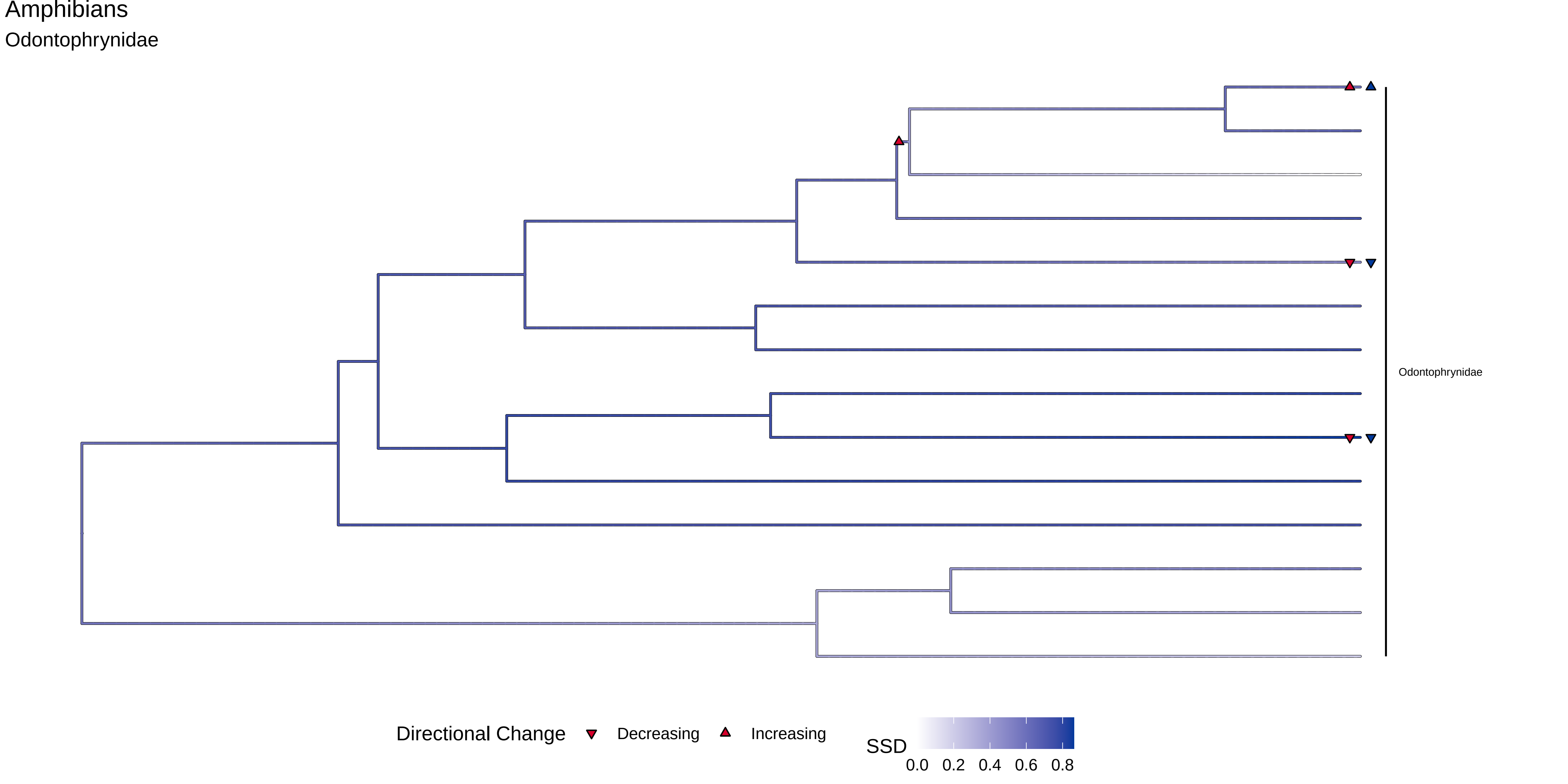

Amphibians
Odontophrynidae
Odontophrynidae
Directional Change
Decreasing
Increasing
SSD
0.0
0.2
0.4
0.6
0.8

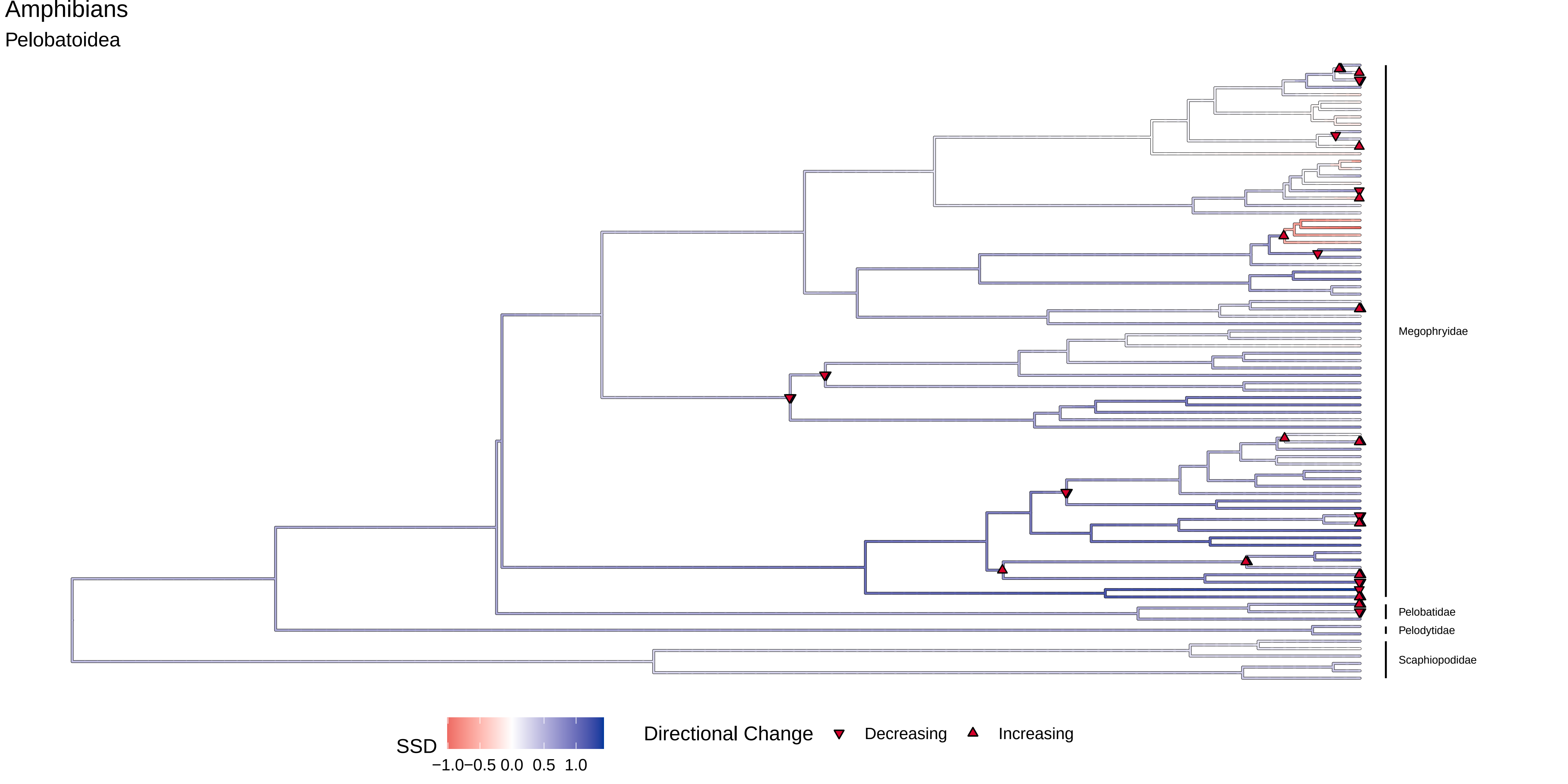

Amphibians
Pelobatoidea
Megophryidae
Pelobatidae
Pelodytidae
Scaphiopodidae
SSD
−1.0 −0.5 0.0 0.5 1.0
Directional Change
Decreasing
Increasing

Amphibians

Petropedetidae

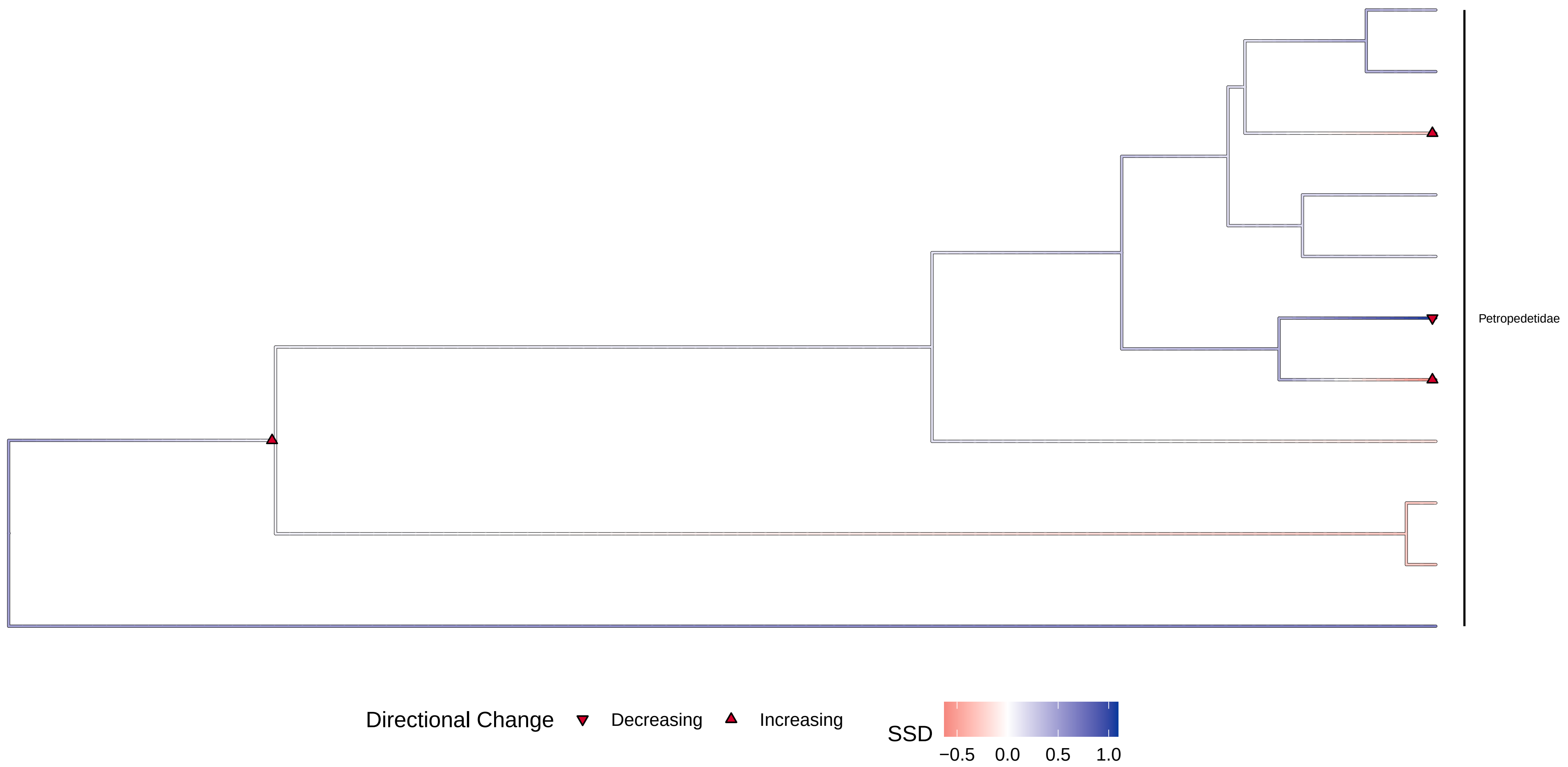

Amphibians

Phrynobatrachidae

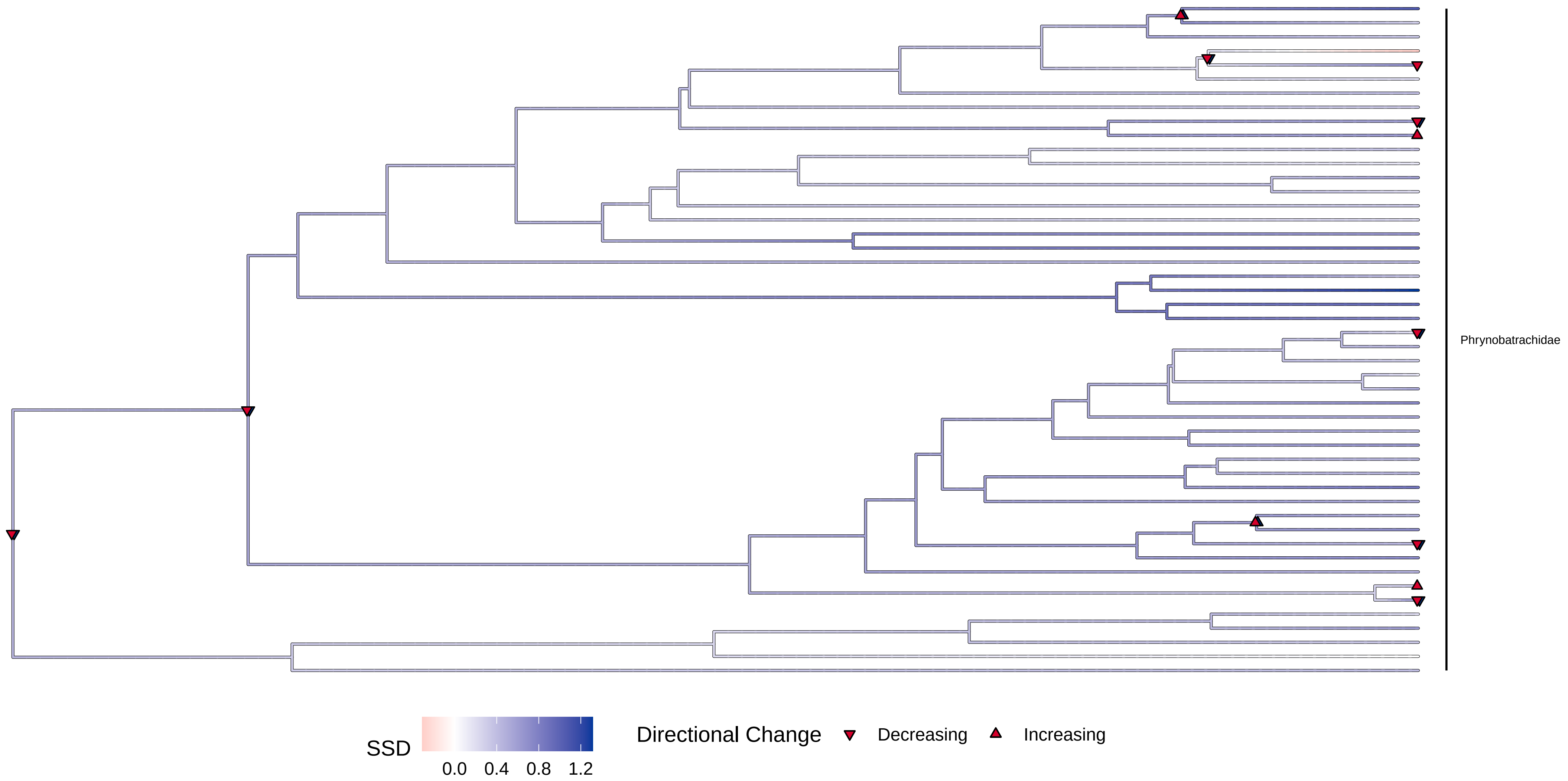

Amphibians

Plethodontidae

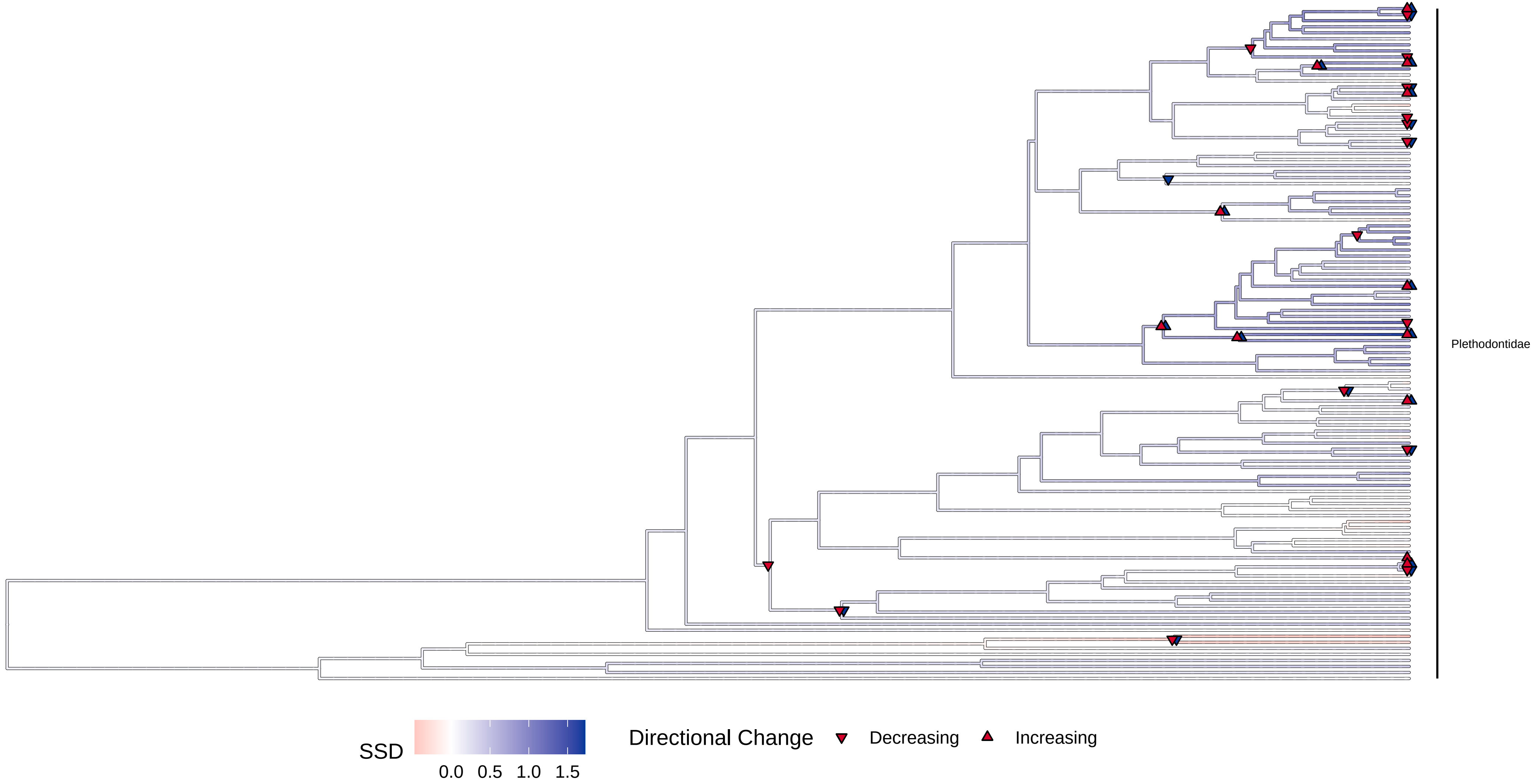

Amphibians

Ptychadenidae

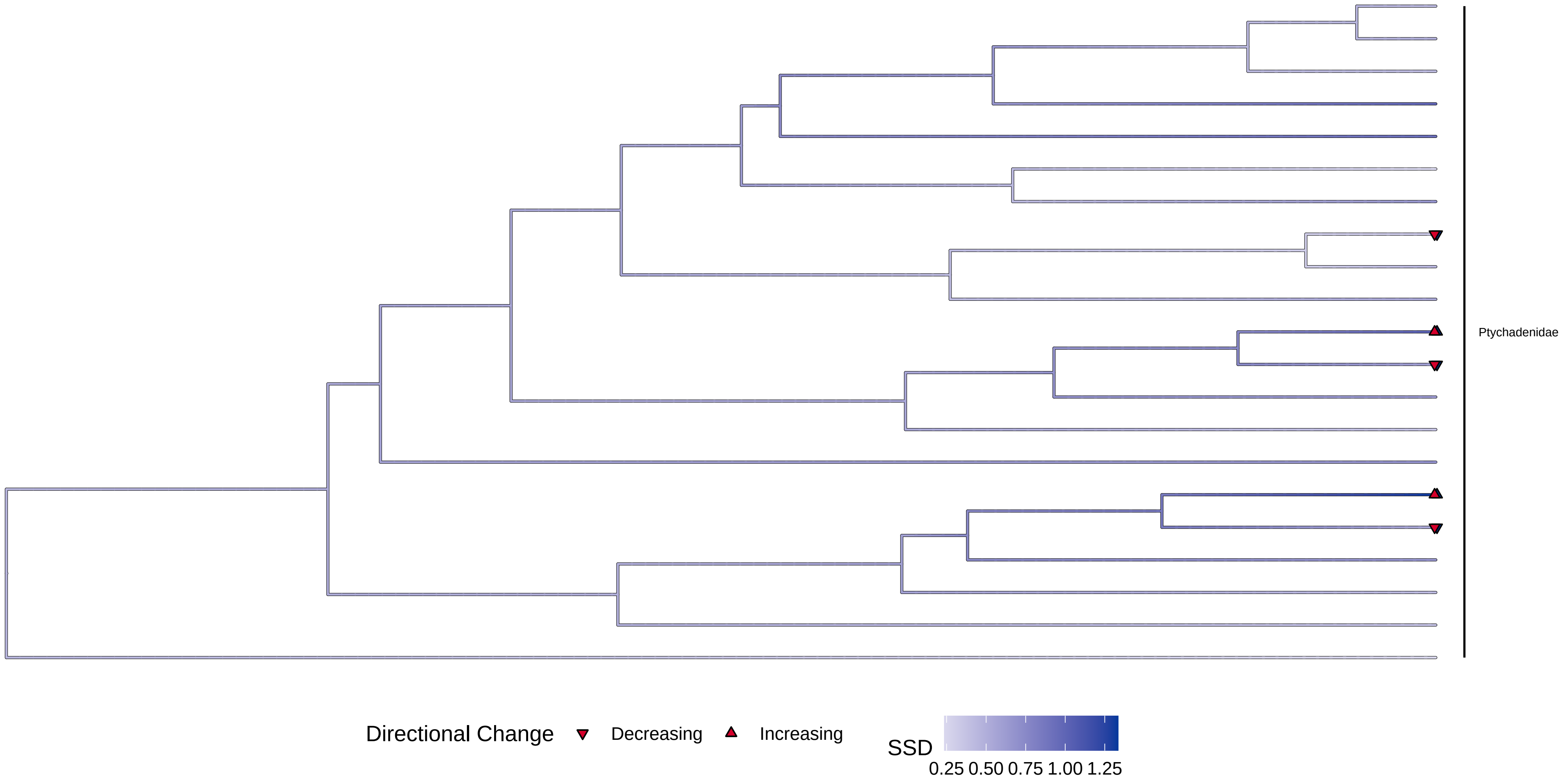

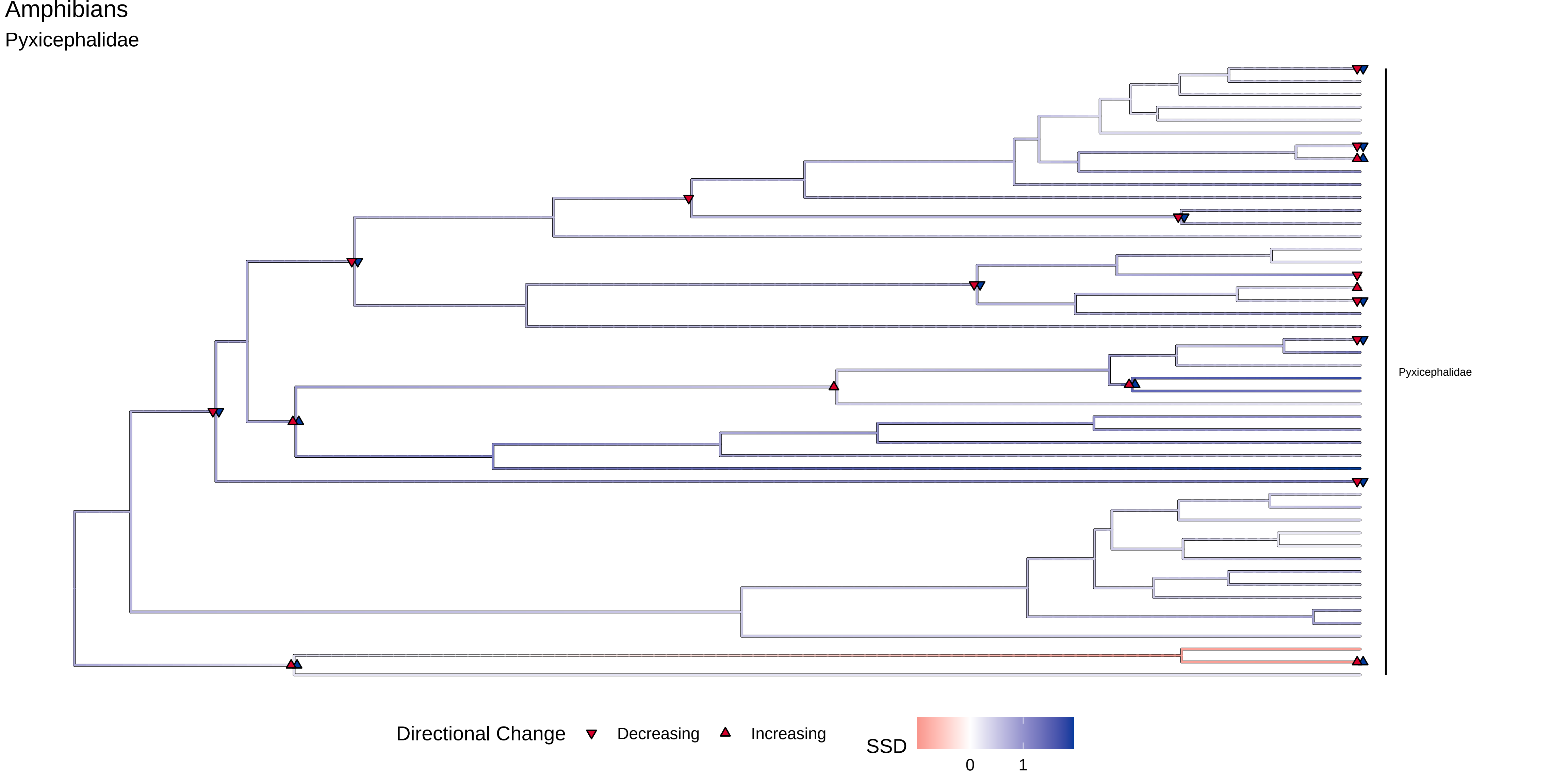

Amphibians
Pyxicephalidae
Pyxicephalidae
Directional Change
Decreasing
Increasing
SSD
0
1

Amphibians

Ranixalidae

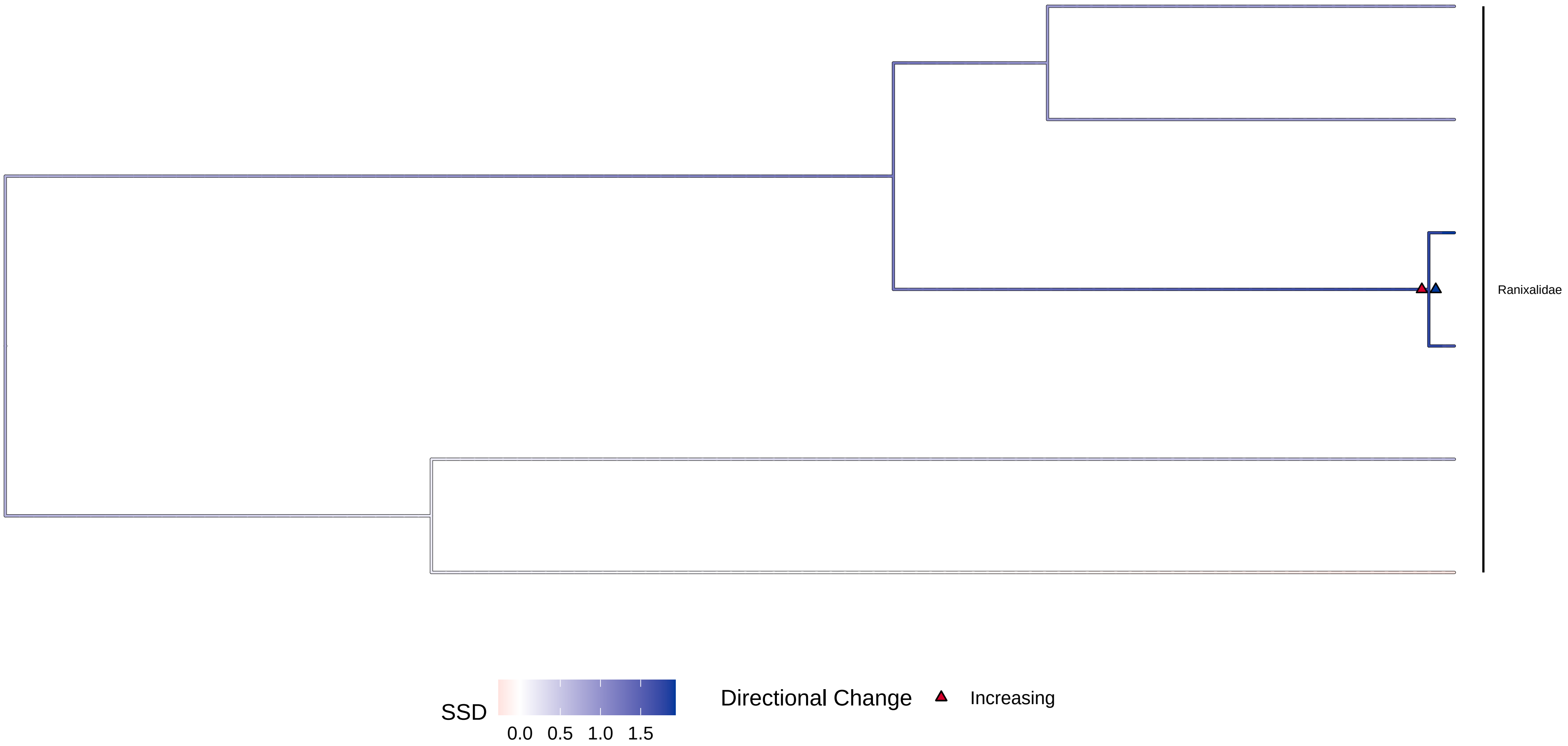

Amphibians

Rhacophoridae

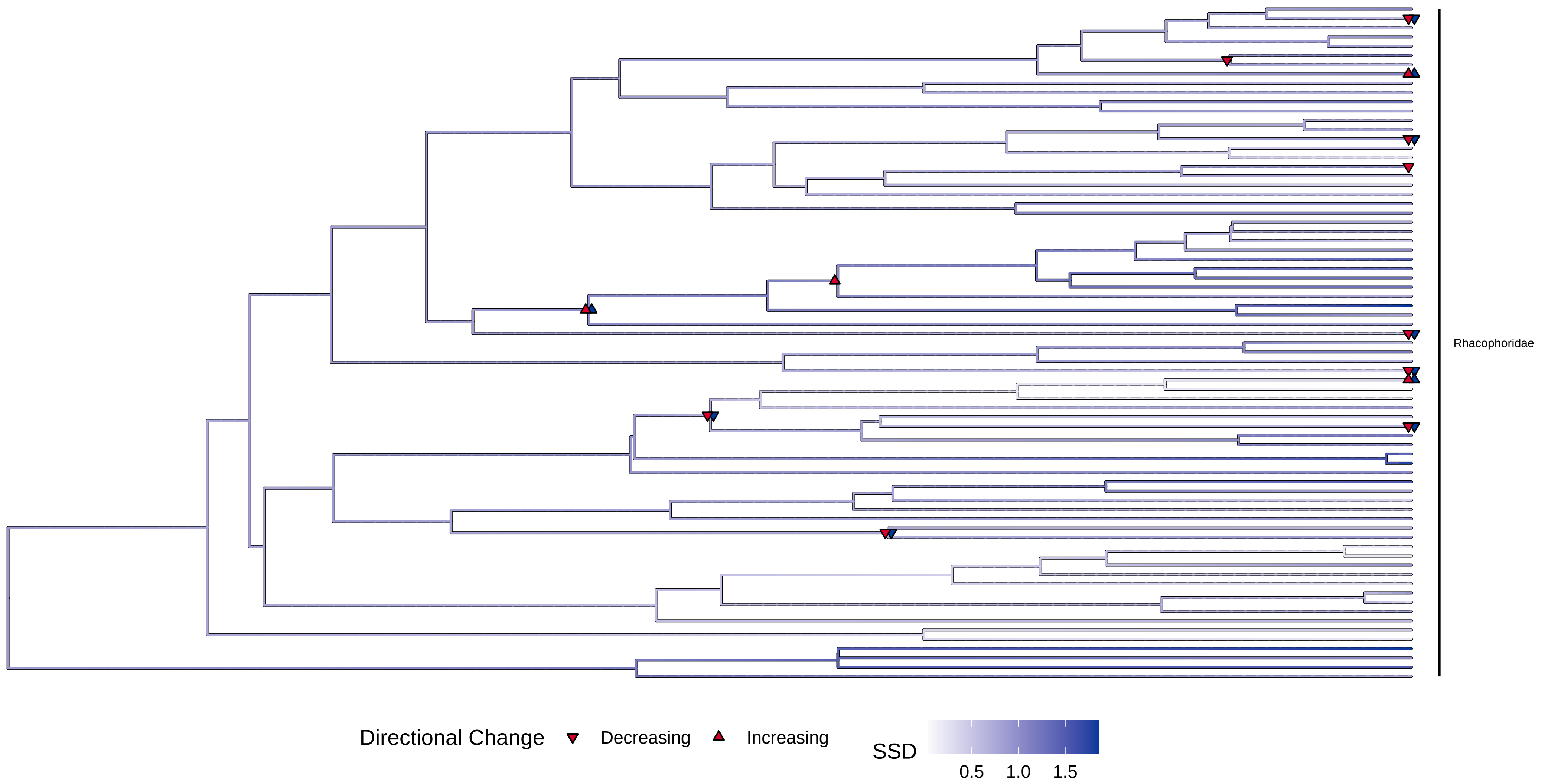

Amphibians

Rhinodermatidae

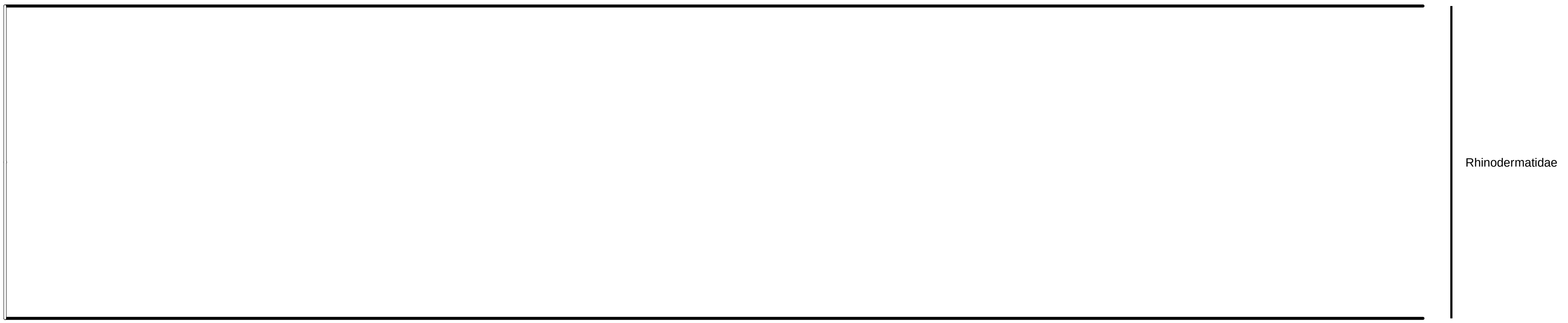

SSD

0.005428376

Amphibians

Salamandridae

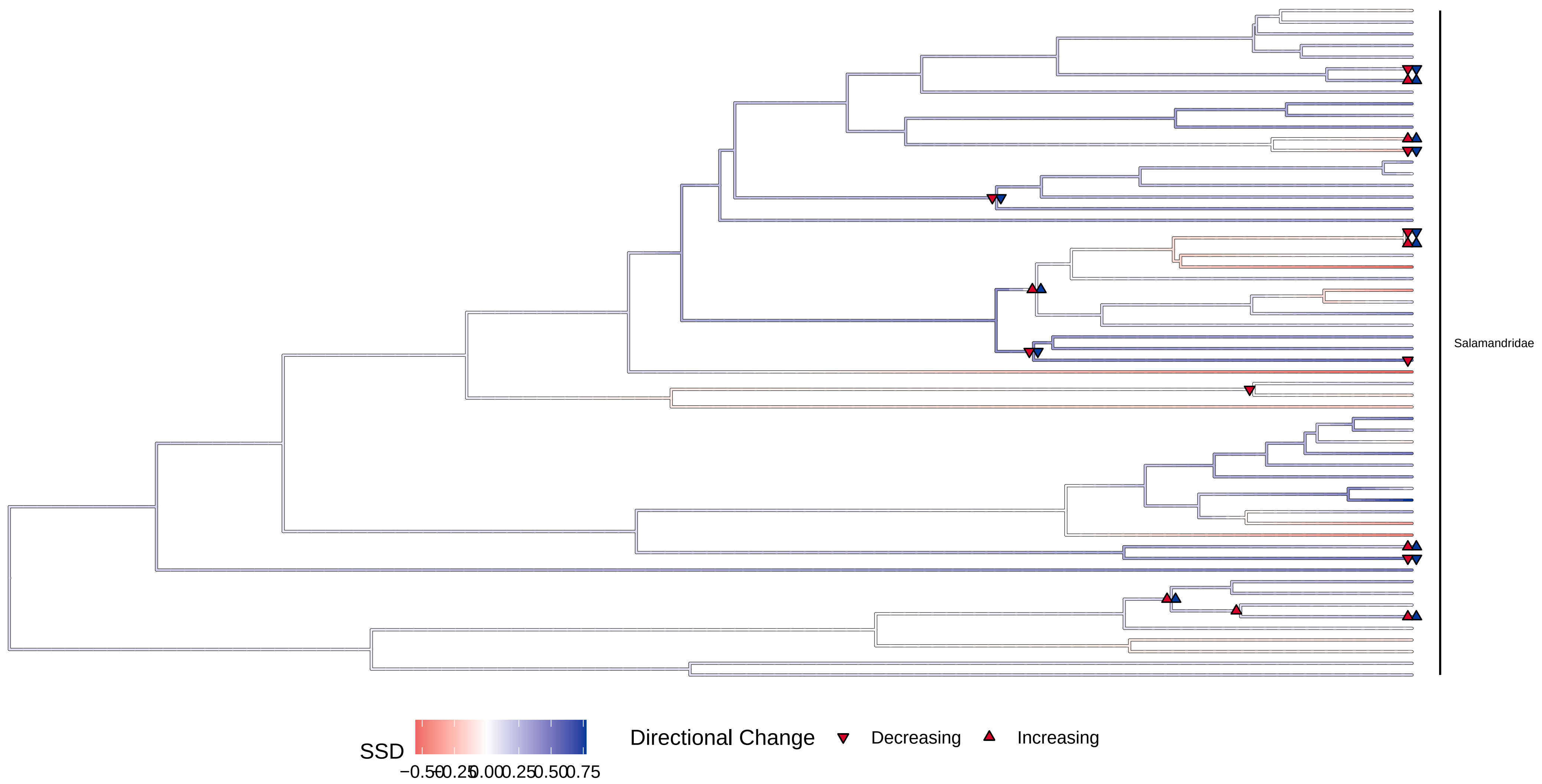

Amphibians
Sooglossidae

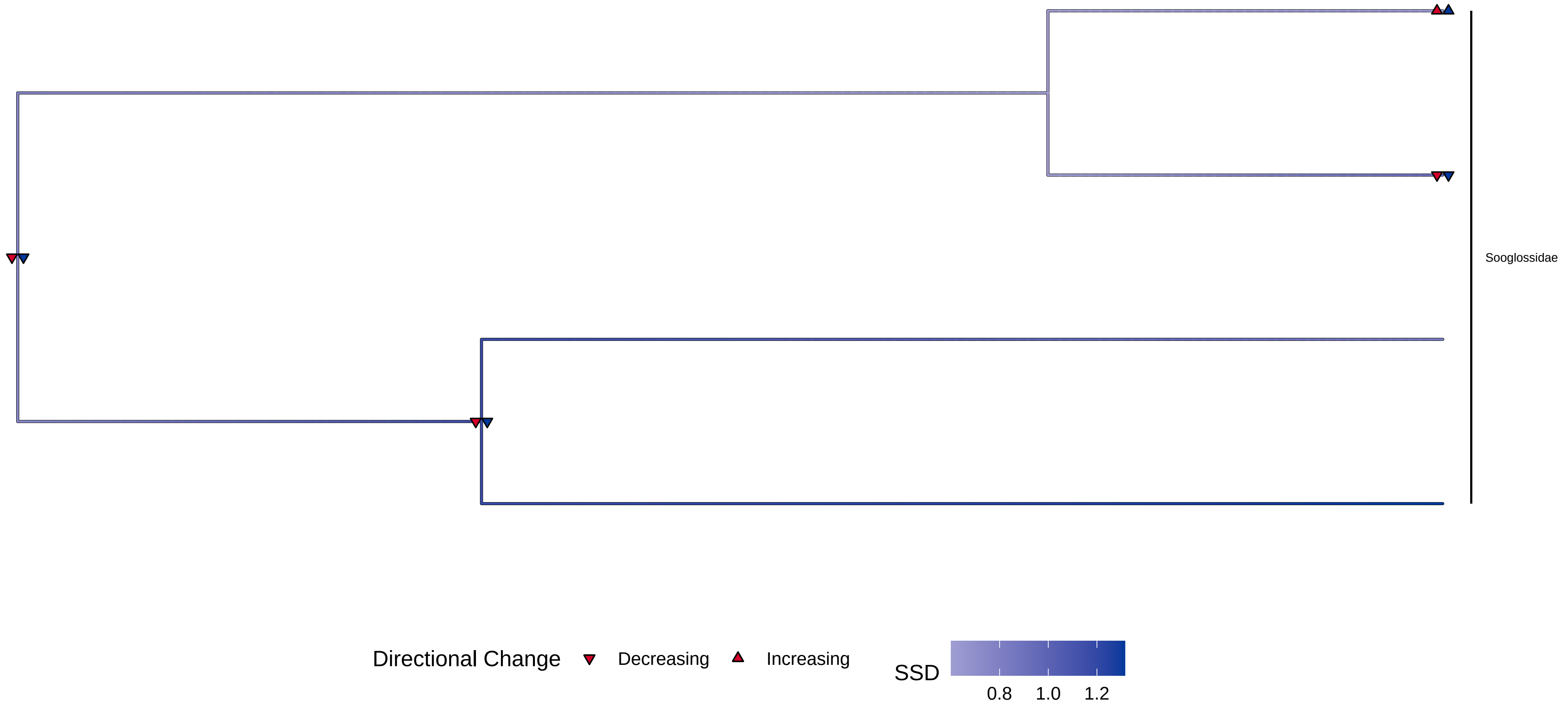

Amphibians

Telmatobiidae

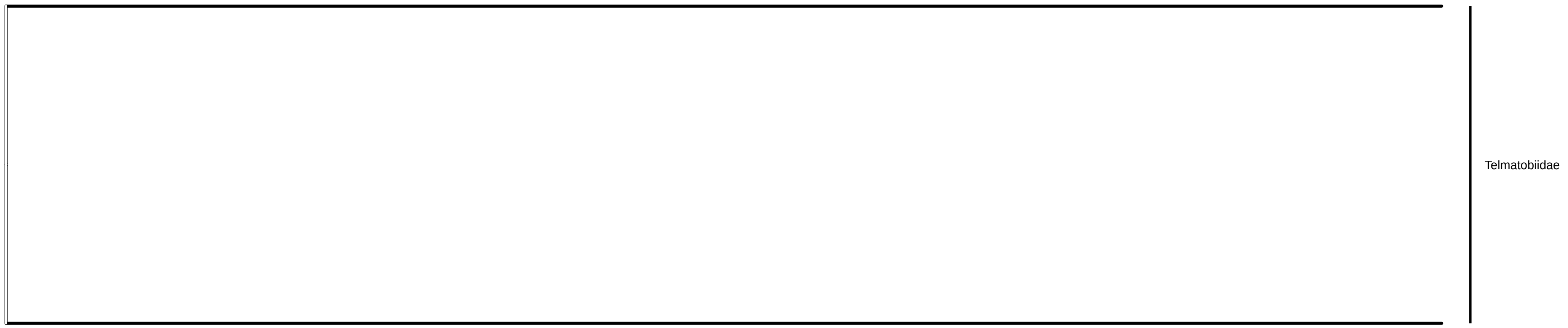

SSD

0.1809634

Amphibians

Hylidae

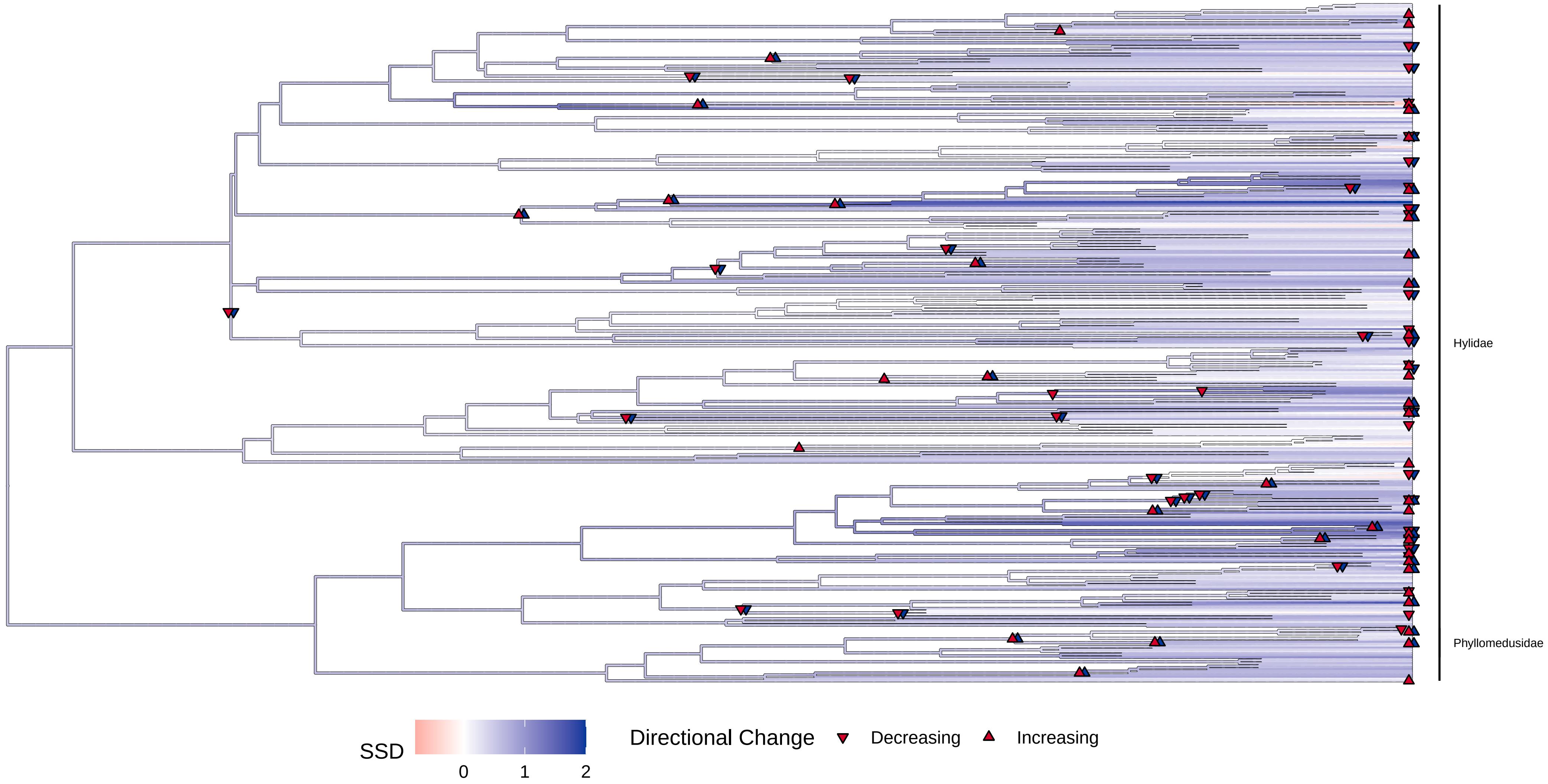

Amphibians

Pipoidea

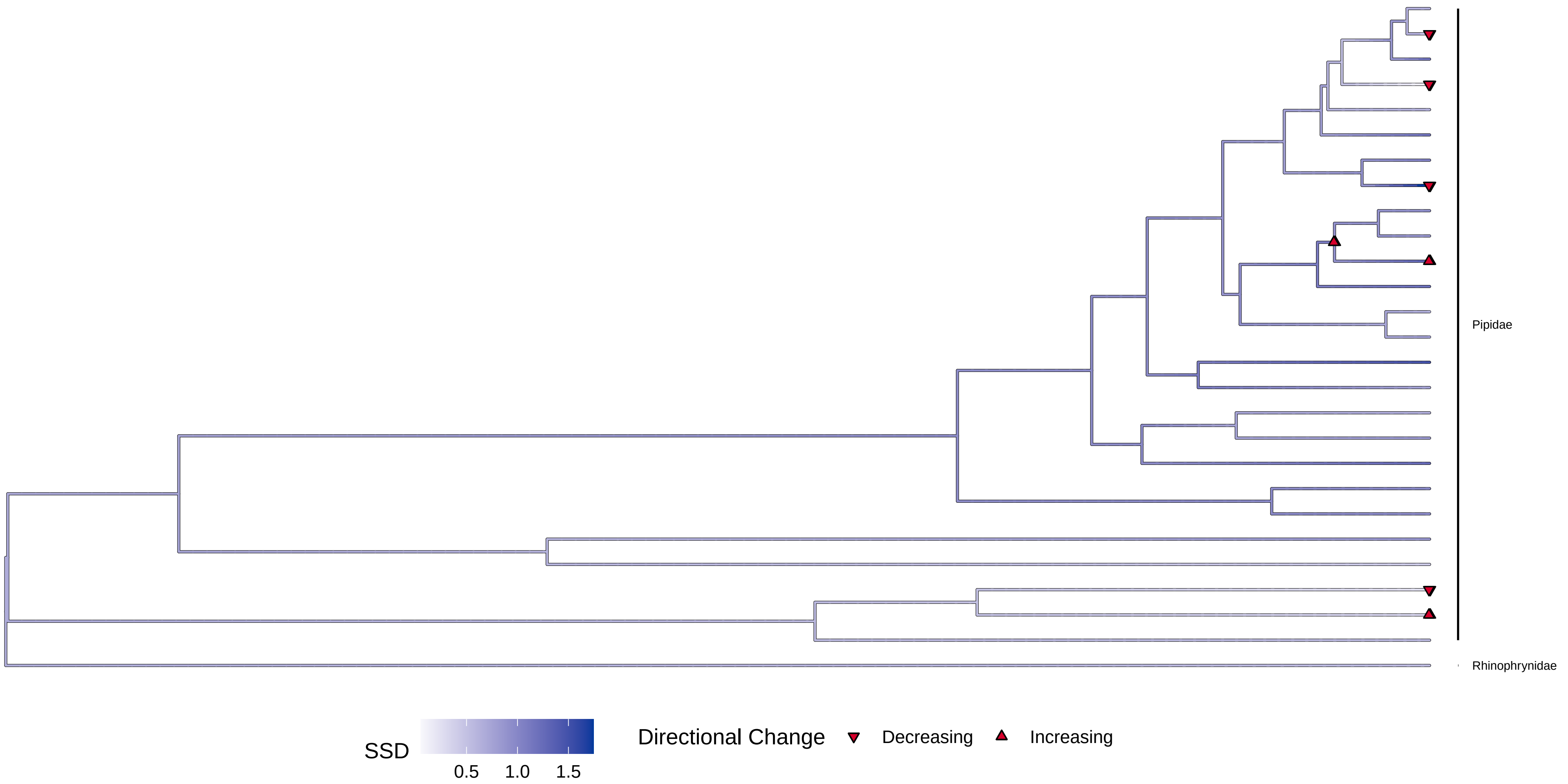

Amphibians

Ranidae

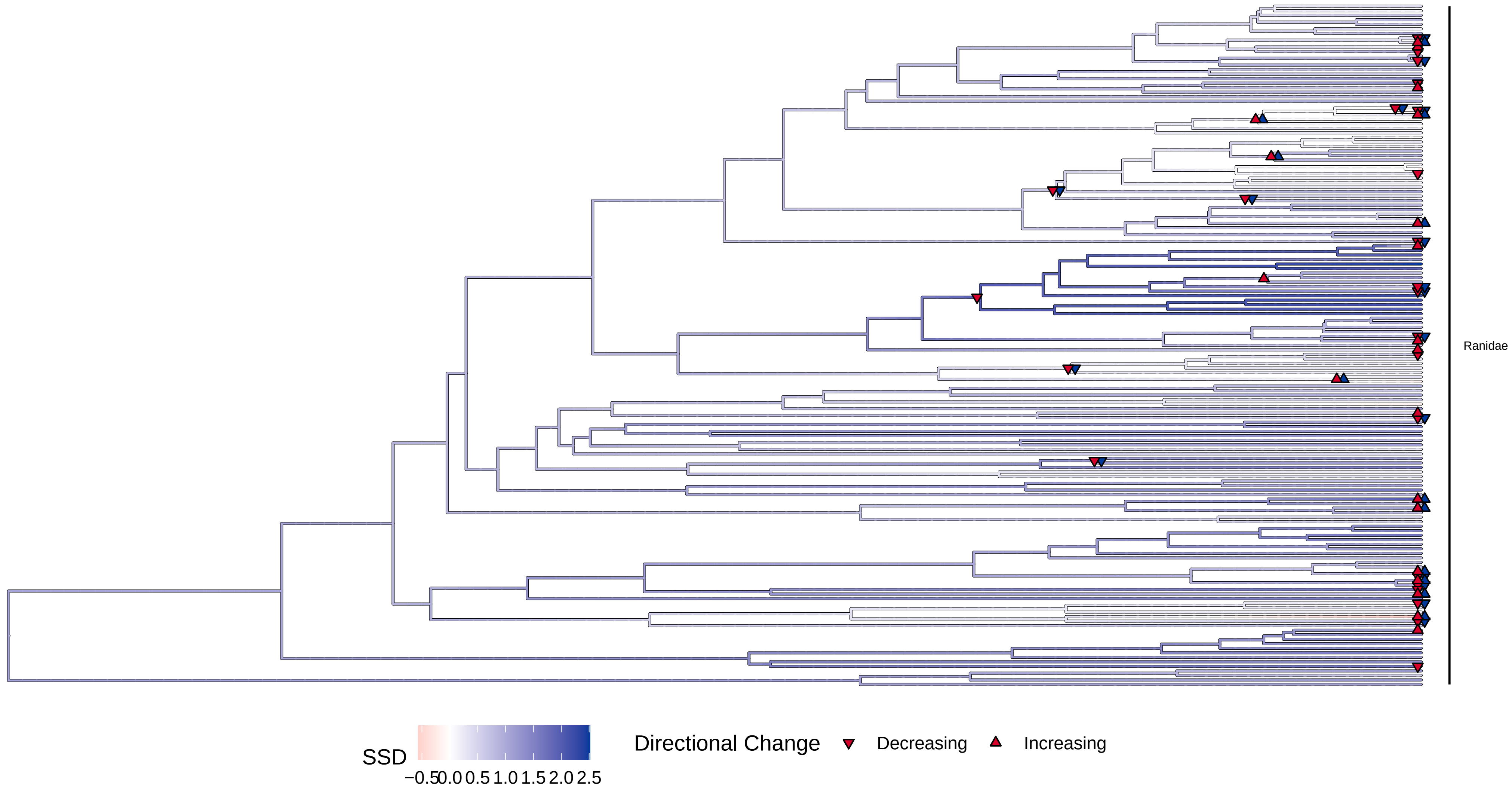

Supplement: Supplementary file 1 — Supplementary Tables 1–5 and Supplementary Information 2. [file 41559_2024_2600_MOESM1_ESM.pdf]
